# Cobalt-catalyzed double hydroboration of pyridines†

**DOI:** 10.1039/d3sc05418g

**Published:** 2024-03-08

**Authors:** Finn Höeg, Lea Luxenberger, Andrey Fedulin, Axel Jacobi von Wangelin

**Affiliations:** a Dept of Chemistry, University of Hamburg Martin Luther King Pl. 6 20146 Hamburg Germany axel.jacobi@uni-hamburg.de; b Philipps-University of Marburg Hans-Meerwein-Str 4 35043 Marburg Germany; c University of Regensburg Universitätsstr 31 93053 Regensburg Germany

## Abstract

Cobalt(ii) complexes were prepared from a modular phosphinopyridonate platform and applied to the hydroboration of pyridines. The synthetically useful, yet challenging, double hydroboration toward tetrahydropyridine derivatives was successfully performed with high activity and regiocontrol. This new method enabled the direct synthesis of *N*-heterocyclic allylic boronates from commercial pyridines and pinacolborane (HBpin). One-pot acetylation afforded the bench-stable borylated *N*-acetyl tetrahydropyridines in good yields. The synthetic utility of this procedure was demonstrated by a gram-scale double hydroboration–acetylation sequence followed by chemical diversification. Mechanistic experiments indicated metal–ligand cooperativity involving ligand-centered C–H activation and the intermediacy of a cobalt(iii) hydride species.

## Abstract 2

Cobalt phosphinopyridonates enabled the synthetically useful, yet challenging, double hydroboration of pyridines. Mechanistic studies indicated metal–ligand cooperativity, ligand-centered C–H activation and the intermediacy of a cobalt(iii) hydride.

## Graphical abstract

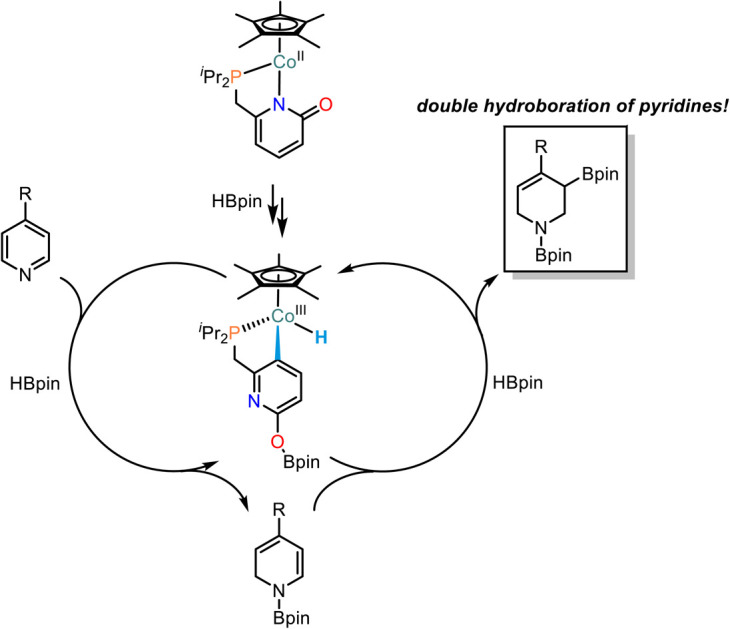

## Introduction

**Scheme 1:**
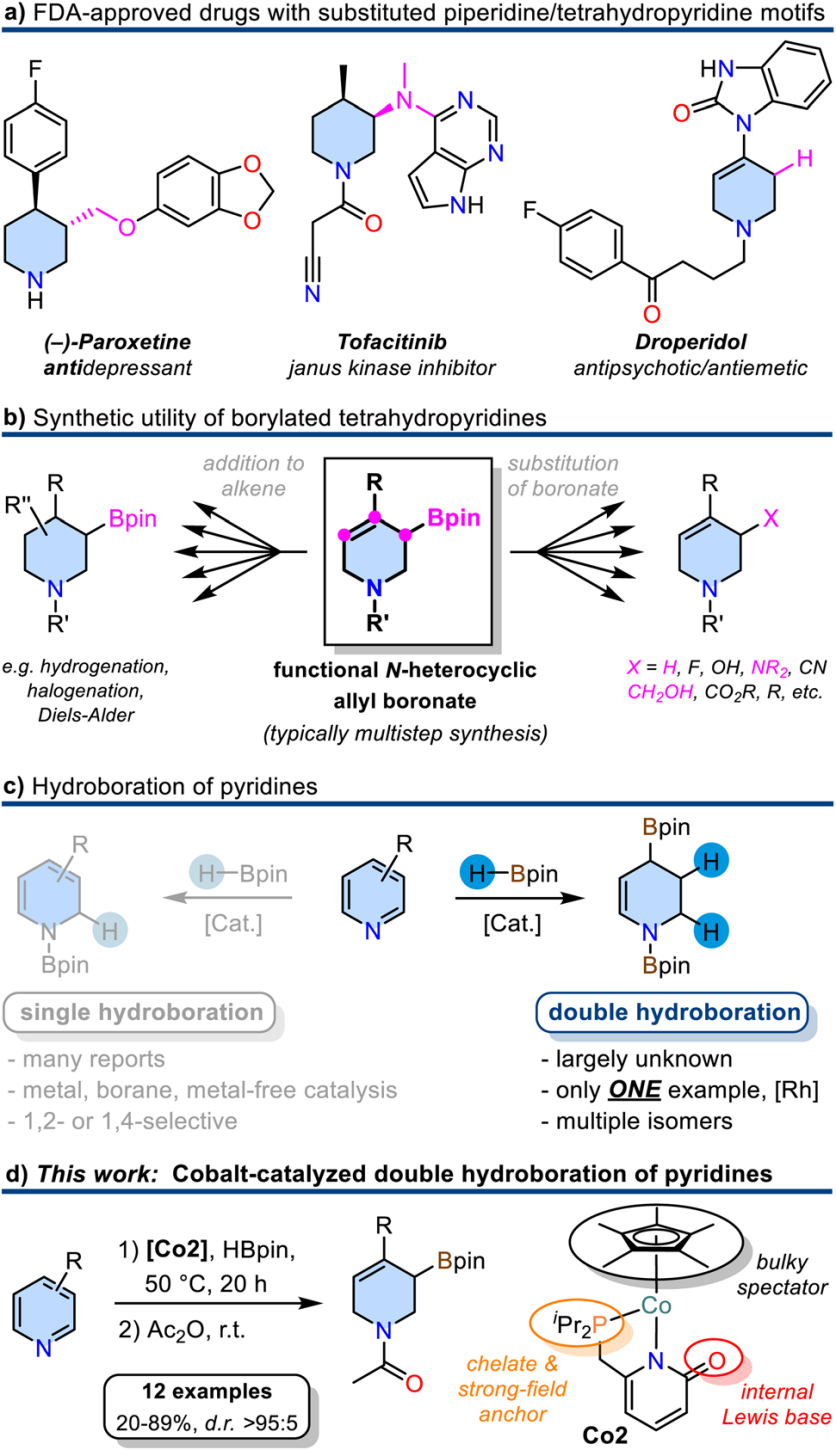
(a) Substituted piperidines and tetrahydropyridines as structural motifs in pharmaceuticals. (b) Synthetic utility of borylated tetrahydropyridines. (c) Methods of single and double hydroboration of pyridines. (d) New development of a cobalt pyridonate catalyzed double hydroboration of pyridines.

Six-membered N-heterocycles such as piperidines and tetrahydropyridines are ubiquitous in natural products and pharmaceuticals (Scheme 1a).^1^ Among the 1086 FDA-approved small-molecule drugs as of 2012, 59% contained *N*-heterocycles.^1^ The availability of straight-forward, robust, and selective synthetic routes to such building blocks is of utmost interest for medicinal chemistry and drug design endeavours. The synthesis of densely functionalized *N*-heterocycles from inexpensive starting materials in one synthetic operation is especially attractive by virtue of the high gain of complexity and ample opportunities for further derivatization. Borylated tetrahydropyridines could fulfill these criteria if they were accessible from pyridines by simple and selective addition reactions of borane reagents as the resultant C–B motif can easily be functionalized under various conditions (Scheme 1b). Typical methods of tetrahydropyridine boronate synthesis include the addition of organometallics to activated pyridine boronic esters,^2^ Tsuji-Trost type allylic substitution of *N*-heterocyclic allyl alcohol derivatives with B_2_pin_2_,^3^ or Pd-catalyzed borylation/isomerization of alkenyl nonaflates.^4,5^ However, these procedures require multiple reaction steps either in the substrate preparation or follow-up reactions. The partial reduction of readily accessible pyridines with organoboranes could provide a most direct alternative approach to *N*-heterocyclic allyl boronates. Selective 1,2- and 1,4-mono-hydroborations of pyridines were studied in great detail with metal catalysts (Rh,^6^ Ru,^7^ La,^8^ Zn,^9^ Th,^10^ Mg,^11–13^ Mn,^14^ Ni,^15,16^ Fe,^17^ Co^18^) and organoboranes^19–21^ as well as metal-free conditions^22–24^ (Scheme 1c). The more challenging double hydroboration of pyridines to tetrahydropyridines, however, has remained mostly unsuccessful. Double hydro-boration of quinolines was achieved both in the presence of catalytic amounts of B(C_6_F_5_)_3_,^25^ with Rh^26^ or Co^27^ catalysts. To the best of our knowledge, there is only a single report on the catalytic double hydroboration of pyridine (Scheme 1c).^28^ The Rh-phosphine catalyst gave 1,2,3,4-tetrahydropyridine diboronate in good yield and moderate regiocontrol. No substrate scope beyond the parent pyridine was explored.^28^

Following this lead and our recent explorations of selective hydrofunctionalizations with 3d transition metal catalysts,^29*a*–*e*^ we aimed at the development of a base metal-catalyzed double hydroboration of pyridines. In light of the recent advances in metal–ligand cooperativity (MLC) of base metal catalysts,^30^ we sought to exploit a dual activation approach that would enable metal-centered pyridine coordination, ligand-centered borane activation, and metal hydride formation without ligand dissociation. Such MLC scenario was utilized in the 1,2-selective hydroboration of pyridines with Fe and Ni catalysts.^15,17^ 2-Pyridonates constitute a suitable ligand family for such reactions: The bidentate 1,3 *N*,*O* motif features a rich coordination chemistry with most transition metals due to flexible binding modes, hemilability, and metal–ligand cooperativity in bond activations.^31–34^ We reasoned that such multifunctional behaviour of metal pyridonates may facilitate hydroborations of pyridines. Herein, we report the successful implementation of these mechanistic scenarios into the first base metal-catalyzed double hydroboration of pyridines to tetrahydropyridines. Complementing our current investigations into the coordination chemistry of 3d metal pyridonates,^34^ cobalt complexes featuring a 2-pyridonate motif modified by 6-dialkylphosphine substituents were prepared. The regio-selective Co-catalyzed borylative reduction of pyridines enabled the synthesis of highly versatile *N*-heterocyclic allyl boronates (Scheme 1d), which can be converted into bench-stable *N*-acetyl tetrahydropyridinyl boronates and various further derivatives.^2,35*a*–*g*^

## Results and discussion

### Catalyst synthesis

**Scheme 2:**
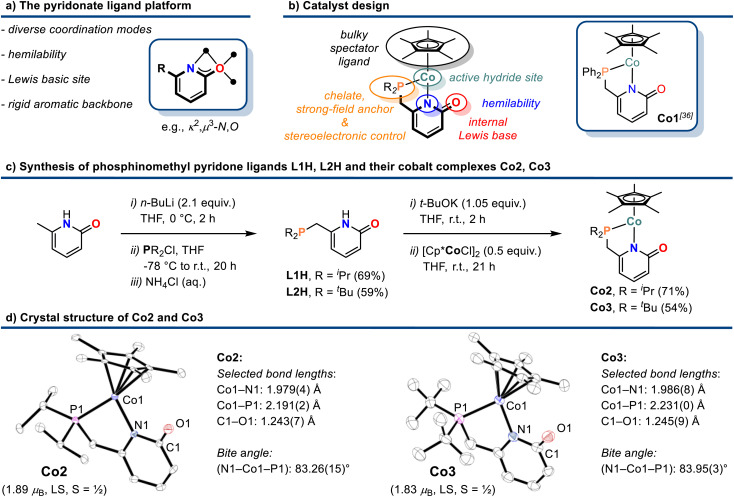
Design concept (a) and (b), synthesis (c), and crystal structures (d) of cobalt pyridonate complexes Co2 and Co3. Ellipsoids are shown at 50% probability.

Efficient 3d transition metal-catalyzed mono-hydroborations of pyridines have been accomplished with cyclopentadienyl metal complexes bearing hemilabile donor-functionalized phosphines.^15,17^ Upon employment of the modular ligand platform 6-phosphinomethyl-2-pyridone, we prepared heteroleptic cobalt(ii) complexes of the general formula (*η*^5^-C_5_Me_5_)Co(R_2_P∩N). We believed that a combination of the Lewis basic pyridonate with a strong-field phosphine and a bulky spectator ligand would prohibit unwanted catalyst aggregation and coordinative saturation with substrate molecules while enabling dual activation of both substrates of the targeted hydroboration reaction, the borane and the pyridine (Schemes 2a and 2b). Complementing the literature-known diphenylphosphino complex Co1,^36^ we varied the phosphine substituents to cover a wider structure–activity window. The di-*iso*-propyl (Co2) and di-*tert*-butyl (Co3) derivatives were prepared by a similar route involving sequential deprotonation of commercial 6-methyl-2(1*H*)-pyridone, phosphinylation, deprotonation, and substitution of the [Cp*CoCl]_2_ dimer with the pyridonate (Scheme 2c). Single crystal structure analyses confirmed the expected *κ*^2^-*P*,*N* chelating modes and pendent C=O moieties (Scheme 2d). The C–O bond distances (Co2: 1.243(7) Å; Co3: 1.245(9) Å) are consistent with a double bond character and align with the values of Co1 and related Ru and Ir phosphinopyridonates.^36–38^ The bite angles (N–Co–P) and the bond lengths (Co–P, Co–N) of the phosphinopyridonate ligands increase in the series Ph_2_P < ^*i*^Pr_2_P < ^*t*^Bu_2_P.

The magnetic moments (Evans: Co2 = 1.89 *μ*_B_; Co3 = 1.83 *μ*_B_) are in full agreement with low-spin Co(ii) ions (d^7^, *S* = 1/2) and 17-electron complexes.

### Comparison of cobalt pyridonate complexes

Investigations into catalytic hydroborations were performed on the model reaction between 4-methylpyridine (1a) and pinacolborane (HBpin) in the presence of the cobalt(ii) phosphinopyridonate complexes Co1–Co3 (Table 1). The reactions (1a, 2.2 equiv. HBpin, 3 mol% catalyst, C_6_D_6_) were monitored by ^1^H-NMR spectroscopy. The diphenylphosphino complex Co1 exhibited minimal activity in the mono-hydroboration (5% conversion) with no activity in the double hydroboration (Table 1, entry 1). Nearly full conversion was observed after 20 h at 50 °C with both Co2 and Co3, respectively. Employment of the less bulky di-*iso*-propylphosphino complex Co2 afforded minor amounts of the mono-hydroboration product, as the *N*-boryl 1,2-dihydropyridine isomer (2a, 12%), and predominantly the double hydroboration product as a single 1,3-di-borylated 1,2,3,6-tetrahydropyridine isomer (3a, 56% yield; Table 1, entry 2). The di-*tert*-butyl complex Co3 showed good selectivity toward the double hydroboration product 3a but with lower overall yield (32%, Table 1, entry 3) due to the formation of considerable amounts of unidentified byproducts. A similar trend in reactivity was observed with the model substrate 4-phenylpyridine (1b). Co2 gave the corresponding di-boryl tetrahydropyridine 3b in 42% yield, while Co3 fared poorer and afforded only 7% yield of 3b (Table 1, entries 4–5). Unlike for 4-methylpyridine 1a, the 4-phenylpyridine 1b reactions afforded the *N*-boryl tetrahydro-pyridine byproduct 3b′ without a C–Bpin motif (by NMR, GC-MS, and independent synthesis from 4-phenyl-1,2,3,6-tetrahydropyridine and HBpin, see Schemes S1–S3†). In no case was the 1,4-dihydropyridine derivative formed. With the identification of Co2 as most active and selective pre-catalyst in the double hydroboration of pyridines, further optimizations of the reaction conditions were performed (stoichiometry, concentration, solvent, temperature; see Tables 2 and S1†). Under mild conditions (50 °C), best yields of the double hydroboration product 3a were obtained with 2.2 equiv. HBpin and 3 mol% Co2 at 0.6 M substrate concentration in C_6_D_6_ (63% yield, Table 2, entry 6; = Method A). The yield of 3a could be increased to 86% in toluene-*d*_8_ solution at elevated temperature (100 °C, Table 2, entry 7; = Method B). Several experiments confirmed the crucial role of all catalyst components: The catalyst-free background reaction gave no conversion (entry 8); phosphinopyridone L1H and its conjugate base (L1H + KO^*t*^Bu) showed no activities (entries 9, 10). The complexes [Cp*CoCl]_2_ (Co4) and Cp*_2_Co (Co5) were also inactive in the double hydroboration of 1a (entries 11, 12).

**Table 1:** Catalytic activities of Co1–Co3 in the double hydroboration of pyridines^a^

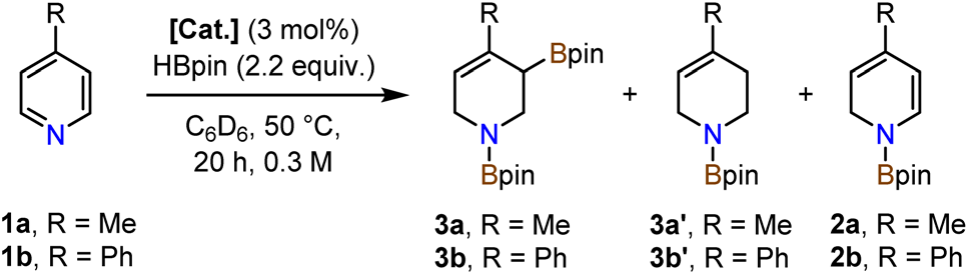					
Entry	Catalyst	Conversion [%]	Yield [%]		
			3a	3a′	2a
1	Co1	5	0	0	4
**2**	**Co2**	**95**	**56**	**0**	**12**
3	Co3	>99^b^	32	0	<1
			3b	3b′	2b
**4**	**Co2**	**>99**	**42**	**18**	**<1**
5	Co3	>99	7	7	64

a Conditions: Substrate (0.20 mmol), HBpin (2.2 equiv.), [Cat.] (3 mol%), 0.3 m in C_6_D_6_, 50 °C, 20 h, in a J. Young NMR tube without stirring unless otherwise specified. Yields and conversions from ^1^H-NMR *vs.* internal hexamethylbenzene (in C_6_D_6_).

b Formation of unidentified byproducts.

**Table 2:** Screening of reaction conditions in the double hydroboration of 4-methylpyridine 1a^a^

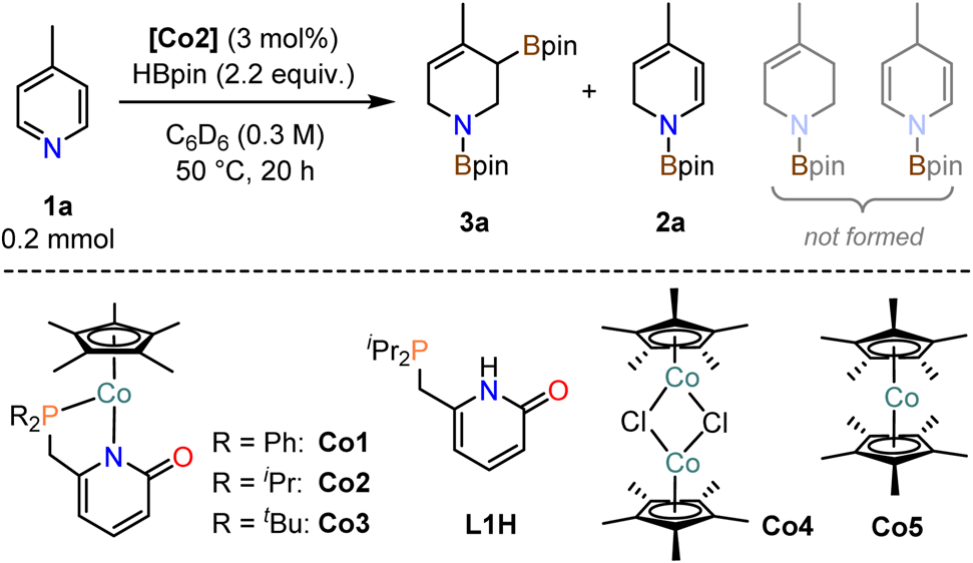			
Entry	Deviation from standard conditions	Yield [%]	
		3a	2a
1	None	56	12
2	Toluene-*d*_8_ as solvent	44	34
3	THF-*d*_8_ as solvent	16	60
4	3.3 equiv. HBpin	56	13
5	5 mol% Co2	48	19
**6^b^**	**0.6 M**	**63^c^**	**8**
**7**	**0.6 M, toluene-*d***_**8**_ **, 100 °C**	**86**	**4**
8^b^	No catalyst, toluene-*d*_8_, 100 °C	0	0
9^b^	3 mol% L1H, toluene-*d*_8_, 100 °C	0	0
10^b^	3 mol% (L1H + KO^*t*^Bu), toluene-*d*_8_, 100 °C	0	1
11^b^	3 mol% [Cp*CoCl]_2_ (Co4), toluene-*d*_8_, 100 °C	0	5
12	3 mol% Cp*_2_Co (Co5), toluene-*d*_8_, 100 °C	0	10

a Standard conditions: 1a (0.20 mmol), HBpin (2.2 equiv.), Co2 (3 mol%), 0.3 m in C_6_D_6_, 50 °C, 20 h, in a headspace vial (5 mL) with magnetic stirring unless otherwise specified. Yields from ^1^H-NMR *vs.* internal hexamethylbenzene (in C_6_D_6_) or 1,3,5-trimethoxybenzene (in toluene-*d*_8_).

b 0.40 mmol reaction.

c Isolated after *N*-acetylation in 54% yield.

### Substrate scope

**Scheme 3:**
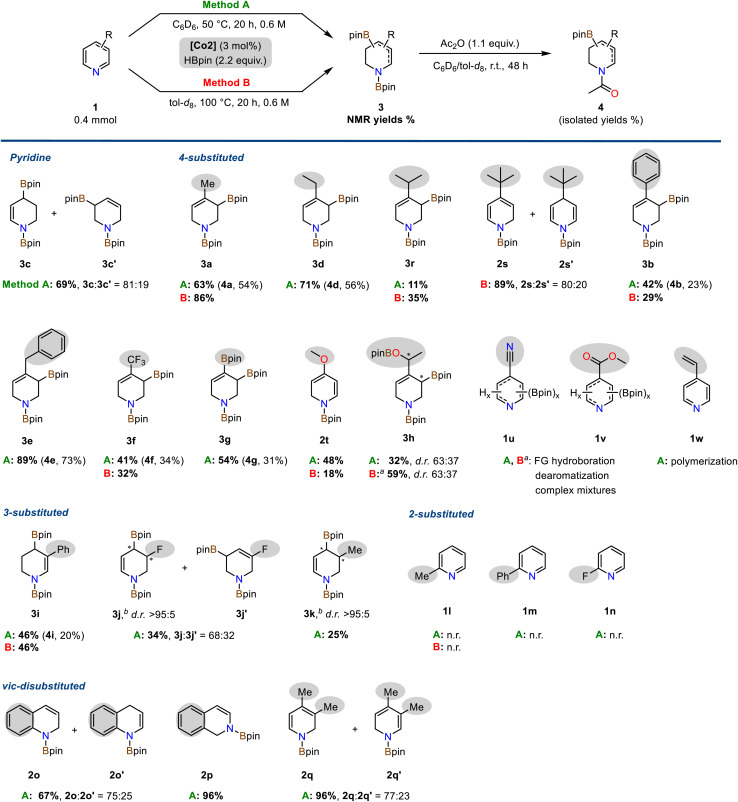
Substrate scope of the double hydroboration of pyridines. Conditions: Pyridine (0.40 mmol), HBpin (0.88 mmol, 2.2 equiv.), Co2 (3 mol%) in C_6_D_6_ at 50 °C (Method A) or in tol-d_8_ at 100 °C (Method B) at 0.6 m for 20 h inside a headspace vial (5 mL) with magnetic stirring unless otherwise specified. Yields of di-boryl products (shown in bold) were determined by ^1^H-NMR spectra of the crude mixtures *vs.* internal hexamethylbenzene (Method A) or 1,3,5-trimethoxybenzene (Method B). Isolated yields of the *N*-acetyl allyl boronates given in parentheses. ^*a*^ 4.4 equiv. HBpin. ^*b*^ Only a single diastereomer observed in ^1^H-NMR spectra. *n. r.* = no reaction.

The optimized conditions were applied to a diverse set of pyridine derivatives in order to explore the scope of the double hydroboration reaction (Scheme 3). The parent pyridine underwent double hydroboration in 69% yield furnishing a mixture of two regioisomers (*r.r.* 4/1). Remarkably, 4-substituted pyridines cleanly afforded a single isomer in the double hydroboration, *i.e.* the 1,3-di-boryl-1,2,3,6-tetrahydropyridines, in up to 89% yield. Treatment of the crude mixtures with acetic anhydride allowed one-pot conversions to the bench-stable *N*-acetyl allyl boronate derivatives. Alkyl, aryl, benzyl, trifluoromethyl, and boronate functions were well tolerated. The bulky 4-*tert*-butylpyridine and the electron-rich 4-methoxypyridine enabled only single hydroborations (2s, 2s′ and 2t), respectively. Acetylation of the triboronate 3g resulted in the formation of the highly versatile *N*-heterocyclic building block 4g containing both a vinyl and allyl boronate motif. 4-Acetylpyridine 1h underwent double hydroboration of the pyridine ring and mono-hydroboration of the keto substituent to give two diastereomeric tetrahydropyridines 3h with chiral C–B and C–OB motifs (with 4.4 equiv. HBpin). Pyridines bearing carboxylate substituents (1u: 4-CN, 1v: CO_2_Me) gave complex mixtures of multiply reduced products. 4-Vinylpyridine 1w engaged in rapid polymerization under the reaction conditions. Interestingly, partially reduced tetrahydropyridine products carrying no C(sp^3^)–B bond (like 3b′) were only observed for selected substrates including 4-Ph (3b : 3b′ = 70 : 30), 4-CF_3_ (3f : 3f′ = 83 : 17) and 4-pinB substituted pyridines (3g : 3g′ = 81 : 19). 3-Substituted pyridines could also be subjected to double hydroborations which were strongly governed by substituent effects. 3-Methylpyridine 1k and 3-phenylpyridine 1i were converted to predominantly one major isomer, respectively, the 1,4-di-boryl-1,2,3,4-tetrahydropyridine derivatives 3i (46%) and 3k (25% yield). Borylative reduction of 3-fluoropyridine 1j resulted in a mixture of two regioisomers with *N*-heterocyclic allyl boronate motifs (*r.r.* 2/1); the major regioisomer 3j was produced as a single diastereomer (*d.r.* >95 : 5). Minor amounts of defluorination were observed (to 3c, 12% yield). Isolation of 3-substituted *N*-acetyl tetrahydro-pyridines was generally plagued by the complexity of the crude product. The formation of minor species impeded with isolation. The cobalt pyridonate catalyst strongly discriminated between differing substitution patterns: While 3- and 4-substituted pyridines were subjected to double hydroboration with a high preference for the allyl boronate motif, 2-substituted pyridines (1l, 1m, 1n) exhibited no reactivity, neither in mono-hydroboration nor in double hydroboration. Quinoline 1o, isoquinoline 1p, and 3,4-lutidine 1q, on the other hand, underwent single hydroboration in 67–96% yield with no second hydroboration event occurring. Further substitution patterns that prohibited double hydroboration under the standard conditions were bulky *tert*-butyl groups, methoxy, *sec*-amine, halides, ester, nitrile, and vinyl groups (Scheme S4†).

### Thermodynamic and kinetic properties

**Scheme 4:**
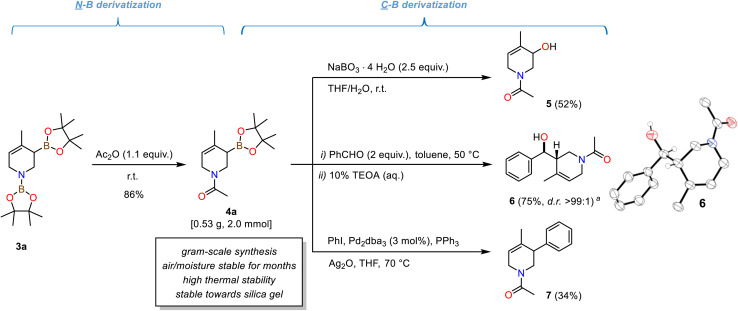
Examples of chemical transformations of tetrahydropyridine diboronates: N-Acetylation to bench-stable derivative 4a; oxidation to allyl alcohol 5; stereoselective allylboration of benzaldehyde to 1,3-aminoalcohol 6; Suzuki-type arylation to bicyclic 7.

The isolated *N*-acetyl tetrahydropyridine derivatives exist as two rotamers about the C–N amide bond in solution.^39^^1^H and ^13^C-NMR spectra displayed two distinct set of signals; from temperature-gradient ^1^H and ^13^C-NMR analysis in toluene-*d*_8_ between 27 °C and 100 °C the rotation barrier was determined to be ∼73.6 kJ mol^−1^ (from a modified Eyring analysis, see Schemes 4, S5 and S6†).^40^ The compounds exhibited high thermal stability: Complete conservation of compound integrity and spectral data was observed when heating 4a to 100 °C under air for 6 h (Scheme S7†). The *N*-heterocyclic allyl boronates were also stable toward silica gel and moisture, so that purification by flash chromatography did not require special handling procedures. This is surprising as cyclic allylic boronates were occasionally described as being unstable and difficult to isolate.^3,41,42^ Nevertheless, very minor amounts of unidentified pinacolato-boron species remained after purifications. Long-term storage of the *N*-acetyl tetrahydropyridines for two to four months under an atmosphere of air (both in solution and as an oily liquid) at −30 °C led to a slight increase in pinB-containing impurities with otherwise complete product integrity (NMR spectra, Schemes S8 and S9†). The synthetic utility of the isolated *N*-acetyl tetrahydropyridines is evident from several literature reports on (allyl)boronate and tetrahydropyridine derivatizations.^2,35*a*–*g*^ The methallyl boronate 4a was isolated as a colorless oil from a gram-scale reaction. Oxidation of 4a with sodium perborate cleanly afforded the *N*-heterocyclic allylic alcohol 5. Stereoselective allylboration of benzaldehyde proceeded with excellent diastereoselectivity (*d.r.* >99 : 1). NMR spectral data and single crystal X-ray diffraction confirmed the formation of the *syn*-diastereomer (Scheme 4, right).^3,41^ Palladium-catalysed Suzuki-type arylative cross-coupling enabled the synthesis of the tetrahydropyridine 7.

### Mechanistic studies

Various spectroscopic and preparative experiments were performed to gain insight into the nature of the catalytically active cobalt hydride species and the formation of pyridine-derived products.

### Cobalt hydride complexes

**Scheme 5:**
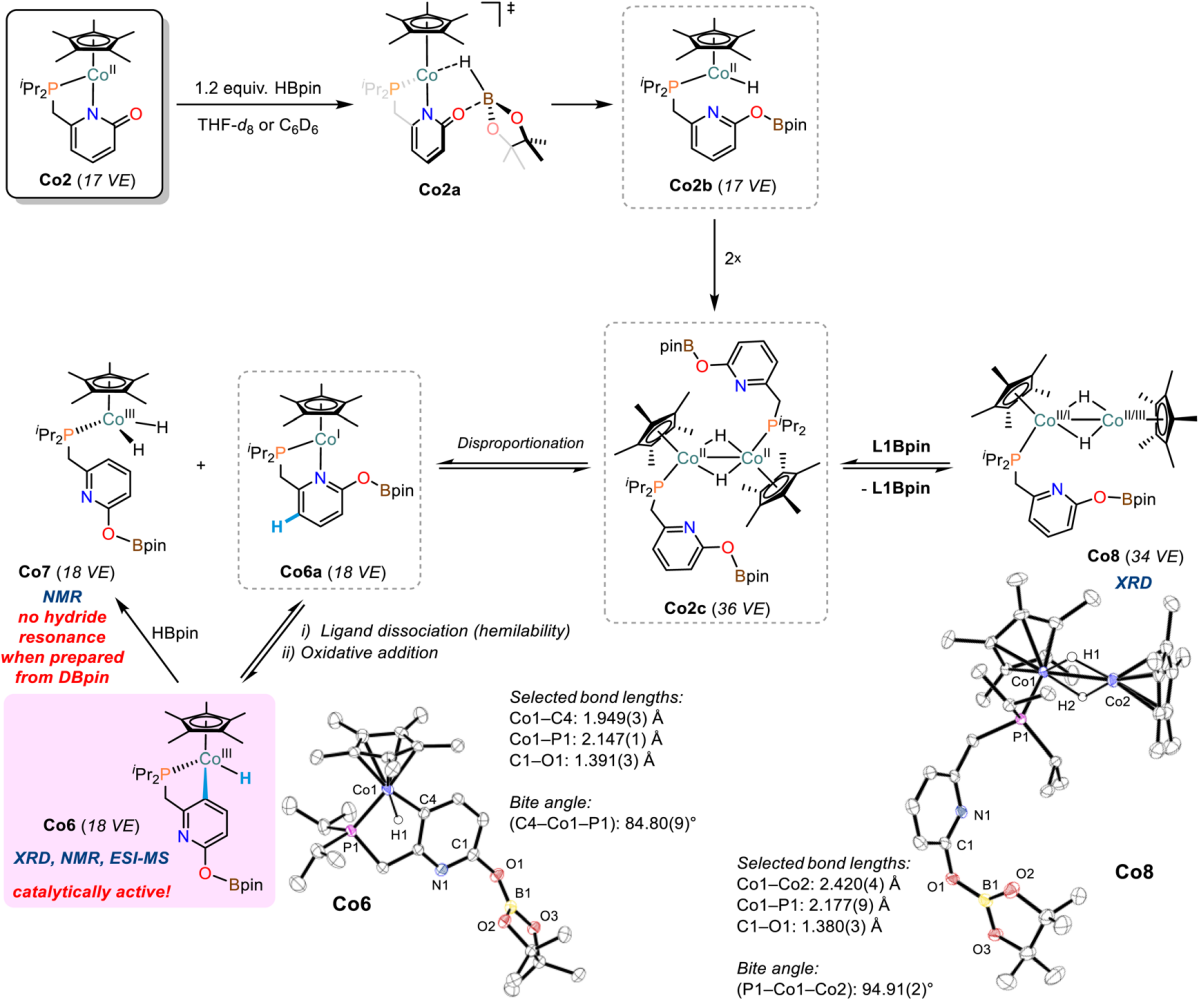
The formation of cobalt hydride complexes from reactions of Co2 with HBpin. Ellipsoids are shown at 50% probability.

Addition of pinacolborane (1.2 equiv.) to a solution of cobalt pyridonate complex Co2 in THF-*d*_8_ or C_6_D_6_ resulted in immediate color change from red to black. Careful ^1^H and ^11^B-NMR monitoring indicated formations of diamagnetic and paramagnetic species (Schemes S10–S20†): (i) very minor amounts of the borylated ligand L1Bpin were observed by ^1^H-NMR. (ii) we postulate the formation of dinuclear complexes of the formula [(Cp*Co)_2_H_*n*_(L1)_*m*_] as major paramagnetic species with Co(i), Co(ii), or mixed valence states, which is supported by ESI-MS analyses displaying peaks of *m/z* = 612.1975 (*n* = 0, *m* = 1) and 837.3194 (*n* = 1, *m* = 2; see Schemes S21–24†). Similar hydride- and pyridonate-bridged dinuclear complexes were prepared from [Cp*CoCl]_2_ by addition of LiAlH_4_ (ref. 43) and from [Cp*IrCl(2-pyridonate)]^44^ by H-atom transfer, respectively. (iii) Two distinct doublets of hydride complexes were observed at −16.51 ppm (^2^*J*_(P,H)_ = 87.3 Hz, C_6_D_6_) and −17.96 ppm (^2^*J*_(P,H)_ = 84.4 Hz, C_6_D_6_) and assigned to Co(iii) hydride species, in full agreement with the spectral signature of closely related Cp(*)Co^III^H(PR_3_) complexes.^45,46^ The more downfield resonance originates from the diamagnetic pyridyl monohydride complex Co6, which was isolated as a pure crystalline compound and characterized in solid state (XRD, elemental analysis) and solution (NMR, ESI-MS). Co6 features a cyclometalated κ-C-pyridonate ligand that underwent ligand-C-H activation (Scheme 5). The slightly upfield hydride signal presumably is derived from a dihydrido cobalt complex and was tentatively assigned to complex Co7 (Scheme 5, see also Scheme S20†).

An identical reaction between Co2 and DBpin cleanly documented that the hydride resonance of Co6 is unaffected from the use of the isotopomeric borane reagent, so that this cobalt hydride is ligand-derived. The concomitantly formed Co7 showed strongly reduced intensity of the hydride resonance which supports the notion that the hydrido ligands are borane-derived (Scheme S25†). This is further corroborated by the observed formation of Co7 (and minor amounts of other cobalt hydrides) from the reaction of pure Co6 with pinacolborane (Scheme S26†).

Attempts to isolate pure Co7 were unsuccessful, but upon crystallization, a [Cp*Co]-capped derivative thereof was obtained, the dihydrido-bridged Co8 (Scheme 5). The Co–Co bond distance in Co8 of 2.420(4) Å suggests significant metal–metal bonding.^43,47,48^ The residual electron density located between the cobalt centers of Co8 is consistent with bridging hydride ligands.^43^ The differences of model and diffraction data resulted in an uneven electron distribution among the Co ions, with higher electron density on the phosphino-bound cobalt ion (Co1) and lower electron density on the other center (Co2). This may be a strong argument for a Cp*(R_3_P)Co(i) fragment and a Cp*Co(iii) cap, which could have formed *via* an intermediate cobalt(ii) hydride species upon dinuclear disproportionation.^48^ Solutions of Co8 produced ^1^H-NMR spectra displaying the resonances of Co6, Co7, L1Bpin, as well as unidentified paramagnetic species. We assume dynamic interconversions between several cobalt hydride species in solution (*e.g.*Co8 → Co6/Co7) and/or co-crystallization of different cobalt hydride complexes. Based on the isolated complexes and the collected spectroscopic data, we propose the following mechanism of cobalt hydride catalyst formation (Scheme 5): The pyridonate ligand in Co2 is believed to support borane activation *via* metal–ligand cooperativity (Co2a) yielding transient cobalt(ii) hydride Co2b which dimerizes with another equivalent of Co2b to give Co2c. This bimetallic complex may engage in disproportionation to give the proposed dihydride species Co7 (^1^H-NMR) and the short-lived Co6a. The hemilabile nature of the pyridonate ligand becomes evident by oxidative addition of the cobalt(i) centre into the ligand backbone to give Co6 (XRD, NMR, ESI-MS). However, the dimeric cobalt complex Co2c may also lose a phosphine ligand which results in the formation of Co8 (XRD).

### Hydroboration products analysis

**Scheme 6:**
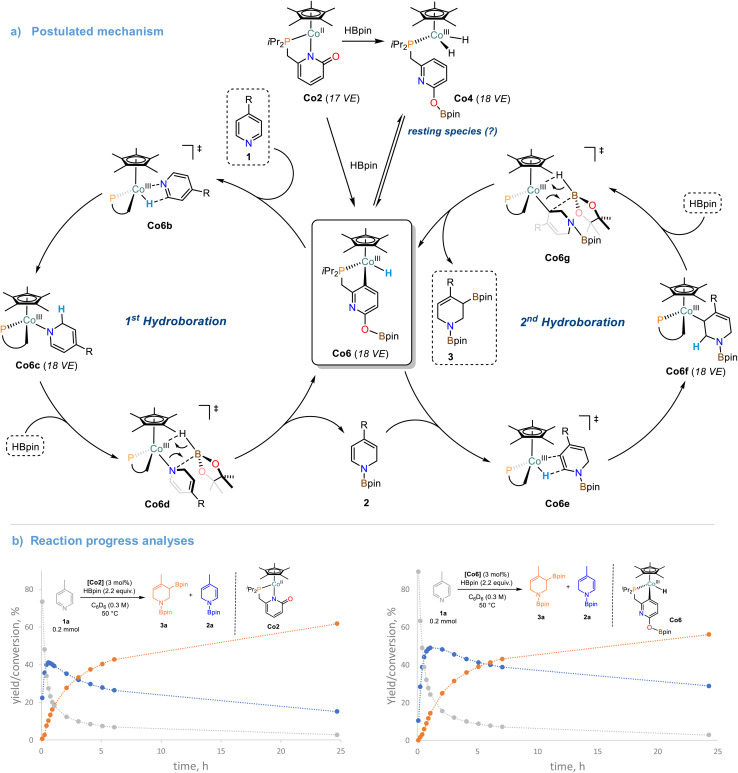
(a) Proposed mechanism of the cobalt-pyridonate catalyzed double hydroboration of pyridines. (b) Reaction progress analyses of the double hydroboration of 4-methylpyridine with catalytic Co2 and Co6, respectively.

The cobalt monohydride complex Co6 was found to be equally active to Co2 in the catalytic double hydroboration of 4-phenylpyridine 1b, which again suggests rapid interconversion from Co2 to Co6. Full conversion of 4-phenylpyridine 1b to the tetrahydropyridine derivatives 3b and 3b′ was observed when employing Co6 as catalyst (3 mol%). The stoichiometric reaction between Co6 and 1a resulted in rapid consumption of Co6 at room temperature (complete consumption at 50 °C, Scheme S27†). This equimolar reaction did not lead to the formation of the mono-reduction product 2a. Addition of one equiv. HBpin to the Co6/pyridine reaction mixture afforded the mono-hydroboration product 2a and regeneration of the cobalt hydride complex Co6 as well as formation of Co7 (Schemes S27 and S28†). Formation of the tetra-hydropyridine product 3a was only observed after addition of a 2nd equiv. HBpin and heating to 50 °C (Scheme S28†). When Co2 was reacted with one equiv. 4-Benzylpyridine 1e and one or two equiv. HBpin at room temperature, only traces of Co6 were observed while the hydride signal of Co7 was rather unaffected (Schemes S30–S32†). The formation of the mono/double hydro-boration products was associated with the same stoichiometry of HBpin addition. Reaction of the isotopomeric DBpin with 1b in the presence of 3 mol% Co2 led to deuteration in 2- and 6-positions of the tetrahydropyridine product (Schemes S33 and S34†). Based on the collected analytical data and previously reported DFT studies,^27,28^ we propose a mechanism for the Co-catalyzed double hydroboration of pyridines (Scheme 6a): The direct reaction of Co6 with pyridine substrates suggest rapid insertion into the Co–H bond to give the 2-hydropyridinyl complex Co6c. The borate function at the ligand is most likely only involved in the initiation steps but rather not operative in *O*-to-*N* boryl transfer onto the substrates. Thus, liberation of the mono-hydroboration product 2 is believed to occur *via σ*-bond metathesis between Co6d and HBpin which also regenerates Co6. The 2nd hydroboration event commences with a regioselective insertion step of the Co–H bond of Co6 into the polarized C=C bond of the enamine motif within 2. The resultant Co6f may undergo another *σ*-bond metathesis with HBpin to give the desired tetrahydropyridine derivative 3. The high regiocontrol of the second hydroboration is dictated by the polarity of the enamine-C=C bond, the lower sterics, and the formation of the thermodynamically more stable allyl boronate species (*vs.* alkyl boronate). The close mechanistic relationship between the precursor Co2 and the postulated catalyst species Co6 becomes also evident from kinetic reaction progress analyses (Scheme 6b). Under identical conditions, very similar reaction profiles were observed that include identical reaction slopes of the 1st hydroboration event, very similar yields and lifetimes of the intermediate 1,2-dihydropyridine derivative, a significantly slower 2nd hydroboration step, and comparable yields and selectivities of the final product mixture. It is important to note, that the operation of a “hidden” BH_3_ catalysis was largely excluded: ^11^B NMR studies of various mixtures of HBpin with the Co2 and the pyridine under reaction conditions did not exhibit the characteristic upfield resonance of BH_3_·L, even in the presence of added TMEDA (*N*,*N*,*N*′,*N*′-tetramethylethylenediamine, see Scheme S38†).^49^ A similar stepwise hydroboration mechanism may be operative during the hydroboration of other polarized π-systems such as the cobalt pyridonate catalyzed hydroboration of CO_2_ currently studied in our laboratory.^50^

## Conclusions

The demand for highly functionalized and synthetically versatile platform molecules for the synthesis of biologically active compounds has prompted great interest in selective catalytic transformations. Six-membered *N*-heterocycles are an especially attractive class of building blocks for medicinal chemistry programs, yet, straight-forward synthetic routes that utilize commercial starting materials are rare. Based on a modular ligand platform and easily available substrates, an efficient cobalt-catalyzed double hydroboration of pyridines to tetrahydropyridines was developed. The catalyst was easily prepared from commercially available 6-methyl-2(1*H*)-pyridone by sequential deprotonation, phosphinylation, deprotonation, and salt metathesis with [Cp*CoCl]_2_ Co4. The double hydro-boration reaction afforded versatile *N*-heterocyclic allyl boronates that can be converted to bench-stable borylated *N*-acetyl tetrahydropyridines in a one-pot fashion. The double hydroboration of 4-substituted pyridines was found to occur in a regioselective manner with the formation of a single isomer. Remarkably, 1,2,3,6-tetrahydropyridines were the only reduced species formed with mostly full conversions of the intermediate 1,2-dihydropyridines and no onward hydroboration to the fully saturated piperidines. 3-Substituted pyridines gave somewhat lower regiocontrol. Mechanistic studies have revealed critical steps of catalyst and substrate activations and deep insight into the coordination chemistry at cobalt.^26–28^ Structural variation of the modular 2-pyridonate ligand enabled high activities of the iso-propyl derivative. The presumably formed cobalt(ii) hydride complex Co2b underwent disproportionation to give cobalt(iii) hydride complex Co6 that was found to be catalytically active. Hydride insertion of Co6 into the pyridinyl C=N and C=C bonds and *σ*-bond metathesis with pinacolborane are believed to be key steps of the double hydroboration mechanism. The easy access to the pre-catalyst Co2 and the identification of Co6 as active catalyst species may prompt further studies into the exploration of base metal catalysts for polar hydro-functionalization reactions of π-systems.^50^

## Data availability

All data including experimental and analytical details are in the ESI.† More data incl metadata will be stored at the authors institutional database center.

## Author contributions

F. H., L. L. and A. F. conducted the experiments and analytical studies. F. H., A. F. and A. J. v. W. wrote the manuscript.

## Conflicts of interest

There are no conflicts to declare.

## Supplementary Material

SC-015-D3SC05418G-s001

SC-015-D3SC05418G-s002
