# Supplementary material for: Cobalt-catalyzed double hydroboration of pyridines
Source: Chem Sci. 2024 Mar 8;15(14):5201–10. doi: 10.1039/d3sc05418g (PMC10988591; doi:10.1039/d3sc05418g)
Supplement: SC-015-D3SC05418G-s001 [file SC-015-D3SC05418G-s001.pdf]

Electronic Supplementary Material (ESI) for Chemical Science.
This journal is © The Royal Society of Chemistry 2024

Supporting Information for

Cobalt-Catalyzed Double Hydroboration of Pyridines

Finn Höeg,[a,b] Lea Luxenberger,[a] Andrey Fedulin,[a,c] and Axel Jacobi von Wangelin*[a]*

[a] *Dept. of Chemistry, University of Hamburg, Martin Luther King Pl 6, 20146 Hamburg, Germany*

[b] *now at: Philipps-University of Marburg, Hans-Meerwein-Str 4, 35043 Marburg, Germany*

[c] *now at: University of Regensburg, Universitaetsstr 31, 93040 Regensburg, Germany*

Table of contents

1. General Information 1

2. Synthesis and Characterisation of Pyridone Ligands and their Complexes 5

3. Double Hydroboration of Pyridines 13

3.1 Comparison of Cobalt Catalysts and General Procedure for NMR Scale Double Hydroboration Reactions 13

3.2 Identification of reaction products 14

3.3 General Procedure for Reaction Optimisation 18

3.4 General Procedure for the Preparative Double Hydroboration–*N*-Acetylation sequence 20

4. Characterisation Data of Double Hydroboration Products 21

4.1 Characterisation Data of bis(borylated) Tetrahydropyridines 21

4.2 Characterisation of Borylated *N*-Acetyl Tetrahydropyridines 31

5. Unsuccessful substrates 42

6. Rotamer Formation and Long-Term Stability of *N*-Acetyl Allylic Boronates 44

6.1 Temperature Gradient NMR spectroscopy 44

6.2 Long-Term Stability of *N*-Acetyl Allylic Boronates 46

7. Mechanistic Studies 48

8. Gram-Scale Sequential Double Hydroboration–*N*-Acetylation of 4-Methyl-pyridine 76

9. Chemical Diversification of Borylated *N*-Acetyl Tetrahydropyridines 77

10. Other Procedures and Characterisation Data 82

11. Experimental Spectra 87

11.1 Experimental Spectra of Pyridone Ligands and their Complexes 87

11.2 Experimental Spectra of bis(borylated) Tetrahydropyridines 102

11.3 Experimental Spectra of Borylated *N*-Acetyl Tetrahydropyridines 117

11.4 Experimental Spectra of Derivatised Tetrahydropyridines 132

11.5 Other Experimental Spectra 139

12. X-ray Crystallography Data 154

13. References 159

1. General Information

Reactions, solvents and chemicals. Unless stated otherwise, all reactions were performed under argon atmosphere using standard Schlenk or glovebox techniques. Solvents (*n*-hexane, toluene, acetonitrile) were purified by a SPS solvent purification system under nitrogen atmosphere. THF was distilled over sodium using benzophenone as an indicator. Deuterated solvents (C_6D_6, toluene-d_8, THF-d_8) were degassed by freeze-pump-thaw technique and stored over molecular sieves (4 Å) at least 24 h prior to use. All anhydrous solvents were stored over molecular sieves (4 Å) under inert atmosphere. Other solvents used for reactions under atmospheric conditions, column chromatography and extraction under air (EtOAc, pentane, CH_2Cl_2, MeOH, THF) were of technical grade and distilled prior to use. Substrates and other chemicals were sourced from commercial vendors and if not stated otherwise, used as received. Ligand 6-((diphenylphosphanyl)methyl)pyridin-2(1*H*)-one[1], cobalt complex **Co1**[2] and $Co[N(SiMe_3)_2]_2$[3] were prepared according to previously reported procedures. 6-Methylpyridin-2(1*H*)-one was purified by sublimation prior to use. Pinacolborane (HBpin) was degassed by freeze-pump-thaw technique prior to use. All liquid pyridine substrates were distilled under reduced pressure, degassed and stored over molecular sieves (4 Å) at least 24 h prior to use. Solid pyridine substrates were dried in vacuum prior to use.

NMR spectroscopy. Nuclear magnetic resonance spectra (^{1}H-, ^{13}C-, ^{11}B-, ^{19}F-, ^{31}P-, 2D-NMR spectra) were recorded at the Institute of Organic Chemistry of the University of Hamburg on different *Bruker spectrometers* (*Bruker Avance III HD 600 MHz (AVIII600), Bruker Avance I 500 MHz (AV500), Bruker Avance I 400 MHz (AV4001), Bruker FourierHD 300 MHz (F300UHH)*) and analyzed by using the MestReNova software (version 14.0.0-23239, © 2019 by Mestrelab Research S.L.). Chemical shifts (δ) are reported in parts per million (ppm) relative to the reference substance (tetramethylsilane for ^{1}H and ^{13}C, $Et_2O{\cdot}BF_3$ for ^{11}B, $CFCl_3$ for ^{19}F, 85% H_3PO_4 for ^{31}P) and were calibrated using the signal of the corresponding NMR solvent (for ^{1}H and ^{13}C). The ^{11}B-, ^{19}F-, and ^{31}P-shifts are uncorrected. Coupling constants *J* are given in Hertz (Hz). The following abbreviations are used to indicate multiplicities in the ^{1}H-NMR spectra: br = broad, s = singlet; d = doublet; t = triplet; q = quartet; pent = quintet; hept = heptet; m = multiplet; and combinations thereof. The assignment of the ^{1}H- and ^{13}C-NMR signals was supported by the following 2D-NMR spectroscopy techniques:

- Homonuclear COSY (^{1}H-, ^{1}H-correlation spectroscopy)
- HSQC (heteronuclear single quantum correlation spectroscopy)
- HMBC (heteronuclear multiple bond correlation spectroscopy)
- NOESY (nuclear Overhauser effect spectroscopy)

The numbering of the compounds does not match the IUPAC nomenclature. A slash "/" refers to "or" and is used if the signal cannot be assigned with certainty. A "a/b" is used to distinguish between diastereotopic protons or chemically inequivalent groups.

Evans-NMR. The magnetic moment of paramagnetic complexes was determined in solution by performing an NMR experiment following the procedure of Evans.[4] Benzene in C_6D_6 was used as a reference compound.

High-resolution mass spectrometry (HRMS). High resolution ESI (electrospray ionisation) mass spectra (HRMS-ESI) for organic products were recorded at the Institute of Organic Chemistry of the University of Hamburg on an *Agilent 6224 ESI-TOF* mass spectrometer (*Agilent Technologies*) between 110–3200 *m/z* in the positive ion mode by direct injection. The analysis of the spectra was performed by using the MestReNova software (version 14.0.0-23239, © 2019 by Mestrelab Research S.L.). Abbreviations used for MS data: *m/z* = mass-to-charge ratio; M = exact mass of the target compound.

ESI-MS analysis for air sensitive **Co2**. A crystalline sample of **Co2** was dissolved in THF and directly subjected to ESI-MS analysis. The sample solution was transferred into a gas-tight syringe and fed into the ESI source of a micrOTOF-Q II mass spectrometer (*Bruker Daltonik*) at a flow rate of 0.5 $mL \cdot h^{-1}$. The ESI source was operated at a voltage of 4500 V with N_2 as nebulizer (8.0 psi backing pressure) and drying gas (heated to 333 K and held at 3.0 $L \cdot min^{-1}$ flow rate). The hereby produced ions with $50 \leq m/z \leq 3000$ were then allowed to pass the instrument's quadrupole mass filter and collision cell before entering the time-of-flight (TOF) mass analyser. Ions were identified on the basis of their *m/z* ratio, their isotope pattern, and fragmentation patterns.

Gas chromatography–electron impact–mass spectrometry (GC-EI-MS). GC-MS samples were measured on an *Agilent 7820A* GC System equipped with an *Agilent 5977B MSD* mass detector (*Agilent Technologies*). The column is a *J&W HP-5ms* column ((5%-Phenyl)-methylpolysiloxane, 30 m length, 0.25 mm inner diameter, 0.25 µm film thickness) and samples were heated by the following temperature program: T_{start} = 50 °C → 2 min isotherm at 50 °C → 25 K/min heating rate to 300 °C (if needed → 5 min isotherm at 300 °C).

UV-Vis spectroscopy. UV-Vis absorption spectra were recorded at room temperature on an *Agilent Cary 5000 UV-Vis-NIR Spectrophotometer* (*Agilent Technologies*) in double-beam mode using 10 mm quartz cuvettes with a Teflon™ valve. The samples were prepared inside a glove box with <0.1 ppm O_2 and H_2O by dissolving the compound in THF and transferring the adequately diluted solution into the cuvette before closing the valve. The absorption

maxima (λ_{max}) are characterised by their wavelength (nm) and extinction coefficients (ε, $L \cdot mol^{-1} \cdot cm^{-1}$).

Elemental analysis. Elemental analyses were determined on an *EA3000* (*EuroVektor*) under inert atmosphere by the analytical department of the University of Hamburg. The samples were prepared inside a glove box with <0.1 ppm O_2 and H_2O by weighing 1.0–1.6 mg of the homogenised sample in tin capsules. The tin capsules were closed and stored under argon atmosphere until the measurement. The stated values for the C,H,N contents are the mean values of three independent measurements.

Thin-layer chromatography (TLC). TLC was conducted in a chamber saturated with mobile phase at ambient temperature using fluorescence indicator coated silica gel on aluminium sheets (*Alugram®* Xtra SIL G/UV_{254} 0.2 mm, *Macherey Nagel*). The spots were visualised with UV-light (254 nm), alkaline potassium permanganate stain, or vanillin/H_2SO_4 stain with additional heating.

Flash column chromatography. Preparative column chromatography was performed using silica gel (35–70 µm, 60 Å pore size, *Acros Organics*) as the stationary phase and pressure was applied with compressed air. The R_f-values were determined via TLC.

Fourier-transform infrared spectroscopy (FT-IR). FT-IR spectra were recorded neat on an *Agilent Cary 630 FTIR* with an ATR-device at room temperature inside a nitrogen filled glovebox. Absorption bands are characterised by their wavenumbers ($\tilde{\nu}$) and intensity. Abbreviations used for the intensity are as follows: vw = very weak; w = weak; m = medium; s = strong; vs = very strong.

Melting points. Melting points were measured with a *DigiMelt MPA160* (*Stanford Research Systems*) using a glass capillary sealed under reduced pressure. The values are uncorrected.

Single crystal X-ray crystallography. Single crystal X-ray experiments were performed by the X-ray service facility at the Institute of Inorganic and Applied Chemistry of the University of Hamburg at 100 K using a SuperNova four-circle diffractometer in Kappa geometry with a 50 W Cu or Mo (K_α radiation) microfocus tube, an Atlas CCD detector (*Rigaku Oxford Diffraction*), and a Cryostream 700 Plus cooler (*Oxford Cryosystems Ltd.*). Data collection, cell refinement, data reduction, and absorption correction were done using CrysAlis^{Pro}[5]. Intensities were measured using omega scans. Single crystal X-ray data was solved and refined by the X-ray service facility as follows: The space group was determined either by using XPREP (*Bruker AXS Inc.*[6]) or CrysAlis^{Pro} and the phase problem was solved either (a) by structure-invariant direct methods with SHELXS[7], or (b) by using the dual-space algorithm implemented in SHELXT[8]. In every case, full-matrix least-squares refinement was done on F^2 using

SHELXL[8]. Missing secondary atom sites were located from the difference Fourier map. If possible, non-hydrogen atoms were refined using individual, anisotropic displacement parameters. The fully refined data was reviewed using PLATON[9]. Carbon atom bound hydrogen atoms were positioned geometrically and refined riding on their respective parent atoms. U_{iso}(H) was fixed at 1.5 (CH_3) or 1.2 (all other H atoms) of the parent atom's isotropic displacement parameter.

2. Synthesis and Characterisation of Pyridone Ligands and their Complexes

6-((Diisopropylphosphanyl)methyl)pyridin-2(1*H*)-one L1H[10]

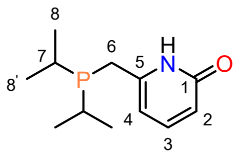

L1H

$C_{12}H_{20}NOP$

225.27 g/mol

To a suspension of 6-methylpyridin-2(1*H*)-one[1] (1.50 g, 13.8 mmol, 1.00 eq.) in THF (15 mL) was added *n*-butyllithium (1.6 M in hexane, 18 mL, 29 mmol, 2.1 eq.) in a dropwise condition over a period of 10 min while stirring at 0 °C. During the addition, the reaction mixture turned orange and the colourless solid dissolved continuously. Subsequently, the orange solution was stirred at 0 °C for 2 h. After completion, the reaction mixture was cooled to –78 °C and added dropwise (over 20 min) to a solution of chlorodiisopropylphosphine (2.20 mL, 13.8 mmol, 1.00 eq.) in THF (10 mL) at –78 °C with vigorous stirring. After the orange reaction mixture was stirred at –78 °C for 1 h, it was allowed to warm up to room temperature and stirred for an additional 19 h. The solvent was removed *in vacuo* and the syrupy, red residue was quenched with degassed[2] aqueous NH_4Cl solution (3.3 M, 30 mL). The suspension was stirred for 10 min, during which time the viscous slightly yellow solid gradually dissolved. Then it was extracted using CH_2Cl_2 (4x15 mL). The combined organic layers were dried over anhydrous $MgSO_4$, filtered and the solvent was evaporated *in vacuo*. Subsequently, the yellow crude product was crystallised from hexane (55 mL) at –30 °C. Lastly, the crystals were collected by filtration and washed with –90 °C cold hexane (2x3 mL). The target compound **L1H** (1.69 g, 7.49 mmol, 54%) was obtained in form of slightly yellow crystals. Fractionated crystallisation of the filtrate gave another 0.447 g of phosphine **L1H**[3] raising the total yield to 2.13 g (9.47 mmol, 69%).

m.p. = 84–85 °C.

^{1}H-NMR (500.1 MHz, C_6D_6, 24.9 °C): δ [ppm] = 14.34 (s, 1 H, -NH), 6.85 (dd, $^{3}J_{(H,H)}$ = 9.1 Hz, $^{3}J_{(H,H)}$ = 7.0 Hz, 1 H, 3-H), 6.42 (d, $^{3}J_{(H,H)}$ = 9.1 Hz, 1 H, 2-H), 6.09–6.06 (m, 1 H, 4-H), 2.69 (s, 2 H, 6-H), 1.61 (heptd, $^{3}J_{(H,H)}$ = 7.1 Hz, $^{2}J_{(P,H)}$ = 1.1 Hz, 2 H, 7-H), 1.01–0.93 (m, 12 H, 8-H and 8'-H)[4].

---

[1] 6-Methylpyridine-2(1*H*)-one was purified by sublimation ($1x10^{-3}$ mbar, 100 °C) prior to use.

[2] A nitrogen stream was bubbled through the aqueous solution for 1 h.

[3] The compound is sensitive towards air.

^{13}C-DEPTQ-NMR (125.8 MHz, C_6D_6, 24.7 °C): δ [ppm] = 166.4 (C-1), 149.9 (d, $^{2}J_{(P,C)}$ = 11.3 Hz, C-5), 141.5 (C-3), 116.4 (d, $^{5}J_{(P,C)}$ = 1.7 Hz, C-2), 106.1 (d, $^{3}J_{(P,C)}$ = 11.1 Hz, C-4), 26.9 (d, $^{1}J_{(P,C)}$ = 24.7 Hz, C-6), 23.9 (d, $^{1}J_{(P,C)}$ = 15.8 Hz, 2 C, C-7), 19.7 (d, $^{2}J_{(P,C)}$ = 15.0 Hz, 2 C, C-8/8'), 19.1 (d, $^{2}J_{(P,C)}$ = 11.3 Hz, 2 C, C-8/8').

^{31}P{^{1}H}-NMR (202.5 MHz, C_6D_6, 25.1 °C): δ [ppm] = 25.6 (s).

IR (neat): $\tilde{\nu}$ [cm^{-1}] = 3270 (vw), 3120 (vw), 2946 (m), 2865 (m), 2806 (w), 2770 (w), 1644 (vs), 1620 (vs), 1545 (m), 1467 (s), 1421 (w), 1376 (w), 1363 (w), 1232 (w), 1190 (w), 1156 (m), 1007 (s), 940 (m), 880 (w), 851 (vw), 831 (w), 815 (w), 794 (vs), 736 (m), 715 (m).

C,H,N-analysis: calc. (%) for $C_{12}H_{20}NOP$: C 63.98, H 8.95, N 6.22; found: C 63.72, H 8.90, N 6.23.

Bis[(*μ*-chlorido)(pentamethylcyclopentadienyl)cobalt(II)] Co4[11,12]

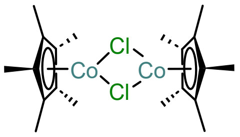

Co4
$C_{20}H_{30}Cl_2Co_2$
459.23 g/mol

*1) Synthesis of Cp*Li:*

Pentamethylcyclopentadiene (2.47 mL, 15.8 mmol, 1.00 eq.) was dissolved in hexane (70 mL) and cooled to –70 °C. Then, *n*-butyllithium (1.6 M in hexane, 10.1 mL, 16.2 mmol, 1.02 eq.) was added dropwise over 10 min while stirring vigorously. The colourless solution was stirred at –70 °C for 30 min and then allowed to warm up to room temperature and stirred for an additional 90 h[5]. After 3 h of stirring, a colourless precipitate formed. The cream suspension was filtrated, and the colourless solid residue was washed using hexane (4x15 mL). The white solid Cp*Li (1.60 g, 11.2 mmol, 71%) was then dried *in vacuo*.

*2) Synthesis of [Cp*CoCl]₂* **Co4**

Inside a glovebox filled with argon, anhydrous $CoCl_2$ (1.42 g, 10.9 mmol, 1.00 eq.) was suspended in –35 °C cold THF (38 mL) and to the blue suspension was added Cp*Li (1.59 g,

[4] The two methyl groups of the isopropyl groups seem to be chemically inequivalent. The signal at 0.98 ppm seems to consist of two overlapping dd which each arise from independent $^{3}J_{(H,H)}$ and $^{3}J_{(P,H)}$ coupling.
[5] Surprisingly, the reaction is tremendously slow in hexane. Filtration of the reaction mixture after 23 h resulted in a yield of 40 % of Cp*Li, while the filtrate underwent further reaction.

11.2 mmol, 1.03 eq.) in portions over 12 min while stirring. During the addition of Cp*Li, the blue suspension turned green and then became a black solution. The reaction mixture was stirred at room temperature for 1.5 h and was then concentrated under reduced pressure. Subsequently, the black solid residue was shortly dried under oil pump vacuum and then taken up in hexane (60 mL). The black suspension was filtrated, and the black-brown solid residue was extracted using additional hexane (2x40 mL, 1x20 mL). Afterwards, the black filtrate was concentrated to approximately 50 mL, cooled to –30 °C for 10 min and then down to –78 °C for 2 h. Decantation at –78 °C and washing with –90 °C cold hexane (8 mL) gave dinuclear complex **Co4** (1.76 g, 3.84 mmol, 71%) as black microcrystals.

m.p. = 174 °C (decomposition).

^{1}H-NMR (300.2 MHz, C_6D_6, 24.9 °C): δ [ppm] = 38.26 (br, s).

C,H-analysis: calc. (%) for $C_{20}H_{30}Cl_2Co_2$: C 52.31, H 6.58; found: C 52.02, H 6.80.

Diisopropyl cobalt pyridonate complex Co2

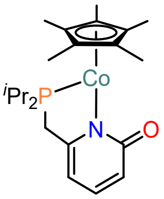

Co2
$C_{22}H_{34}CoNOP$
418.43 g/mol

Inside a glovebox filled with argon, the pyridone **L1H** (699 mg, 3.10 mmol, 1.00 eq.) and potassium *tert*-butoxide (373 mg, 3.32 mmol, 1.07 eq.) were dissolved in THF (130 mL) and stirred at ambient temperature for 2 h. Subsequently, a solution of $[Cp^*CoCl]_2$ **Co4** (713 mg, 1.55 mmol, 0.500 eq.) in THF (7 mL) was added to the reaction mixture in a dropwise condition over 10 min while stirring. During the addition, the reaction mixture turned from slightly yellow to dark red (almost black). The reaction solution was then stirred at ambient temperature for 20.5 h. After completion, the solvent was removed under reduced pressure and the orange solid residue was taken up in toluene (20 mL) before filtrating it over a short pad of Celite® (~2 cm). Afterwards, the red-orange residue was further extracted using toluene (1x10 mL, 2x5 mL). The filtrate was concentrated *in vacuo* and the dark red solid residue was washed with hexane (1x4 mL, 1x2 mL) by decantation. Subsequently, the dark red residue was dried and recrystallised from MeCN (63 mL) at –30 °C to give analytically pure **Co2**[6] (924 mg, 2.21 mmol, 71%) as dark red crystals.[7]

m.p. = 184–186 °C.

Evans-NMR (300.2 MHz, C_6D_6, 24.9 °C): μ = 1.89 μ_B, unpaired electrons (spin-only) n = 1.

^{1}H-NMR (300.2 MHz, C_6D_6, 24.9 °C): δ [ppm] = 5.32 (br, s), 3.29 (br, s), –0.51 (br, s).

IR (neat): $\tilde{\nu}$ [cm^{-1}] = 3069 (vw), 3019 (vw), 2974 (w), 2964 (w), 2944 (w), 2935 (w), 2899 (m), 1618 (vs), 1557 (s), 1519 (vs), 1458 (s), 1374 (vs), 1242 (m), 1191 (w), 1158 (w), 1139 (m), 1126 (m), 1095 (w), 1037 (m), 1070 (w) 1010 (m), 954 (w), 884 (m), 826 (vs), 783 (s), 764 (m), 725 (vs), 654 (s).

UV-Vis (THF, 0.05 mg/mL): λ_{max} (ε) = 396 nm (3358 $L\cdot mol^{-1}\cdot cm^{-1}$), 332 nm (8633 $L\cdot mol^{-1}\cdot cm^{-1}$), 240 nm (15906 $L\cdot mol^{-1}\cdot cm^{-1}$), 209 nm (18409 $L\cdot mol^{-1}\cdot cm^{-1}$).

C,H,N-analysis: calc. (%) for $C_{22}H_{34}CoNOP$: C 63.15, H 8.19, N 3.35; found: C 62.87, H 8.28, N 3.28.

ESI-MS (*m/z*): calc. for $C_{22}H_{34}CoNOP^+$ $[M]^+$: 418.1710, found: 418.1649.

6-((Di-*tert*-butylphosphanyl)methyl)pyridin-2(1*H*)-one L2H

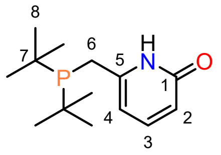

L2H
$C_{14}H_{24}NOP$
253.33 g/mol

To a suspension of 6-methylpyridin-2(1*H*)-one[8] (1.50 g, 13.8 mmol, 1.00 eq.) in THF (15 mL) was added *n*-butyllithium (1.6 M in hexane, 18 mL, 29 mmol, 2.1 eq.) in a dropwise manner over a period of 20 min while stirring at 0 °C. During the addition, the colorless suspension turned into an orange solution. The reaction solution was then stirred at 0 °C for 2 h. Subsequently, the reaction mixture was cooled to –78 °C and added dropwise to a solution of di-*tert*-butylchlorophosphine (2.60 mL, 13.7 mmol, 0.996 eq.,) in THF (10 mL) at –78 °C over a period 20 min with vigorous stirring. After stirring the red reaction mixture at –78 °C for 2 h,

[6] The compound is sensitive towards air.
[7] When recrystalising from MeCN, considerable amounts of a fine orange powder stay undissolved. The insoluble orange solid is not dissolved by adding additional MeCN or increasing the temperature. Quite the opposite; increasing the temperature to 40 °C leads to precipitation of an insoluble green solid. Therefore, dissolving the crude solid at room temperature followed by inert filtration is necessary prior to cooling. It is advised to combine the undissolved orange solid with the mother liquid after the first recrystallisation and repeat the process to increase the yield.
[8] 6-Methylpyridine-2(1*H*)-one was purified by sublimation (1x10^{-3} mbar, 100 °C) prior to use.

it was allowed to warm up to room temperature and stirred for an additional 18 h. The solvent was removed under reduced pressure and the syrupy, red residue was quenched with degassed[9] sat. aqueous NH_4Cl solution (30 mL). The aqueous suspension was stirred at room temperature for 90 min, during which time the orange foam gradually dissolved until only a colorless solid remained. Afterwards, the yellow suspension was extracted with CH_2Cl_2 (4x15 mL) and the combined organic layers were dried over anhydrous $MgSO_4$, filtered and concentrated under reduced pressure. The remaining yellow solid was then recrystallised from hexane (20 mL, reflux)[10]. To remove remnants of 6-methylpyridin-2(1*H*)-one[11], the slightly yellow crystals were further purified by sublimation ($1x10^{-3}$ mbar, 70 °C) to give target phosphine **L2H**[12] (2.04 g, 8.04 mmol, 59%) as slightly yellow crystals.

m.p. = 130–131 °C.

^{1}H-NMR (400.1 MHz, C_6D_6, 26.5 °C): δ [ppm] = 14.32 (s, 1 H, -NH), 6.89 (dd, $^3J_{(H,H)}$ = 9.0 Hz, $^3J_{(H,H)}$ = 7.0 Hz, 1 H, 3-H), 6.43 (d, $^3J_{(H,H)}$ = 9.0 Hz, 1 H, 2-H), 6.32–6.28 (m, 1 H, 4-H), 2.77 (d, $^2J_{(P,H)}$ = 2.3 Hz, 2 H, 6-H), 1.06 (d, $^3J_{(P,H)}$ = 11.0 Hz, 18 H, 8-H).

^{13}C-DEPTQ-NMR (100.6 MHz, C_6D_6, 26.5 °C): δ [ppm] = 166.4 (C-1), 151.2 (d, $^2J_{(P,C)}$ = 16.7 Hz, C-5), 141.6 (C-3), 116.2 (d, $^5J_{(P,C)}$ = 1.7 Hz, C-2), 106.8 (d, $^3J_{(P,C)}$ = 14.6 Hz, C-4), 31.8 (d, $^1J_{(P,C)}$ = 23.4 Hz, C-7), 29.6 (d, $^2J_{(P,C)}$ = 14.0 Hz, C-8), 25.8 (d, $^1J_{(P,C)}$ = 26.5 Hz, C-6).

^{31}P{^{1}H}-NMR (162.0 MHz, C_6D_6, 26.5 °C): δ [ppm] = 32.7 (s).

IR (neat): $\tilde{\nu}$ [cm^{-1}] = 3111 (vw), 3080 (vw), 2938 (m), 2897 (m), 2860 (m), 2796 (w), 1648 (vs), 1622 (vs), 1544 (m), 1447 (m), 1423 (m), 1399 (w), 1387 (w), 1363 (m), 1243 (w), 1208 (vw), 1175 (m), 1158 (m), 1063 (vw), 1010 (m), 968 (w), 887 (m), 818 (s), 789 (vs), 762 (m), 736 (m), 723 (s).

C,H,N-analysis: calc. (%) for $C_{14}H_{24}NOP$: C 66.38, H 9.55, N 5.53; found: C 66.41, H 9.48, N 5.61.

Di-*tert*-butyl cobalt pyridonate complex Co3

[9] A nitrogen stream was bubbled through the aqueous solution for 1 h.
[10] A hot filtration is necessary before crystallisation to remove remnants of insoluble particles.
[11] After the first purification by recrystallisation, the product is contaminated with 5% of starting material.
[12] The compound is sensitive towards air.

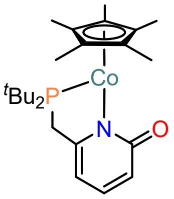

Co3
$C_{24}H_{38}CoNOP$
446.48 g/mol

Inside a glovebox filled with argon, the pyridone **L2H** (200 mg, 0.790 mmol, 1.00 eq.) and potassium *tert*-butoxide (93 mg, 0.83 mmol, 1.05 eq.) were dissolved in THF (35 mL) and stirred at ambient temperature for 2 h. Subsequently, a solution of $[Cp^{*}CoCl]_2$ **Co4** (181 mg, 0.394 mmol, 0.499 eq.) in THF (5 mL) was added to the reaction mixture in a dropwise condition over 7 min while stirring. During the addition, the reaction mixture turned from slightly yellow to dark red (almost black). The reaction solution was then stirred at ambient temperature for 21.5 h. After completion, the solvent was removed under reduced pressure, the dark red oily residue was taken up in toluene (5 mL) and filtrated. The red solid residue was further extracted using toluene (5 mL) and the filtrate was concentrated *in vacuo*. Subsequently, the red-brown residue was dried and recrystallised from hexane (45 mL) at –30 °C to give analytically pure **Co3**[13] (191 mg, 0.428 mmol, 54%) as red-orange crystals.[14]

m.p. = 168–169 °C.

Evans-NMR (400.1 MHz, C_6D_6, 26.4 °C): μ = 1.83 μ_B, unpaired electrons (spin-only) n = 1.

^{1}H-NMR (300.2 MHz, C_6D_6, 24.9 °C): δ [ppm] = 6.19 (br, s), 3.33 (br, s), –1.68 (br, s), –5.73 (br, s).

IR (neat): $\tilde{\nu}$ [cm^{-1}] = 2945 (w), 2891 (w), 1621 (s), 1561 (s), 1522 (vs), 1474 (m), 1414 (m), 1389 (m), 1374 (s), 1366 (s), 1251 (m), 1180 (m), 1150 (m), 1133 (m), 1076 (w), 1020 (s), 957 (m), 941 (m), 828 (vs), 810 (s), 784 (s), 763 (s), 731 (vs), 667 (m).

UV-Vis (THF, 0.05 mg/mL): λ_{max} (ε) = 397 nm (shoulder, 3152 L·mol^{-1}·cm^{-1}), 347 nm (9340 L·mol^{-1}·cm^{-1}), 287 nm (10471 L·mol^{-1}·cm^{-1}).

C,H,N-analysis: calc. (%) for $C_{24}H_{38}CoNOP$: C 64.56, H 8.58, N 3.14; found: C 64.69, H 8.63, N 3.25.

Cobalt(III) hydride Co6 and cobalt dimer Co8

[13] The compound is sensitive towards air.
[14] Orange crude solid that does not dissolve at room temperature in hexane, dissolves readily when heating to 50 °C. Hot filtration was performed prior to cooling.

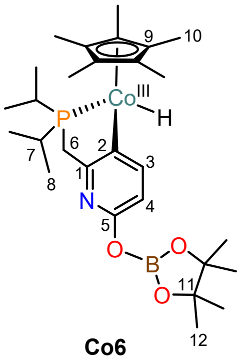

Co6
$C_{28}H_{46}BCoNO_3P$
545.40 g/mol

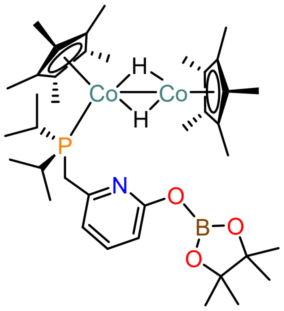

Co8
$C_{38}H_{63}BCo_2NO_3P$
741.58 g/mol

Inside a glovebox filled with argon, **Co2** (157 mg, 0.375 mmol, 1.00 eq.) was dissolved in THF (3 mL) and to the dark red solution was added pinacolborane (65 µL, 0.45 mmol, 1.2 eq.). Upon addition of HBpin, the solution turned black. The reaction mixture was then stirred at ambient temperature for 26 h. Subsequently, the volatile components were removed under reduced pressure yielding a viscous black oil. The residual oil was then coevaporated with hexane thrice (3x1 mL) giving a black solid. To the black solid was added hexane (25 mL) and the resulting suspension (black suspension with orange solid) was filtrated. The residual orange solid (**Co6**, 10 mg, 18 µmol) was washed with hexane (3 mL) and the filtrate was left for crystallisation at –35 °C. The orange crystals were collected by decantation and washed with –35 °C cold hexane until all of the black staining was removed (5x1 mL). Drying of the orange crystals in the glovebox atmosphere for 4 h followed by a short application of vacuum (3 min) yielded **Co6** (33 mg, 61 µmol) as orange crystals. The mother liquid was concentrated to approximately 6 mL and left for crystallisation at –35 °C. The crystals were collected by decantation and washed with –35 °C cold hexane until all of the black staining was removed (5x1 mL). Drying of the orange crystals in the glovebox atmosphere for 4 h followed by a short application of vacuum (3 min) yielded the third batch of **Co6** (18 mg, 33 µmol) as golden crystals. Combined yield of **Co6** (61 mg, 0.11 mmol, 30%).

The mother liquid was concentrated to approx. 1.5 mL, filtered and left at –35 °C for crystallisation yielding black crystals suitable for XRD analysis. NMR analysis of the black crystals revealed that the dimeric cobalt species **Co8** identified by XRD analysis either co-crystallises with other cobalt hydride species or interconverts into them in solution (see Figure 27 and 28).

Characterisation data of **Co6***:*

^{1}H-NMR (300.2 MHz, C_6D_6, 22.9 °C): δ [ppm] = 7.74 (d, $^{3}J_{(H,H)}$ = 8.1 Hz, 1 H, 3-H), 6.92 (d, $^{3}J_{(H,H)}$ = 8.1 Hz, 1 H, 4-H), 2.81–2.48 (m, 2 H, 6-H), 1.67 (s, 15 H, 10-H), 1.08 (s, 12 H, 12-H), 0.99–0.62 (m, 14 H, 7-H and 8-H), –16.50 (d, $^{2}J_{(P,H)}$ = 87.3 Hz, 1 H, Co-*H*).

^{13}C{^{1}H}-NMR (150.9 MHz, C_6D_6, 24.9 °C): δ [ppm] = 165.0 (d, $^{2}J_{(P,C)}$ = 19.9 Hz, C-1), 157.1 (C-5), 149.9 (C-4), 148.4 (C-2), 110.4 (C-3), 92.3 (d, $^{2}J_{(P,C)}$ = 1.6 Hz, 5 C, C-9), 82.9 (2 C, C-11), 36.8 (d, $^{1}J_{(P,C)}$ = 39.8 Hz, C-6), 26.5 (d, $^{1}J_{(P,C)}$ = 12.6 Hz, C-7$^{a/b}$), 24.7 (4 C, C-12), 22.8 (d, $^{1}J_{(P,C)}$ = 25.4 Hz, C-7$^{a/b}$), 19.3 (C-8$^{a/b/c/d}$), 18.4 (d, $^{2}J_{(P,C)}$ = 2.2 Hz, C-8$^{a/b/c/d}$), 18.1 (2 C, C-8$^{a/b/c/d}$), 11.1 (5 C, C-10).

^{11}B-NMR (192.6 MHz, C_6D_6, 24.9 °C): δ [ppm] = 22.81 (br, s).

^{31}P{^{1}H}-NMR (243.0 MHz, C_6D_6, 24.9 °C): δ [ppm] = 93.27 (br, d).

C,H,N-analysis: calc. (%) for $C_{28}H_{46}BCoNO_3P$: C 61.66, H 8.50, N 2.57; found: C 61.71, H 8.67, N 2.57.

ESI-MS (*m/z*): calc. for $C_{28}H_{46}BCoNO_3P^+$ $[M]^+$: 545.2640, found: 545.2546.

3. Double Hydroboration of Pyridines

3.1 Comparison of Cobalt Catalysts and General Procedure for NMR Scale Double Hydroboration Reactions

Inside a glovebox filled with argon, an oven-dried scintillation vial was charged with the respective cobalt pyridonate complex (6 µmol, 3 mol%) and the catalyst was dissolved in C_6D_6 or toluene-d_8 (0.6 mL). To the solution was added internal standard (hexamethylbenzene for C_6D_6, 1,3,5-trimethoxybenzene for toluene-d_8; 0.02 mmol) either by addition of an aliquot of a stock solution in C_6D_6 (0.4 M, 50 µL) or directly as a solid. Then, the respective pyridine (0.20 mmol, 1.0 eq.) was added first followed by the addition of pinacolborane (64 µL, 0.44 mmol, 2.2 eq.). The reaction mixture was mixed with a Pasteur pipette before transferring it to a J. Young NMR tube. Subsequently, the NMR tube was closed and taken out of the glovebox. The reaction mixtures were then heated to 50 °C (C_6D_6) or 100 °C (tol-d_8) for the respective times and afterwards immediately analysed by ^{1}H- and ^{11}B-NMR spectroscopy.

3.2 Identification of reaction products

Monitoring of the double hydroboration of 4-phenylpyridine **1b**:

4-Phenylpyridine **1b** (0.2 mmol) was reacted in C_6D_6 according to general procedure 3.1 and monitored by NMR spectroscopy.

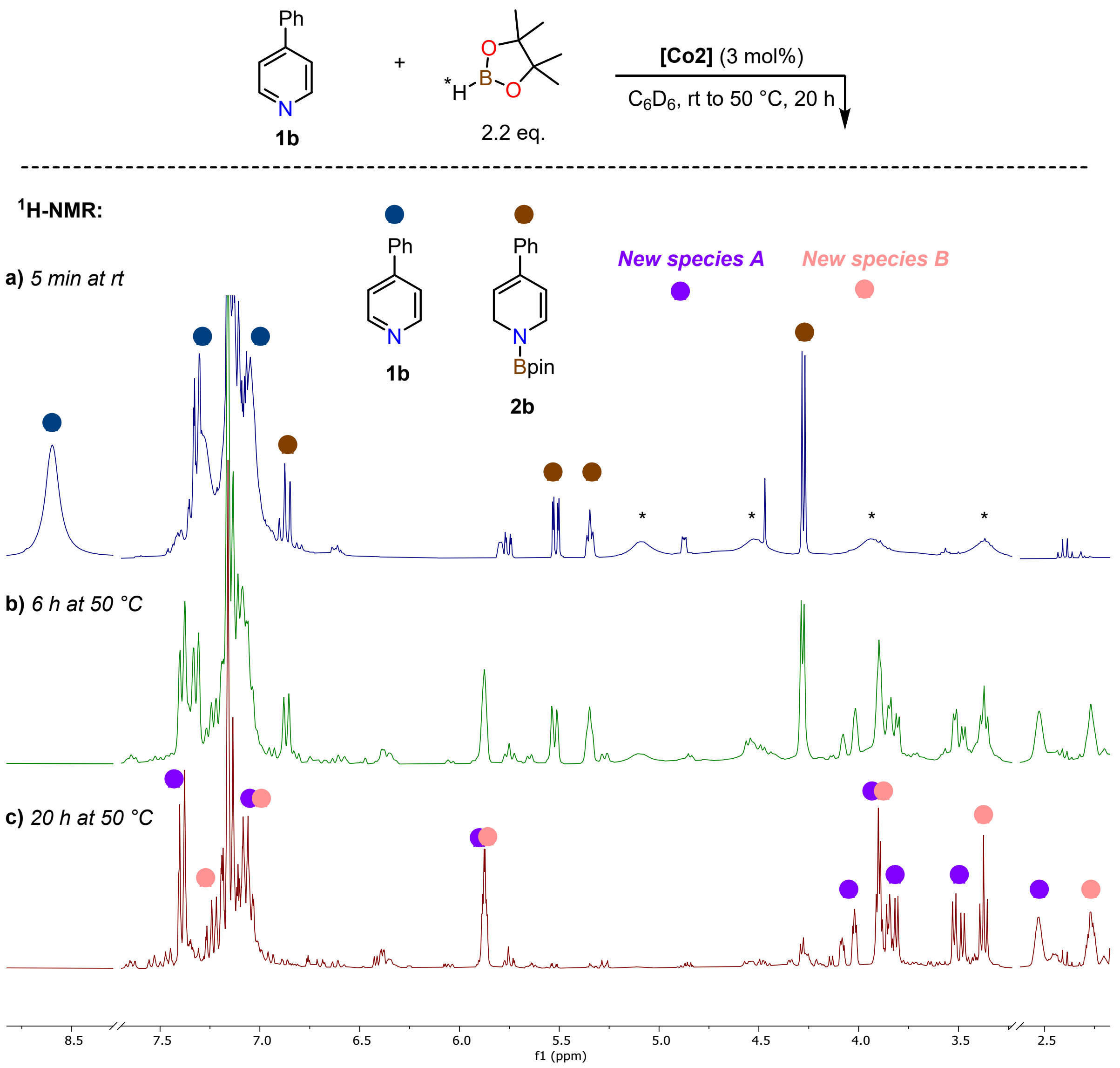

Scheme S1: Monitoring the reaction of 4-phenylpyridine **1b** with HBpin in the presence of **Co2** (3 mol%) by ^{1}H-NMR spectroscopy. a) Reaction mixture after 5 min at room temperature. b) Reaction mixture after 6 h at 50 °C. c) Reaction mixture after 20 h at 50 °C. (*) Signal of pinacolborane.

When 4-phenylpyridine **1b** is mixed with 2.2 eq. of HBpin in the presence of 3 mol% **Co2** in C_6D_6 and immediately analysed by ^{1}H-NMR spectroscopy (about 5 min at room temperature), the single hydroboration product **2b** is already observed (Scheme S1a). After heating the reaction mixture to 50 °C for 6 h, the starting material is completely consumed, and together with **2b** two new species are observed in the ^{1}H-NMR spectrum (Scheme S1b). The 1,4-dihydropyridine isomer was not detected. After 20 h at 50 °C, *N*-boryl 1,2-dihydropyridine

(DHP) **2a** and HBpin are fully converted to the unidentified species (Scheme S1c). ^{11}B-NMR spectroscopy of the crude mixture after 20 h at 50 °C reveals the presence of both a R_3C–$B(OR)_2$ as well as a R_2N–$B(OR)_2$ species as indicated by two signals at 33.3 ppm and 24.0 ppm, respectively (Scheme S2). Addition of a third equivalent of HBpin to the reaction mixture followed by heating to 50 °C for another 20 h did not induce any changes to the ^{1}H-NMR spectrum. The same reaction with an initial loading of 3.3 eq. of HBpin gave similar results. Derivatisation with 1.1 eq. of trifluoroacetic anhydride followed by GC-MS analysis (see procedure 10.2) gave the mass to charge ratio of a trifluoroacetylated double hydroboration product (*N*-boryl group substituted for trifluoroacetyl group, $[M]^{\bullet+}$ = *m/z* 381.2) as the main component (Scheme S2). A peak with the mass of an acylated tetrahydropyridine without a carbon-boryl group was also detected ($[M]^{\bullet+}$ = *m/z* 255.1). Thus, both ^{1}H-NMR as well as GC-MS analysis indicate that 4-phenylpyridine **1b** is hydroborated twice but not a third time. ^{1}H-, ^{13}C-, COSY, HSQC and NOESY NMR analysis suggest that the predominantly formed product is double hydroborated allylic boronate **3b** in 42% (NMR) yield (Scheme S2). The ^{1}H-NMR spectrum shows only one signal corresponding to an alkenyl proton at 5.89–5.86 ppm. Additionally, a strong NOE signal is observed between the vinylic proton and the aryl protons. This indicates the presence of a 1,2,3,6-tetrahydropyridine species rather than a 1,2,3,4-tetrahydropyridine (enamine) isomer. Furthermore, four diastereotopic proton signals for the respective CH_2N-groups are detected between 3.3 ppm and 4.1 ppm. A strong NOE signal between the aryl protons and the hydrogen atom geminal to the boryl moiety is also observed. Together, this rules out the presence of the *α*-Bpin isomer. The signals of the major side product (18% yield) were assigned to mono-borylated tetrahydropyridine **3b’** (Scheme S2). Other minor species could not be assigned.

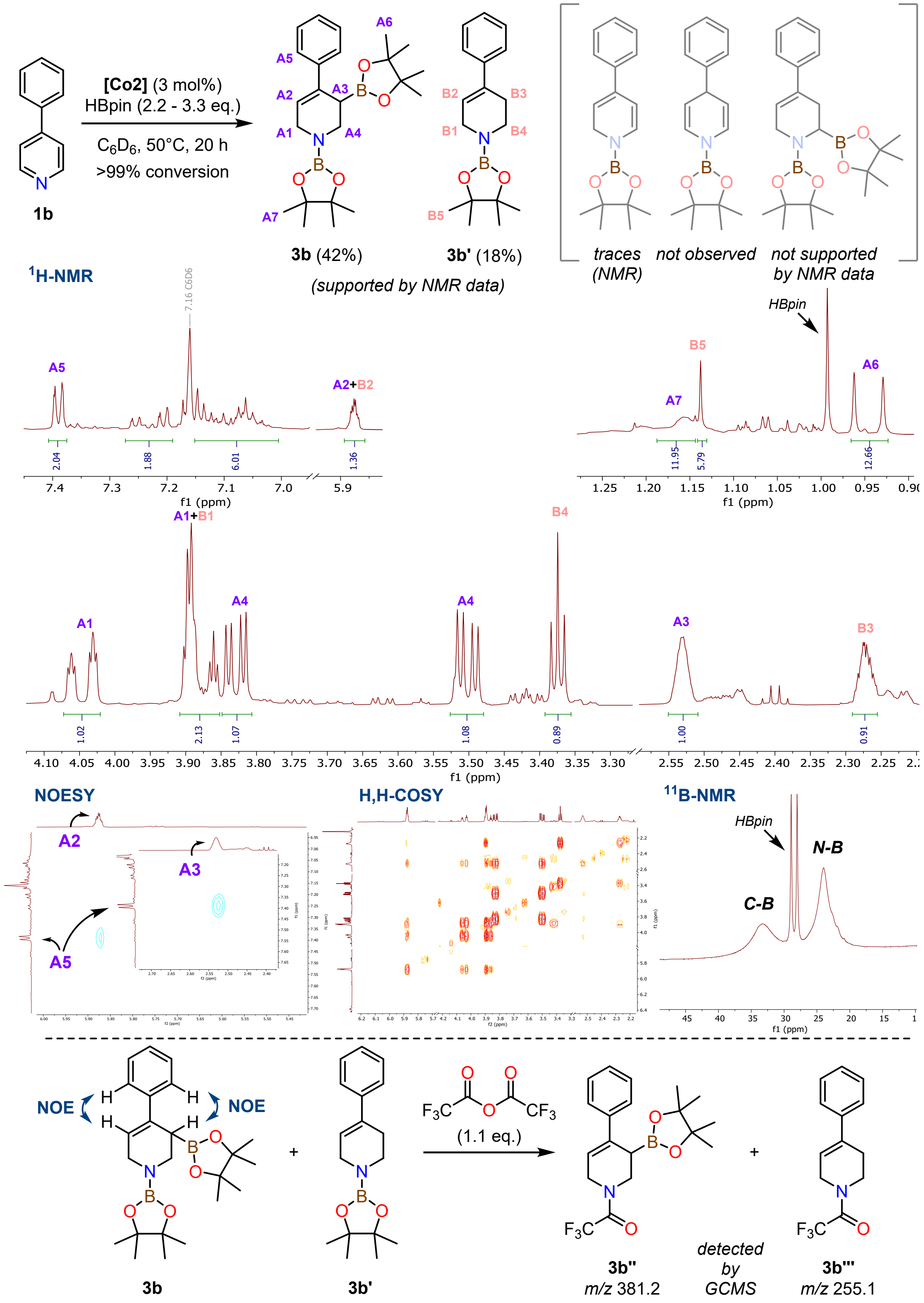

Scheme S2: Products formed in the reaction of 4-phenylpyridine **1b** (0.2 mmol, 1.00 eq.) with HBpin (2.2-3.3 eq.) in the presence of **Co2** (3 mol%) at 50 °C after 20 h as identified by NMR analysis of the crude mixture and GC-MS after acylation. The yields were determined by ^{1}H-NMR based on internal standard (hexamethylbenzene).

Confirmation of the identity of mono-borylated tetrahydropyridine *byproduct* **3b'***:*

To confirm that the major side product observed during the reaction of 4-phenylpyridin **3b** with HBpin is indeed mono-borylated tetrahydropyridine **3b'**, pure 4-phenyl-1,2,3,6-tetrahydropyridine **8** was isolated after protodeboronation of **3b** (refer to procedure 10.3) and reacted with 1.05 eq. HBpin (refer to procedure 10.4) to generate **3b'** *in situ*. Subsequent, ^{1}H-NMR analysis indicated that tetrahydropyridine **8** was readily deprotonated by HBpin resulting in the formation of hydrogen (singlet at 4.47 ppm)[13] and *N*-boryl species **3b'**. An overlay of the ^{1}H-NMR spectra of the crude mixture of 4-phenylpyridine **1b** hydroboration and *in situ* generated **3b'** gives a perfect match (Scheme S3). The data thereby clearly suggests that the other species formed during the hydroboration of 4-phenylpyridine **1b** is indeed mono(boryl) tetrahydropyridine **3b'**.

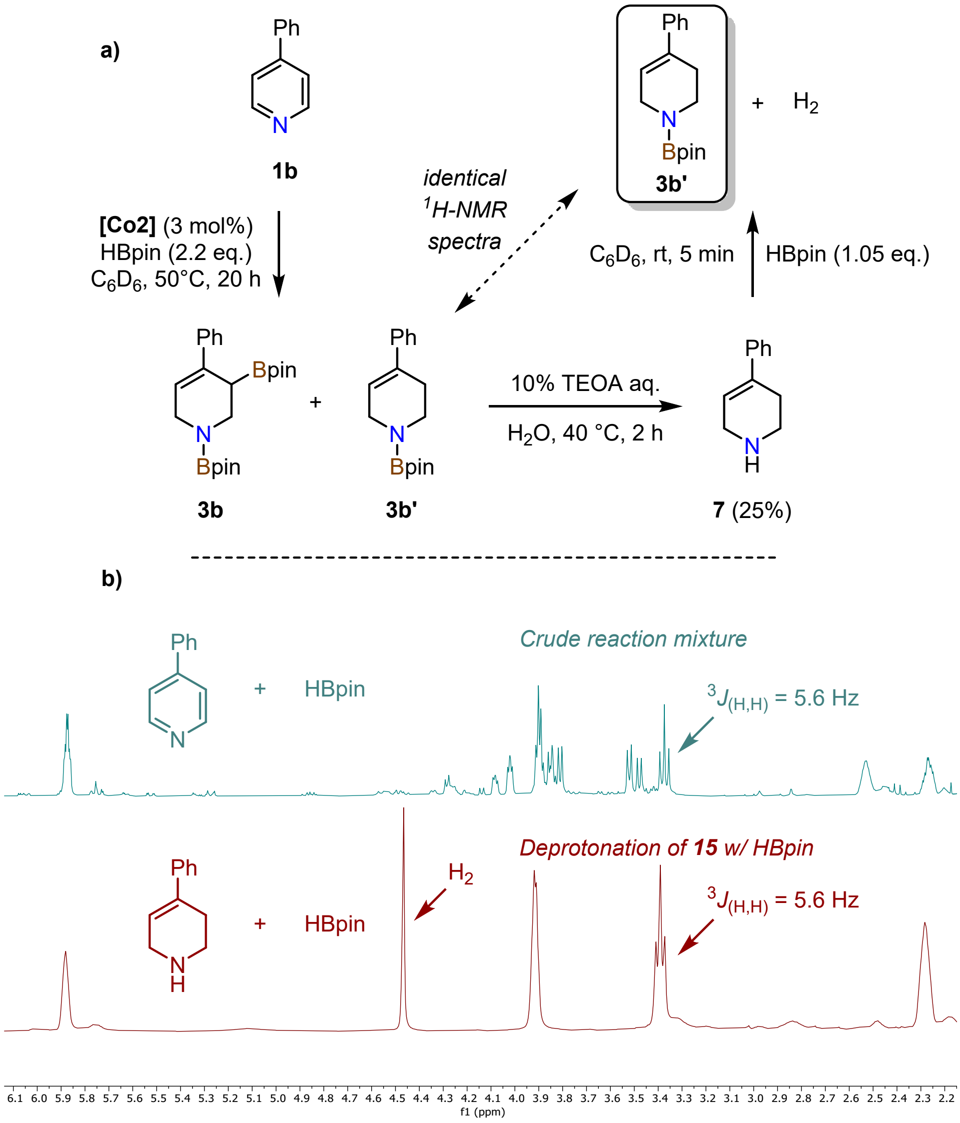

Scheme S3: a) Reaction sequence for the *in situ* formation of **3b'** to confirm the structure of the major side product of the double hydroboration of 4-phenylpyridine **1b**. b) Overlay of the ^{1}H-NMR spectra of the crude hydroboration mixture (upper turquoise) and with HBpin deprotonated **7**. The overlay gives a perfect match.

3.3 General Procedure for Reaction Optimisation

Inside a glovebox filled with argon, an oven-dried headspace vial (5 mL) was charged with precatalyst **Co2** (1–7 mol%) and a magnetic stirring bar. The precatalyst was dissolved in the respective solvent and to the red solution was added first 4-methylpyridine **1a** (0.20 mmol, 1.0 eq.)[15] and subsequently pinacolborane (1.0–3.3 eq.) During the addition of HBpin the reaction mixture turned black. The headspace vial was closed, taken out of the glovebox, and stirred at the respective temperature for 20 h. Subsequently, the vial was opened inside the glovebox and to the reaction mixture was added internal standard (hexamethylbenzene for C_6D_6 and THF-d_8, 1,3,5-trimethoxybenzene for toluene-d_8; 0.02 mmol) either by addition of an aliquot of a stock solution in C_6D_6 (0.4 M, 50 µL) or directly as a solid. After fully dissolved, the crude mixture was transferred to a J. Young NMR tube and analysed by ^{1}H-NMR spectroscopy to determine the yield, conversion, and product ratio (Table S1).

[15] If the amount of catalyst became too low for reliable weighing or the amount of solvent became to little so that solvent evaporation greatly influenced the concentration, the reactions were performed on 0.4 mmol scale.

Table S1: Variation of the reaction conditions for the double hydroboration of 4-methylpyridine **1a**.

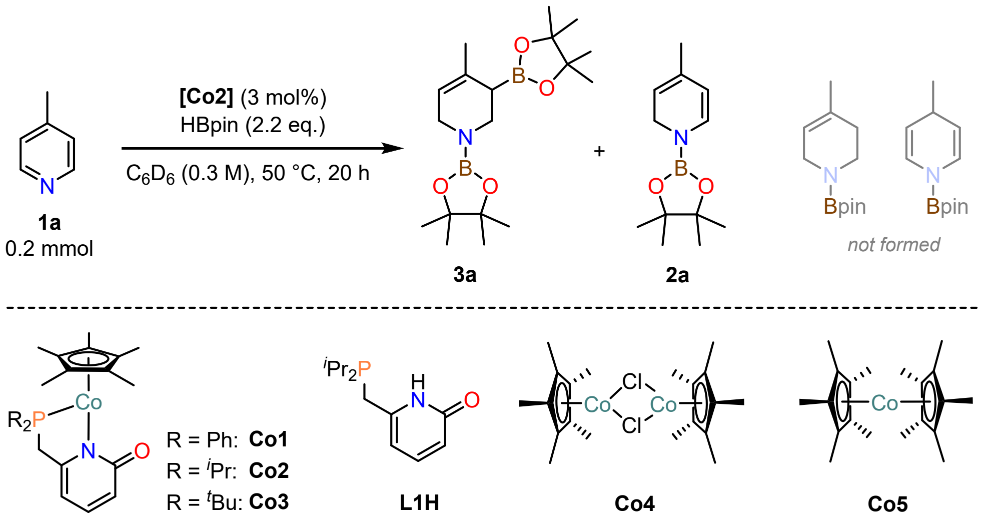

	Entry	Sol.	HBpin (eq.)	T (°C)	Cat. (mol%)	Conc. (M)	Conv. (%)	Yield[a] (%) 3a	2a
**Cat.**	1	C_6D_6	2.20	50	**Co2** (3)	0.33	95	56	12
	2[b]	C_6D_6	2.20	50	**Co3** (3)	0.33	>99[c]	32	traces
	3[b]	C_6D_6	2.20	50	**Co1** (3)	0.33	5	0	4
**Sol.**	4	THF-d_8	2.20	50	**Co2** (3)	0.33	>99	16	60
	5	Tol-d_8	2.20	50	**Co2** (3)	0.33	96	44	34
**Load.**	6[d]	C_6D_6	2.20	50	**Co2** (1)	0.33	70	35	14
	7	C_6D_6	2.20	50	**Co2** (5)	0.33	97	48	19
	8	C_6D_6	2.20	50	**Co2** (7)	0.33	98	44	24
	9	Tol-d_8	2.20	100	**Co2** (5)	0.33	98	72	4
**Temp.**	10	C_6D_6	2.20	25	**Co2** (3)	0.60	33	traces	18
	11	C_6D_6	2.20	70	**Co2** (3)	0.33	87	57	12
	12	Tol-d_8	2.20	80	**Co2** (3)	0.33	94	68	8
	13	Tol-d_8	2.20	100	**Co2** (3)	0.33	92	70	4
**Eq.**	14	C_6D_6	1.00	50	**Co2** (3)	0.33	74	14	60
	15	C_6D_6	3.30	50	**Co2** (3)	0.33	95	56	13
	16	Tol-d_8	3.30	100	**Co2** (3)	0.33	96	78	5
**Conc.**	**17[d]**	**C_6D_6**	**2.20**	**50**	**Co2 (3)**	**0.60**	**96**	**63[e]**	**8**
	18[d]	C_6D_6	2.20	50	**Co2** (3)	1.0	>99	46	26
	**19**	**Tol-d_8**	**2.20**	**100**	**Co2 (3)**	**0.60**	**98**	**86**	**4**
**Test**	20[d]	Tol-d_8	2.20	100	–	0.60	0	–	–
	21[d]	Tol-d_8	2.20	100	**L1H** (3)	0.60	0	–	–
	22[d]	Tol-d_8	2.20	100	**L1H** (3) + KOtBu	0.60	55	0	1
	23[d]	Tol-d_8	2.20	100	**Co4** (3)	0.60	>99[f]	0	5
	24	Tol-d_8	2.20	100	**Co5** (3)	0.60	33	0	10

Conditions: **1a** (0.20 mmol, 1.0 eq.), HBpin (1.0–3.3 eq.), [Cat.] (1–7 mol%), solvent (C_6D_6, THF-d_8, tol-d_8), concentration (0.33–1.0 M), 25–100 °C, 20 h inside a headspace vial (4 mL) with stirring unless otherwise specified. *a*) Yields determined by ^{1}H-NMR using hexamethylbenzene (C_6D_6) or trimethoxybenzene (tol-d_8) as an internal standard. *b*) Without stirring inside a J. Young NMR tube. *c*) Formation of unidentified byproducts. *d*) 0.4 mmol scale instead of 0.2 mmol scale. *e*) Isolated after *N*-acetylation in 54% yield. *f*) No traceable products detected.

3.4 General Procedure for the Preparative Double Hydroboration–*N*-Acetylation sequence

1) Double Hydroboration of Pyridines

Inside a glovebox filled with argon, an oven-dried headspace vial (5 mL) was charged with precatalyst **Co2** (5 mg, 12 µmol, 3 mol%) and a magnetic stirring bar. The precatalyst was dissolved in C_6D_6 or toluene-d_8 (0.7 mL, 0.6 M) and to the red solution was added first the respective pyridine (0.40 mmol, 1.0 eq.) and subsequently pinacolborane (128 µL, 0.88 mmol, 2.2 eq.). During the addition of HBpin the reaction mixture turned black. The headspace vial was closed, taken out of the glovebox, and stirred at 50 °C (C_6D_6, *Method A*) or 100 °C (toluene-d_8, *Method B*) for 20 h. Subsequently, the vial was opened inside the glovebox and to the reaction mixture was added internal standard (hexamethylbenzene for C_6D_6, 1,3,5-trimethoxybenzene for toluene-d_8; 0.02 mmol) either by addition of an aliquot of a stock solution in C_6D_6 (0.4 M, 50 µL) or directly as a solid. After fully dissolved, the crude mixture was transferred to a J. Young NMR tube and analysed by ^{1}H- and ^{11}B-NMR spectroscopy to determine the yield and product ratio.

2) *N*-Acylation of Double Hydroborated Pyridines

After NMR analysis, the reaction mixture was transferred back into the same headspace vial inside a glovebox and the NMR tube was rinsed with toluene (2x ~0.3 mL) twice prior to re-closing the vial. Then, the vial was taken out of the glovebox and through the septum of the cap was added acetic anhydride (42 µL, 0.44 mmol, 1.1 eq.) at room temperature. Subsequently, the reaction mixture was stirred at room temperature for 48 h. After completion, the vial was opened and the dark green solution was filtered over a short pad of silica gel (~1 cm inside a Pasteur pipette, elution with 8 mL of EtOAc). The filtrate was concentrated under reduced pressure and the dark oily residue was purified by flash chromatography (SiO_2, EtOAc/pentane or CH_2Cl_2/MeOH, UV/$KMnO_4$/vanillin, within 20–30 min) to give the desired compound.

4. Characterisation Data of Double Hydroboration Products

4.1 Characterisation Data of bis(borylated) Tetrahydropyridines

1,4-Bis(4,4,5,5-tetramethyl-1,3,2-dioxaborolan-2-yl)-1,2,3,4-tetrahydropyridine 3c[14]

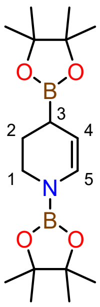

3c
$C_{17}H_{31}B_2NO_4$
335.06 g/mol

The compound was prepared from pyridine **1c** following the general procedure for pyridine double hydroboration described in 3.4 (*Method A*). The product was characterised from the crude spectra. NMR yield: 56%.

^{1}H-NMR (600.1 MHz, C_6D_6, 24.9 °C): δ [ppm] = 6.79 (dd, $^3J_{(H,H)}$ = 8.1 Hz, $^4J_{(H,H)}$ = 2.1 Hz, 1 H, 5-H), 4.96 (dd, $^3J_{(H,H)}$ = 8.1 Hz, $^3J_{(H,H)}$ = 3.6 Hz, 1 H, 4-H), 3.67–3.61 (m, 1 H, 1-H$^{a/b}$)[16], 3.49–3.42 (m, 1 H, 1-H$^{a/b}$), 2.00–1.92 (m, 2 H, 2-H$^{a/b}$ and 3-H), 1.91–1.84 (m, 1 H, 2-H$^{a/b}$), 1.14–0.99 (m, 24 H, Bpin).

^{11}B-NMR (128.4 MHz, C_6D_6, 26.4 °C): δ [ppm] = 33.2 (br, s, C-Bpin), 23.8 (br, s, N-Bpin).

1,3-Bis(4,4,5,5-tetramethyl-1,3,2-dioxaborolan-2-yl)-1,2,3,6-tetrahydropyridine 3c'

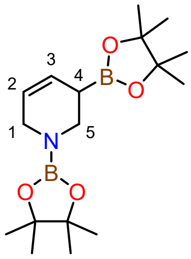

3c'
$C_{17}H_{31}B_2NO_4$
335.06 g/mol

[16] This signal overlaps with a signal of its isomer **3c'**.

The compound was prepared from pyridine **1c** following the general procedure for pyridine double hydroboration described in 3.4 (*Method A*). The product was characterised from the crude spectra. NMR yield: 13%.

^{1}H-NMR (600.1 MHz, C_6D_6, 24.9 °C): δ [ppm] = 6.03 (pseudo-dq, $^3J_{(H,H)}$ = 10.2 Hz, $^{3/4}J_{(H,H)}$ = 2.4 Hz, 1 H, 3-H), 5.56 (pseudo-dq, $^3J_{(H,H)}$ = 10.2 Hz, $^{3/4}J_{(H,H)}$ = 3.0 Hz, 1 H, 2-H), 3.87–3.81 (m, 1 H, 1-$H^{a/b}$), 3.77 (dd, $^2J_{(H,H)}$ = 12.8 Hz, $^3J_{(H,H)}$ = 5.4 Hz, 1 H), 3.70–3.64 (m, 1 H, 1-$H^{a/b}$)[17], 3.41–3.35 (m, 1 H, 5-$H^{a/b}$)[18], 2.08–2.03 (m, 1 H, 4-H), 1.14–0.99 (m, 24 H, Bpin).

^{11}B-NMR (128.4 MHz, C_6D_6, 26.4 °C): δ [ppm] = 33.2 (br, s, C-Bpin), 23.8 (br, s, N-Bpin).

4-Methyl-1,3-bis(4,4,5,5-tetramethyl-1,3,2-dioxaborolan-2-yl)-1,2,3,6-tetrahydropyridine 3a

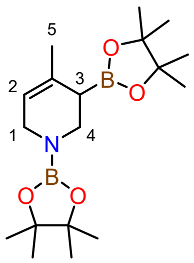

3a
$C_{18}H_{33}B_2NO_4$
349.09 g/mol

The compound was prepared from 4-methylpyridine **1a** following the general procedure for pyridine double hydroboration described in 3.4 (*Method A* and *B*). The product was characterised from the crude spectra. NMR yield: *Method A* = 63%; *Method B* = 86%.

^{1}H-NMR (300.2 MHz, C_6D_6, 22.9 °C): δ [ppm] = 5.33–5.27 (m, 1 H, 2-H), 3.98–3.87 (m, 1 H, 1-$H^{a/b}$), 3.85–3.68 (m, 2 H, 1-$H^{a/b}$ and 4-$H^{a/b}$), 3.41 (dd, $^2J_{(H,H)}$ = 12.5 Hz, $^3J_{(H,H)}$ = 5.0 Hz, 1 H, 4-$H^{a/b}$), 1.85–1.75 (m, 4 H, 5-H and 3-H), 1.22–1.11 (br, m, 12 H, Bpin), 1.07 (s, 6 H, Bpin), 1.05 (s, 6 H, Bpin).

^{11}B-NMR (128.4 MHz, C_6D_6, 26.5 °C): δ [ppm] = 33.3 (br, s, C-Bpin), 23.9 (br, s, N-Bpin).

[17] This signal overlaps with a signal of its isomer **3c**.
[18] This signal overlaps with a signal of an unknown by-product.

4-Phenyl-1,3-bis(4,4,5,5-tetramethyl-1,3,2-dioxaborolan-2-yl)-1,2,3,6-tetrahydropyridine 3b

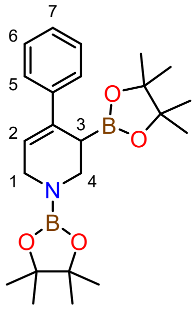

3b
$C_{23}H_{35}B_2NO_4$
411.16 g/mol

The compound was prepared from 4-phenylpyridine **1b** following the general procedure for pyridine double hydroboration described in 3.4 (*Method A* and *B*). The product was characterised from the crude spectra. NMR yield: *Method A* = 42%; *Method B* = 29%.

^{1}H-NMR (600.1 MHz, C_6D_6, 24.9 °C): δ [ppm] = 7.41–7.38 (m, 2 H, 5-H), 7.17–7.04 (m, 3 H, 6-H and 7-H)[19], 5.89–5.86 (m, 1 H, 2-H)[19], 4.05 (br, pseudo-dt, $^{2}J_{(H,H)}$ = 18.4 Hz, $^{3/5}J_{(H,H)}$ = 2.9 Hz, 1 H, 1-H$^{a/b}$), 3.91–3.85 (m, 1 H, 1-H$^{a/b}$)[19], 3.83 (dd, $^{2}J_{(H,H)}$ = 12.5 Hz, $^{3}J_{(H,H)}$ = 4.5 Hz, 1 H, 4-H$^{a/b}$), 3.50 (dd, $^{2}J_{(H,H)}$ = 12.5 Hz, $^{3}J_{(H,H)}$ = 5.0 Hz, 1 H, 4-H$^{a/b}$), 2.55–2.51 (m, 1 H, 3-H), 1.22–0.91 (m, 24 H, Bpin)[19].

^{11}B-NMR (192.6 MHz, C_6D_6, 24.9 °C): δ [ppm] = 33.2 (br, s, C-Bpin), 24.0 (br, s, N-Bpin).

4-Ethyl-1,3-bis(4,4,5,5-tetramethyl-1,3,2-dioxaborolan-2-yl)-1,2,3,6-tetrahydropyridine 3d

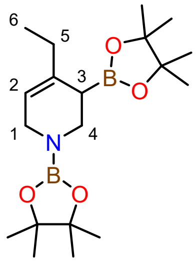

3d
$C_{19}H_{35}B_2NO_4$
363.11 g/mol

[19] This signal overlaps with a signal of the protodeboronation product.

The compound was prepared from 4-ethylpyridine **1d** following the general procedure for pyridine double hydroboration described in 3.4 (*Method A*). The product was characterised from the crude spectra. NMR yield: 71%.

^{1}H-NMR (400.1 MHz, C_6D_6, 26.4 °C): δ [ppm] = 5.30–5.26 (m, 1 H, 2-H), 3.96–3.87 (m, 1 H, 1-$H^{a/b}$), 3.79–3.69 (m, 2 H, 1-$H^{a/b}$ and 4-$H^{a/b}$), 3.35 (dd, $^{2}J_{(H,H)}$ = 12.4 Hz, $^{3}J_{(H,H)}$ = 5.0 Hz, 1 H, 4-$H^{a/b}$), 2.24–2.12 (m, 1 H, 5-$H^{a/b}$), 2.12–1.99 (m, 1 H, 5-$H^{a/b}$), 1.90–1.84 (br, m, 1 H 3-H), 1.20–1.10 (br, m, 12 H, Bpin), 1.07 (s, 6 H, Bpin), 1.06 (s, 6 H, Bpin), 1.03–0.98 (m, 3 H, 6-H)[20].

^{11}B-NMR (128.4 MHz, C_6D_6, 26.4 °C): δ [ppm] = 33.4 (br, s, C-Bpin), 23.8 (br, s, N-Bpin).

4-Isopropyl-1,3-bis(4,4,5,5-tetramethyl-1,3,2-dioxaborolan-2-yl)-1,2,3,6-tetrahydropyridine 3r

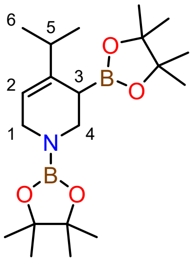

3r
$C_{20}H_{37}B_2NO_4$
377.14 g/mol

The compound was prepared from 4-isopropylpyridine **1r** following the general procedure for pyridine double hydroboration described in 3.4 (*Method A* and *B*). The product was characterised from the crude spectra. NMR yield: *Method A* = 11%; *Method B* = 36%.

^{1}H-NMR (300.1 MHz, toluene-d_8, 25.9 °C): δ [ppm] = 5.31–5.23 (m, 1 H, 2-H), 3.95–3.83 (m, 1 H, 1-$H^{a/b}$), 3.75–3.59 (m, 2 H, 1-$H^{a/b}$ and 4-$H^{a/b}$), 3.20 (dd, $^{2}J_{(H,H)}$ = 12.3 Hz, $^{3}J_{(H,H)}$ = 4.6 Hz, 1 H, 4-$H^{a/b}$), 2.31–2.18 (m, 1 H, 5-H), 1.93–1.85 (m, 1 H, 3-H), 1.20–1.04 (m, 24 H, Bpin), 0.96 (d, $^{3}J_{(H,H)}$ = 6.9 Hz, 6 H, 6-H)[21].

^{11}B-NMR (128.4 MHz, toluene-d_8, 26.3 °C): δ [ppm] = 38.4 (br, s, C-Bpin), 28.9 (br, s, N-Bpin).

[20] This signal overlaps with the signal of HBpin.
[21] This signal overlaps with the signal of HBpin and the methyl groups of the 1,2-single hydroboration product.

4-Benzyl-1,3-bis(4,4,5,5-tetramethyl-1,3,2-dioxaborolan-2-yl)-1,2,3,6-tetrahydropyridine 3e

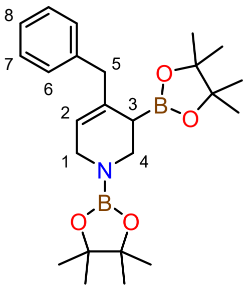

3e
$C_{24}H_{37}B_2NO_4$
425.18 g/mol

The compound was prepared from 4-benzylpyridine **1e** following the general procedure for pyridine double hydroboration described in 3.4 (*Method A*). The product was characterised from the crude spectra. NMR yield: 89%.

^{1}H-NMR (400.1 MHz, C_6D_6, 26.4 °C): δ [ppm] = 7.26–7.20 (m, 2 H, 6-H), 7.19–7.12 (m, 2 H, 7-H), 7.09–7.03 (m, 1 H, 8-H), 5.30–5.23 (m, 1 H, 2-H), 3.98–3.88 (m, 1 H, 1-$H^{a/b}$), 3.82 (dd, $^2J_{(H,H)}$ = 12.4 Hz, $^3J_{(H,H)}$ = 3.4 Hz, 1 H, 4-$H^{a/b}$), 3.72–3.62 (m, 1 H, 1-$H^{a/b}$), 3.52–3.41 (m, 2 H, 5-H), 3.20 (dd, $^2J_{(H,H)}$ = 12.4 Hz, $^3J_{(H,H)}$ = 4.9 Hz, 1 H, 4-$H^{a/b}$), 1.87–1.80 (m, 1 H, 3-H), 1.22–1.05 (m, 24 H, Bpin).

^{11}B-NMR (128.4 MHz, C_6D_6, 26.4 °C): δ [ppm] = 33.3 (br, s, C-Bpin), 23.5 (br, s, N-Bpin).

1,3-Bis(4,4,5,5-tetramethyl-1,3,2-dioxaborolan-2-yl)-4-(trifluoromethyl)-1,2,3,6-tetrahydropyridine 3f

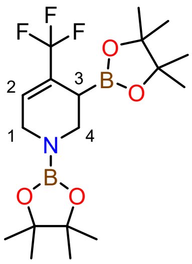

3f
$C_{18}H_{30}B_2F_3NO_4$
403.06 g/mol

The compound was prepared from 4-(trifluoromethyl)pyridine **1f** following the general procedure for pyridine double hydroboration described in 3.4 (*Method A* and *B*). The product was characterised from the crude spectra. NMR yield: *Method A* = 41%; *Method B* = 32%.

^{1}H-NMR (600.1 MHz, C_6D_6, 24.9 °C): δ [ppm] = 5.92–5.87 (m, 1 H, 2-H)[22], 3.84–3.77 (m, 1 H, 1-$H^{a/b}$), 3.73 (dd, $^{2}J_{(H,H)}$ = 12.7 Hz, $^{3}J_{(H,H)}$ = 2.2 Hz, 1 H, 4-$H^{a/b}$), 3.37–3.30 (m, 1 H, 1-$H^{a/b}$), 2.93 (dd, $^{2}J_{(H,H)}$ = 12.7 Hz, $^{3}J_{(H,H)}$ = 4.7 Hz, 1 H, 4-$H^{a/b}$), 2.04–2.01 (m, 1 H, 3-H), 1.21–0.92 (m, 24 H, Bpin)[22].

^{19}F-NMR (564.6 MHz, C_6D_6, 24.9 °C): δ [ppm] = –68.2 (s, CF_3).

^{11}B-NMR (192.6 MHz, C_6D_6, 24.9 °C): δ [ppm] = 32.5 (br, s, C-Bpin), 23.9 (br, s, N-Bpin).

1,3,4-Tris(4,4,5,5-tetramethyl-1,3,2-dioxaborolan-2-yl)-1,2,3,6-tetrahydropyridine 3g

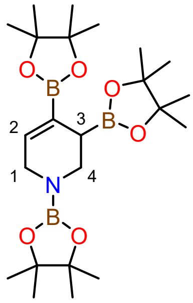

3g
$C_{23}H_{42}B_3NO_6$
461.02 g/mol

The compound was prepared from 4-(4,4,5,5-tetramethyl-1,3,2-dioxaborolan-2-yl)pyridine **1g** following the general procedure for pyridine double hydroboration described in 3.4 (*Method A*). The product was characterised from the crude spectra. NMR yield: 54%.

^{1}H-NMR (400.1 MHz, C_6D_6, 26.4 °C): δ [ppm] = 6.66–6.62 (m, 1 H, 2-H), 3.92–3.84 (m, 1 H, 1-$H^{a/b}$), 3.74–3.64 (m, 2 H, 1-$H^{a/b}$ and 4-$H^{a/b}$), 3.31–3.24 (m, 1 H, 4-$H^{a/b}$)[23], 2.27–2.21 (m, 1 H, 3-H), 1.20–1.01 (m, 36 H, Bpin)[23].

^{11}B-NMR (128.4 MHz, C_6D_6, 26.4 °C): δ [ppm] = 30.7 (br, s, C-Bpin), 24.0 (br, s, N-Bpin).

[22] This signal overlaps with a signal of the protodeboronation product.
[23] This signal overlaps with a signal of the protodeboronation product.

1,3-Bis(4,4,5,5-tetramethyl-1,3,2-dioxaborolan-2-yl)-4-(1-((4,4,5,5-tetramethyl-1,3,2-dioxaborolan-2-yl)oxy)ethyl)-1,2,3,6-tetrahydropyridine 3h

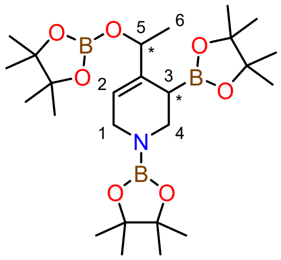

3h
$C_{25}H_{46}B_3NO_7$
505.07 g/mol

The compound was prepared from 4-acetylpyridine **1h** following the general procedure for pyridine double hydroboration described in 3.4 (*Method B*, 4.4 eq. of HBpin). The product was characterised from the crude spectra. NMR yield: 59%.

One diastereomer is preferably formed over the other. According to ^{1}H-NMR analysis, the ratio between the diastereomers amounts to 63:37.

Major diastereomer:

^{1}H-NMR (400.1 MHz, toluene-d_8, 26.4 °C): δ [ppm] = 5.57–5.51 (m, 1 H, 2-H), 4.77–4.66 (overlap with minor diastereomer, m, 1 H, 5-H), 3.88–3.74 (overlap with minor diastereomer, m, 1 H, 1-$H^{a/b}$), 3.67–3.52 (overlap with minor diastereomer, m, 2 H, 1-$H^{a/b}$ and 4-$H^{a/b}$), 3.13 (dd, $^2J_{(H,H)}$ = 12.4 Hz, $^3J_{(H,H)}$ = 4.7 Hz, 1 H, 4-$H^{a/b}$), 1.90–1.85 (m, 1 H, 3-H), 1.30 (overlap with minor diastereomer, d, $^3J_{(H,H)}$ = 6.4 Hz, 3 H, 6-H), 1.21–0.99 (overlap with minor diastereomer, m, 36 H, Bpin)[24].

Minor diastereomer:

^{1}H-NMR (400.1 MHz, toluene-d_8, 26.4 °C): δ [ppm] = 5.45–5.41 (m, 1 H, 2'-H), 4.77–4.66 (overlap with major diastereomer, m, 1 H, 5'-H), 3.88–3.74 (overlap with major diastereomer, m, 1 H, 1'-$H^{a/b}$), 3.67–3.52 (overlap with major diastereomer, m, 2 H, 1'-$H^{a/b}$ and 4'-$H^{a/b}$), 3.22 (dd, $^2J_{(H,H)}$ = 12.5 Hz, $^3J_{(H,H)}$ = 4.9 Hz, 1 H, 4'-$H^{a/b}$), 2.05–1.99 (m, 1 H, 3'-H), 1.28 (overlap with major diastereomer, d, $^3J_{(H,H)}$ = 6.5 Hz, 3 H, 6'-H), 1.21–0.99 (overlap with major diastereomer, m, 36 H, Bpin)[24].

^{11}B-NMR (128.4 MHz, toluene-d_8, 26.4 °C): δ [ppm] = 32.7 (br, s, C-Bpin), 21.7 (br, s, N-Bpin and O-Bpin).

[24] This signal overlaps with the signal of HBpin and with Bpin signals of unknown byproducts.

5-Phenyl-1,4-bis(4,4,5,5-tetramethyl-1,3,2-dioxaborolan-2-yl)-1,2,3,4-tetrahydropyridine 3i

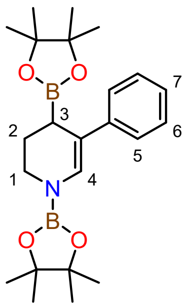

3i
$C_{23}H_{35}B_2NO_4$
411.16 g/mol

The compound was prepared from 3-phenylpyridine **3i** following the general procedure for pyridine double hydroboration described in 3.4 (*Method A* and *B*). The product was characterised from the crude spectra. NMR yield: *Method A* = 46%; *Method B* = 46%.

^{1}H-NMR (400.1 MHz, C_6D_6, 24.9 °C): δ [ppm] = 7.52–7.48 (m, 2 H, 5-H), 7.43 (d, $^4J_{(H,H)}$ = 1.3 Hz, 1 H, 4-H), 7.17–7.10 (m, 2 H, 6-H)[25], 7.03–6.97 (m, 1 H, 7-H)[25], 3.70–3.64 (m, 2 H, 1-H^a and 1-H^b), 2.54 (br, pseudo-t, $^3J_{(H,H)}$ = 4.7 Hz, 1 H, 3-H), 2.07–1.98 (m, 1 H, 2-H$^{a/b}$), 1.96–1.85 (m, 1 H, 2-H$^{a/b}$), 1.07 (br, s, 12 H, Bpin), 0.91 (s, 6 H, Bpin), 0.87 (s, 6 H, Bpin).

^{11}B-NMR (128.4 MHz, C_6D_6, 24.9 °C): δ [ppm] = 33.7 (br, s, C-Bpin), 24.0 (br, s, N-Bpin).

3-Fluoro-1,4-bis(4,4,5,5-tetramethyl-1,3,2-dioxaborolan-2-yl)-1,2,3,4-tetrahydropyridine 3j

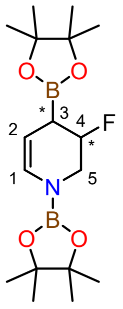

3j
$C_{17}H_{30}B_2FNO_4$
353.05 g/mol

[25] This signal overlaps with a signal of a different compound.

The compound was prepared from 3-fluoropyridine **1j** following the general procedure for pyridine double hydroboration described in 3.4 (*Method A*). The product was characterised from the crude spectra. NMR yield: 23%.

^{1}H-NMR analysis shows the presence of only one single diastereomer. However, an exact assignment of the relative configuration from the crude spectra proves difficult.

^{1}H-NMR (600.1 MHz, C_6D_6, 24.9 °C): δ [ppm] = 6.76 (dd, $^3J_{(H,H)}$ = 8.1 Hz, $^4J_{(H,H)}$ = 2.1 Hz, 1 H, 1-H), 5.17–5.05 (m, $^2J_{(H,F)}$ = 49.0 Hz, 4-H), 4.79 (pseudo-ddt, $^3J_{(H,H)}$ = 8.0 Hz, $^3J_{(H,H)}$/$^4J_{(H,F)}$ = 4.5 Hz, $^4J_{(H,H)}$ = 1.2 Hz, 1 H, 2-H), 3.87 (dddd, $^2J_{(H,H)}$ = 12.7 Hz, $^3J_{(H,F)}$ = 7.4 Hz, $^3J_{(H,H)}$ = 4.9 Hz, $^4J_{(H,H)}$ = 2.3 Hz, 1 H, 5-H$^{a/b}$), 3.60–3.50 (m, 1 H, 5-H$^{a/b}$)[26], 2.32–2.25 (m, $^3J_{(H,F)}$ = 20.3 Hz, 1 H, 3-H), 1.16–0.92 (m, 24 H, Bpin)[27].

^{11}B-NMR (192.6 MHz, C_6D_6, 24.9 °C): δ [ppm] = 32.5 (br, s, C-Bpin), 24.0 (br, s, N-Bpin).

5-Fluoro-1,3-bis(4,4,5,5-tetramethyl-1,3,2-dioxaborolan-2-yl)-1,2,3,6-tetrahydropyridine 3j'

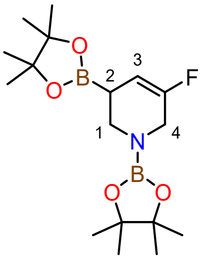

3j'
$C_{17}H_{30}B_2FNO_4$
353.05 g/mol

The compound was prepared from 3-fluoropyridine **1j** following the general procedure for pyridine double hydroboration described in 7.3.1 (Method A). The product was characterised from the crude spectra. NMR yield: 11%.

^{1}H-NMR (600.1 MHz, C_6D_6, 24.9 °C): δ [ppm] = 5.60–5.55 (m, 1 H, 3-H), 3.94–3.89 (m, 1 H, 4-H$^{a/b}$), 3.71–3.62 (overlapping with defluorination product **3c**, m, 2 H, 1-H$^{a/b}$ and 4-H$^{a/b}$), 3.15 (dd, $^2J_{(H,H)}$ = 13.0 Hz, $^3J_{(H,H)}$ = 8.4 Hz, 1 H, 1-H$^{a/b}$), 2.04–1.99 (overlapping with defluorination product **19**, m, 1 H, 2-H), 1.16–0.92 (m, 24 H, Bpin)[28].

^{11}B-NMR (192.6 MHz, C_6D_6, 24.9 °C): δ [ppm] = 32.5 (br, s, C-Bpin), 24.0 (br, s, N-Bpin).

[26] This signal overlaps with a signal of a different compound.
[27] This signal overlaps with the signal of the Bpin groups of other by-products and the signal of HBpin.
[28] This signal overlaps with the signal of the Bpin groups of other byproducts and the signal of HBpin.

3-Methyl-1,4-bis(4,4,5,5-tetramethyl-1,3,2-dioxaborolan-2-yl)-1,2,3,4-tetrahydropyridine 3k

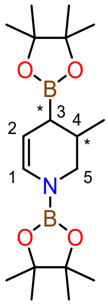

3k
$C_{18}H_{33}B_2NO_4$
349.09 g/mol

The compound was prepared from 3-methylpyridine **1i** following the general procedure for pyridine double hydroboration described in 3.4 (*Method A*). The product was characterised from the crude spectra. NMR yield: 25%.

^{1}H-NMR analysis shows the presence of only one single diastereomer. However, an exact assignment of the relative configuration from the crude spectra proves difficult.

^{1}H-NMR (400.1 MHz, C_6D_6, 26.4 °C): δ [ppm] = 6.80 (dd, $^3J_{(H,H)}$ = 7.9 Hz, $^4J_{(H,H)}$ = 2.6 Hz, 1 H, 1-H), 4.91 (dd, $^3J_{(H,H)}$ = 7.9 Hz, $^3J_{(H,H)}$ = 3.0 Hz, 1 H, 2-H), 3.70 (dd, $^2J_{(H,H)}$ = 11.9 Hz, $^3J_{(H,H)}$ = 3.3 Hz, 1 H, 5-H$^{a/b}$), 2.91 (dd, $^2J_{(H,H)}$ = 11.9 Hz, $^3J_{(H,H)}$ = 8.9 Hz, 1 H, 5-H$^{a/b}$), 2.20–2.08 (m, 1 H, 4-H), 1.66–1.60 (m, 1 H, 3-H)[29], 1.10–0.99 (m, 24 H, Bpin)[30], 1.02–0.99 (m, 3 H, 6-H)[31].

^{11}B-NMR (128.4 MHz, C_6D_6, 26.4 °C): δ [ppm] = 33.7 (br, s, C-Bpin), 24.0 (br, s, N-Bpin).

[29] This signal overlaps with a signal of an unidentified byproduct.
[30] This signal overlaps with the Bpin group of the single hydroborated 1,2-product, HBpin and the signal of the methyl group.
[31] This signal overlaps with the signal of the Bpin groups and the signal of HBpin.

4.2 Characterisation of Borylated *N*-Acetyl Tetrahydropyridines

1-(4-Methyl-3-(4,4,5,5-tetramethyl-1,3,2-dioxaborolan-2-yl)-3,6-dihydropyridin-1(2*H*)-yl)ethan-1-one 4a

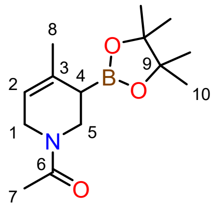

4a
$C_{14}H_{24}BNO_3$
265.16 g/mol

The compound was prepared from 4-methylpyridine **1a** (0.39 mmol) following the general procedure for *N*-acetylation after double hydroboration described in 3.4. **4a** (56 mg, 0.21 mmol, 54%) was obtained as a colourless oil after purification by flash chromatography (SiO_2, EtOAc/pentane = 1:2 → 1:1, (V/V), $KMnO_4$/vanillin, Ø = 1.5 cm x h = 12 cm).

R

$\boldsymbol{R_f}$**-value**: 0.20 (SiO_2, EtOAc/pentane = 1:1, (V/V), $KMnO_4$/vanillin).

Due to limited rotation around the C-N amide bond, the compound forms two stable rotamers in solution at room temperature. This gives rise to two different signal sets in the ^{1}H- and ^{13}C-NMR spectra. One rotamer is energetically preferred over the other. According to ^{1}H-NMR analysis, the ratio between the rotamers amounts to 70:30.

Major rotamer:

^{1}H-NMR (400.1 MHz, $CDCl_3$, 26.3 °C): δ [ppm] = 5.32–5.27 (overlap with minor rotamer, m, 1 H, 2-H), 4.32–4.23 (m, 1 H, 1-$H^{a/b}$), 3.83 (overlap with minor rotamer, dd, $^2J_{(H,H)}$ = 12.7 Hz, $^3J_{(H,H)}$ = 3.0 Hz, 1 H, 5-$H^{a/b}$), 3.60–3.51 (m, 1 H, 1-$H^{a/b}$), 3.24 (dd, $^2J_{(H,H)}$ = 12.7 Hz, $^3J_{(H,H)}$ = 4.2 Hz, 1 H, 5-$H^{a/b}$), 2.14 (s, 3 H, 7-H), 1.80–1.66 (overlap with minor rotamer, m, 4 H, 8-H and 4-H), 1.18 (overlap with minor rotamer, s, 12 H, 10-H).

^{13}C{^{1}H}-NMR (100.6 MHz, $CDCl_3$, 26.3 °C): δ [ppm] = 169.8 (C-6), 133.9 (C-3), 116.7 (C-2), 83.8 (2 C, C-9), 44.8 (C-5), 41.9 (C-1), 28.2 (very weak, C-4)[32], 24.9 (4 C, C-10), 23.5 (C-8), 21.6 (C-7).

Minor rotamer:

^{1}H-NMR (400.1 MHz, $CDCl_3$, 26.3 °C): δ [ppm] = 5.32–5.27 (overlap with major rotamer, m, 1 H, 2'-H), 4.05 (dd, $^2J_{(H,H)}$ = 12.7 Hz, $^3J_{(H,H)}$ = 4.7 Hz, 1 H, 5'-$H^{a/b}$), 3.97–3.87 (m, 1 H, 1'-$H^{a/b}$),

[32] This signal is only visible in the HSQC and HMBC spectrum.

3.84–3.76 (overlap with major rotamer, m, 1 H, 1'-H[a/b]), 3.33 (dd, $^{2}J_{(H,H)}$ = 12.7 Hz, $^{3}J_{(H,H)}$ = 4.9 Hz, 1 H, 5'-H[a/b]), 2.02 (s, 3 H, 7'-H), 1.80–1.66 (overlap with major rotamer, m, 4 H, 8'-H and 4'-H), 1.18 (overlap with major rotamer, s, 12 H, 10'-H).

^{13}C{^{1}H}-NMR (100.6 MHz, CDCl$_3$, 26.3 °C): δ [ppm] = 169.1 (C-6'), 135.8 (C-3'), 115.9 (C-2'), 83.7 (2 C, C-9'), 45.6 (C-1'), 40.0 (C-5'), 27.3 (very weak, C-4')[33], 24.8 (4 C, C-10'), 23.4 (C-8'), 21.8 (C-7').

^{11}B-NMR (128.4 MHz, CDCl$_3$, 26.3 °C): δ [ppm] = 32.6 (br, s).

Additionally, a signal of low intensity at 22.3 ppm can be observed in the ^{11}B-NMR spectrum for residues of a B$_x$pin$_y$ impurity. The presence of such an impurity is supported by a singlet at 1.19 ppm in the ^{1}H-NMR and a signal at 24.7 ppm in the ^{13}C-NMR spectrum.

HRMS-ESI (*m/z*): calc. for $C_{14}H_{25}BNO_3^+$ [M+H]$^+$: 266.1922, found: 266.1922.

1-(4-Phenyl-3-(4,4,5,5-tetramethyl-1,3,2-dioxaborolan-2-yl)-3,6-dihydropyridin-1(2*H*)-yl)ethan-1-one 4b

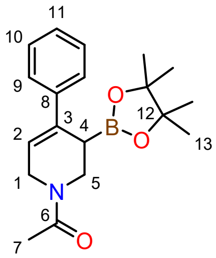

4b

$C_{19}H_{26}BNO_3$

327.23 g/mol

The compound was prepared from 4-phenylpyridine **1b** (0.40 mmol) following the general procedure for *N*-acetylation after double hydroboration described in 3.4.

The product **4b** (30 mg, 92 µmol, 23%) was obtained as a colourless oil after twofold purification by flash chromatography (SiO$_2$, EtOAc/pentane = 1:1 → 3:1, (V/V), KMnO$_4$/vanillin/UV, Ø = 1.5 cm x h = 21 cm/11 cm). The non-borylated tetrahydropyridine byproduct **4b'** elutes just after **4b**.

***R*$_f$-value**: 0.27 (SiO$_2$, EtOAc/pentane = 3:1, (V/V), KMnO$_4$/vanillin/UV).

Due to limited rotation around the C-N amide bond, the compound forms two stable rotamers in solution at room temperature. This gives rise to two different signal sets in the ^{1}H- and ^{13}C-

[33] This signal is only visible in the HSQC and HMBC spectrum.

NMR spectra. One rotamer is energetically preferred over the other. According to ^{1}H-NMR analysis, the ratio between the rotamers amounts to 61:39.

Major rotamer:

^{1}H-NMR (600.1 MHz, $CDCl_3$, 24.9 °C): δ [ppm] = 7.39–7.34 (overlap with minor rotamer, m, 2 H, 9-H), 7.32–7.28 (overlap with minor rotamer, m, 2 H, 10-H), 7.25–7.21 (overlap with minor rotamer, m, 2 H, 11-H), 6.02 (pseudo-t, $^{3}J_{(H,H)}$ = 3.3 Hz, 1 H, 2-H), 4.44–4.39 (m, 1 H, 1-$H^{a/b}$), 4.01–3.93 (m, 2 H, 1-$H^{a/b}$ and 5-$H^{a/b}$), 3.51–3.45 (overlap with minor rotamer, m, 1 H, 5-$H^{a/b}$), 2.49–2.44 (overlap with minor rotamer, m, 1 H, 4-H), 2.20 (s, 3 H, 7-H), 1.14 (s, 6 H, $13^{a/b}$-H), 1.12 (overlap with minor rotamer, s, 6 H, $13^{a/b}$-H).

^{13}C-DEPTQ-NMR (150.9 MHz, $CDCl_3$, 24.9 °C): δ [ppm] = 169.7 (C-6), 141.0 (C-8), 137.5 (C-3), 128.35 (2 C, C-10), 127.2 (C-11), 125.6 (2 C, C-9), 119.5 (C-2), 84.1 (2 C, C-12), 45.4 (C-5), 42.6 (C-1), 26.4 (very weak, C-4)[34], 24.8 (2 C, C-$13^{a/b}$), 24.72 (overlap with minor rotamer, 2 C, C-$13^{a/b}$), 21.8 (C-7).

Minor rotamer:

^{1}H-NMR (600.1 MHz, $CDCl_3$, 24.9 °C): δ [ppm] = 7.39–7.34 (overlap with major rotamer, m, 2 H, 9'-H), 7.32–7.28 (overlap with major rotamer, m, 2 H, 10'-H), 7.25–7.21 (overlap with major rotamer, m, 2 H, 11'-H), 5.98–5.95 (m, 1 H, 2'-H), 4.25 (dd, $^{2}J_{(H,H)}$ = 12.8 Hz, $^{3}J_{(H,H)}$ = 4.7 Hz, 1 H, 5'-$H^{a/b}$), 4.22–4.17 (m, 1 H, 1'-$H^{a/b}$), 4.11–4.06 (m, 1 H, 1'-$H^{a/b}$), 3.51–3.45 (overlap with major rotamer, m, 1 H, 5'-$H^{a/b}$), 2.49–2.44 (overlap with major rotamer, m, 1 H, 4'-H), 2.11 (s, 3 H, 7'-H), 1.11 (overlap with major rotamer, s, 6 H, $13'^{a/b}$-H), 1.08 (s, 6 H, $13'^{a/b}$-H).

^{13}C-DEPTQ -NMR (150.9 MHz, $CDCl_3$, 24.9 °C): δ [ppm] = 169.2 (C-6'), 140.9 (C-8'), 139.1 (C-3'), 128.43 (2 C, C-10'), 127.4 (C-11'), 125.4 (2 C, C-9'), 118.3 (C-2'), 83.8 (2 C, C-12'), 46.0 (C-1'), 40.1 (C-5'), 25.4 (very weak, C-4')[35], 24.71 (overlap with major rotamer, 2 C, C-$13'^{a/b}$), 24.5 (2 C, C-$13'^{a/b}$), 21.9 (C-7).

^{11}B-NMR (192.6 MHz, $CDCl_3$, 24.9 °C): δ [ppm] = 32.9 (br, s).

Additionally, a signal of low intensity at 22.4 ppm can be observed in the ^{11}B-NMR spectrum for residues of a $B_x pin_y$ impurity. The presence of such an impurity is supported by a signal at 1.26 ppm in the ^{1}H-NMR.

HRMS-ESI (*m/z*): calc. for $C_{19}H_{27}BNO_3^+$ $[M+H]^+$: 328.2079, found: 328.2075.

[34] This signal is only visible in the HSQC and HMBC spectrum.
[35] This signal is only visible in the HSQC and HMBC spectrum.

1-(4-Ethyl-3-(4,4,5,5-tetramethyl-1,3,2-dioxaborolan-2-yl)-3,6-dihydropyridin-1(2*H*)-yl)ethan-1-one 4d

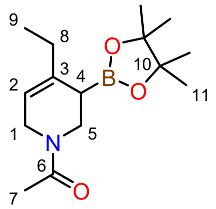

4d
$C_{15}H_{26}BNO_3$
279.19 g/mol

The compound was prepared from 4-ethylpyridine **1d** (0.39 mmol) following the general procedure for *N*-acetylation after double hydroboration described in 3.4.

The product **4d** (61 mg, 0.22 mmol, 56%) was obtained as a colourless oil after twofold purification by flash chromatography (SiO_2, EtOAc/pentane = 1:2 → 1:1, (V/V), $KMnO_4$/vanillin, Ø = 1.5 cm x h = 14 cm/14 cm).

***R*$_f$-value**: 0.24 (SiO_2, EtOAc/pentane = 1:1, (V/V), $KMnO_4$/vanillin).

Due to limited rotation around the C-N amide bond, the compound forms two stable rotamers in solution at room temperature. This gives rise to two different signal sets in the ^{1}H- and ^{13}C-NMR spectra. One rotamer is energetically preferred over the other. According to ^{1}H-NMR analysis, the ratio between the rotamers amounts to 66:34.

Major rotamer:

^{1}H-NMR (600.1 MHz, $CDCl_3$, 24.9 °C): δ [ppm] = 5.30–5.26 (overlap with minor rotamer, m, 1 H, 2-H), 4.30–4.24 (m, 1 H, 1-H$^{a/b}$), 3.85–3.79 (overlap with minor rotamer, m, 1 H, 5-H$^{a/b}$), 3.63–3.56 (m, 1 H, 1-H$^{a/b}$), 3.25–3.18 (overlap with minor rotamer, m, 1 H, 5-H$^{a/b}$), 2.18–2.09 (m, 4 H, 7-H and 8-H$^{a/b}$), 2.07–1.91 (overlap with minor rotamer, m, 1 H, 8-H$^{a/b}$), 1.81–1.76 (br, m, 1 H, 4-H), 1.19–1.15 (overlap with minor rotamer, m, 12 H, 11-H), 1.00–0.95 (overlap with minor rotamer, m, 3 H, 9-H).

^{13}C{^{1}H}-NMR (150.9 MHz, $CDCl_3$, 24.9 °C): δ [ppm] = 169.7 (C-6), 139.4 (C-3), 114.8 (C-2), 83.7 (2 C, C-10), 45.0 (C-5), 42.0 (C-1), 29.3 (C-8), 27.0 (very weak, br, C-4)[36], 24.81 (overlap with minor rotamer, 2 C, C-11$^{a/b}$), 24.79 (2 C, C-11$^{a/b}$), 21.6 (C-7), 11.8 (C-9).

Minor rotamer:

[36] This signal is barely visible even in highly concentrated samples. It is more clearly visible in the HSQC and HMBC spectra.

^{1}H-NMR (600.1 MHz, $CDCl_3$, 24.9 °C): δ [ppm] = 5.30–5.26 (overlap with major rotamer, m, 1 H, 2'-H), 4.16 (dd, $^{2}J_{(H,H)}$ = 12.6 Hz, $^{3}J_{(H,H)}$ = 4.2 Hz, 1 H, 5'-$H^{a/b}$), 3.99–3.92 (m, 1 H, 1'-$H^{a/b}$), 3.85–3.79 (overlap with major rotamer, m, 1 H, 1'-$H^{a/b}$), 3.25–3.18 (overlap with major rotamer, m, 1 H, 5'-$H^{a/b}$), 2.07–1.91 (overlap with major rotamer, m, 5 H, 7'-H and 8'-H), 1.85–1.81 (br, m, 1 H, 4'-H), 1.19–1.15 (overlap with major rotamer, m, 12 H, 11'-H), 1.00–0.95 (overlap with major rotamer, m, 3 H, 9'-H).

^{13}C{^{1}H}-NMR (150.9 MHz, $CDCl_3$, 24.9 °C): δ [ppm] = 169.1 (C-6'), 141.4 (C-3'), 114.1 (C-2'), 83.6 (2 C, C-10'), 45.6 (C-1'), 40.0 (C-5'), 29.9 (C-8'), 26.3 (very weak, br, C-4')[37], 24.81 (overlap with major rotamer, 2 C, C-11'$^{a/b}$), 24.65 (2 C, C-11'$^{a/b}$), 21.8 (C-7'), 12.0 (C-8').

^{11}B-NMR (192.6 MHz, $CDCl_3$, 24.9 °C): δ [ppm] = 32.5 (br, s).

Additionally, a signal of low intensity at 22.3 ppm can be observed in the ^{11}B-NMR spectrum for residues of a $B_x pin_y$ impurity. The presence of such an impurity is supported by a singlet at 1.20 ppm in the ^{1}H-NMR and a signal at 24.67 ppm in the ^{13}C-NMR spectrum.

HRMS-ESI (*m/z*): calc. for $C_{15}H_{27}BNO_3^+$ $[M+H]^+$: 280.2079, found: 280.2083.

1-(4-Benzyl-3-(4,4,5,5-tetramethyl-1,3,2-dioxaborolan-2-yl)-3,6-dihydropyridin-1(2*H*)-yl)ethan-1-one 4e

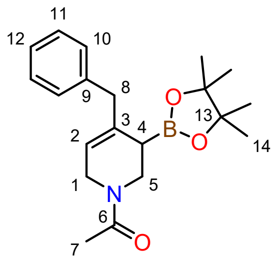

4e
$C_{20}H_{28}BNO_3$
341.26 g/mol

The compound was prepared from 4-benzylpyridine **1e** (0.40 mmol) following the general procedure for *N*-acetylation after double hydroboration described in 3.4.

The product **4e** (99 mg, 0.29 mmol, 73%) was obtained as a colourless oil after twofold purification by flash chromatography (SiO_2, EtOAc/pentane = 1:2 → 1:1, (V/V), $KMnO_4$/vanillin/UV, Ø = 1.5 cm x h = 15 cm/10 cm).

***R*$_f$-value**: 0.24 (SiO_2, EtOAc/pentane = 1:1, (V/V), $KMnO_4$/vanillin/UV).

[37] This signal is barely visible even in highly concentrated samples. It is more clearly visible in the HSQC and HMBC spectra.

Due to limited rotation around the C-N amide bond, the compound forms two stable rotamers in solution at room temperature. This gives rise to two different signal sets in the ^{1}H- and ^{13}C-NMR spectra. One rotamer is energetically preferred over the other. According to ^{1}H-NMR analysis, the ratio between the rotamers amounts to 73:27.

Major rotamer:

^{1}H-NMR (400.1 MHz, $CDCl_3$, 26.4 °C): δ [ppm] = 7.31–7.24 (overlap with minor rotamer, m, 2 H, 11-H), 7.22–7.13 (overlap with minor rotamer, m, 3 H, 10-H and 12-H), 5.35–5.31 (m, 1 H, 2-H), 4.45–4.36 (m, 1 H, 1-$H^{a/b}$), 3.88 (overlap with minor rotamer, dd, $^{2}J_{(H,H)}$ = 12.7 Hz, $^{3}J_{(H,H)}$ = 2.4 Hz, 1 H, 5-$H^{a/b}$), 3.62–3.53 (m, 1 H, 1-$H^{a/b}$), 3.53–3.45 (m, 1 H, 8-$H^{a/b}$), 3.43–3.35 (overlap with minor rotamer, m, 1 H, 8-$H^{a/b}$), 3.14 (overlap with minor rotamer, dd, $^{2}J_{(H,H)}$ = 12.7 Hz, $^{3}J_{(H,H)}$ = 4.1 Hz, 1 H, 5-$H^{a/b}$), 2.16 (s, 3 H, 7-H), 1.77–1.71 (br, m, 1 H, 4-H), 1.22 (s, 12 H, 14-H).

^{13}C{^{1}H}-NMR (100.6 MHz, $CDCl_3$, 26.4 °C): δ [ppm] = 169.9 (C-6), 139.5 (C-9), 137.6 (C-3), 129.3 (2 C, C-10), 128.4 (2 C, C-11), 126.2 (C-12), 118.1 (C-2), 83.8 (2 C, C-13), 45.1 (C-5), 43.1 (C-8), 42.1 (C-1), 26.3 (overlap with minor rotamer, very weak, br, C-4)[38], 25.0 (2 C, C-$14^{a/b}$), 24.8 (2 C, C-$14^{a/b}$), 21.7 (C-7).

Minor rotamer:

^{1}H-NMR (400.1 MHz, $CDCl_3$, 26.4 °C): δ [ppm] = 7.31–7.24 (overlap with major rotamer, m, 2 H, 11'-H), 7.22–7.13 (overlap with major rotamer, m, 3 H, 10'-H and 12'-H), 5.28–5.24 (m, 1 H, 2'-H), 4.32 (dd, $^{2}J_{(H,H)}$ = 12.6 Hz, $^{3}J_{(H,H)}$ = 3.5 Hz, 1 H, 5'-$H^{a/b}$), 4.04–3.95 (m, 1 H, 1'-$H^{a/b}$), 3.88–3.80 (overlap with major rotamer, m, 1 H, 1'-$H^{a/b}$), 3.43–3.35 (overlap with major rotamer, m, 1 H, 8'-$H^{a/b}$), 3.32–3.25 (m, 1 H, 8'-$H^{a/b}$), 3.09 (overlap with major rotamer, dd, $^{2}J_{(H,H)}$ = 12.6 Hz, $^{3}J_{(H,H)}$ = 4.6 Hz, 1 H, 5'-$H^{a/b}$), 2.04 (s, 3 H, 7'-H), 1.86–1.81 (br, m, 1 H, 4'-H), 1.19 (s, 6 H, $14'^{a/b}$-H), 1.18 (s, 6 H, $14'^{a/b}$-H).

^{13}C{^{1}H}-NMR (100.6 MHz, $CDCl_3$, 26.4 °C): δ [ppm] = 169.2 (C-6'), 139.7 (C-3'), 139.0 (C-9'), 129.4 (2 C, C-10'), 128.5 (2 C, C-11'), 126.3 (C-12'), 117.0 (C-2'), 83.8 (2 C, C-13'), 45.6 (C-1'), 43.6 (C-8'), 40.2 (C-5'), 26.3 (overlap with major rotamer, very weak, br, C-4')[39], 24.9 (2 C, C-$14'^{a/b}$), 24.7 (2 C, C-$14'^{a/b}$), 21.9 (C-7').

^{11}B-NMR (128.4 MHz, $CDCl_3$, 26.4 °C): δ [ppm] = 32.1 (br, s).

[38] This signal is barely visible even in highly concentrated samples. It is more clearly visible in the HSQC and HMBC spectra.
[39] This signal is barely visible even in highly concentrated samples. It is more clearly visible in the HSQC and HMBC spectra.

Additionally, a signal of low intensity at 22.3 ppm can be observed in the ^{11}B-NMR spectrum for residues of a B_xpin_y impurity. The presence of such an impurity is supported by a weak singlet at 1.24 ppm in the ^{1}H-NMR.

HRMS-ESI (*m/z*): calc. for $C_{20}H_{29}BNO_3^+$ $[M+H]^+$: 342.2235, found: 342.2236.

1-(3-(4,4,5,5-Tetramethyl-1,3,2-dioxaborolan-2-yl)-4-(trifluoromethyl)-3,6-dihydro-pyridin-1(2*H*)-yl)ethan-1-one 4f

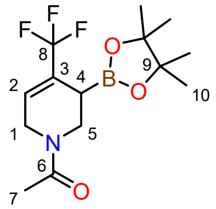

4f
$C_{14}H_{21}BF_3NO_3$
319.13 g/mol

The compound was prepared from 4-(trifluoromethyl)pyridine **1f** (0.39 mmol) following the general procedure for *N*-acetylation after double hydroboration described in 3.4.

The product **4f** (43 mg, 0.14 mmol, 34%) was obtained as a colourless oil after purification by flash chromatography (SiO_2, EtOAc/pentane = 1:2 → 1:1, (V/V), $KMnO_4$/vanillin, Ø = 1.5 cm x h = 19 cm). The non-borylated tetrahydropyridine byproduct elutes just after **4f**.

***R*$_f$-value**: 0.16 (SiO_2, EtOAc/pentane = 1:1, (V/V), $KMnO_4$/vanillin).

Due to limited rotation around the C-N amide bond, the compound forms two stable rotamers in solution at room temperature. This gives rise to two different signal sets in the ^{1}H- and ^{13}C-NMR spectra. One rotamer is energetically preferred over the other. According to ^{1}H-NMR analysis, the ratio between the rotamers amounts to 59:41.

Major rotamer:

^{1}H-NMR (600.1 MHz, $CDCl_3$, 24.9 °C): δ [ppm] = 6.30–6.26 (m, 1 H, 2-H), 4.57–4.50 (m, 1 H, 1-$H^{a/b}$), 4.01–3.93 (overlap with minor rotamer, m, 1 H, 5-$H^{a/b}$), 3.75–3.68 (m, 1 H, 1-$H^{a/b}$), 3.28 (dd, $^2J_{(H,H)}$ = 12.9 Hz, $^3J_{(H,H)}$ = 4.0 Hz, 1 H, 5-$H^{a/b}$), 2.16–2.11 (m, 4 H, 7-H and 4-H), 1.21–1.17 (overlap with minor rotamer, m, 12 H, 10-H).

$^{13}C\{^1H\}$-NMR (125.8 MHz, $CDCl_3$, 19.8 °C): δ [ppm] = 169.9 (C-6), 128.9 (q, $^2J_{(C,F)}$ = 31.7 Hz, C-3), 125.8 (q, $^3J_{(C,F)}$ = 5.8 Hz, C-2), 123.22 (overlap with C-2', q, $^1J_{(C,F)}$ = 272.0 Hz, C-8), 84.4 (2 C, C-9), 44.6 (C-5), 41.4 (C-1), 24.8 (4 C, C-10), 21.7 (C-7).

^{19}F-NMR (564.6 MHz, $CDCl_3$, 24.9 °C): δ [ppm] = –68.3 (s, 8-F).

Minor rotamer:

^1H-NMR (600.1 MHz, $CDCl_3$, 24.9 °C): δ [ppm] = 6.22–6.19 (m, 1 H, 2'-H), 4.62 (dd, $^2J_{(H,H)}$ = 12.9 Hz, $^3J_{(H,H)}$ = 2.2 Hz, 1 H, 5'-$H^{a/b}$), 4.23–4.16 (m, 1 H, 1'-$H^{a/b}$), 4.01–3.93 (overlap with major rotamer, m, 1 H, 1'-$H^{a/b}$), 2.95 (dd, $^2J_{(H,H)}$ = 12.9 Hz, $^3J_{(H,H)}$ = 4.3 Hz, 1 H, 5'-$H^{a/b}$), 2.10–2.06 (m, 4 H, 7'-H and 4'-H), 1.21–1.17 (overlap with major rotamer, m, 12 H, 10-H).

$^{13}C\{^1H\}$-NMR (125.8 MHz, $CDCl_3$, 19.8 °C): δ [ppm] = 169.2 (C-6'), 130.8 (q, $^2J_{(C,F)}$ = 31.1 Hz, C-3'), 124.2 (overlap with C-8 and C-8', q, $^3J_{(C,F)}$ = 5.9 Hz, C-2'), 123.25 (overlap with C-2', q, $^1J_{(C,F)}$ = 271.7 Hz, C-8'), 84.3 (2 C, C-9'), 44.8 (C-1'), 39.2 (C-5'), 24.5 (4 C, C-10'), 21.8 (C-7').

^{19}F-NMR (564.6 MHz, $CDCl_3$, 24.9 °C): δ [ppm] = –69.0 (s, 8'-F)

^{11}B-NMR (192.6 MHz, $CDCl_3$, 24.9 °C): δ [ppm] = 31.9 (br, s).

Additionally, a signal of low intensity at 22.3 ppm can be observed in the ^{11}B-NMR spectrum for residues of a B_xpin_y impurity. The presence of such an impurity is supported by a singlet at 1.23 ppm in the ^1H-NMR and a signal at 24.7 ppm in the ^{13}C-NMR spectrum.

HRMS-ESI (*m/z*): calc. for $C_{14}H_{22}BF_3NO_3^+$ $[M+H]^+$: 320.1639, found: 320.1636.

1-(3,4-Bis(4,4,5,5-tetramethyl-1,3,2-dioxaborolan-2-yl)-3,6-dihydropyridin-1(2*H*)-yl)ethan-1-one 4g

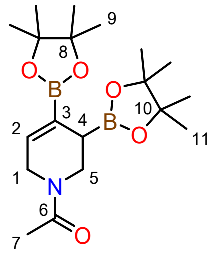

4g
$C_{19}H_{33}B_2NO_5$
377.10 g/mol

The compound was prepared from 4-(4,4,5,5-tetramethyl-1,3,2-dioxaborolan-2-yl)pyridine **1g** (0.39 mmol) following the general procedure for *N*-acetylation after double hydroboration described in 3.4.

The product **4g** (46 mg, 0.12 mmol, 31%) was obtained as a colourless oil after twofold purification by flash chromatography (SiO_2, EtOAc/pentane = 1:1 → 3:1, (V/V), $KMnO_4$/vanillin, Ø = 1.5 cm x h = 14 cm/10 cm). The mono-borylated tetrahydropyridine byproduct **4g'** elutes just after **4g**.

***R*f-value**: 0.26 (SiO_2, EtOAc/pentane = 3:1, (V/V), $KMnO_4$/vanillin).

Due to limited rotation around the C-N amide bond, the compound forms two stable rotamers in solution at room temperature. This gives rise to two different signal sets in the ^{1}H- and ^{13}C-NMR spectra. One rotamer is energetically preferred over the other. According to ^{1}H-NMR analysis, the ratio between the rotamers amounts to 76:24.

Major rotamer:

^{1}H-NMR (400.1 MHz, $CDCl_3$, 26.4 °C): δ [ppm] = 6.39–6.34 (overlap with minor rotamer, m, 1 H, 2-H), 4.44–4.35 (m, 1 H, 1-$H^{a/b}$), 3.84 (dd, $^2J_{(H,H)}$ = 12.8 Hz, $^3J_{(H,H)}$ = 3.0 Hz, 1 H, 5-$H^{a/b}$), 3.67 (pseudo-dt, $^2J_{(H,H)}$ = 20.1 Hz, $^{3/5}J_{(H,H)}$ = 2.7 Hz, 1 H, 1-$H^{a/b}$), 3.22 (overlap with minor rotamer, dd, $^2J_{(H,H)}$ = 12.8 Hz, $^3J_{(H,H)}$ = 4.4 Hz, 1 H, 5-$H^{a/b}$), 2.14 (s, 3 H, 7-H), 2.11–2.05 (overlap with minor rotamer, br, m, 1 H, 4-H), 1.25–1.20 (overlap with minor rotamer, m, 12 H, 9/11-H), 1.18–1.15 (overlap with minor rotamer, m, 12 H, 9/11-H).

^{13}C{^{1}H}-NMR (100.6 MHz, $CDCl_3$, 26.4 °C): δ [ppm] = 169.9 (C-6), 135.7 (C-2), 129.5 (very weak, C-3)[40], 83.7 (2 C, C-8/10), 83.5 (2 C, C-8/10), 44.5 (C-5), 43.1 (C-1), 25.1 (2 C, C-$9^{a/b}$/C-$11^{a/b}$), 25.0 (2 C, C-$9^{a/b}$/C-$11^{a/b}$), 24.9 (very weak, C-4), 24.77 (overlap with minor rotamer, 2 C, C-$9^{a/b}$/C-$11^{a/b}$), 24.5 (2 C, C-$9^{a/b}$/C-$11^{a/b}$), 21.6 (C-7).

Minor rotamer:

^{1}H-NMR (400.1 MHz, $CDCl_3$, 26.4 °C): δ [ppm] = 6.39–6.34 (overlap with major rotamer, m, 1 H, 2'-H), 4.17 (dd, $^2J_{(H,H)}$ = 12.7 Hz, $^3J_{(H,H)}$ = 4.0 Hz, 1 H, 5'-$H^{a/b}$), 4.06–3.98 (m, 1 H, 1'-$H^{a/b}$), 3.91 (pseudo-dt, $^2J_{(H,H)}$ = 18.6 Hz, $^{3/5}J_{(H,H)}$ = 2.9 Hz, 1 H, 1'-$H^{a/b}$), 3.21–3.15 (overlap with major rotamer, m, 1 H, 5'-$H^{a/b}$), 2.11–2.05 (overlap with major rotamer, br, m, 1 H, 4'-H), 2.02 (s, 3 H, 7'-H), 1.25–1.20 (overlap with major rotamer, m, 12 H, 9'/11'-H), 1.18–1.15 (overlap with major rotamer, m, 12 H, 9'/11'-H).

^{13}C{^{1}H}-NMR (100.6 MHz, $CDCl_3$, 26.4 °C): δ [ppm] = 169.0 (C-6'), 134.8 (C-2'), 131.3 (very weak, C-3')[41], 83.6 (2 C, C-8'/10'), 83.4 (2 C, C-8'/10'), 46.7 (C-1'), 39.6 (C-5'), 24.9 (2 C, C-$9'^{a/b}$/C-$11'^{a/b}$), 24.8 (2 C, C-$9'^{a/b}$/C-$11'^{a/b}$), 24.72 (2 C, C-$9'^{a/b}$/C-$11'^{a/b}$), 24.7 (2 C, C-$9'^{a/b}$/C-$11'^{a/b}$), 23.5 (very weak, C-4')[42], 21.9 (C-7').

[40] This signal is only visible in the HMBC spectrum.
[41] This signal is only visible in the HMBC spectrum.

^{11}B-NMR (128.4 MHz, $CDCl_3$, 26.4 °C): δ [ppm] = 32.2 (overlapping, br, s, 4-B), 29.4 (overlapping, br, s, 3-B).

Additionally, a signal of low intensity at 22.2 ppm can be observed in the ^{11}B-NMR spectrum for residues of a $B_x$pin$_y$ impurity. The presence of such an impurity is supported by a singlet at 1.19 ppm in the ^{1}H-NMR and a signal at 24.8 ppm (overlap with product signals) in the ^{13}C-NMR spectrum.

HRMS-ESI (*m/z*): calc. for $C_{19}H_{34}B_2NO_5^+$ $[M+H]^+$: 378.2618, found: 378.2616.

1-(5-Phenyl-4-(4,4,5,5-tetramethyl-1,3,2-dioxaborolan-2-yl)-3,4-dihydropyridin-1(2*H*)-yl)ethan-1-one 4i

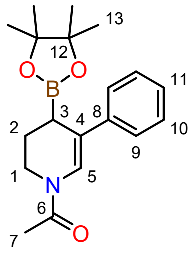

4i
$C_{19}H_{26}BNO_3$
327.23 g/mol

The compound was prepared from 3-phenylpyridine **1i** (0.40 mmol) following the general procedure for *N*-acetylation after double hydroboration described in 3.4.

The product **4i** (26 mg, 80 µmol, 20%) was obtained as a slightly yellow oil after twofold purification by flash chromatography (SiO_2, CH_2Cl_2 +1% MeOH, (V/V), $KMnO_4$/vanillin, Ø = 1.5 cm x h = 12 cm/16 cm). Non-borylated tetrahydropyridine and isomeric byproducts elute just before and after **4i**.

***R*$_f$-value**: 0.17 (SiO_2, CH_2Cl_2 +1% MeOH, (V/V), $KMnO_4$/vanillin/UV).

Due to limited rotation around the C-N amide bond, the compound forms two stable rotamers in solution at room temperature. This gives rise to two different signal sets in the ^{1}H- and ^{13}C-NMR spectra. One rotamer is energetically preferred over the other. According to ^{1}H-NMR analysis, the ratio between the rotamers amounts to 75:25.

Major rotamer:

---

[42] This signal is only visible in the HSQC and HMBC spectra.

^{1}H-NMR (600.1 MHz, $CDCl_3$, 24.8 °C): δ [ppm] = 7.37–7.35 (m, 2 H, 9-H), 7.32–7.28 (overlap with minor rotamer, m, 2 H, 10-H), 7.22–7.19 (m, 1 H, 11-H), 6.98 (d, $^{4}J_{(H,H)}$ = 1.1 Hz, 1 H, 5-H), 3.97 (pseudo-dt, $^{2}J_{(H,H)}$ = 13.1 Hz, $^{3}J_{(H,H)}$ = 4.7 Hz, , $^{3}J_{(H,H)}$ = 4.7 Hz, 1 H, 1-$H^{a/b}$), 3.56 (ddd, $^{2}J_{(H,H)}$ = 13.1 Hz, $^{3}J_{(H,H)}$ = 9.8 Hz, , $^{3}J_{(H,H)}$ = 4.0 Hz, 1 H, 1-$H^{a/b}$), 2.45–2.42 (overlap with minor rotamer, m, 1 H, 3-H), 2.26 (s, 3 H, 7-H), 2.04–1.95 (overlap with minor rotamer, m, 2 H, 2-H^{a} and 2-H^{b}), 1.13 (overlap with minor rotamer, s, 6 H, $13^{a/b}$-H), 1.09 (overlap with minor rotamer, s, 6 H, $13^{a/b}$-H).

^{13}C{^{1}H}-NMR (150.9 MHz, $CDCl_3$, 24.8 °C): δ [ppm] = 168.5 (C-6), 140.6 (C-8), 128.5 (2 C, C-10), 126.7 (C-11), 125.36 (2 C, C-9), 122.4 (C-5), 121.0 (C-4), 83.8 (2 C, C-12), 39.5 (C-1), 24.7 (2 C, C-$13^{a/b}$), 24.6 (2 C, C-$13^{a/b}$), 23.4 (C-2), 21.8 (overlap with minor rotamer, 2 C, C-3[43] and C-7).

Minor rotamer:

^{1}H-NMR (600.1 MHz, $CDCl_3$, 24.8 °C): δ [ppm] = 7.73 (d, $^{4}J_{(H,H)}$ = 1.2 Hz, 1 H, 5'-H), 7.44–7.40 (m, 2 H, 9'-H), 7.31–7.27 (overlap with major rotamer, m, 2 H, 10'-H), 7.19–7.15 (m, 1 H, 11'-H), 3.72–3.63 (m, 2 H, 1'-H^{a} and 1'-H^{b}), 2.45–2.42 (overlap with major rotamer, m, 1 H, 3'-H), 2.20 (s, 3 H, 7'-H), 2.09–2.04 (overlap with major rotamer, m, 2 H, 2'-H^{a} and 2'-H^{b}), 1.13 (overlap with major rotamer, s, 6 H, 13'-$H^{a/b}$), 1.09 (overlap with major rotamer, s, 6 H, 13'-$H^{a/b}$).

^{13}C{^{1}H}-NMR (150.9 MHz, $CDCl_3$, 24.8 °C): δ [ppm] = 168.0 (C-6'), 140.4 (C-8'), 128.3 (2 C, C-10'), 126.4 (C-11'), 125.42 (2 C, C-9'), 120.8 (C-4'), 120.6 (C-5'), 83.7 (2 C, C-12'), 43.4 (C-1'), 24.62 (2 C, C-13'$^{a/b}$), 24.60 (2 C, C-13'$^{a/b}$), 24.1 (C-2'), 22.4 (C-7'), 21.8 (very weak, overlap with major rotamer, C-3')[44].

^{11}B-NMR (192.6 MHz, $CDCl_3$, 24.8 °C): δ [ppm] = 32.8 (br, s).

Additionally, a signal of low intensity at 22.5 ppm can be observed in the ^{11}B-NMR spectrum for residues of a $B_x pin_y$ impurity. The presence of such an impurity is supported by a singlet at 1.27 ppm in the ^{1}H-NMR. The compound is slightly contaminated with unidentified isomers or a non-borylated tetrahydropyridine product as observed by additional signals in the ^{1}H- and ^{13}C-NMR spectra.

HRMS-ESI (*m/z*): calc. for $C_{19}H_{27}BNO_3^+$ $[M+H]^+$: 328.2079, found: 328.2071.

[43] This signal is only visible in the HSQC and HMBC spectrum.
[44] This signal is only visible in the HSQC and HMBC spectrum.

5. Unsuccessful substrates

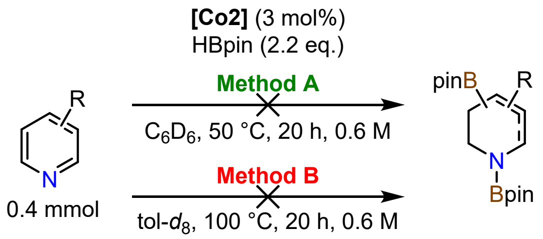

Unsuccessful substrates

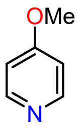

Method A: 1,2-Hydroboration (48%)
Method B: 1,2-Hydroboration (18%)

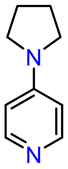

Method A: n.r.
Method B: 1,2-Hydroboration (5%)

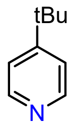

Method B: 1,2-Hydroboration (71%), 1,4-hydroboration (18%)

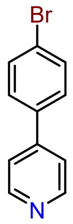

Method A: Precipitation of a turquoise solid

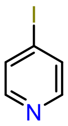

Method A: Precipitation of a purple solid

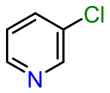

Method A: Precipitation of a turquoise solid

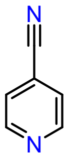

Method A: Nitrile hydroboration (13%), complex reaction mixture
Method B[a]: Complex reaction mixture

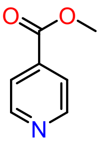

Method A and **Method B**[a]**:** Dearomatisation and ester hydroboration

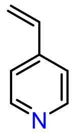

Method A: Polymerisation

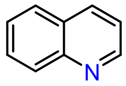

Method A: 1,2-Hydroboration (50%), 1,4-hydroboration (17%)
Method B: paramagnetic reaction mixture

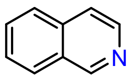

Method A: 1,2-Hydroboration (96%)

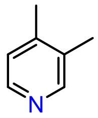

Method A: 1,2-Hydroboration (74%), 1,6-hydroboration (22%)

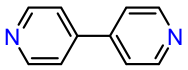

Method A: Precipitation of a colourless solid

Scheme S4: Unsuccessful substrates tested in the double hydroboration of pyridines. Conditions: Substrate (0.40 mmol, 1.0 eq.), HBpin (0.88 mmol, 2.2 eq.), **Co2** (3 mol%) in C_6D_6 at 50 °C (*Method A*) or in tol-d_8 at 100 °C (*Method B*) at 0.6 M for 20 h inside a headspace vial (4 mL) with magnetic stirring unless otherwise specified. *a*) 4.4 eq. of HBpin used. n.r. = No reaction. Yield determined by ^{1}H-NMR using hexamethylbenzene (C_6D_6) or trimethoxybenzene (tol-d_8) as an internal standard.

Single hydroboration products (quinoline[15,16], isoquinoline[15,16], 3,4-lutidine[17,18], 4-methoxypyridine[17,19,20], 4-*tert*-butylpyridine[20]) were identified by ^{1}H-NMR spectroscopy by matching the NMR data with the values reported in literature (also see Figure 105–114).

6. Rotamer Formation and Long-Term Stability of *N*-Acetyl Allylic Boronates

6.1 Temperature Gradient NMR spectroscopy

^{1}H- and ^{13}C-NMR spectra of **4a** in toluene-d_8 were measured in steps of 10 °C between 27 °C and 100 °C. After heating of the sample, another ^{1}H-NMR spectrum was measured at room temperature to test for decomposition.

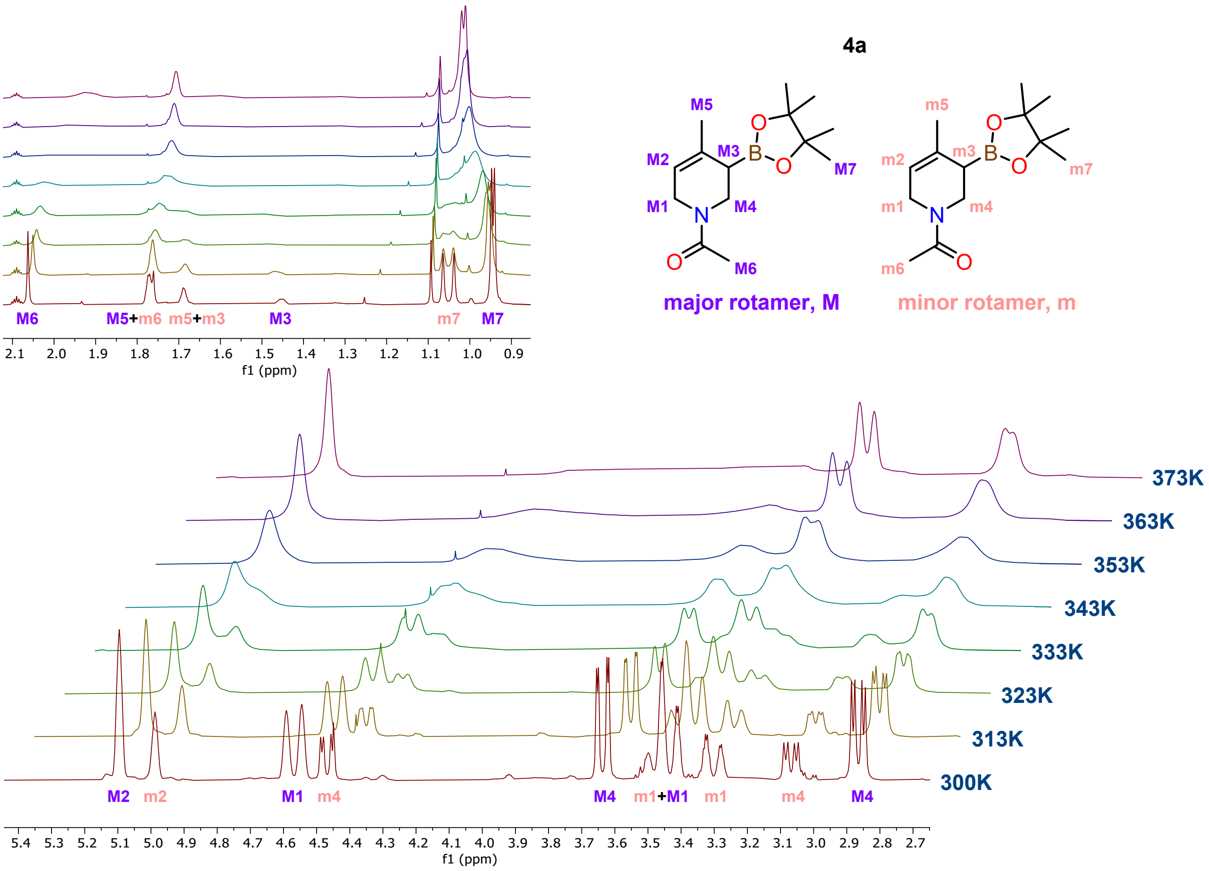

Scheme S5: Temperature gradient ^{1}H-NMR of **4a** in toluene-d_8 between 27 °C and 100 °C.

With the help of a modified version of the Eyring equation (eq. 1), the energy barrier for rotation around the C–N amide bond can be determined.[21]

$$\Delta G^{\ddagger} = RT_C\left(22.96 + ln\left(\frac{T_C}{\Delta v}\right)\right) \qquad (1)$$

In equation (1) R is the gas constant (8.314 J/mol·K), T_C is the coalescence temperature (in K) and Δv is the difference in chemical shift (δ_M–δ_m) in the low-temperature spectra (in Hz). Considering the signal of the vinylic proton (M2–m2, Δv = 43.1 Hz, T_C = 353 K) the rotational barrier was determined to be 73.6 kJ/mol.

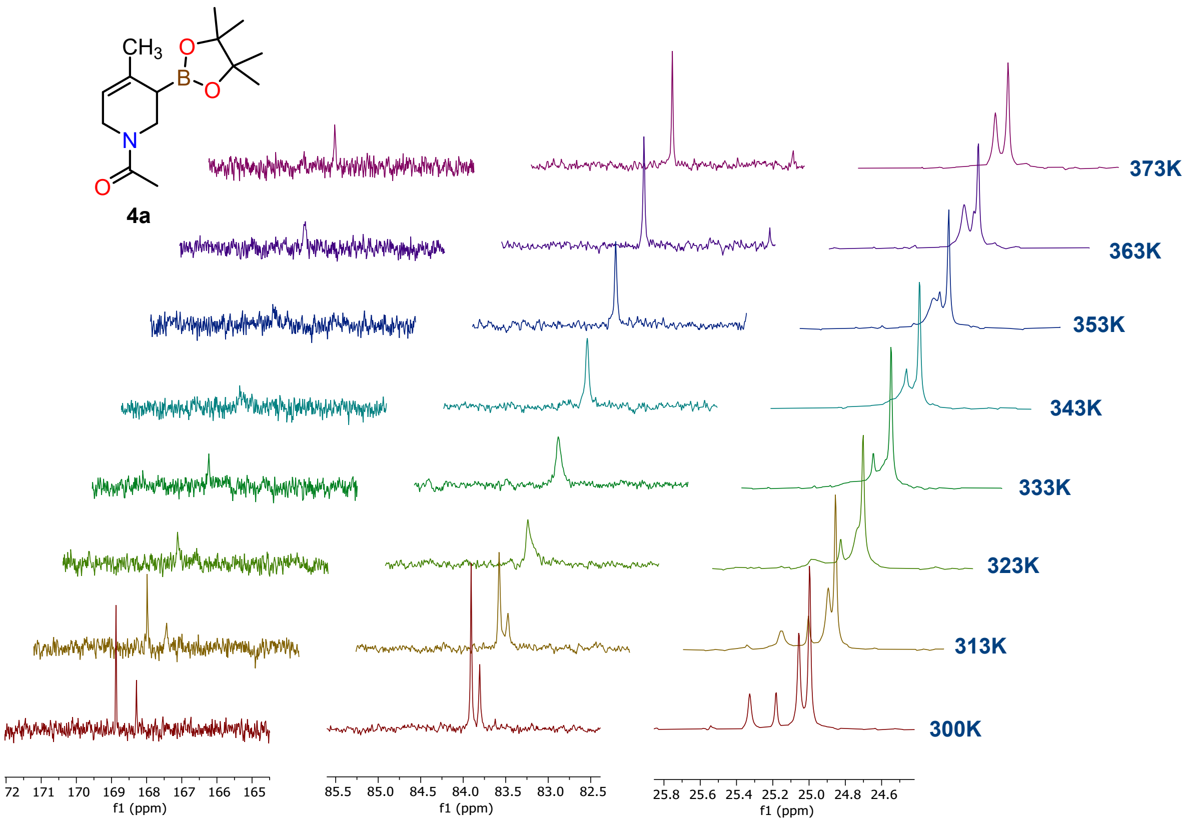

Scheme S6: Temperature gradient $^{13}C\{^{1}H\}$-NMR analysis of **4a** between 27 °C (bottom, maroon) and 100 °C (top, light purple) in steps of 10 °C (from bottom to top) in toluene-d_8.

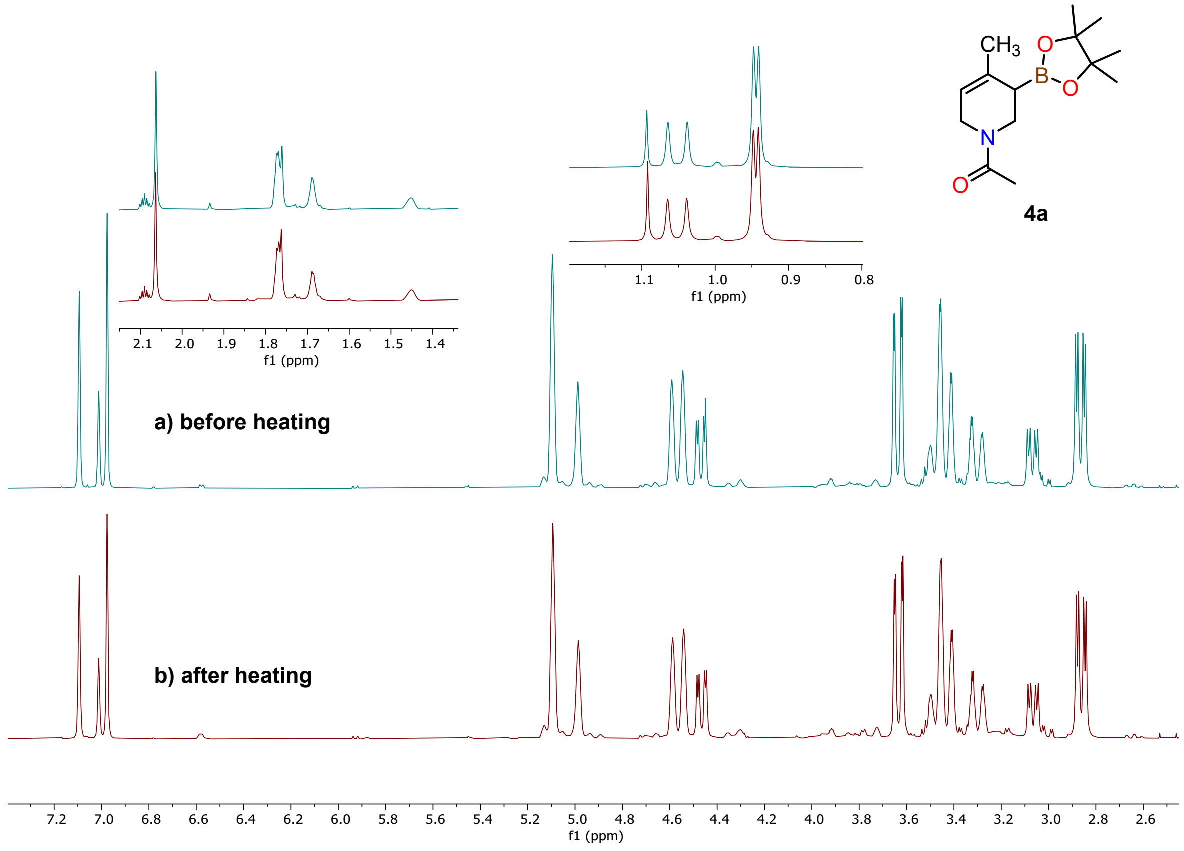

Scheme S7: Overlay of ^{1}H-NMR spectra of **4a** before (a, turquoise) and after (b, maroon) heating to 100 °C over 6 h in toluene-d_8.

6.2 Long-Term Stability of *N*-Acetyl Allylic Boronates

To test for long-term stability of the *N*-acetyl allylic boronates, NMR spectra of the compounds were measured immediately after isolation. The NMR samples were stored at –30 °C for two to three months and measured once again (Scheme S8).

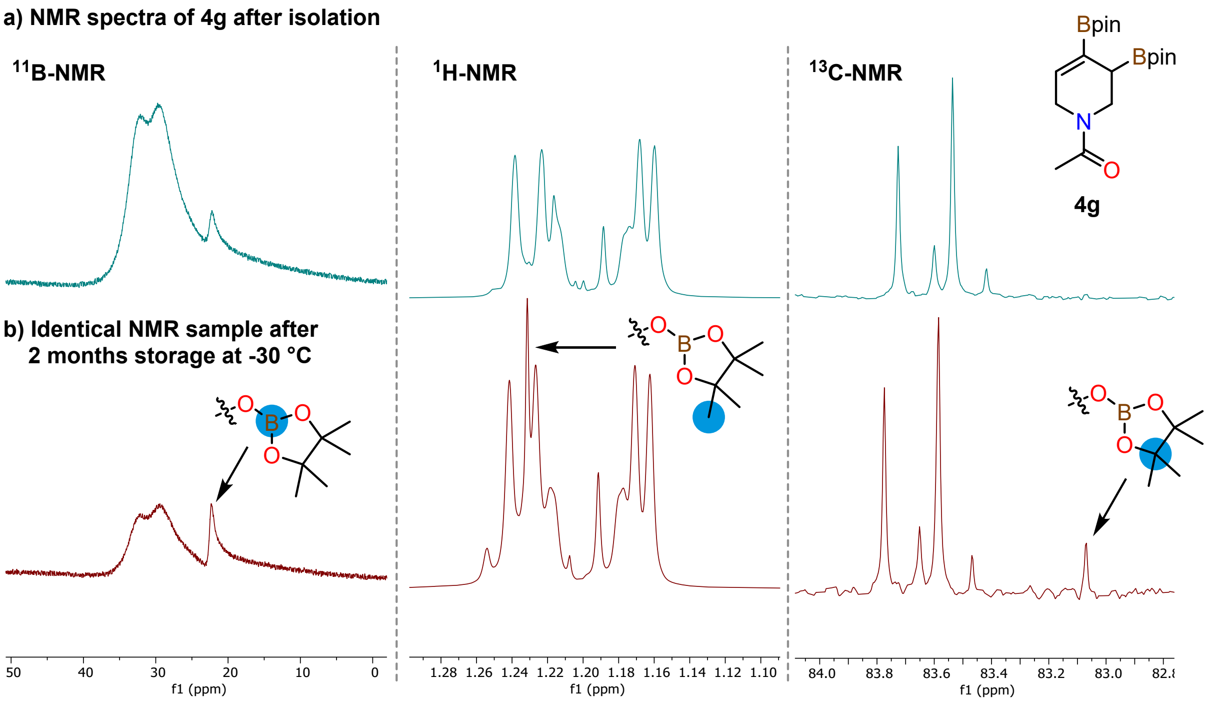

Scheme S8: Investigation of long-term stability of cyclic *N*-acetyl allylic boronates examplified by NMR analysis of **4g**. a) NMR spectra in $CDCl_3$ measured directly after isolation. b) NMR spectra of the identical sample after 2-month storage at –30 °C in solution.

Additionally, compound integrity of **4a** was examined by NMR spectroscopy after storing the oily compound for four months at –30 °C on air (Scheme S9).

The increase in B_xpin_y impurity upon long-term storage is most likely the cause of slow protodeboronation of the allylic boronate esters.[22] Hence, the allylboron species were found to be bench-stable enough for handling on air. Long-term storage over inert atmosphere at low temperatures should however be considered.

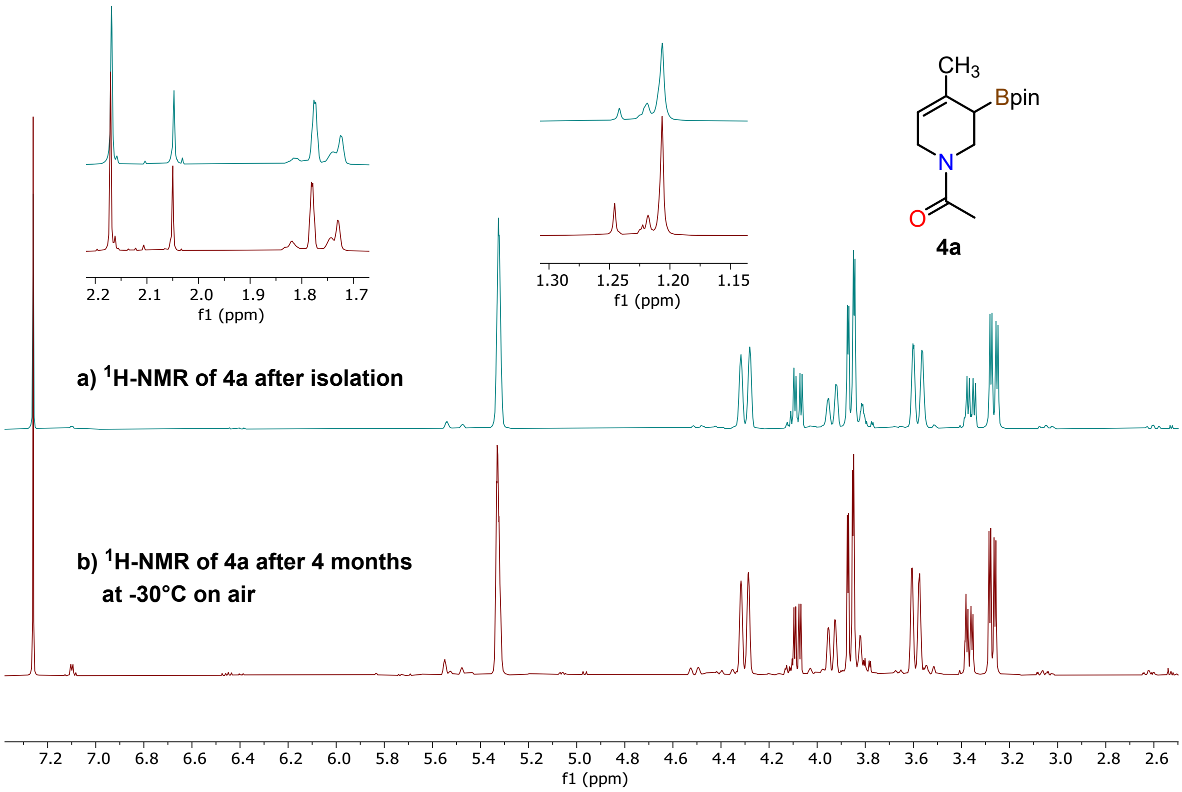

Scheme S9: Overlay of ^{1}H-NMR spectra of **4a** immediately after isolation (a, turquoise) and after 4 months storage at –30 °C on air (b, maroon) in $CDCl_3$.

7. Mechanistic Studies

7.1 NMR kinetics

Inside a glovebox filled with argon, a J. Young NMR tube was charged with precatalyst (**Co2**/**Co6**, 3 mol%, 5.0/6.4 mg), **1a** (0.4 mmol, 39 µL), hexamethylbenzene (0.02 mmol, 50 µL of a 0.4 M stock solution in C_6D_6), and C_6D_6 (0.5 mL). The resulting solution was cooled to –35°C for 16 h. Then, pinacolborane (0.88 mmol, 128 µl) was added on top of the frozen solution. The thawing reaction mixture was shaken for 5 s and the NMR tube was quickly placed into a pre-heated NMR device at 50 °C. After 5 min (shimming and locking), the hydroboration reaction was monitored by *in situ* ^{1}H- and ^{11}B-NMR (see Scheme S10–S14).

Conversion of **1a** and yields of **2a**/**3a** were determined by integrating the signals at 8.43 ppm (**1a**), 1.58 ppm (**2a**) and 5.28 ppm (**3a**) relative to the internal standard (hexamethylbenzene).

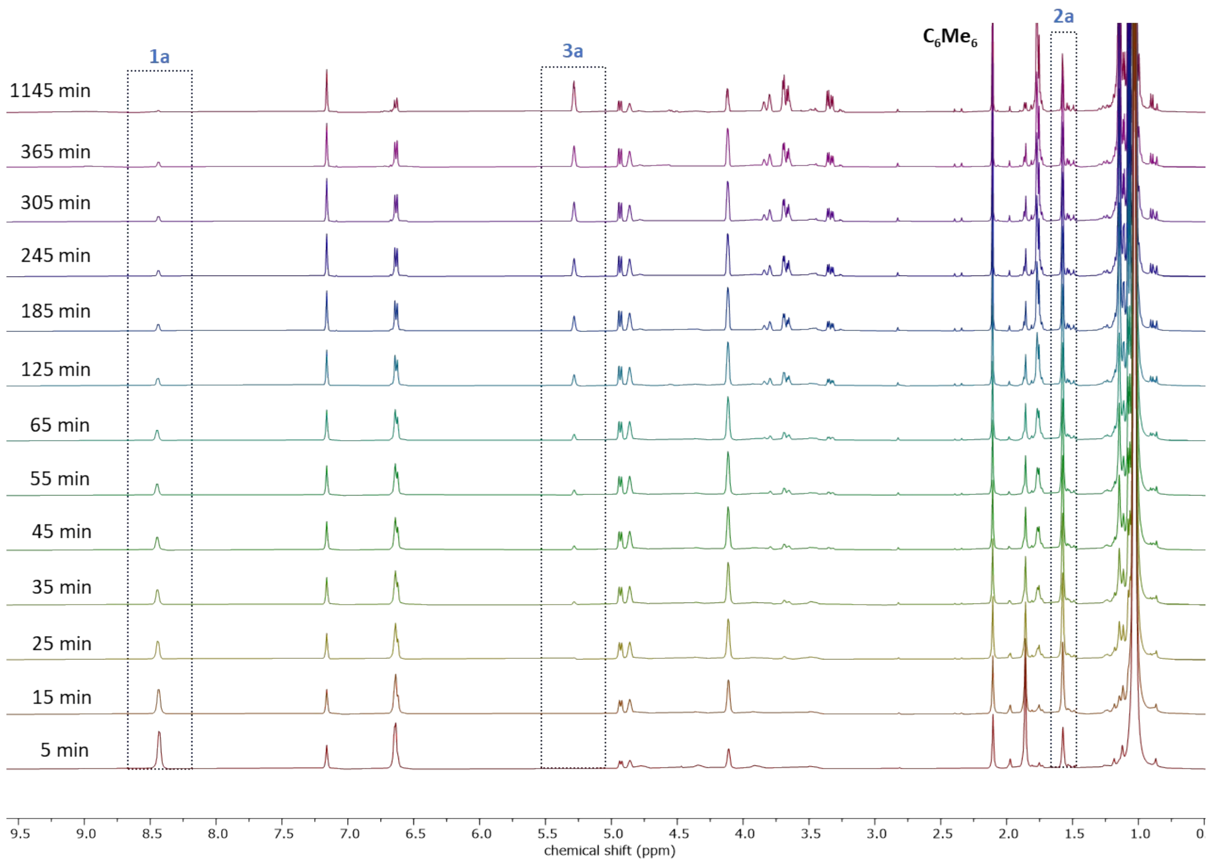

Scheme S10: *In situ* ^{1}H-NMR monitoring of the **Co2** catalysed hydroboration of **1a**. Overlay of the ^{1}H-NMR spectra recorded at differing time points. The resonances used for quantification are highlighted.

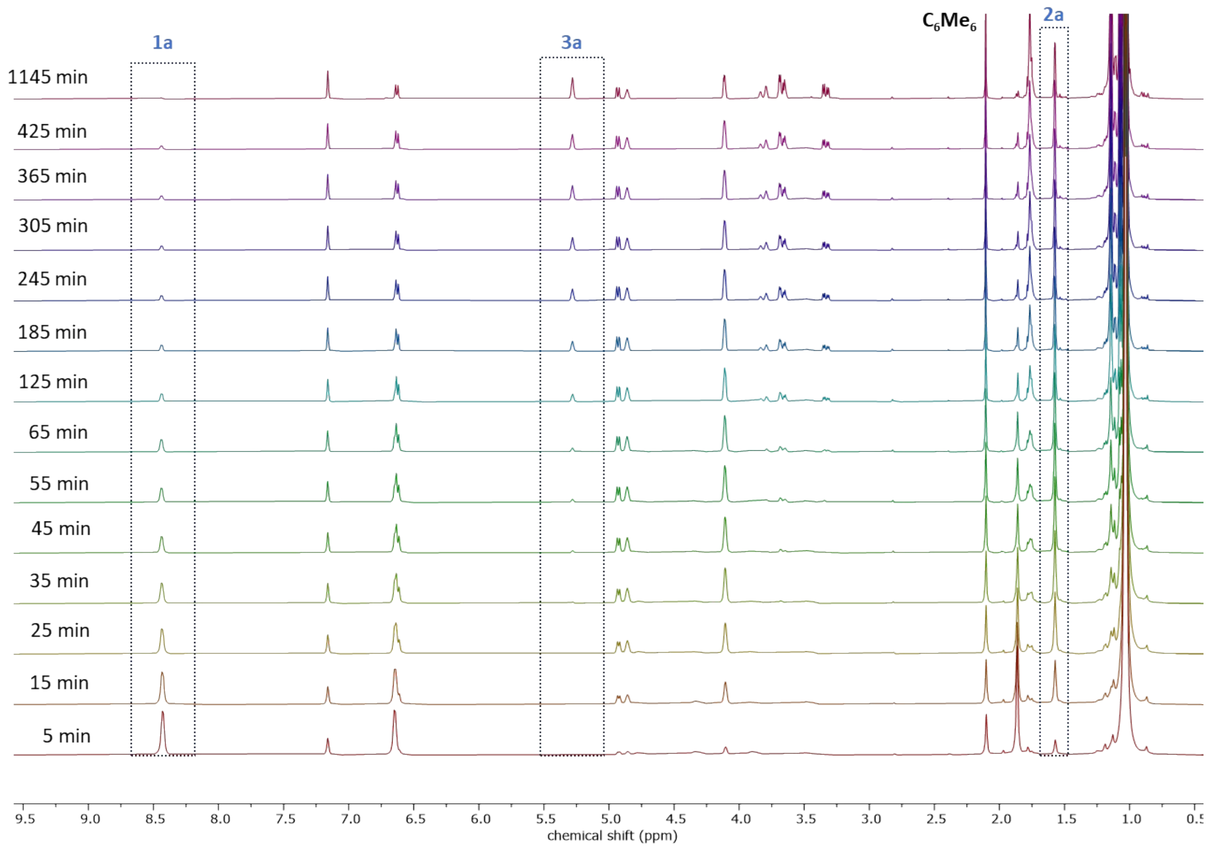

Scheme S11: *In situ* ^{1}H-NMR monitoring of the **Co6** catalysed hydroboration of **1a**. Overlay of the ^{1}H-NMR spectra recorded at differing time points. The resonances used for quantification are highlighted.

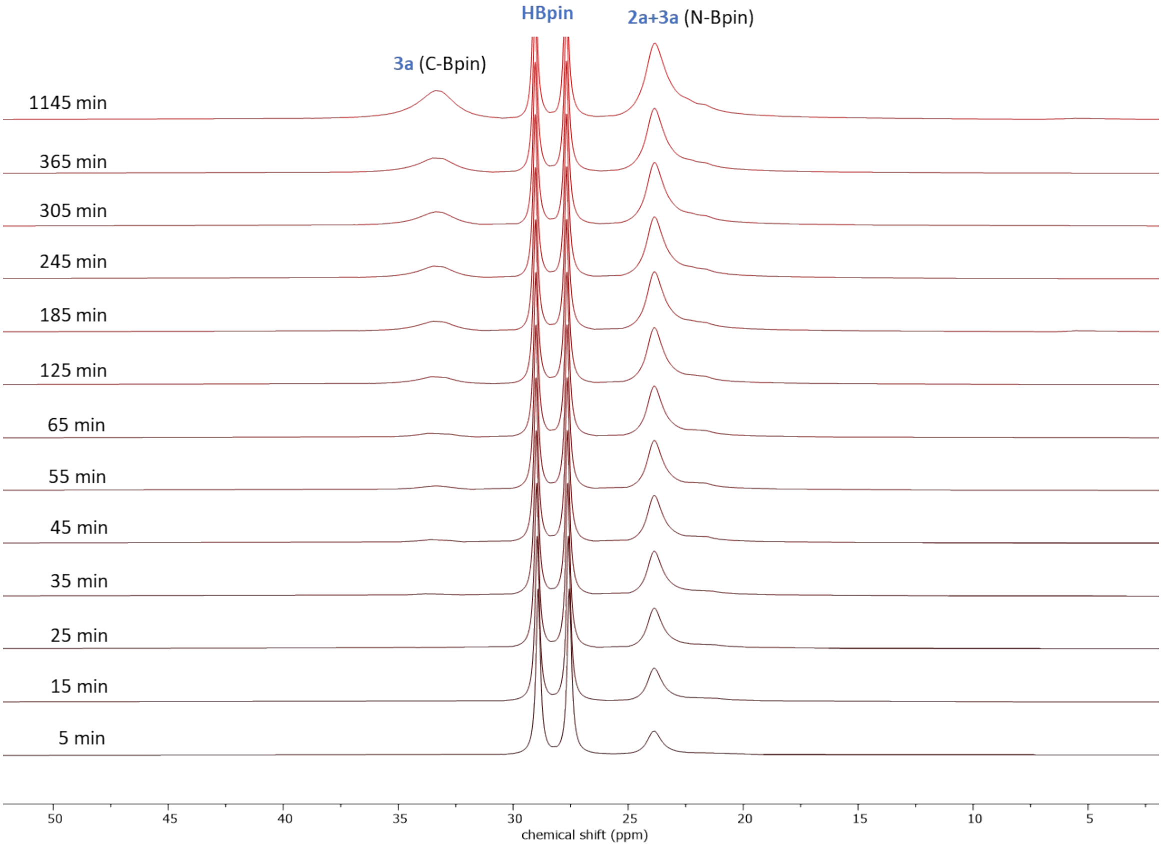

Scheme S12: *In situ* ^{11}B-NMR monitoring of the **Co2** catalysed hydroboration of **1a**. Overlay of the ^{11}B-NMR spectra recorded at differing time points.

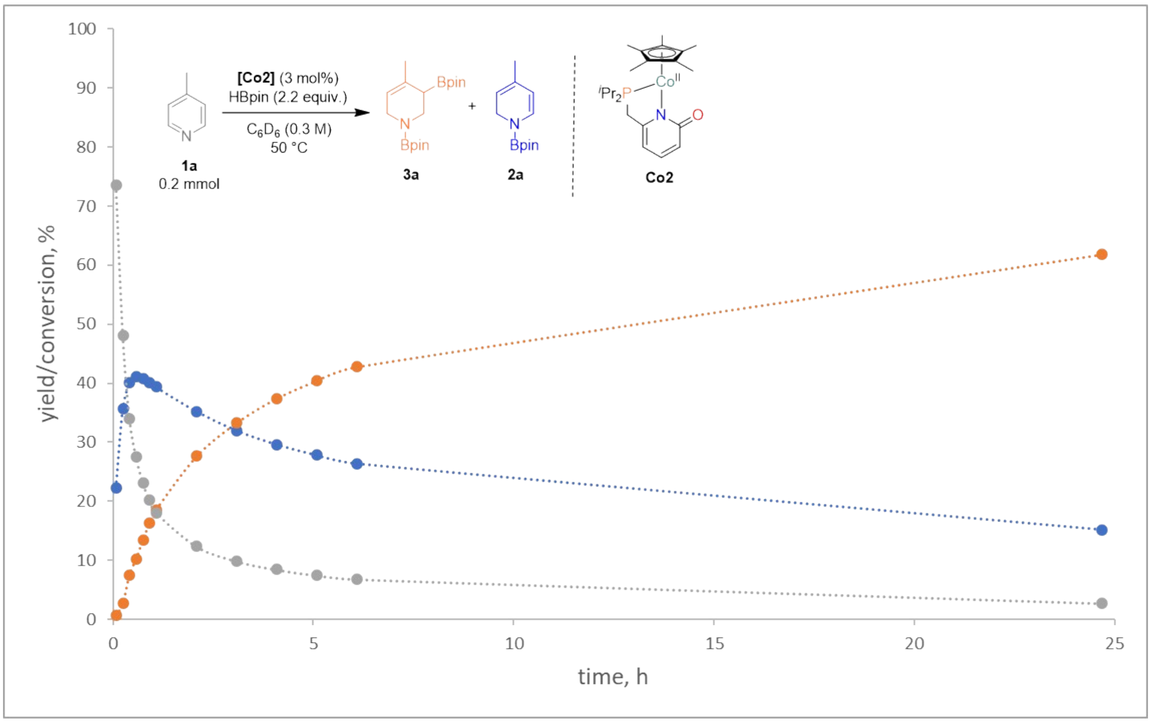

Scheme S13: Kinetic profile of the **Co2** catalysed hydroboration of **1a** as monitored by ^{1}H-NMR spectroscopy.

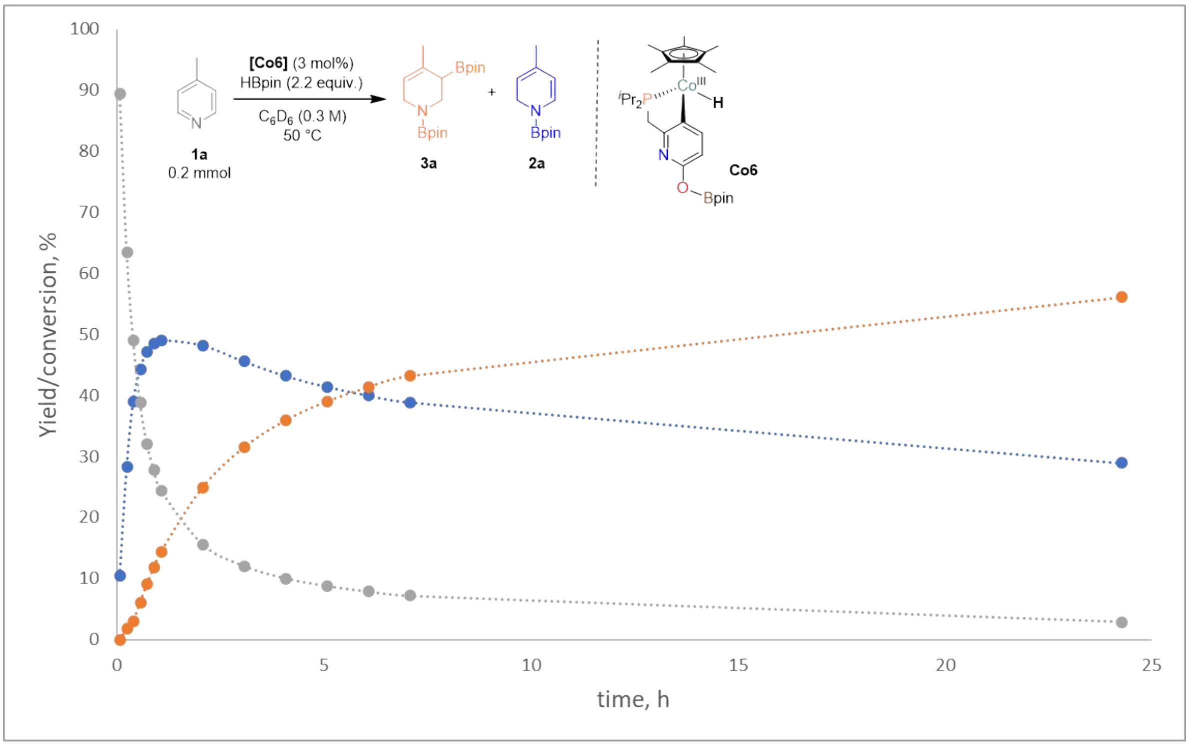

Scheme S14: Kinetic profile of the **Co6** catalysed hydroboration of **1a** as monitored by ^{1}H-NMR spectroscopy.

7.2 *In situ* Generation of L1Bpin

Inside a glovebox filled with argon, a J. Young NMR tube was charged with **L1H** (15 mg, 67 µmol)**,** HBpin (10 µL, 67 µmol) and 0.6 mL THF-d_8 and the reaction mixture was heated to 60 **°**C for 16 h. Clean conversion of starting materials to form the coupling product **L1Bpin** was confirmed by ^{1}H-, ^{11}B- and ^{31}P-NMR spectra.

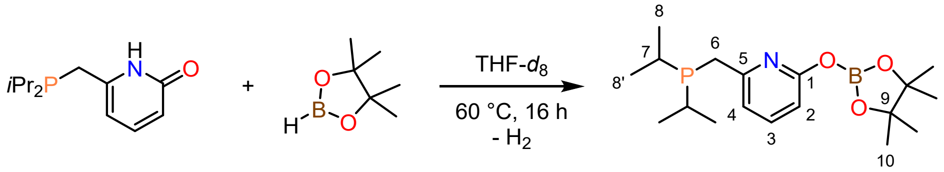

^{1}H-NMR (300.1 MHz, THF-d_8, 22.9 °C): δ [ppm] = 7.52 (pseudo-t, $^3J_{(H,H)}$ = 7.7 Hz, 1 H, 3-H), 6.97 (br, d, $^3J_{(H,H)}$ = 7.4 Hz, 1 H, 4-H), 6.63 (br, d, $^3J_{(H,H)}$ = 8.0 Hz, 2-H), 2.86 (br, s, 2 H, 6-H), 1.80 (overlap with THF-d_8, heptd, $^3J_{(H,H)}$ = 7.1 Hz, $^2J_{(P,H)}$ = 1.6 Hz, 2 H, 7-H), 1.28 (s, 12 H, 10-H), 1.13–0.98 (m, 12 H, 8-H).

^{13}C{^{1}H}-NMR (75.5 MHz, THF-d_8, 22.9 °C): δ [ppm] = 160.8 (C-1), 160.6 (d, $^2J_{(P,C)}$ = 8.2 Hz, C-5), 140.0 (C-3), 119.4 (d, $^3J_{(P,C)}$ = 5.7 Hz, C-4), 110.5 (br, C-2), 84.1 (2 C, C-9), 32.7 (d,

$^{1}J_{(P,C)}$ = 23.9 Hz, C-6), 25.1 (overlap with THF-d_8, 4 C, C-10), 24.5 (d, $^{1}J_{(P,C)}$ = 16.1 Hz, C-7), 20.3 (d, $^{2}J_{(P,C)}$ = 15.5 Hz, 8/8'-C), 19.7 (d, $^{2}J_{(P,C)}$ = 11.4 Hz, 8/8'-C).

^{31}P{^{1}H}-NMR (162.0 MHz, THF-d_8, 26.5 °C): δ [ppm] = 12.1 (s).

^{11}B-NMR (128.4 MHz, THF-d_8, 26.5 °C): δ [ppm] = 22.1 (br, s).

7.3 Stoichiometric Reaction between Co2 and HBpin

NMR analysis:

Inside a glovebox filled with argon, an oven-dried scintillation vial was charged with a solution of **Co2** (16 mg, 38 µmol, 1.0 eq) in THF-d_8 or C_6D_6 (0.6 mL). Then, HBpin (6 µL, 40 µmol, 1.05 eq.) was added resulting in an instant colour change from red to black. The reaction mixture was stirred overnight at ambient temperature and transferred to a J. Young NMR tube. ^{1}H- and ^{11}B-NMR spectra were recorded. The ^{11}B-NMR spectrum showed complete consumption of HBpin (see Scheme S15 and S16). The ^{1}H NMR spectrum showed a mixture of paramagnetic and diamagnetic compounds (Scheme S17–Scheme S20).

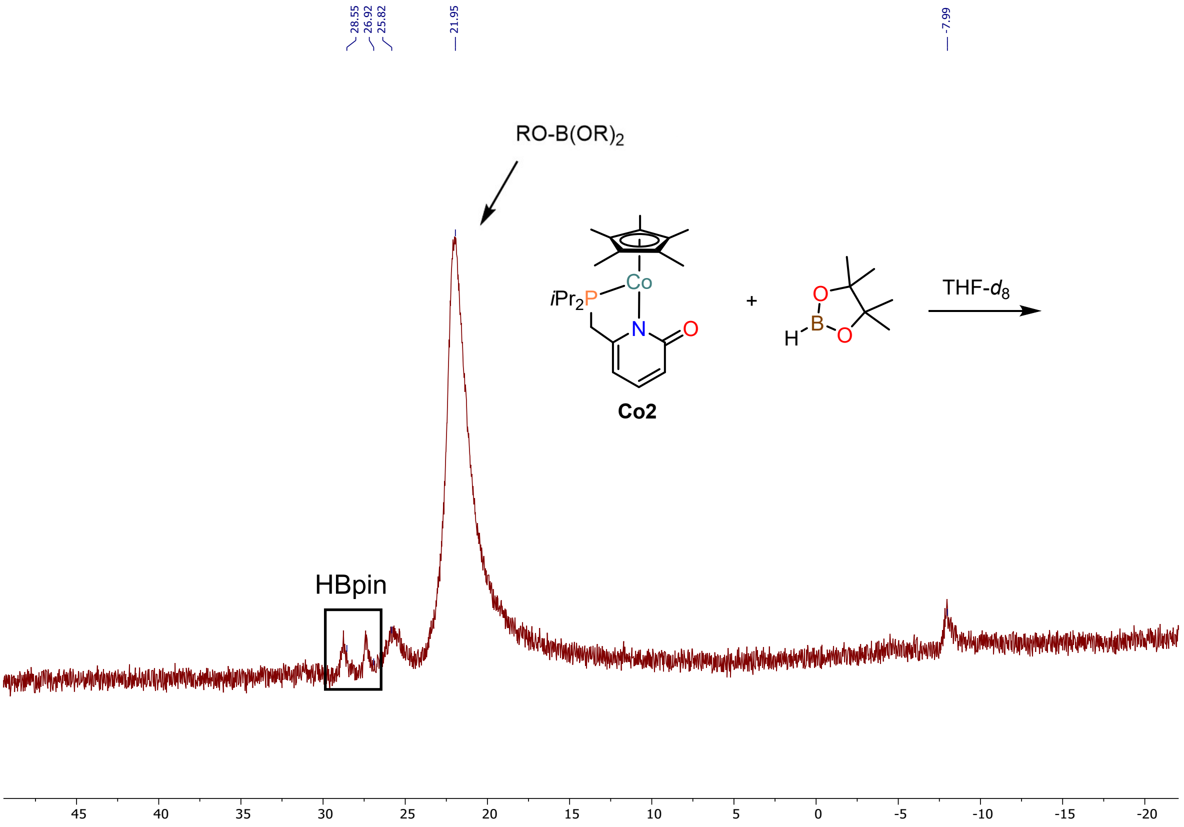

Scheme S15: 128 MHz ^{11}B-NMR spectrum of the reaction mixture of **Co2** and pinacolborane in THF-d_8.

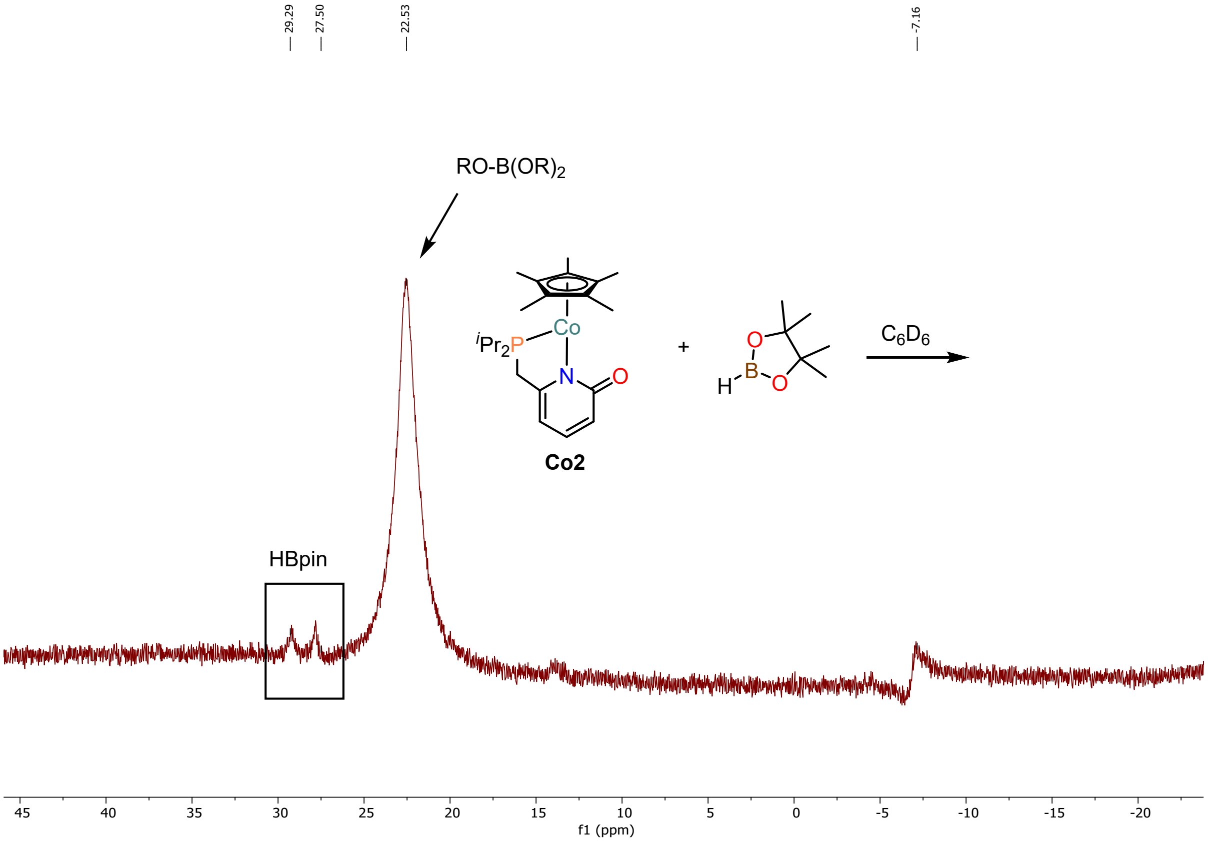

Scheme S16: 128 MHz ^{11}B-NMR spectrum of the reaction mixture of **Co2** and pinacolborane in C_6D_6.

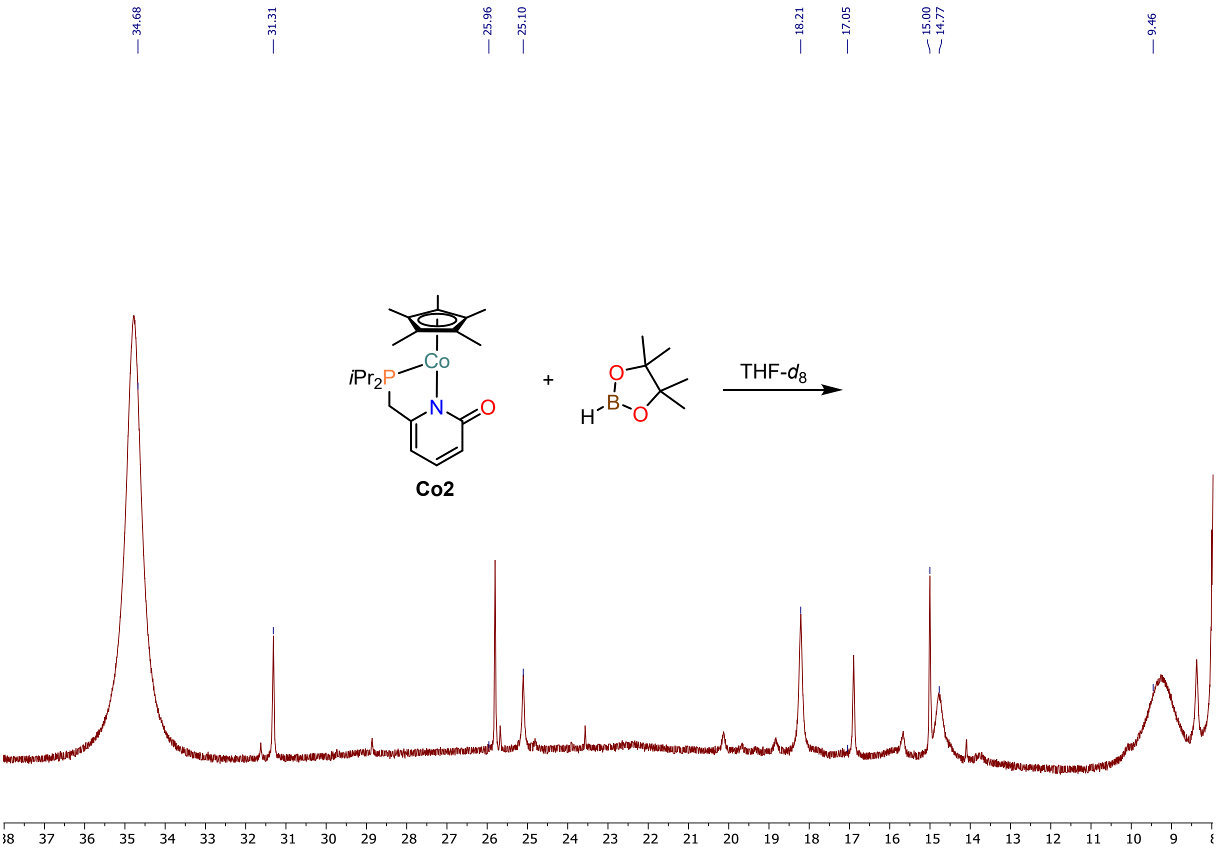

Scheme S17: Paramagnetic region of the ^{1}H-NMR spectrum (600 MHz, THF-d_8) of the **Co2** + HBpin mixture.

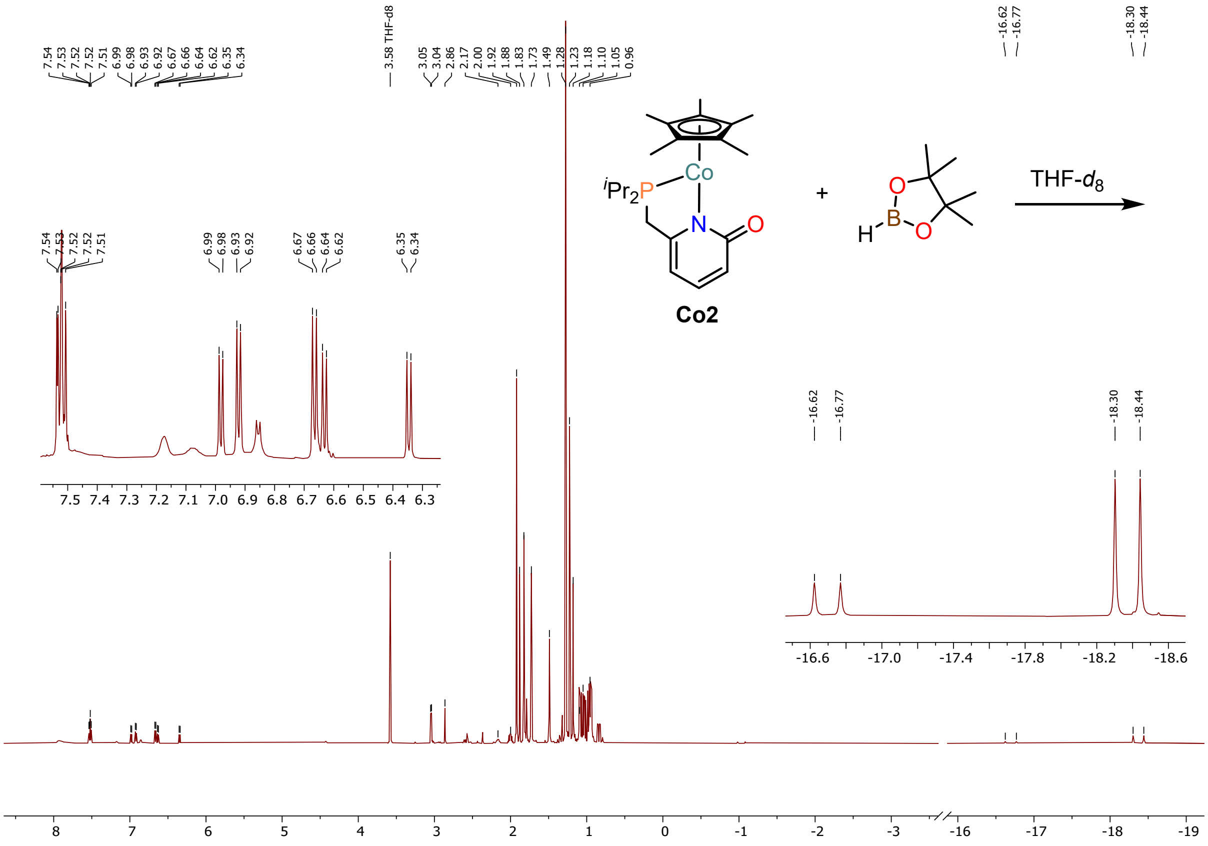

Scheme S18: Diamagnetic region of the ^{1}H-NMR spectrum (600 MHz, THF-d_8) of the **Co2** + HBpin mixture.

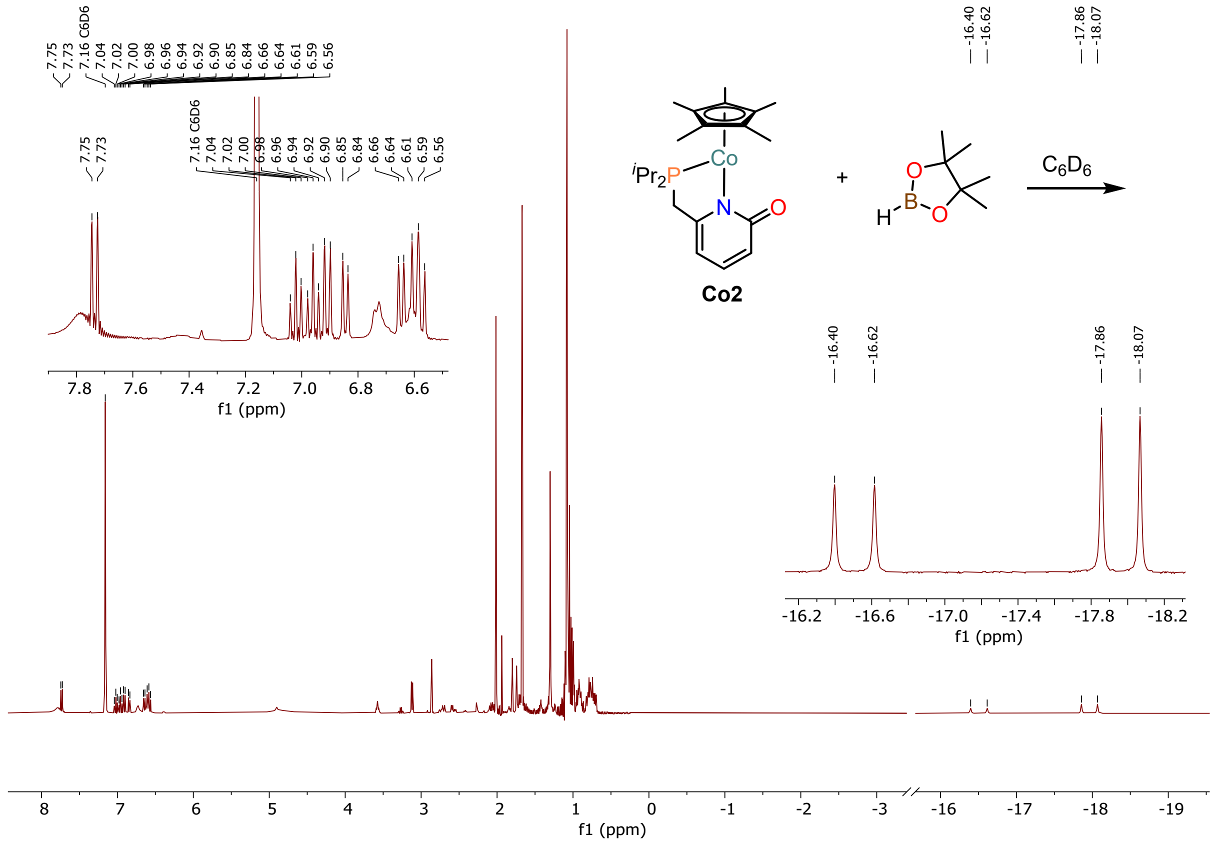

Scheme S19: Diamagnetic region of the ^{1}H-NMR spectrum (400 MHz, C_6D_6) of the **Co2** + HBpin mixture.

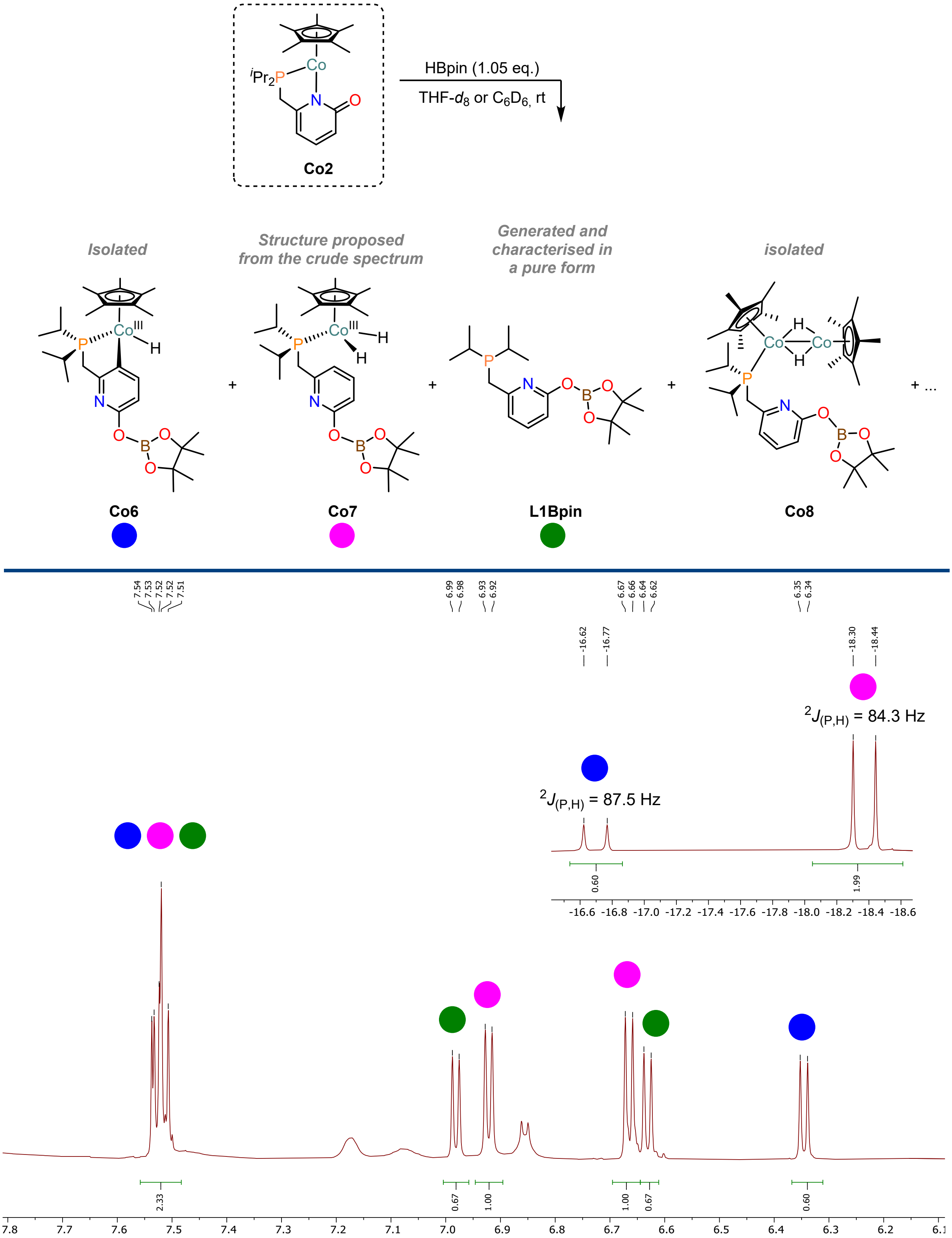

Scheme S20: ^{1}H-NMR spectrum (600 MHz, THF-d_8) of the **Co2** + HBpin mixture with assignments.

MS-ESI analysis:

Sample preparation: A mixture of **Co2** and HBpin (1.2 eq.) was prepared *in situ* in THF at room temperature and directly subjected to ESI-MS analysis.

The sample solution was transferred into a gas-tight syringe and fed into the ESI source of a micrOTOF-Q II mass spectrometer (*Bruker Daltonik*) at a flow rate of 0.5 mL·h^{-1}. The ESI source was operated at a voltage of 4500 V with N_2 as nebulizer (8.0 psi backing pressure) and drying gas (heated to 333 K and held at 3.0 L·min^{-1} flow rate). The hereby produced ions with 50 ≤ *m/z* ≤ 3000 were then allowed to pass the instrument's quadrupole mass filter and collision cell before entering the time-of-flight (TOF) mass analyser. Ions were identified on the basis of their *m/z* ratio, their isotope pattern, and fragmentation patterns.

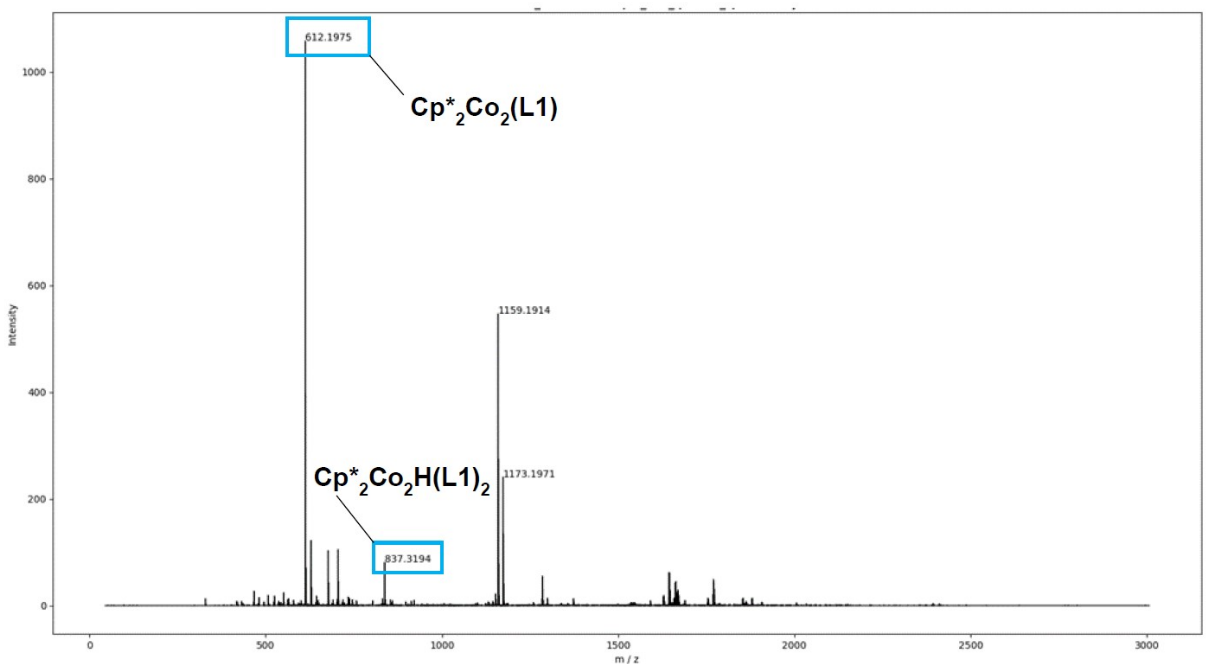

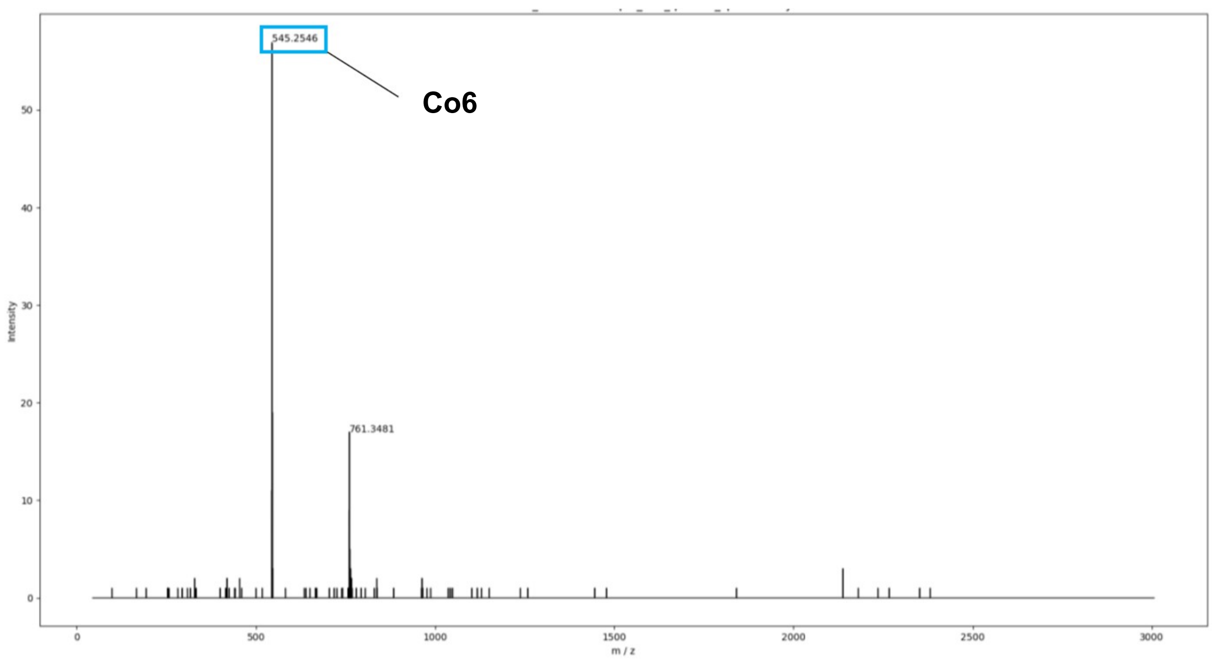

Scheme S21: Positive-ion mode ESI mass spectra of the products formed in the reaction of **Co2** with HBpin (1.2 eq.) in THF.

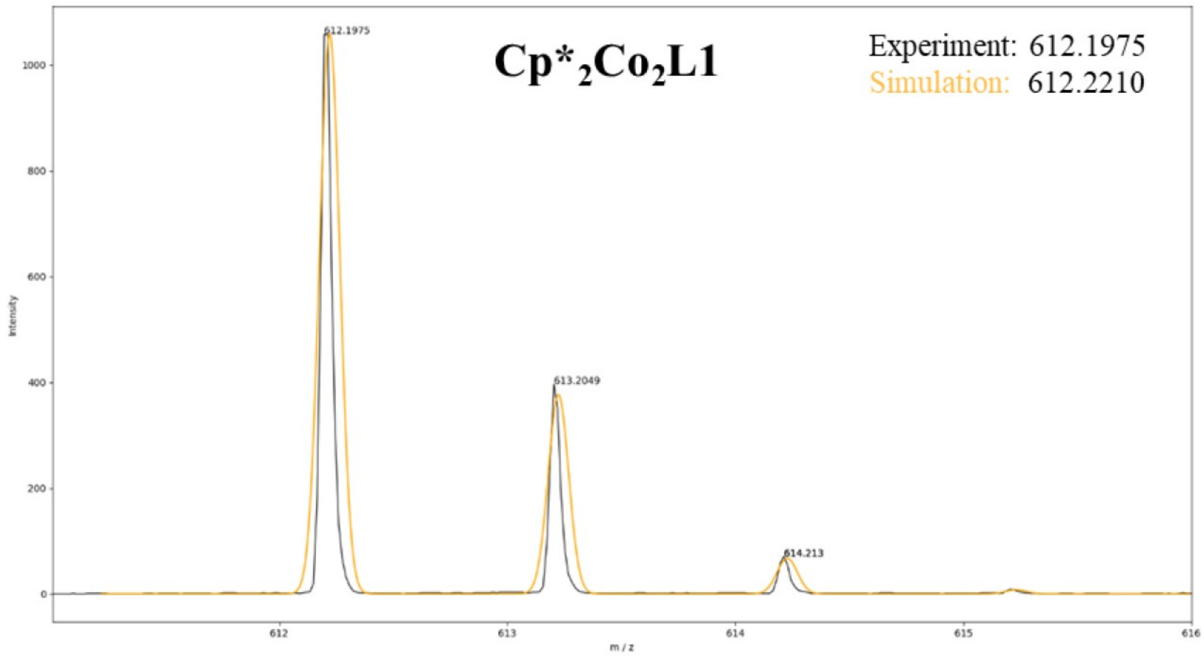

Scheme S22: Measured (black) and simulated (orange) isotopic patterns of *in situ* generated Cp*$_2$Co$_2$(L1).

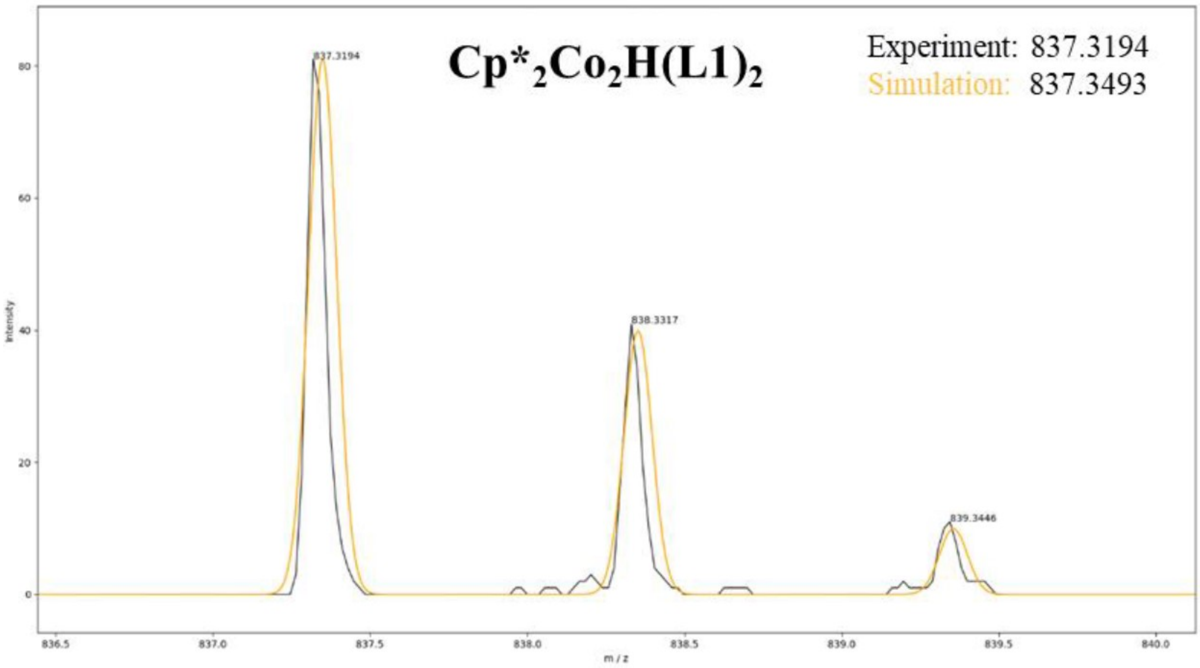

Scheme S23: Measured (black) and simulated (orange) isotopic patterns of *in situ* generated $Cp^*{}_2Co_2H(L1)_2$.

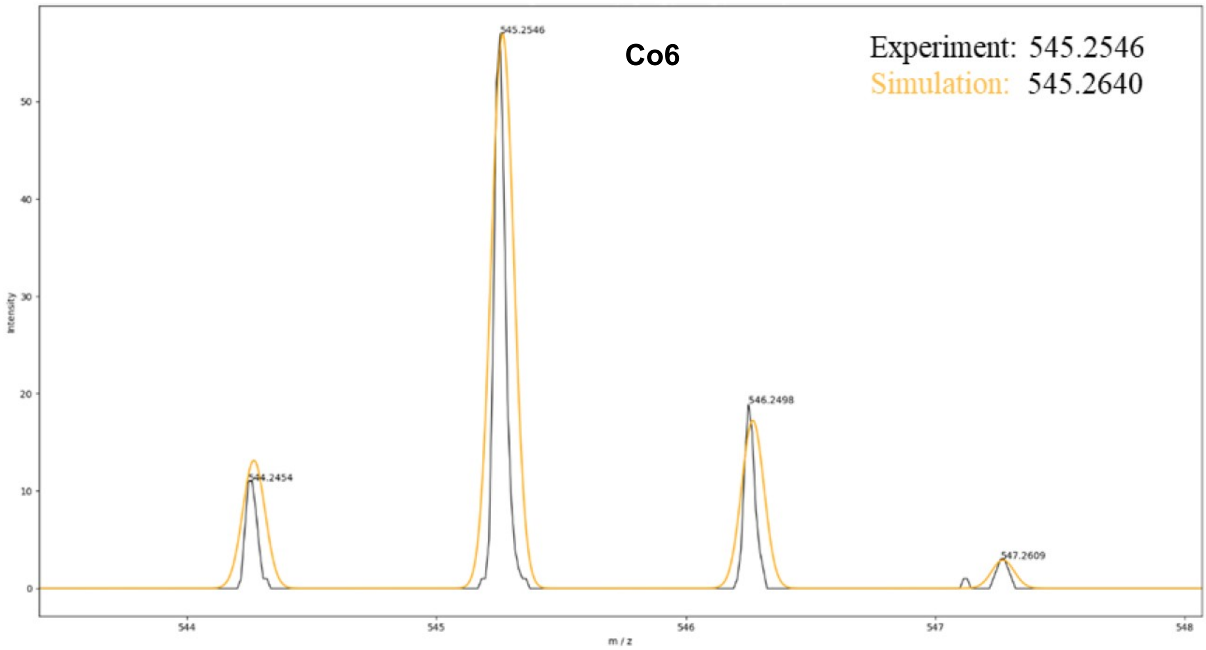

Scheme S24: Measured (black) and simulated (orange) isotopic patterns of *in situ* generated **Co6**.

7.4 Stoichiometric Reaction between Co2 and DBpin

Inside a glovebox filled with argon, an aliquote of a DBpin stock solution (1.22 M in C_6D_6, 47 µL, 57 µmol) was added to a solution of **Co2** (20 mg, 48 µmol) in THF-d_8 (0.6 mL). The reaction mixture was transferred to a J. Young NMR tube and analysed by ^{1}H-NMR spectroscopy.

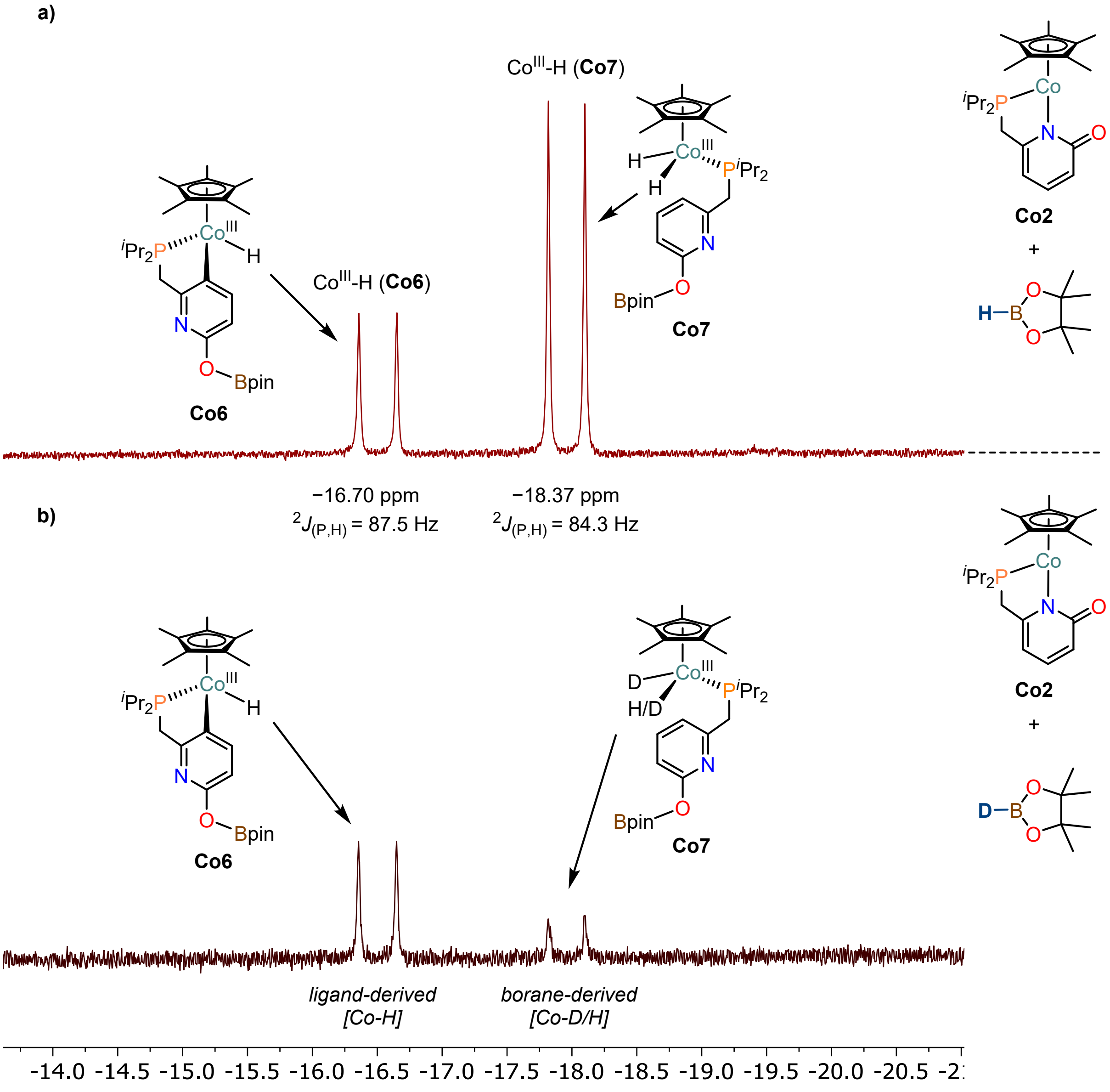

Scheme S25: Upfield section of the ^{1}H-NMR (300 MHz, THF-d_8) spectra of the deuteration experiment for cobalt hydride generation. a) Reaction of **Co2** and **H**Bpin in THF-d_8. b) Reaction of **Co2** and **D**Bpin in THF-d_8.

7.5 Stoichiometric Reaction between Co6 and HBpin

Inside a glovebox filled with argon, a solution of **Co6** (11 mg, 20 µmol, 1.0 eq.) in C_6D_6 (0.6 mL) was transferred to a J. Young NMR tube and subsequently analysed by ^{1}H-NMR spectroscopy. Then, one equivalent of HBpin (3 µL, 20 µmol, 1.0 eq.) was added, the NMR tube was closed and shaken vigorously. After 4 hours at ambient temperature, NMR spectra were recorded once again. Subsequently, a second equivalent of HBpin (3 µL, 20 µmol, 1.0 eq.) was added, the NMR tube was closed and shaken vigorously. After leaving the reaction mixture at ambient temperature for 24 h, NMR spectra were recorded. Lastly, the reaction mixture was heated to 50 °C for 20 h before measuring the last NMR spectra.

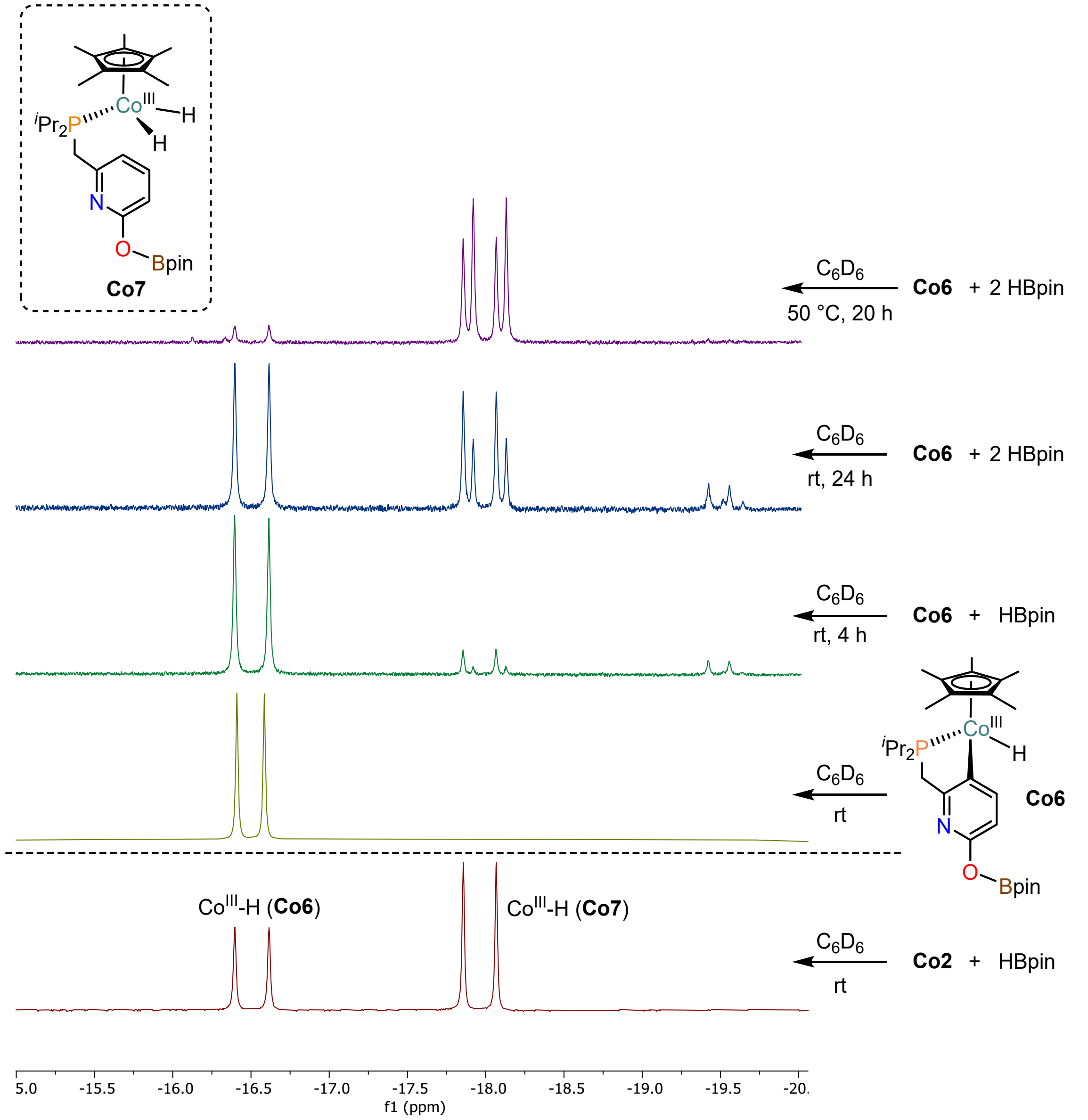

Scheme S26: Upfield region of the ^{1}H-NMR spectra (400–500 MHz, C_6D_6) recorded to test for the stability of **Co6** towards HBpin. The respective spectra were recorded after applying the reaction conditions on the right.

7.6 Stoichiometric Reaction between Co6 and 4-methylpyridine 1a

Inside a glovebox filled with argon, a solution of **Co6** (20 mg, 37 µmol, 1.0 eq.) in C_6D_6 (0.6 mL) was transferred to a J. Young NMR tube and subsequently analysed by ^{1}H- and ^{11}B-NMR spectroscopy. Then, 4-methylpyridine **1a** (3.6 µL, 37 µmol, 1.0 eq.) was added. The NMR tube was closed, shaken vigorously and subsequently analysed by ^{1}H- and ^{11}B-NMR spectroscopy. Then, the reaction mixture was heated to 50 °C for 17 h and NMR spectra were recorded once again. One equivalent of HBpin (5.5 µL, 37 µL, 1.0 eq.) was added, the NMR tube was closed, shaken vigorously, left at room temperature for 17 h and analysed by NMR spectroscopy. The reaction mixture was heated to 50 °C for 4 h and NMR spectra were recorded. Lastly, a second equivalent of HBpin (5.5 µL, 37 µL, 1.0 eq.) was added, the NMR tube was closed, shaken vigorously and the mixture was heated to 50 °C for 17 hours before measuring a last set of NMR spectra.

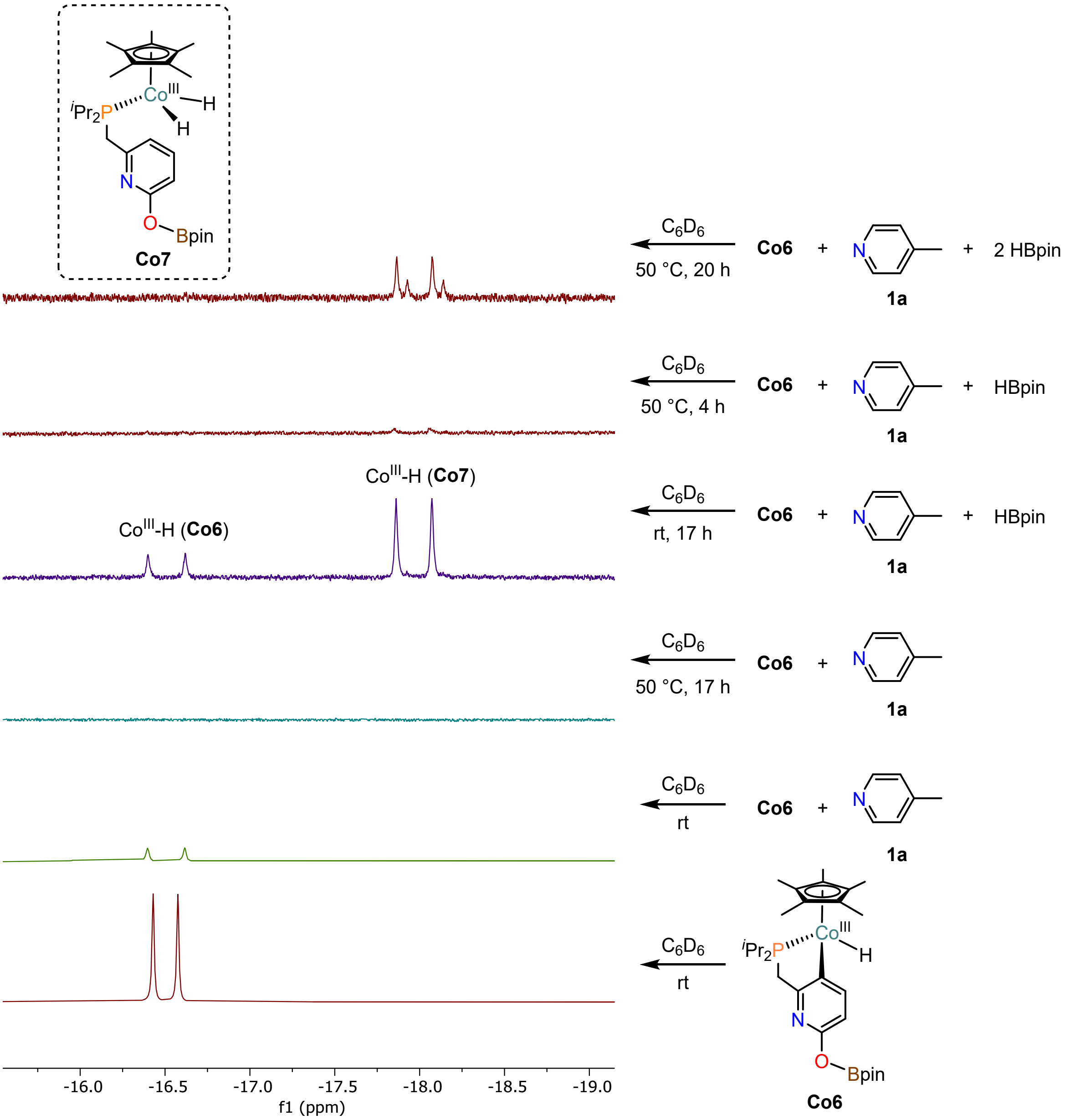

Scheme S27: Upfield regions of the ^{1}H-NMR spectra (400–600 MHz, C_6D_6) of the stoichiometric reaction between **Co6** and **1a** with subsequent addition of HBpin. The respective spectra were recorded after applying the reaction conditions on the right.

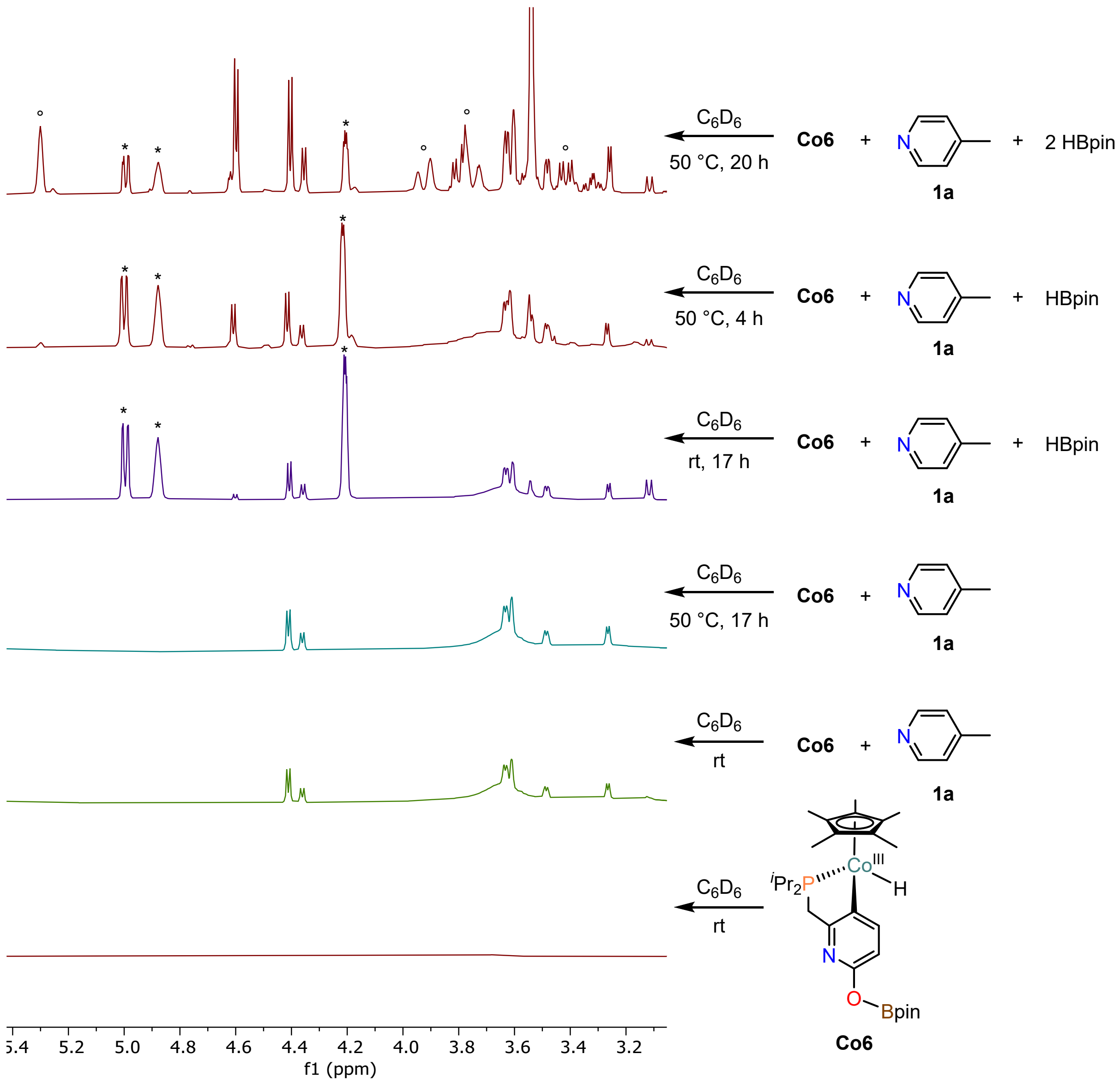

Scheme S28: Fragments of the ^{1}H-NMR spectra (400–600 MHz, C_6D_6) of the stoichiometric reaction between **Co6** and **1a** with subsequent addition of HBpin. The respective spectra were recorded after applying the reaction conditions on the right. (*) Signals of 1,2-single hydroboration product **2a**. (°) Signals of double hydroboration product **3a**.

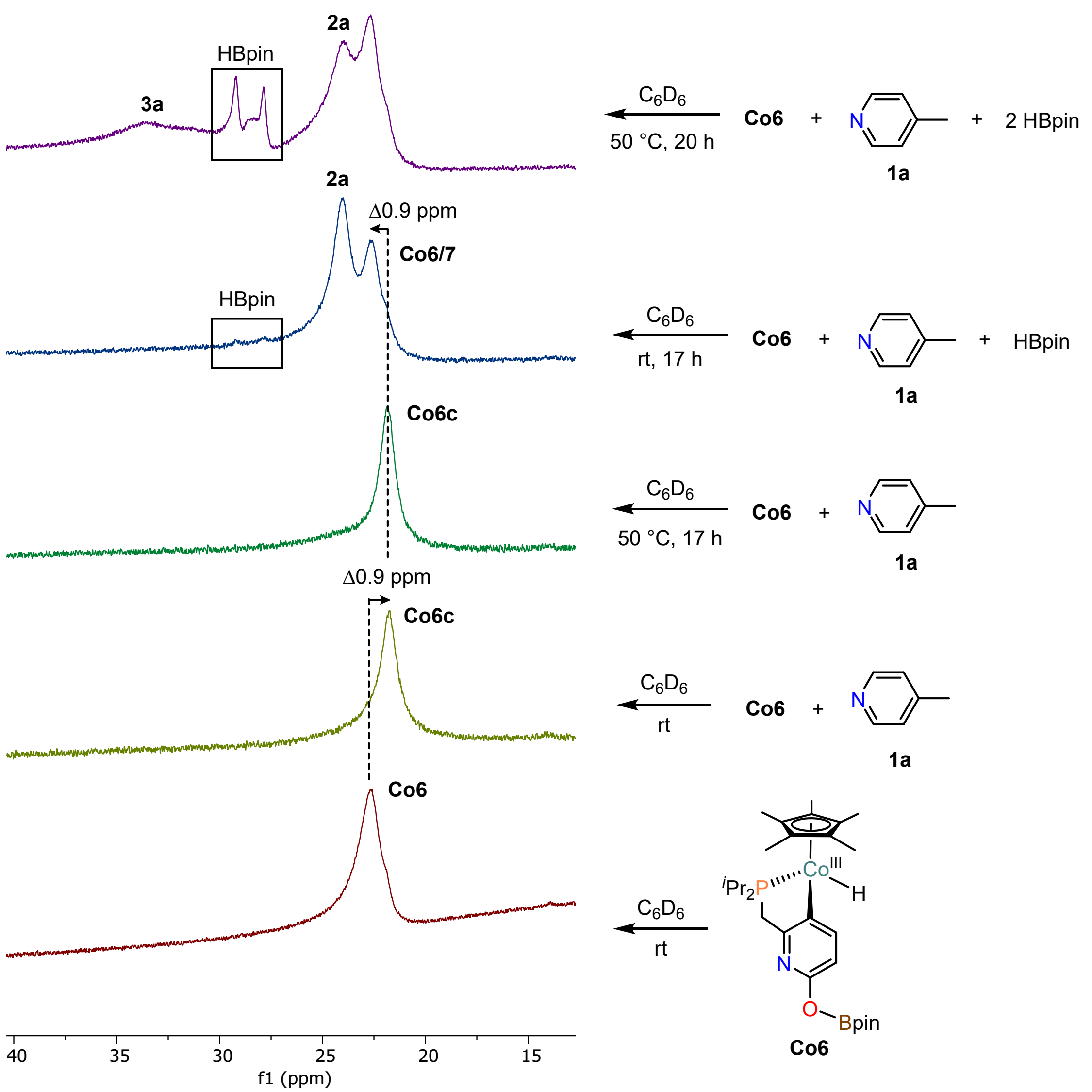

Scheme S29: ^{11}B-NMR spectra (128 MHz, C_6D_6) of the stoichiometric reaction between **Co6** and **1a** with subsequent addition of HBpin. The respective spectra were recorded after applying the reaction conditions on the right.

7.7 Stoichiometric Reaction between Co2, 4-benzylpyridine 1e and HBpin

Inside a glovebox filled with argon, an oven-dried scintillation vial was charged with a solution of **Co2** (20 mg, 48 µmol, 1.0 eq) in C_6D_6 (0.6 mL). Then, 4-benzylpyridine **1e** (7.6 µL, 48 µmol, 1.0 eq.) was added. Subsequently, HBpin (14 µL, 96 µmol, 2.0 eq.) was added resulting in an immediate colour change from red to black. The reaction mixture was transferred to a J. Young NMR tube and analysed by ^{1}H- and ^{11}B-NMR spectroscopy. Afterwards, the reaction mixture was heated to 50 °C for 17 h before recording NMR spectra once again. Then, a third equivalent of HBpin (7 µL, 48 µmol, 1.0 eq.) was added, the NMR tube was closed, shaken vigorously, and heated to 50 °C for 18 h. A last set of NMR spectra were measured.

The same experiment was repeated using only one equivalent of HBpin (7 µL, 48 µmol, 1.0 eq.).

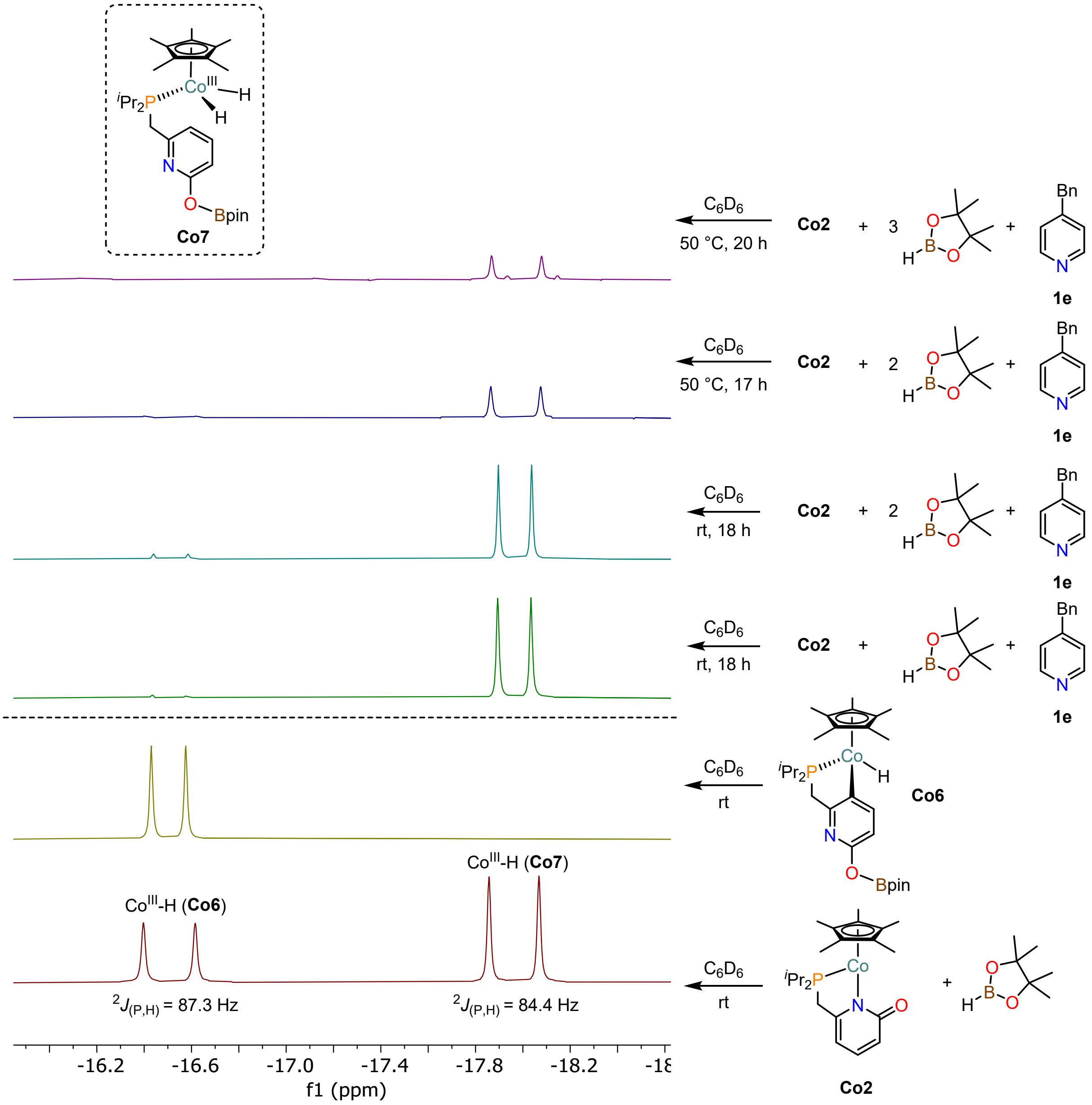

Scheme S30: Upfield regions of the ^{1}H-NMR spectra (400–600 MHz, C_6D_6) of the stoichiometric reaction between **Co2, 1e** and HBpin. The respective spectra were recorded after applying the reaction conditions on the right.

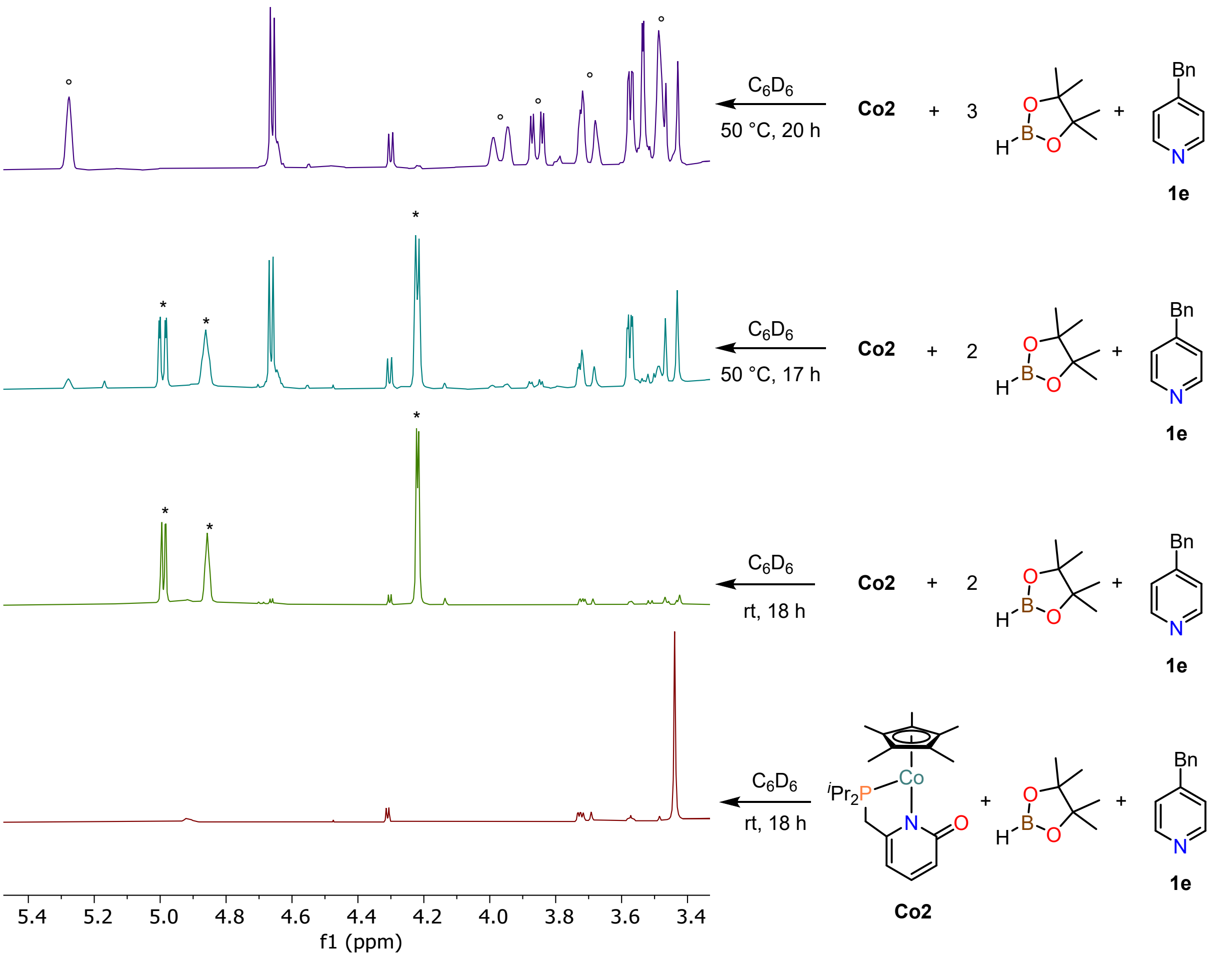

Scheme S31: Fragments of the ^{1}H-NMR spectra (400–600 MHz, C_6D_6) of the stoichiometric reaction between **Co2**, **1e** and HBpin. The respective spectra were recorded after applying the reaction conditions on the right. (*) Signals of 1,2-single hydroboration product **2e**. (°) Signals of double hydroboration product **3e**.

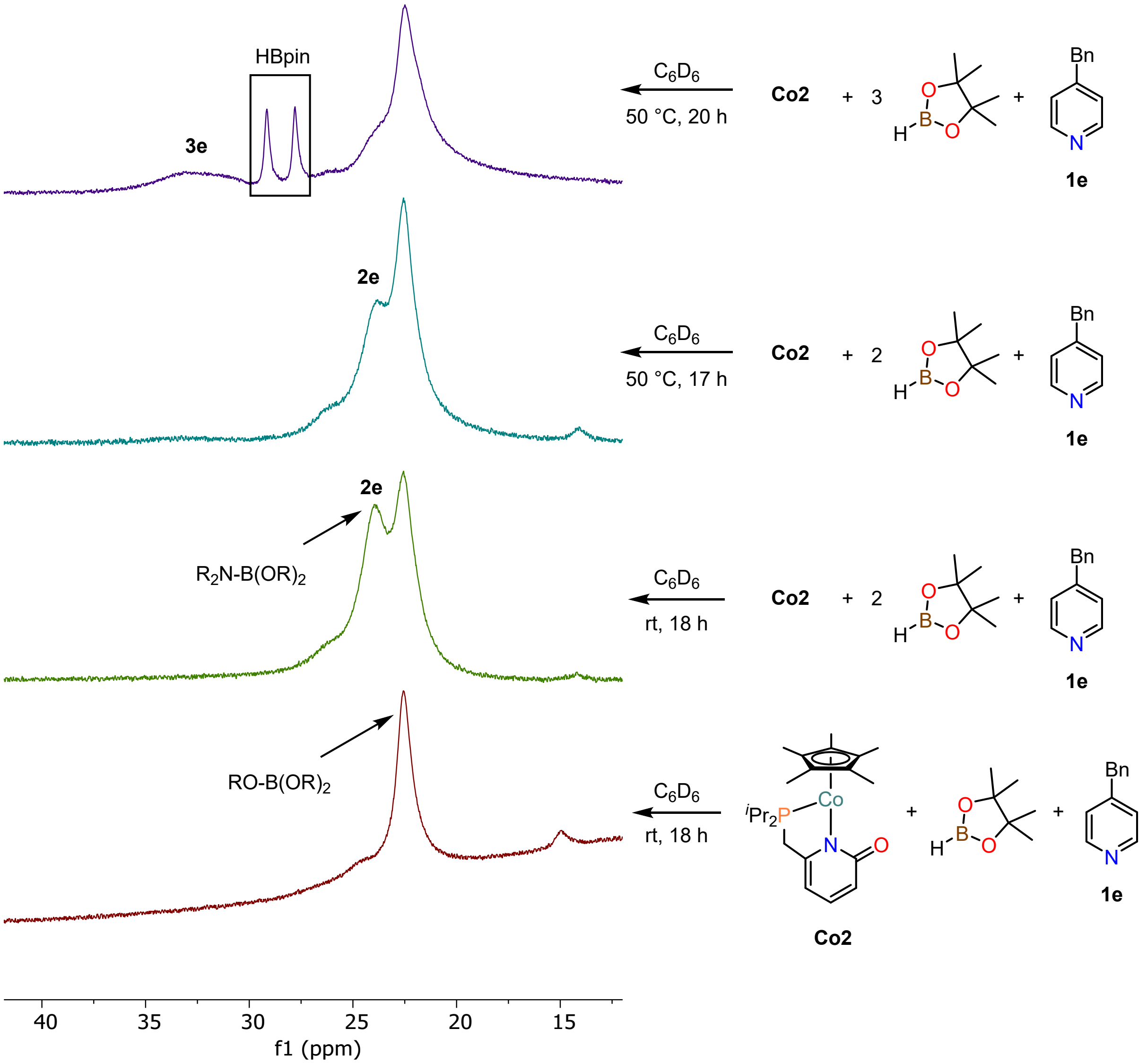

Scheme S32: ^{11}B-NMR spectra (128 MHz, C_6D_6) of the stoichiometric reaction between **Co2**, **1e** and HBpin. The respective spectra were recorded after applying the reaction conditions on the right.

7.8 Deuteration Experiment with 4-Phenylpyridine 1b and DBpin

Inside a glovebox filled with argon, precatalyst **Co2** (2.5 mg, 6.0 µmol, 3.0 mol%) was dissolved in C_6D_6 (0.2 mL) and to the red solution was added 4-phenylpyridine[45] **1b** (2 M in C_6D_6, stored over MS4Å, 100 µL, 0.200 mmol, 1.00 eq.). Then, an aliquot of a stock solution of DBpin in C_6D_6 (1.22 M, 330 µL, 0.403 mmol, 2.01 eq.) was added to the solution. During the addition of DBpin the reaction mixture turned black. The reaction mixture was transferred into a J. Young NMR tube, the NMR tube was closed, taken out of the glovebox and heated to 50 °C for 20 h. Subsequently, the sample was directly analysed by ^{1}H- and ^{2}H-NMR

[45] For this experiment, 4-phenylpyridine **1b** was dried in a vacuum oven at 50 °C for 4 hours, dissolved in C_6D_6 and stored over molecular sieves (4 Å) for 24 h prior to use.

spectroscopy. After NMR analysis the crude mixture was subjected to acylation with trifluoroacetic anhydride (TFAA) followed by GC-MS analysis according to the general procedures described 10.2.

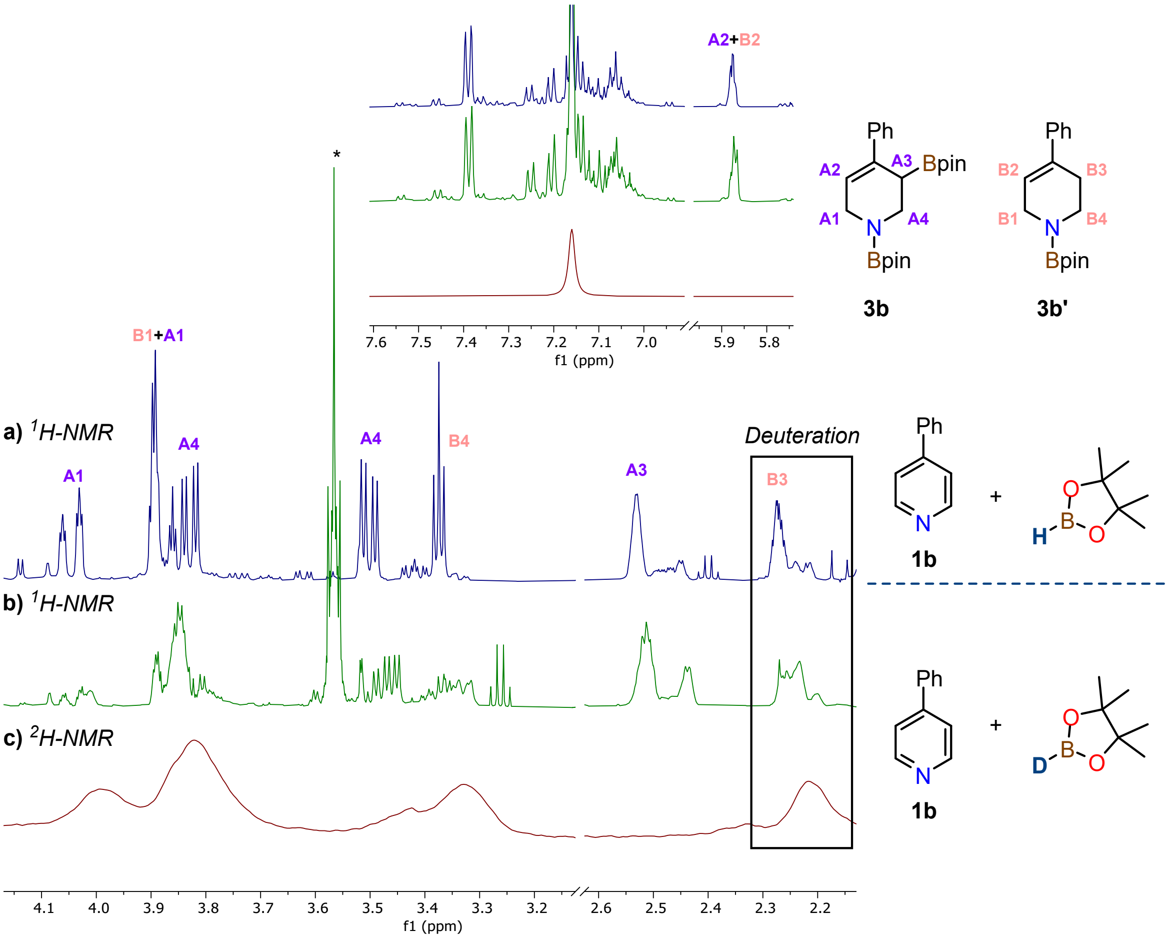

Scheme S33: Deuteration experiment with 4-phenylpyridine **1b**. a) ^{1}H-NMR spectrum (600 MHz, C_6D_6) of the crude mixture of the reaction of 4-phenylpyridine **1b** (0.2 mmol) with **H**Bpin (2.2 eq.) using *Method A*. b) ^{1}H-NMR spectrum (600 MHz, C_6D_6) of the crude mixture of the reaction of 4-phenylpyridine **1b** (0.2 mmol) with **D**Bpin (2.0 eq.) using *Method A*. c) ^{2}H-NMR spectrum (92 MHz, C_6D_6) of the latter. (*) Unidentified signal.

The ^{1}H-NMR signals of the alkenyl protons as well as the aromatic protons were not affected (Scheme S33, upper panel) by the use of DBpin. However, the ^{1}H-NMR signals of the CH_2N groups of **3b** and **3b'** significantly decreased in intensity and new coupling modes were observed due to deuteration (A1, A4, B1, B4 in Scheme S33a+b). Furthermore, while the allylic (pinB)C*H* signal of **3b** (A3) was unaffected by the use of DBpin, the allylic C=C(Ph)CH_2 resonance of **3b'** (B3) was approximately halved in intensity and exhibited a change in multiplicity indicating deuteration (Scheme S33a+b). These results were supported by ^{2}H-NMR analysis. A signal at 2.21 ppm can be observed for the C=C(Ph)CH*D* group together with signals at 3.33 ppm, 3.82 ppm and 3.99 ppm for the respective CH*D*N moieties (Scheme S33c).

GC-MS analysis after acylation with trifluoroacetic anhydride allowed for the detection of dideuterated allylboron species **3b’’-*d*$_2$** ([M]$^{•+}$ = *m/z* 383.0) as well as trideuterated tetrahydropyridine **3b’’’-*d*$_3$** ([M]$^{•+}$ = *m/z* 258.1) (Scheme S34).

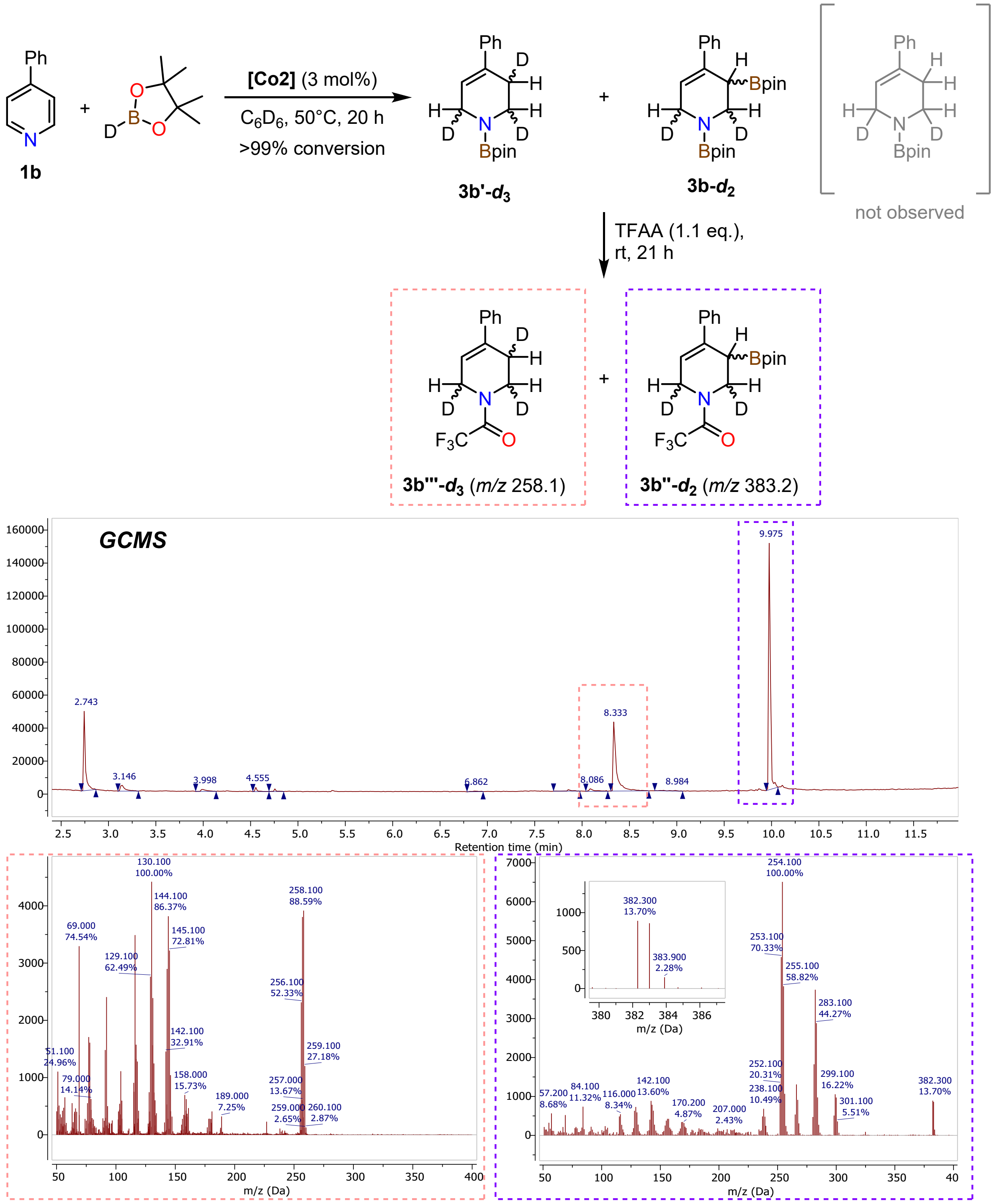

Scheme S34: Deuteration experiment with 4-phenylpyridine **1b**. Derivatisation of the crude products with TFAA allowed for the detection of dideuterated **3b’’-*d*$_2$** as well as trideuterated **3b’’’-*d*$_3$** by GC-MS. The [M-H]$^{•+}$ peak found in the EI-MS spectra is caused by proton abstraction.

7.9 Catalytic Double Hydroboration of Pyridine-2-d

Pyridine-2-d (0.4 mmol) was subjected to **Co2** catalysed hydroboration in C_6D_6 according to the general procedure for NMR scale double hydroboration reactions described in 3.1. Subsequently, the reaction mixture was monitored by ^{1}H-, ^{2}H- and ^{11}B-NMR spectroscopy. Then the crude mixture was treated with trifluoroacetic anhydride and analysed by GC-MS according to procedure 10.2.

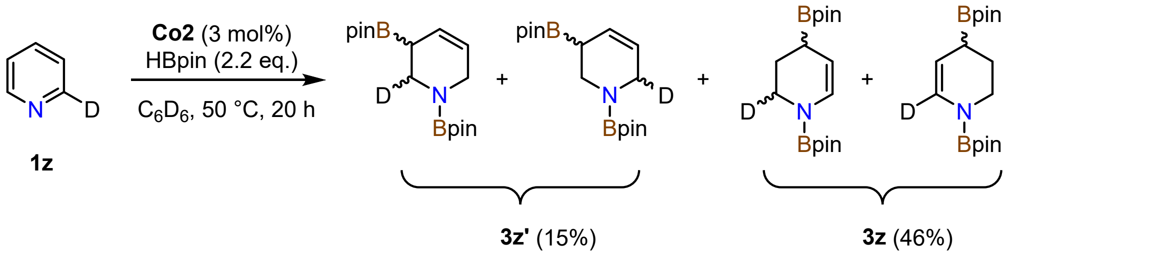

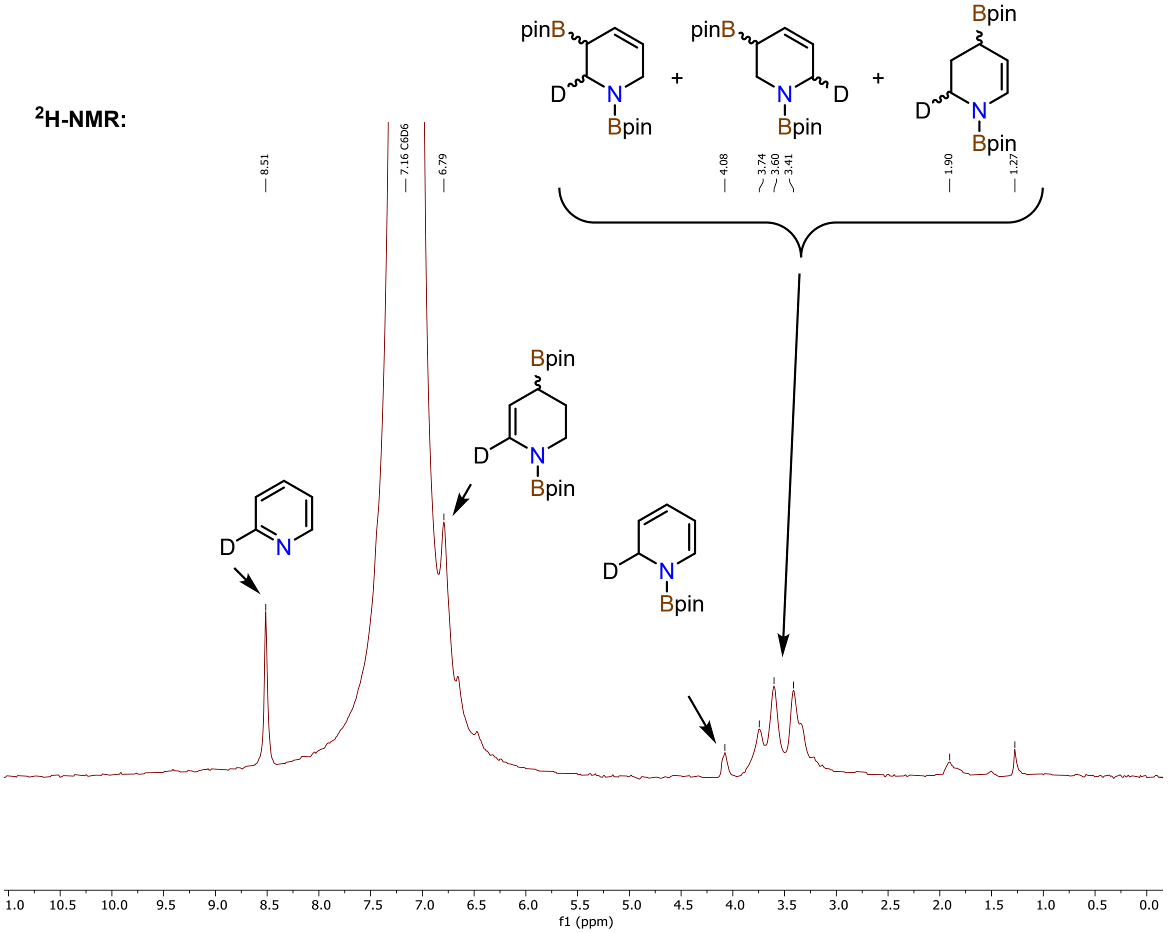

Scheme S35: Products observed during the **Co2** catalysed double hydroboration of pyridine-2-d **1z**.

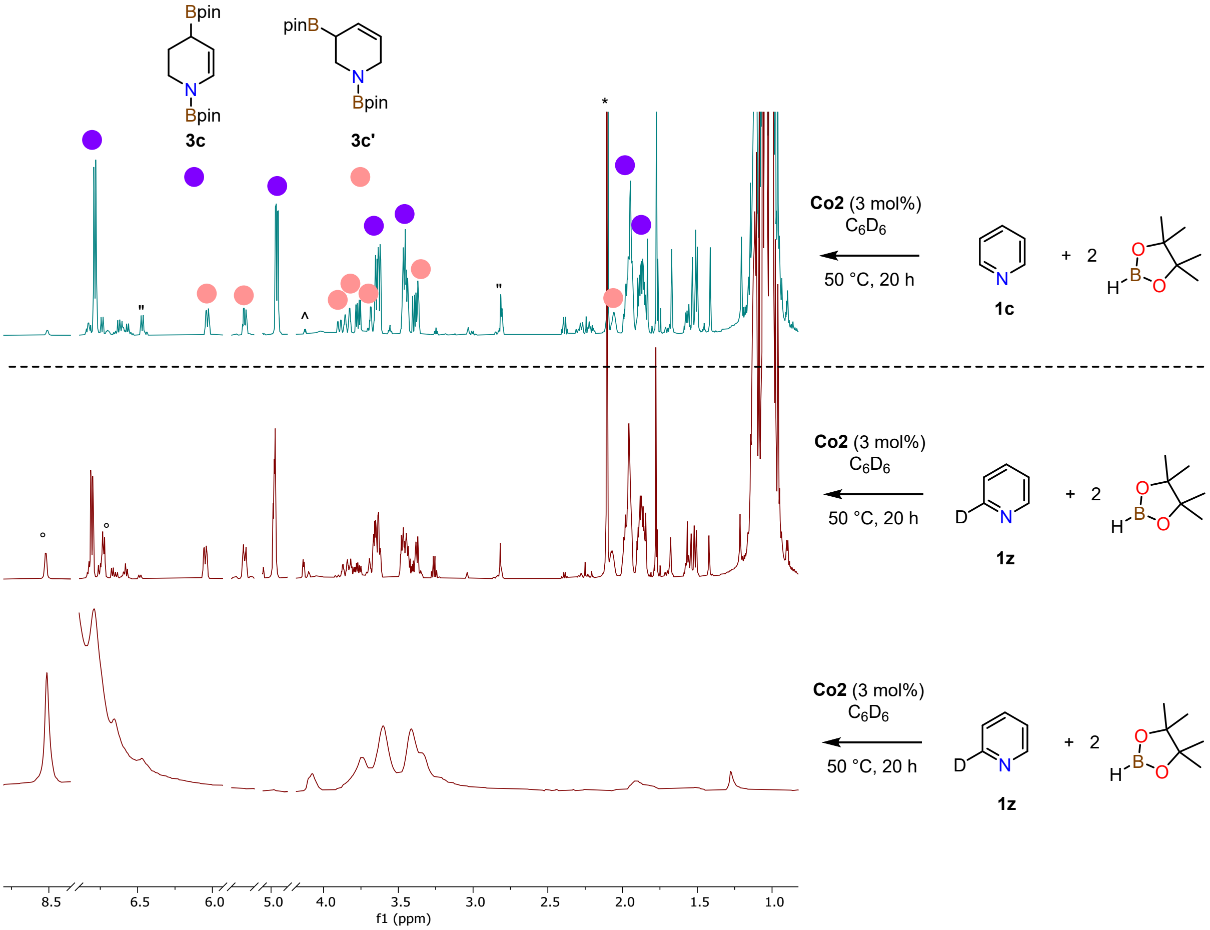

Scheme S36: Overlay of the ^{1}H- and ^{2}H-NMR spectra obtained after applying the reaction conditions on the right to either pyridine **1c** or pyridine-2-d **1z**. (°) Signals of **1z**. (^) Signals of the 1,2-DHP product. (") Signals of the 1,4-DHP product.

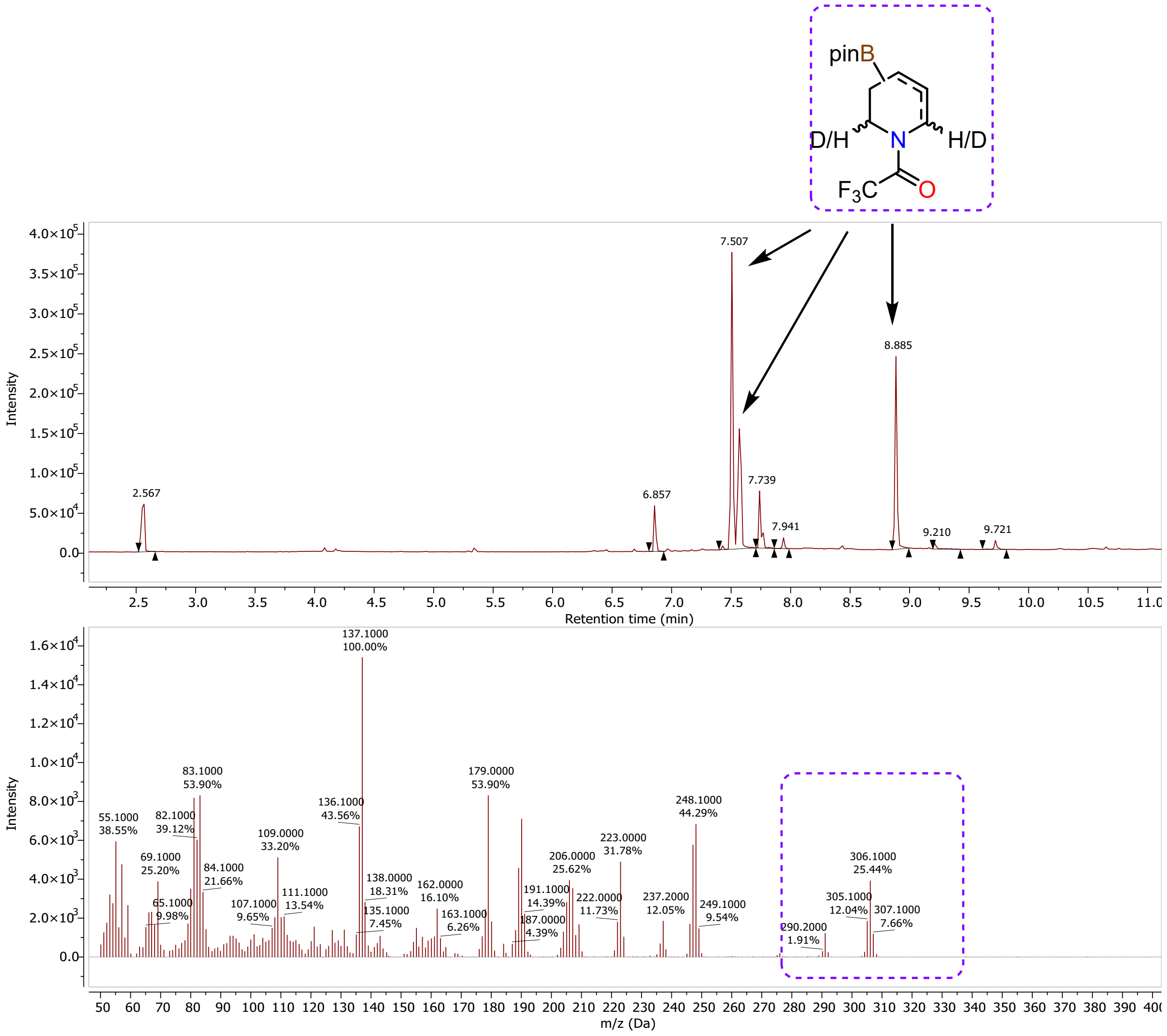

Scheme S37: GC-MS analysis of the crude mixture of pyridine-2-d **1z** hydroboration after TFAA treatment. At least three deuterated double hydroboration products were detected.

7.10 Control Experiments for Hidden Boron Catalysis

To eliminate the possibility of the formation of catalytically relevant borane species through the cobalt pyridonate-promoted decomposition of HBpin (hidden boron catalysis), we conducted a series of NMR scale control experiments guided by protocols of Thomas et al.[23,24] Mixtures of pinacolborane and **Co2** with/without substrate were subjected to reaction condition and analyzed by ^{11}B-NMR spectroscopy. Also, TMEDA was used as a trapping agent for *in situ* formed BH_3.

Inside a glovebox filled with argon, an oven-dried scintillation vial was charged with:

(1) HBpin (0.88 mmol, 128 µL), **Co2** (0.012 mmol, 5.0 mg) and C_6D_6 (0.5 mL).

(2) HBpin (0.88 mmol, 128 µL), **Co2** (0.012 mmol, 5.0 mg), TMEDA (0.036 mmol, 6.0 µL) and C_6D_6 (0.5 mL).

(3) HBpin (0.88 mmol, 128 µL), **Co2** (0.012 mmol, 5.0 mg), 4-methylpyridine (0.4 mmol, 39 µL), TMEDA (0.036 mmol, 6.0 µL) and C_6D_6 (0.5 mL).

The reaction mixtures were mixed with a Pasteur pipette before transferring them to J. Young NMR tubes. Subsequently, the NMR tubes were closed and taken out of the glovebox. The reaction mixtures were then heated to 50 °C for 20 h and afterwards immediately analysed by ^{11}B-NMR spectroscopy.

(1) ^{11}B-NMR spectrum shows no traces of BH_3 adducts (*lit. –12 ppm)*[24].

(2) ^{11}B-NMR spectrum shows absence of $(BH_3)_n$-TMEDA adducts. [*compare with authentic sample generated from BH_3-SMe_2 and TMEDA*].

(3) ^{11}B-NMR spectrum shows absence of $(BH_3)_n$-TMEDA adducts. [*compare with authentic sample generated from BH_3-SMe_2 and TMEDA*]. Note that both the single as well as the double hydroboration product is forming as indicated by the appearance of the respective signals at 33 ppm and 24 ppm.

(4) BH_3-SMe_2 and TMEDA were mixed in C_6D_6 (0.5 mL) and analysed by ^{11}B-NMR spectroscopy to generate the reference spectrum of $(BH_3)_n$-TMEDA adducts.

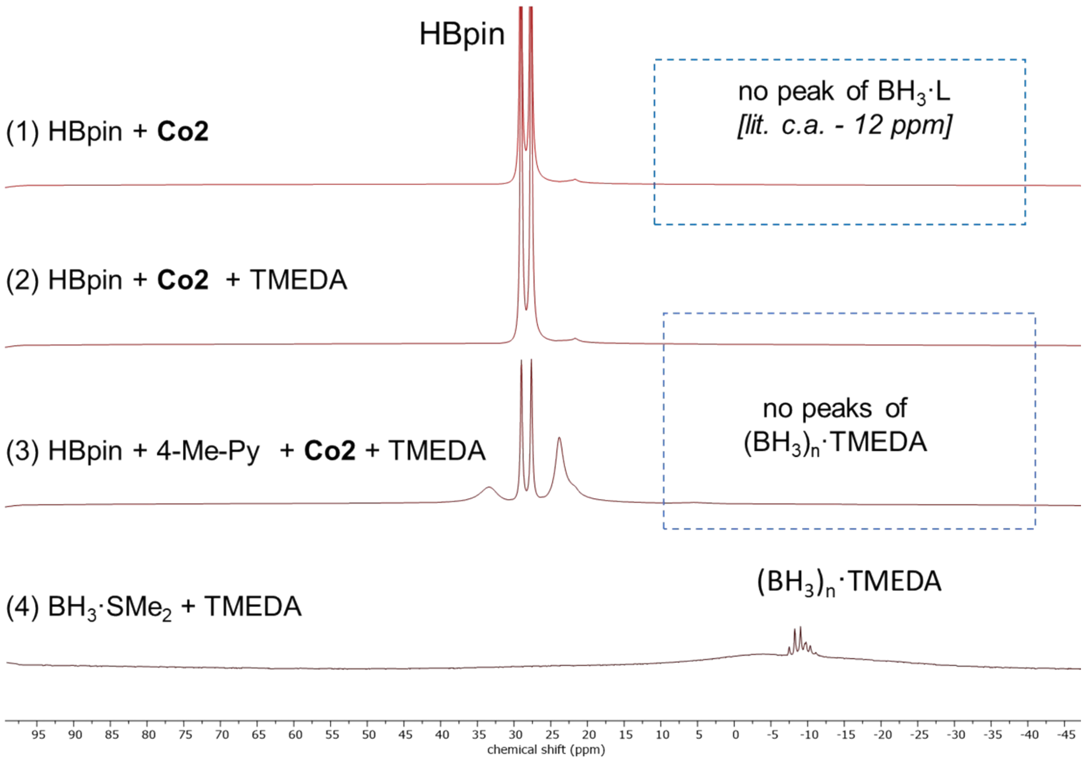

Scheme S38: Stacked ^{11}B-NMR spectra of control experiments conducted to rule out hidden borane catalysis.

8. Gram-Scale Sequential Double Hydroboration–*N*-Acetylation of 4-Methyl-pyridine

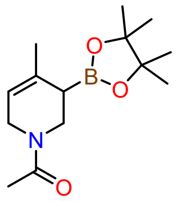

4a
$C_{14}H_{24}BNO_3$
265.16 g/mol

Inside a glovebox filled with argon, an oven-dried 100 mL Schlenk-round bottom flask was charged with precatalyst **Co2** (134 mg, 0.321 mmol, 3 mol%) and a magnetic stirring bar. The precatalyst was dissolved in toluene (18 mL, 0.6 M) and to the dark red solution was added 4-methylpyridine **1a** (1.07 mL, 10.8 mmol, 1.00 eq.). Then, HBpin (3.50 mL, 23.4 mmol, 2.17 eq.) was added in one portion. During the addition of HBpin the reaction mixture turned black. The Schlenk flask was taken out of the glovebox and stirred at 100 °C for 20 h. Subsequently, acetic anhydride (1.10 mL, 11.2 mmol, 1.08 eq.) was added and the reaction mixture was stirred for an additional 48 h. The dark brown solution was filtrated over a short pad of silica gel (Ø = 4 cm x h = 1 cm) and the residue was washed with EtOAc (80 mL). Afterwards, the dark brown filtrate was cocentrated *in vacuo* and the red-brown oily residue was purified by flash chromatography twice (SiO_2, EtOAc/pentane = 1:1 → 2:1, (V/V), $KMnO_4$/vanillin, Ø = 6 cm/2.5 cm x h = 11 cm/11.5 cm) to give allylic boronate **3a** (527 mg, 1.99 mmol, 18%) as a colourless oil.

For characterisation see section 4.2.

9. Chemical Diversification of Borylated *N*-Acetyl Tetrahydropyridines

1-(3-Hydroxy-4-methyl-3,6-dihydropyridin-1(2*H*)-yl)ethan-1-one 5

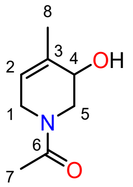

5

$C_8H_{13}NO_2$

155.20 g/mol

This reaction was performed under atmospheric conditions. The title compound was prepared following a procedure reported in literature.[25]

To a colourless solution of **4a** (53 mg, 0.20 mmol, 1.0 eq.) in THF/H_2O (1:1, 2 mL) was added $NaBO_3 \cdot 4H_2O$ (77 mg, 0.50 mmol, 2.5 eq.) at room temperature while stirring. The resulting suspension was stirred at ambient temperature for 3 h. After completion (TLC control, EtOAc/pentane = 2:1, $KMnO_4$), the reaction mixture was diluted with dest. H_2O (10 mL) and extracted with CH_2Cl_2 (4x10 mL). The combined organic layers were washed with brine (30 mL), dried over anhydrous Na_2SO_4, filtered, and concentrated *in vacuo*. Subsequently, the colourless crude oil was purified by flash chromatography (SiO_2, EtOAc → CH_2Cl_2 +5 % MeOH, (V/V), $KMnO_4$/vanillin, Ø = 1.5 cm x h = 10.5 cm) to give allylic alcohol **5** as a colourless oil (16.0 mg, 0.103 mmol, 52%).

***R*$_f$-value**: 0.10 (SiO_2, EtOAc, $KMnO_4$/vanillin).

Due to limited rotation around the C-N amide bond, the compound forms two stable rotamers in solution at room temperature. This gives rise to two different signal sets in the ^{1}H- and ^{13}C-NMR spectra. One rotamer is energetically preferred over the other. According to ^{1}H-NMR analysis, the ratio between the rotamers amounts to 67:33.

Major rotamer:

^{1}H-NMR (500.1 MHz, $CDCl_3$, 18.0 °C): δ [ppm] = 5.54–5.50 (m, 1 H, 2-H), 4.56–4.49 (m, 1 H, 1-$H^{a/b}$), 3.95–3.89 (br, m, 1 H, 4-H), 3.85–3.76 (overlap with minor rotamer, m, 1 H, 5-$H^{a/b}$), 3.49–3.41 (m, 1 H, 1-$H^{a/b}$), 3.36–3.28 (overlap with minor rotamer, m, 1 H, 5-$H^{a/b}$), 3.06 (overlap with minor rotamer, br, d, $^3J_{(H,H)}$ = 8.0 Hz, 4-O*H*), 2.14 (s, 3 H, 7-H), 1.84–1.81 (overlap with minor rotamer, m, 3 H, 8-H).

$^{13}C\{^1H\}$-NMR (125.8 MHz, $CDCl_3$, 21.2 °C): δ [ppm] = 170.5 (C-6), 134.4 (C-3), 122.3 (C-2), 67.7 (C-4), 50.9 (C-5), 41.7 (C-1), 21.5 (C-7), 20.4 (C-8).

Minor rotamer:

^1H-NMR (500.1 MHz, $CDCl_3$, 18.0 °C): δ [ppm] = 5.48–5.44 (m, 1 H, 2'-H), 4.12 (dd, $^2J_{(H,H)}$ = 13.4 Hz, $^3J_{(H,H)}$ = 4.0 Hz, 1 H, 5'-$H^{a/b}$), 4.05–3.95 (m, 2 H, 4'-H and 1'-$H^{a/b}$), 3.85–3.76 (overlap with major rotamer, m, 1 H, 1'-$H^{a/b}$), 3.36–3.28 (overlap with major rotamer, m, 1 H, 5'-$H^{a/b}$), 2.99 (br, s, 1 H, 4'-O*H*), 2.08 (s, 3 H, 7'-H), 1.84–1.81 (overlap with major rotamer, m, 3 H, 8'-H).

$^{13}C\{^1H\}$-NMR (125.8 MHz, $CDCl_3$, 21.2 °C): δ [ppm] = 170.6 (C-6'), 136.3 (C-3'), 120.1 (C-2'), 67.0 (C-4'), 45.8 (C-1'/C-5'), 45.7 (C-1'/C-5'), 21.8 (C-7'), 20.1 (C-8').

HRMS-ESI (*m/z*): calc. for $C_8H_{13}NNaO_2^+$ [M+Na]$^+$: 178.0838, found: 178.0839.

1-(3-(Hydroxy(phenyl)methyl)-4-methyl-3,6-dihydropyridin-1(2H)-yl)ethan-1-one 6

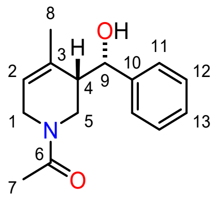

6

$C_{15}H_{19}NO_2$

245.32 g/mol

The title compound was prepared following a procedure reported in literature.[26]

Inside a glovebox filled with argon, an oven-dried headspace vial (5 mL) was charged with **4a** (53 mg, 0.20 mmol, 1.0 eq.) and a magnetic stirring bar. After dissolving the allylic boronate **4a** in toluene (0.4 mL) the vial was closed and taken out of the glovebox. Through the septum of the cap was added benzaldehyde (41 µL, 0.40 mmol, 2.0 eq.) at ambient temperature while stirring. Subsequently, the colourless reaction mixture was stirred at 50 °C for 17 h. After completion (TLC control, EtOAc/pentane = 1:1, UV/$KMnO_4$), the yellow reaction mixture was diluted with CH_2Cl_2 (1 mL) and quenched with an aqueous triethanolamine solution (10 w% in H_2O, 1 mL). Once the mixture was stirred for 10 min, the layers were separated (addition of 2 mL H_2O and 2 mL CH_2Cl_2 for better phase separation) and the aqueous phase was extracted using CH_2Cl_2 (3x3 mL). The combined organic layers were dried over anhydrous Na_2SO_4, filtered and the solvent was removed under reduced pressure. Subsequently, the colourless crude oil was purified by flash chromatography (SiO_2, EtOAc/pentane = 2:1 → 3:1, (V/V),

$KMnO_4$/vanillin/UV, Ø = 1.5 cm x h = 12 cm) to give target compound **6** (37 mg, 0.15 mmol, 75%) as a colourless oil which crystallises on standing. Suitable single crystals for X-ray crystal structure analysis were obtained by recrystallisation from toluene (400 µL, dissolved at 60 °C) at –30 °C.

***R*$_f$-value**: 0.23 (SiO_2, EtOAc/pentane = 3:1, (V/V), $KMnO_4$/vanillin/UV).

Due to limited rotation around the C-N amide bond, the compound forms two stable rotamers in solution at room temperature. This gives rise to two different signal sets in the ^{1}H- and ^{13}C-NMR spectra. One rotamer is energetically preferred over the other. According to ^{1}H-NMR analysis, the ratio between the rotamers amounts to 53:47.

Major rotamer:

^{1}H-NMR (400.1 MHz, $CDCl_3$, 26.4 °C): δ [ppm] = 7.37–7.22 (overlap with minor rotamer, m, 5 H, Ar-H), 5.46–5.40 (m, 1 H, 2-H), 4.51 (d, $^3J_{(H,H)}$ = 8.3 Hz, 9-H), 4.45–4.36 (m, 1 H, 1-H$^{a/b}$), 4.18 (dd, $^2J_{(H,H)}$ = 12.9 Hz, $^3J_{(H,H)}$ = 2.3 Hz, 1 H, 5-H$^{a/b}$), 3.60–3.48 (m, 1 H, 1-H$^{a/b}$), 3.08 (dd, $^2J_{(H,H)}$ = 12.9 Hz, $^3J_{(H,H)}$ = 3.0 Hz, 1 H, 5-H$^{a/b}$), 2.85–2.70 (overlap with minor rotamer, m, 1 H, 9-O*H*), 2.36–2.26 (overlap with minor rotamer, m, 1 H, 4-H), 2.14 (s, 3 H, 7-H), 1.10–1.03 (m, 3 H, 8-H).

^{13}C{^{1}H}-NMR (100.6 MHz, $CDCl_3$, 26.4 °C): δ [ppm] = 170.9 (overlap with minor rotamer, br, C-6), 143.4 (C-10), 133.1 (C-3), 128.6 (2 C, C-11/C-12), 128.0 (C-13), 126.7 (2 C, C-11/C-12), 121.5 (C-2), 73.6 (C-9), 48.1 (C-4), 44.6 (C-5), 42.3 (C-1), 22.7 (C-8), 21.6 (C-7).

Minor rotamer:

^{1}H-NMR (400.1 MHz, $CDCl_3$, 26.4 °C): δ [ppm] = 7.37–7.22 (overlap with major rotamer, m, 5 H, Ar'-H), 5.38–5.32 (m, 1 H, 2'-H), 4.74 (br, d, $^2J_{(H,H)}$ = 13.2 Hz, 5'-H$^{a/b}$), 4.31 (br, d, $^3J_{(H,H)}$ = 7.9 Hz, 9'-H), 4.07–3.93 (m, 2 H, 1'-H$^{a/b}$ and 9'-O*H*), 3.90–3.80 (m, 1 H, 1'-H$^{a/b}$), 2.85–2.70 (overlap with major rotamer, m, 1 H, 5'-H$^{a/b}$), 2.36–2.26 (overlap with major rotamer, m, 1 H, 4'-H), 2.10 (s, 3 H, 7'-H), 1.19–1.13 (m, 3 H, 8'-H).

^{13}C{^{1}H}-NMR (100.6 MHz, $CDCl_3$, 26.4 °C): δ [ppm] = 170.9 (overlap with major rotamer, br, C-6'), 142.7 (C-10'), 135.9 (C-3'), 128.4 (2 C, C-11'/C-12'), 127.7 (C-13'), 127.0 (2 C, C-11'/C-12'), 119.5 (C-2'), 74.8 (C-9'), 48.2 (C-4'), 46.4 (C-1'), 41.0 (C-5'), 22.9 (C-8'), 21.5 (C-7').

HRMS-ESI (*m/z*): calc. for $C_{15}H_{19}NNaO_2^+$ [M+Na]$^+$: 268.1308, found: 268.1313.

1-(4-Methyl-3-phenyl-3,6-dihydropyridin-1(2*H*)-yl)ethan-1-one 7

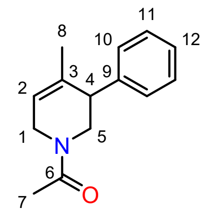

7
$C_{14}H_{17}NO$
215.30 g/mol

The title compound was prepared following a procedure reported in literature.[27,28]

Inside a glovebox filled with argon, a flame-dried Schlenk finger was charged with **4a** (50 mg, 0.19 mmol, 1.0 eq.), Pd_2dba_3 (5.2 mg, 5.7 µmol, 3 mol%), PPh_3 (33 mg, 0.13 mmol, 0.67 eq.), Ag_2O (44 mg, 0.19 mml, 1.0 eq.) and iodobenzene (23 µL, 0.21 mmol, 1.1 eq.). The substances were suspended in THF (2.7 mL), the Schlenk finger was closed and taken out of the glovebox. Then, the reaction mixture was stirred at 70 °C for 22 h. The black suspension was filtered over Celite® (~1 cm, elution with EtOAc), the filtrate was concentrated under reduced pressure and the dark viscous residue was purified by flash chromatography (SiO_2, pentane/EtOAc = 5:1 → 1:2, (V/V), $KMnO_4$/UV, Ø = 3 cm x h = 9 cm) to give target compound **7** (14 mg, 0.65 µmol, 34%) as a slightly yellow oil.

***R*f-value**: 0.24 (SiO_2, EtOAc/pentane = 1:1, (V/V), $KMnO_4$/UV).

Due to limited rotation around the C-N amide bond, the compound forms two stable rotamers in solution at room temperature. This gives rise to two different signal sets in the ^{1}H- and ^{13}C-NMR spectra. One rotamer is energetically preferred over the other. According to ^{1}H-NMR analysis, the ratio between the rotamers amounts to 81:19.

Major rotamer:

^{1}H-NMR (600.1 MHz, $CDCl_3$, 24.9 °C): δ [ppm] = 7.32–7.27 (overlap with minor rotamer, m, 2 H, 11-H), 7.27–7.19 (overlap with minor rotamer, m, 1 H, 12-H), 7.16–7.12 (overlap with minor rotamer, m, 2 H, 10-H), 5.68–5.65 (m, 1 H, 2-H), 4.72–4.65 (m, 1 H, 1-$H^{a/b}$), 3.65–3.56 (m, 3 H, 1-$H^{a/b}$, 5-H^a and 5-H^b), 3.33–3.30 (m, 1 H, 4-H), 1.63–1.61 (m, 3 H, 8-H), 1.50 (s, 3 H, 7-H).

^{13}C{^{1}H}-NMR (150.9 MHz, $CDCl_3$, 24.9 °C): δ [ppm] = 169.9 (C-6), 141.0 (C-9), 133.8 (C-3), 128.8 (C-11), 128.16 (C-10), 127.2 (C-12), 120.8 (C-2), 50.9 (C-5), 46.8 (C-4), 42.0 (C-1), 22.0 (C-8), 20.8 (C-7).

Minor rotamer:

^{1}H-NMR (600.1 MHz, $CDCl_3$, 24.9 °C): δ [ppm] = 7.32–7.27 (overlap with major rotamer, m, 2 H, 11'-H), 7.27–7.19 (overlap with major rotamer, m, 1 H, 12'-H), 7.16–7.12 (overlap with major rotamer, m, 2 H, 10'-H), 5.61–5.58 (m, 1 H, 2'-H), 4.08–4.02 (m, 1 H, 1'-$H^{a/b}$), 3.99–3.94 (m, 1 H, 1'-$H^{a/b}$), 3.83 (dd, $^2J_{(H,H)}$ = 13.0 Hz, $^3J_{(H,H)}$ = 5.0 Hz, 1 H, 5'-$H^{a/b}$), 3.71 (dd, $^2J_{(H,H)}$ = 13.0 Hz, $^3J_{(H,H)}$ = 5.9 Hz, 1 H, 5'-$H^{a/b}$), 3.37–3.33 (m, 1 H, 4'-H), 2.08 (s, 3 H, 7'-H), 1.59–1.57 (m, 3 H, 8'-H).

$^{13}C\{^1H\}$-NMR (150.9 MHz, $CDCl_3$, 24.9 °C): δ [ppm] = 169.5 (C-6'), 141.2 (C-9'), 136.2 (C-3'), 128.6 (C-11'), 128.23 (C-10'), 127.0 (C-12'), 119.2 (C-2'), 45.94 (C-4'), 45.86 (C-1'/5'), 45.8 (C-1'/5'), 21.9 (C-7'), 21.8 (C-8').

HRMS-ESI (*m/z*): calc. for $C_{14}H_{18}NO^+$ $[M+H]^+$: 216.1383, found: 268.1379.

After the first chromatographic purification, the product is slightly contaminated with minimal amounts of PPh_3/$O{=}PPh_3$.

10. Other Procedures and Characterisation Data

10.1 General procedure for the preparation of GC-MS samples

The respective reaction mixtures were quenched using an aqueous $NaHCO_3$-solution (10%, 1 mL) and diluted with EtOAc (1 mL). After extraction, the layers were separated and the organic phase was washed with brine (1 mL), dried over anhydrous Na_2SO_4 and eluted from a short pad of silica gel using EtOAc (3 mL). These stock solutions were then transferred into a MS vial (1.5 mL) in which they were further diluted 1:1 with EtOAc before injecting them into the GC-MS device.

10.2 Acylation of *N*-Boryl Double Hydroboration Products with TFAA for GC-MS Analysis

To the J. Young NMR tube containing the double hydroboration crude mixture was added directly and under argon purge trifluoroacetic anhydride (31 µL, 0.22 mmol, 1.1 eq.). The NMR tube was closed, shaken vigorously for 30 second and then left at room temperature for 5 hours. The reaction mixture was then prepared for GC-MS analysis according to the general procedure described in 10.1.

10.3 Protodeboronation of 3b

4-Phenyl-1,2,3,6-tetrahydropyridine 8[29,30]

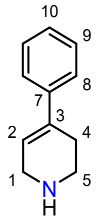

7

$C_{11}H_{13}N$

159.23 g/mol

Inside a glovebox filled with argon, an oven-dried headspace vial (5 mL) was charged with precatalyst **Co2** (6.3 mg, 15 µmol, 3 mol%), 4-phenylpyridine **1b** (78 mg, 0.50 mmol, 1.0 eq.) and a magnetic stirring bar. The solids were dissolved in C_6D_6 (1.7 mL, 0.3 M) and to the red solution was added pinacolborane (160 µL, 1.07 mmol, 2.14 eq.). During the addition of HBpin the reaction mixture turned black. The headspace vial was closed, taken out of the glovebox, and stirred at 50 °C for 20 h. After completion, the vial was opened and an aqueous

triethanolamine solution (10 w% in H_2O, 6.0 mL, 4.0 mmol, 8.0 eq.) was added. Then, the reaction mixture was stirred at 40 °C for 2 hours (under air). The mixture was extracted with CH_2Cl_2 (3x6 mL) and the combined organic layers were dried over anhydrous Na_2SO_4, filtered, and concentrated under reduced pressure. The resulting dark oil was purified by flash chromatography (SiO_2, EtOAc +2 % diethylamine, (V/V), UV/$KMnO_4$, Ø = 1.5 cm x h = 15 cm) to give the target compound **8** (20.0 mg, 0.126 mmol, 25%) as a slightly red oil.

^{1}H-NMR (500.1 MHz, $CDCl_3$, 21.6 °C): δ[ppm] = 7.41–7.36 (m, 2 H, 8/9-H), 7.35–7.29 (m, 2 H, 8/9-H), 7.27–7.21 (m, 1 H, 10-H), 6.15–6.11 (m, 1 H, 2-H), 3.58–3.50 (m, 2 H, 1-H), 3.12 (t, $^3J_{(H,H)}$ = 5.6 Hz, 2 H, 5-H), 2.50–2.44 (m, 2 H, 4-H), 2.27 (br, s, 1 H, N-*H*).

^{13}C{^{1}H}-NMR (125.8 MHz, $CDCl_3$, 22.4 °C): δ[ppm] = 141.5 (C-7), 135.4 (C-3), 128.4 (2 C, C-8/9), 127.1 (C-10), 124.9 (2 C, C-8/9), 123.5 (C-2), 45.6 (C-1/5), 43.4 (C-1/5), 27.8 (C-4).

GC-MS (EI): *m/z* (%): 159.1 (100) $[M]^{\bullet+}$, 130.1 (54.7) $[M–CH_3N]^+$, 115.0 (43.4), 91.1 (27.5), 82.1 (41.5) $[M–C_6H_5]^+$, 77.1 (14.9) $[M–C_5H_8N]^+$.

10.4 *In situ* Generation of *N*-boryl 4-Phenyl-1,2,3,6-tetrahydropyridine 3b’

4-Phenyl-1-(4,4,5,5-tetramethyl-1,3,2-dioxaborolan-2-yl)-1,2,3,6-tetrahydropyridine 3b’

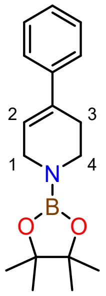

3b'
$C_{17}H_{24}BNO_2$
285.19 g/mol

Inside a glovebox filled with argon, tetrahydropyridine **7** (9.0 mg, 57 µmol, 1.0 eq.) was dissolved in C_6D_6 (0.6 mL) and treated with pinacolborane (8.9 µL, 60 µmol, 1.05 eq.). The reaction mixture was transferred into a screw-cap NMR tube and immediately analysed by ^{1}H-NMR spectroscopy.

^{1}H-NMR (300.2 MHz, C_6D_6, 22.9 °C): δ[ppm] = 7.25–7.00 (m, 5 H, Ar-*H*), 5.91–5.84 (m, 1 H, 2-H), 3.95–3.87 (m, 2 H, 1-H), 3.39 (t, $^3J_{(H,H)}$ = 5.6 Hz, 2 H, 4-H), 2.33–2.23 (m, 2 H, 3-H), 1.14 (s, 12 H, Bpin).

10.5 Characterisation Data for Isolated *N*-Acetyl 4-Phenyl-1,2,3,6-Tetrahydropyridine 4b’

1-(4-Phenyl-3,6-dihydropyridin-1(2*H*)-yl)ethan-1-one 4b’

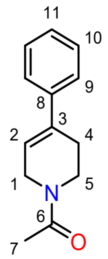

4b'
$C_{13}H_{15}NO$
201.27 g/mol

This compound was isolated as a byproduct of the sequential double hydroboration–*N*-acetylation of 4-phenylpyridine **1b** (procedure 3.4). It elutes just after **4b**.

Due to limited rotation around the C-N amide bond, the compound forms two stable rotamers in solution at room temperature. This gives rise to two different signal sets in the ^{1}H- and ^{13}C-NMR spectra. One rotamer is energetically preferred over the other. According to ^{1}H-NMR analysis, the ratio between the rotamers amounts to 53:47.

Major rotamer:

^{1}H-NMR (400.1 MHz, $CDCl_3$, 26.4 °C): δ [ppm] = 7.40–7.31 (overlap with minor rotamer, m, 4 H, 9-H and 10-H), 7.30–7.24 (overlap with minor rotamer, m, 1 H, 11-H), 6.08 (tt, $^{3}J_{(H,H)}$ = 3.4 Hz, $^{4}J_{(H,H)}$ = 1.5 Hz, 1 H, 2-H), 4.24 (pseudo-q, $^{3/4}J_{(H,H)}$ = 2.9 Hz, 2 H, 1-H), 3.67 (t, $^{3}J_{(H,H)}$ = 5.7 Hz, 2 H, 5-H), 2.63–2.52 (overlap with minor rotamer, m, 2 H, 4-H), 2.17 (s, 3 H, 7-H).

^{13}C{^{1}H}-NMR (100.6 MHz, $CDCl_3$, 26.4 °C): δ [ppm] = 169.4 (C-6), 140.42 (C-8), 135.0 (C-3), 128.61 (2 C, C-9/10), 127.5 (C-11), 125.05 (2 C, C-9/10), 121.2 (C-2), 43.5 (C-1/5), 42.3 (C-1/5), 28.1 (C-4), 21.6 (C-7).

Minor rotamer:

^{1}H-NMR (400.1 MHz, $CDCl_3$, 26.4 °C): δ [ppm] = 7.40–7.31 (overlap with major rotamer, m, 4 H, 9’-H and 10’-H), 7.30–7.24 (overlap with major rotamer, m, 1 H, 11’-H), 6.01 (tt, $^{3}J_{(H,H)}$ = 3.4 Hz, $^{4}J_{(H,H)}$ = 1.5 Hz, 1 H, 2’-H), 4.13 (pseudo-q, $^{3/4}J_{(H,H)}$ = 2.8 Hz, 2 H, 1’-H), 3.82 (t, $^{3}J_{(H,H)}$ = 5.8 Hz, 2 H, 5’-H), 2.63–2.52 (overlap with major rotamer, m, 2 H, 4’-H), 2.14 (s, 3 H, 7’-H).

^{13}C{^{1}H}-NMR (100.6 MHz, $CDCl_3$, 26.4 °C): δ[ppm] = 169.5 (C-6’), 140.39 (C-8’), 137.0 (C-3’), 128.63 (2 C, C-9’/10’), 127.7 (C-11’), 125.08 (2 C, C-9’/10’), 119.5 (C-2’), 46.0 (C-1’/5’), 38.4 (C-1’/5’), 27.3 (C-4’), 22.0 (C-7’).

HRMS-ESI (*m/z*): calc. for $C_{13}H_{16}NO^+$ [M+H]$^+$: 202.1226, found: 202.1219.

10.6 Synthesis of DBpin

The preparative procedure for the synthesis of DBpin was developed based on a previous report.[31]

A mixture of HBpin (69 mmol, 10 ml), C_6D_6 (69 mmol, 6.1 ml) and $Co[N(SiMe_3)_2]_2$ (5 mol% 3.5 mmol; 1.31 g) was stirred at room temperature and the H/D exchange progress was monitored by ^{11}B-NMR spectroscopy. The obtained crude DBpin solution was purified by static vacuum distillation and the concentration was determined by ^{1}H-NMR using hexamethylbenzene as an internal standard.

10.7 Synthesis of Pyridine-2-d 1z

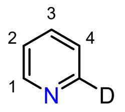

1z
C_5H_4DN
80.10 g/mol

The synthesis was performed according to a protocol of B. Liu et al.[32]

In a dried flame-dried Schlenk flask was dissolved under nitrogen atmosphere 2-bromopyridine (20 g, 12 ml, 0.13 mol,1.0 eq.) in diethyl ether (Et_2O, 100 mL). The solution was cooled to –90°C and *n*-butyllithium (1.6 M solution in hexane, 100 mL, 0.16 mol, 1.2 eq.) was added dropwise while stirring at –90 °C for 2 hours. The reaction mixture was allowed to warm up to room temperature and to the resulting dark red solution was added D_2O (20 g, 12 mL, 1.0 mol, 7.6 eq.). Subsequently, the solution was stirred for an additional 16 h at room temperature. A mixture of concentrated aqueous HCl (37%, 40 ml) and dest. H_2O (40 ml) was added and the aqueous layer was separated. The organic phase was washed with dest. H_2O (2x20 ml) and to the combined aqueous layers was added potassium hydroxide (KOH, 15 g) while cooling to 0 °C. The deuterated pyridine was extracted from the aqueous layer with Et_2O (5x20 ml). The organic phases were dried over KOH powder and the solvent was removed under reduced

pressure (830 mbar, 37 °C). Pyridine-d_2 was obtained by distillation (1 bar, 104 °C) as a colourless oil with >97% deuterium incorporation (9.6 g, 0.12 mol, 92%).

^{1}H-NMR (300.1 MHz, $CDCl_3$, 26.5 °C): δ [ppm] = 8.63–8.56 (m, 1 H, 1-H), 7.69–7.60 (m, 1 H, 3-H), 7.29–7.22 (overlap with solvent signal, m, 2 H, 2-H and 4-H).

^{1}H-NMR (600.1 MHz, C_6D_6, 26.5 °C): δ [ppm] = 8.41-8.40 (dd, J = 5.8, 1.8 Hz, 1H), 7.43 (td, J = 7.7, 1.9 Hz, 1H), 7.05 (dd, *J* = 7.7, 4.2 Hz, 2H).

^{2}H-NMR (92 MHz, C_6D_6) δ [ppm] = 8.52.

10.8 Preparation of Internal Standard Stock Solutions

On an analytical balance, a volumetric flask (4 mL) was charged with hexamethylbenzene (259.6 mg, 1.600 mmol) or 1,3,5-trimethoxybenzene (269.1 mg, 1.600 mmol). The volumetric flask was transferred into a glovebox filled with argon and the respective solids were dissolved in 4 mL of anhydrous C_6D_6 to give a 0.4 M stock solution. The prepared stock solution was then transferred into an oven-dried headspace vial (5 mL), the vial was closed, and the solution was stored at –30 °C under argon atmosphere. Prior to withdrawing a given volume of the stock solution, the solution was allowed to warm up to room temperature and the vial was pressurised with argon in the amount of the desired withdrawal volume.

11. Experimental Spectra

11.1 Experimental Spectra of Pyridone Ligands and their Complexes

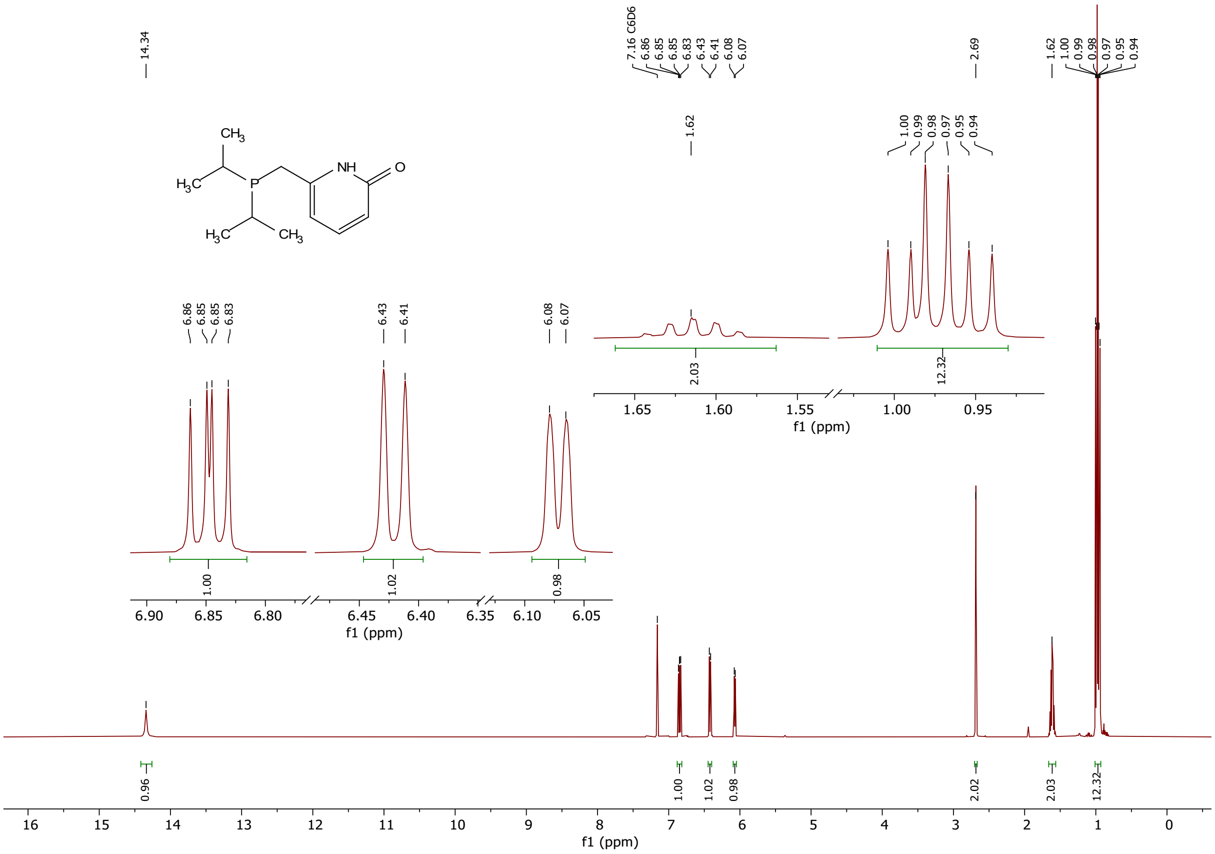

Figure 1: 500 MHz ^{1}H-NMR spectrum of pyridone ligand **L1H** in C_6D_6.

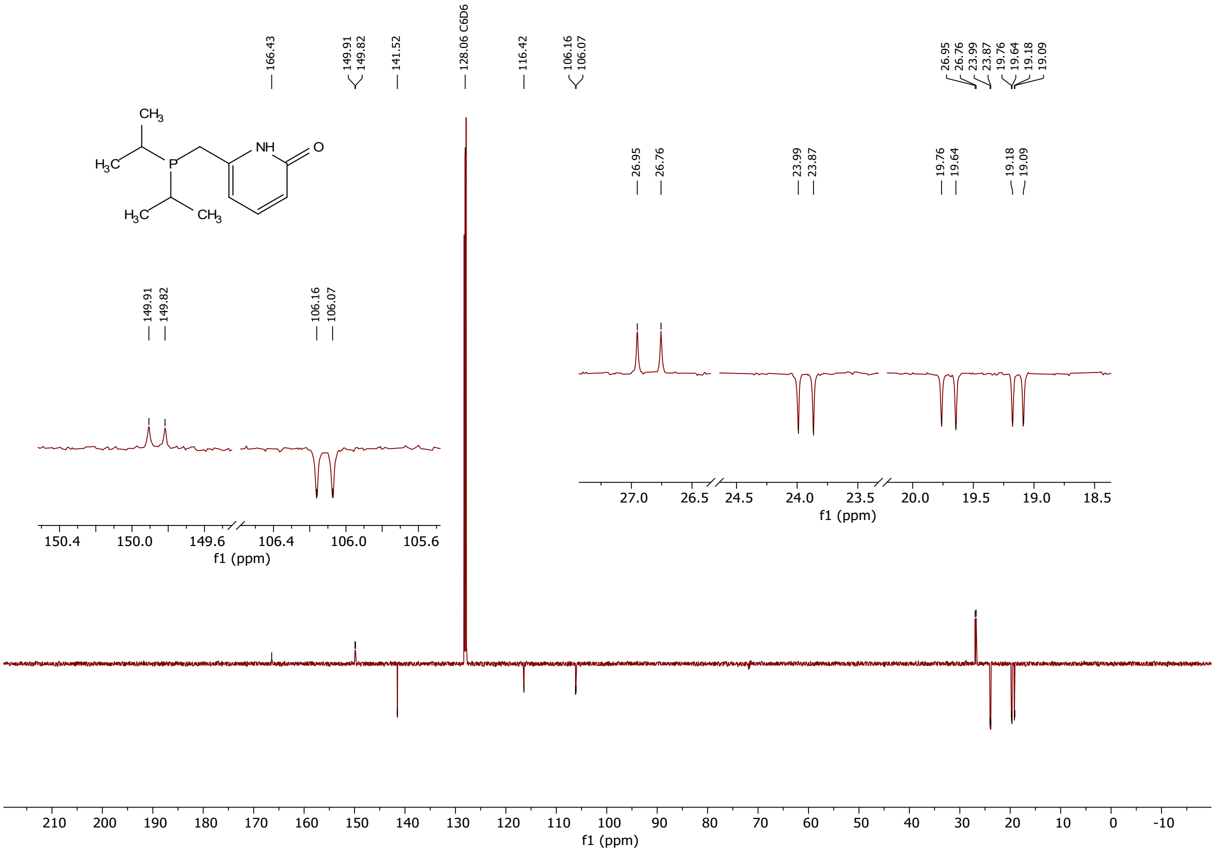

Figure 2: 126 MHz ^{13}C-DEPTQ-NMR spectrum of pyridone ligand **L1H** in C_6D_6.

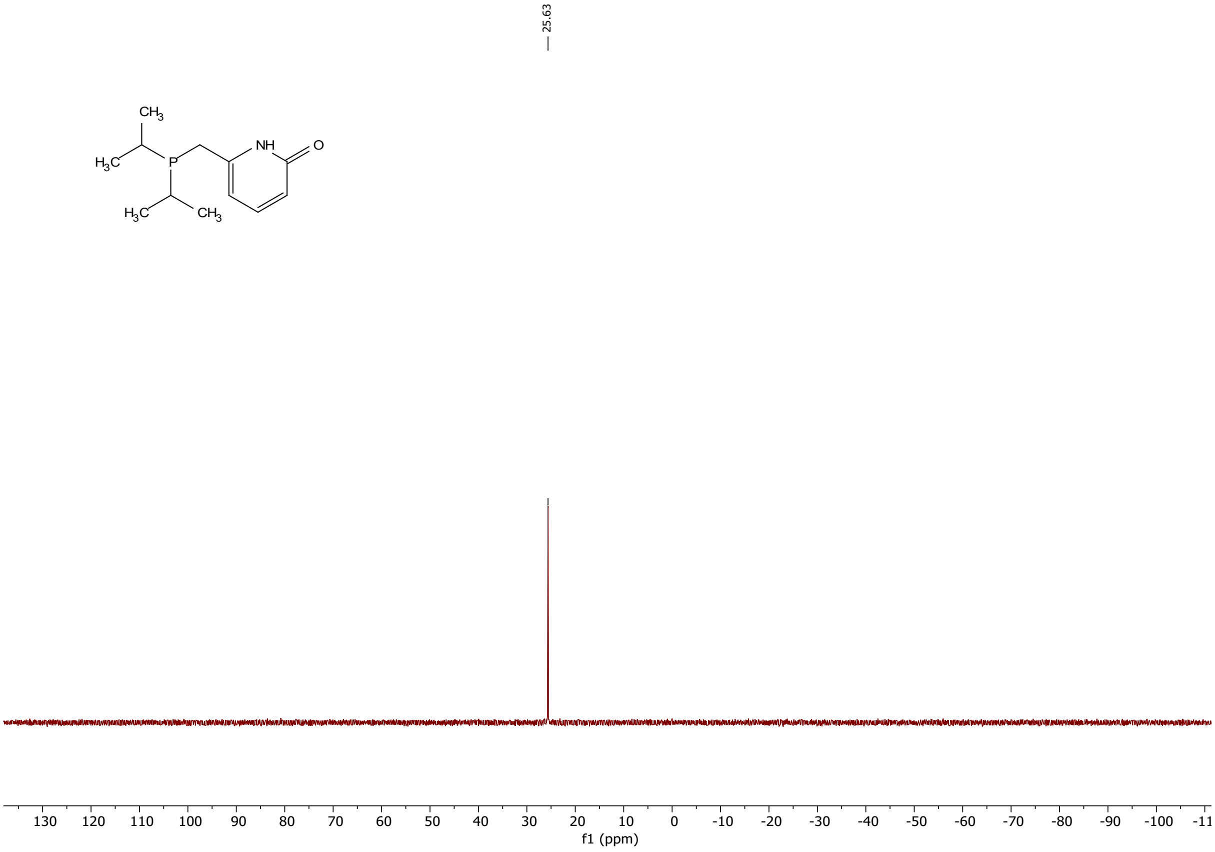

Figure 3: 203 MHz $^{31}P\{^1H\}$-NMR spectrum of pyridone ligand **L1H** in C_6D_6.

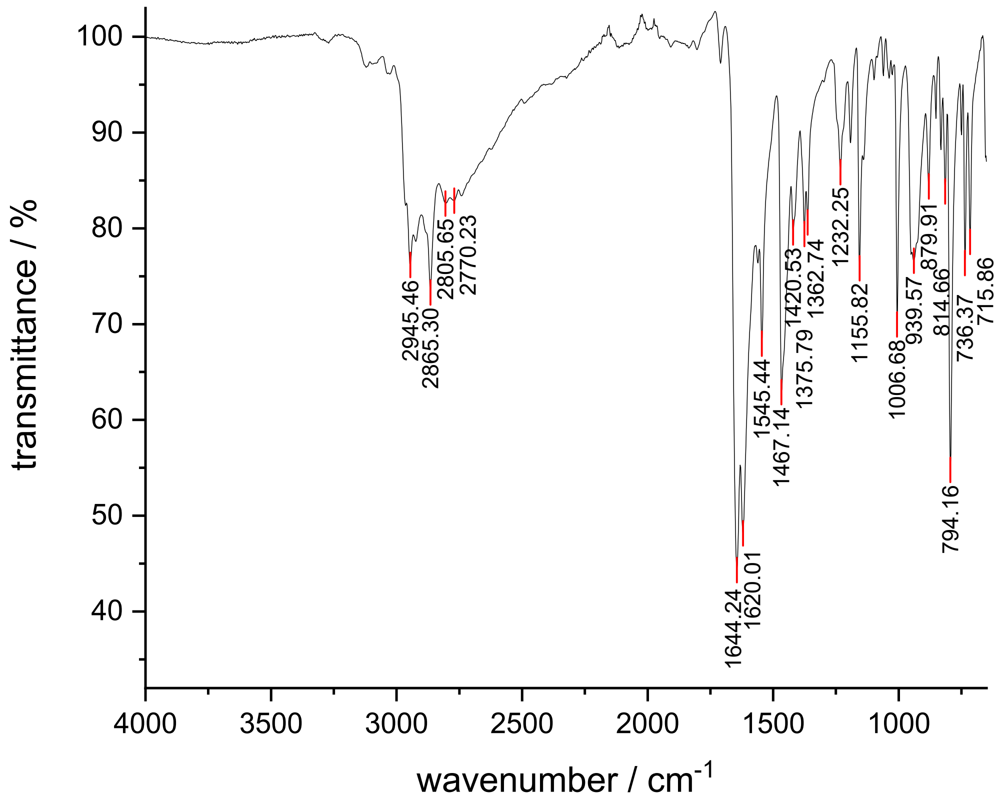

Figure 4: FT-IR spectrum of pyridone ligand **L1H**.

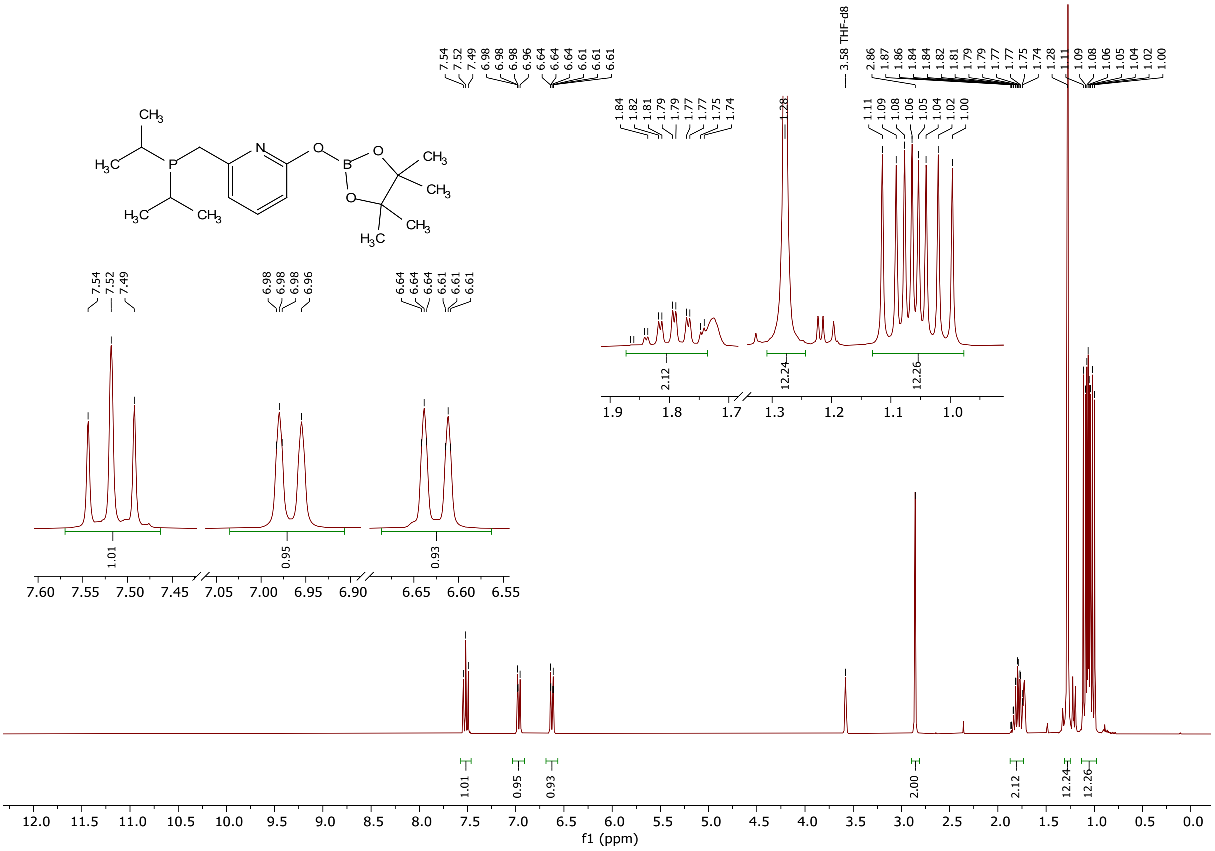

Figure 5: 300 MHz ^{1}H-NMR spectrum of **L1Bpin** in THF-d_8.

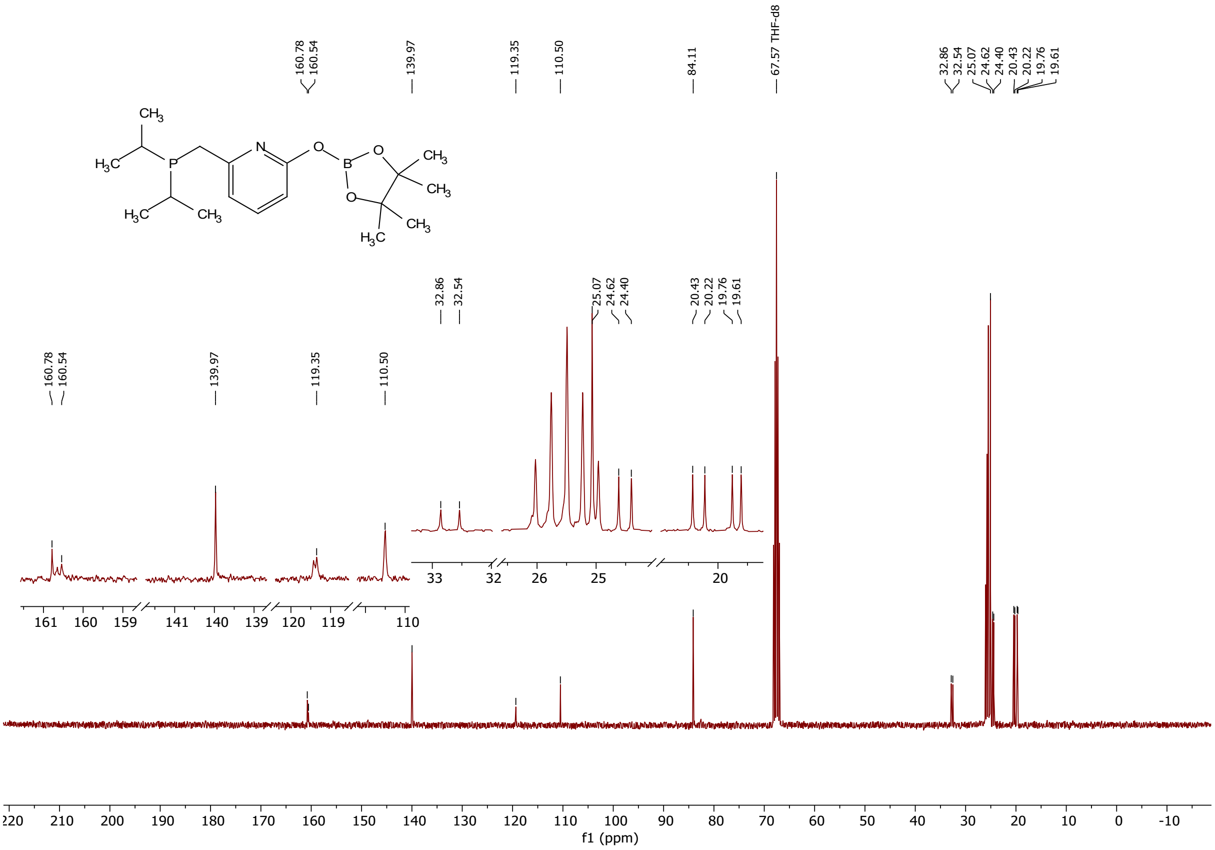

Figure 6: 76 MHz $^{13}C\{^{1}H\}$-NMR spectrum of **L1Bpin** in THF-d_8.

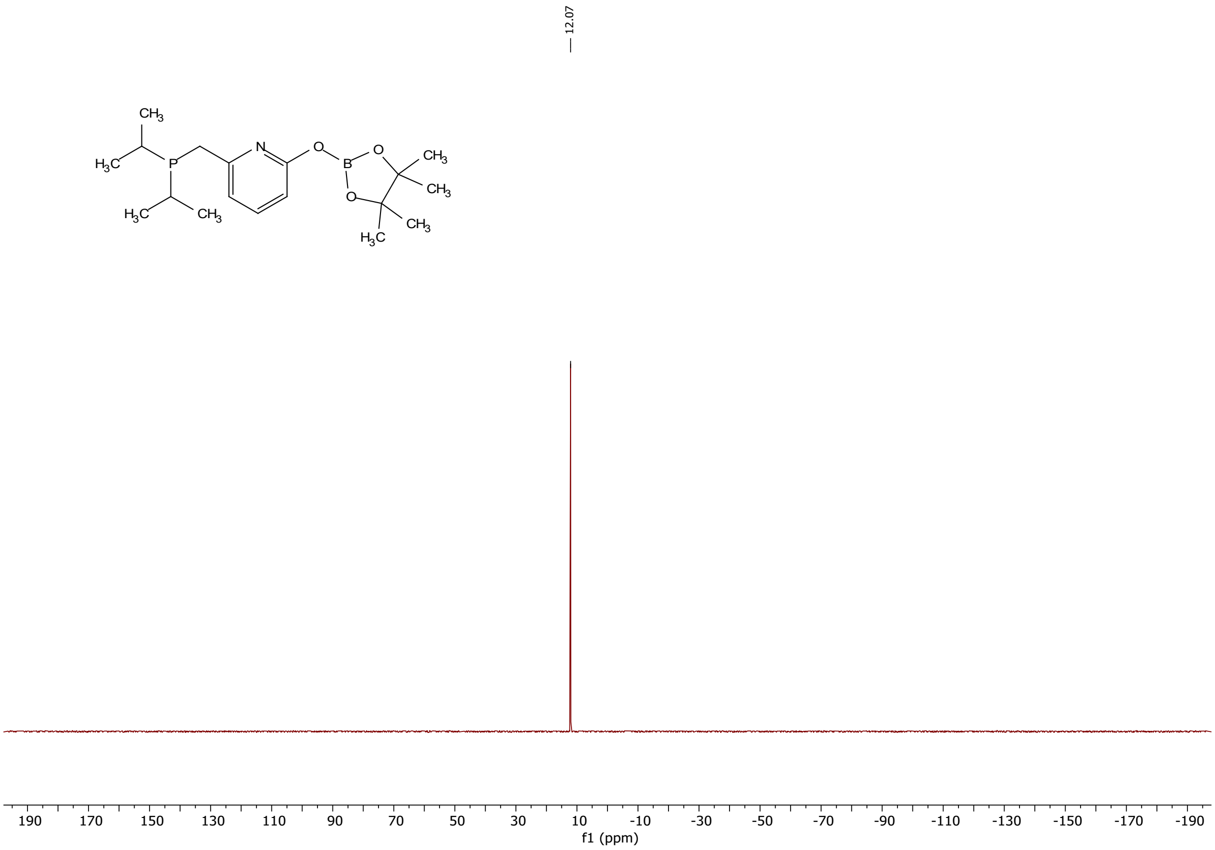

Figure 7: 162 MHz $^{31}P\{^{1}H\}$-NMR spectrum of **L1Bpin** in THF-d_8.

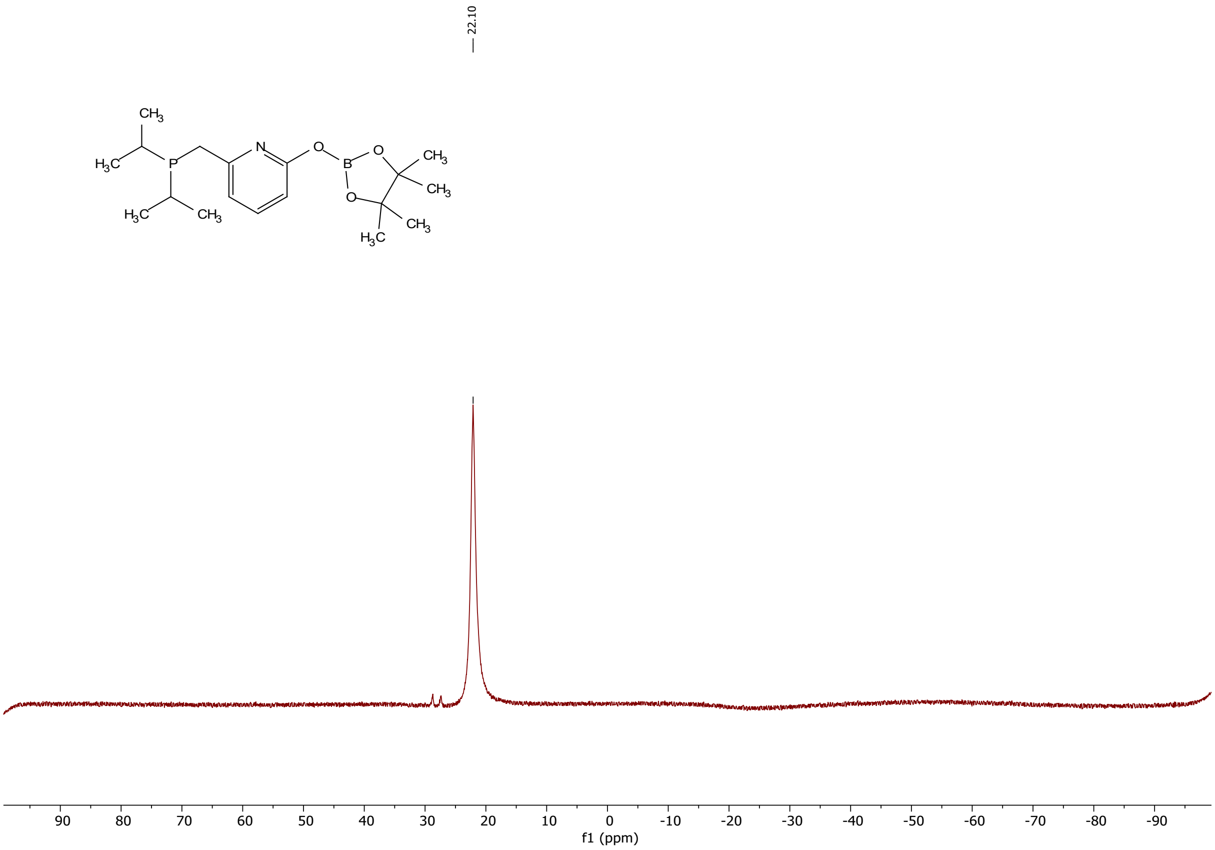

Figure 8: 128 MHz ^{11}B-NMR spectrum of **L1Bpin** in THF-d_8.

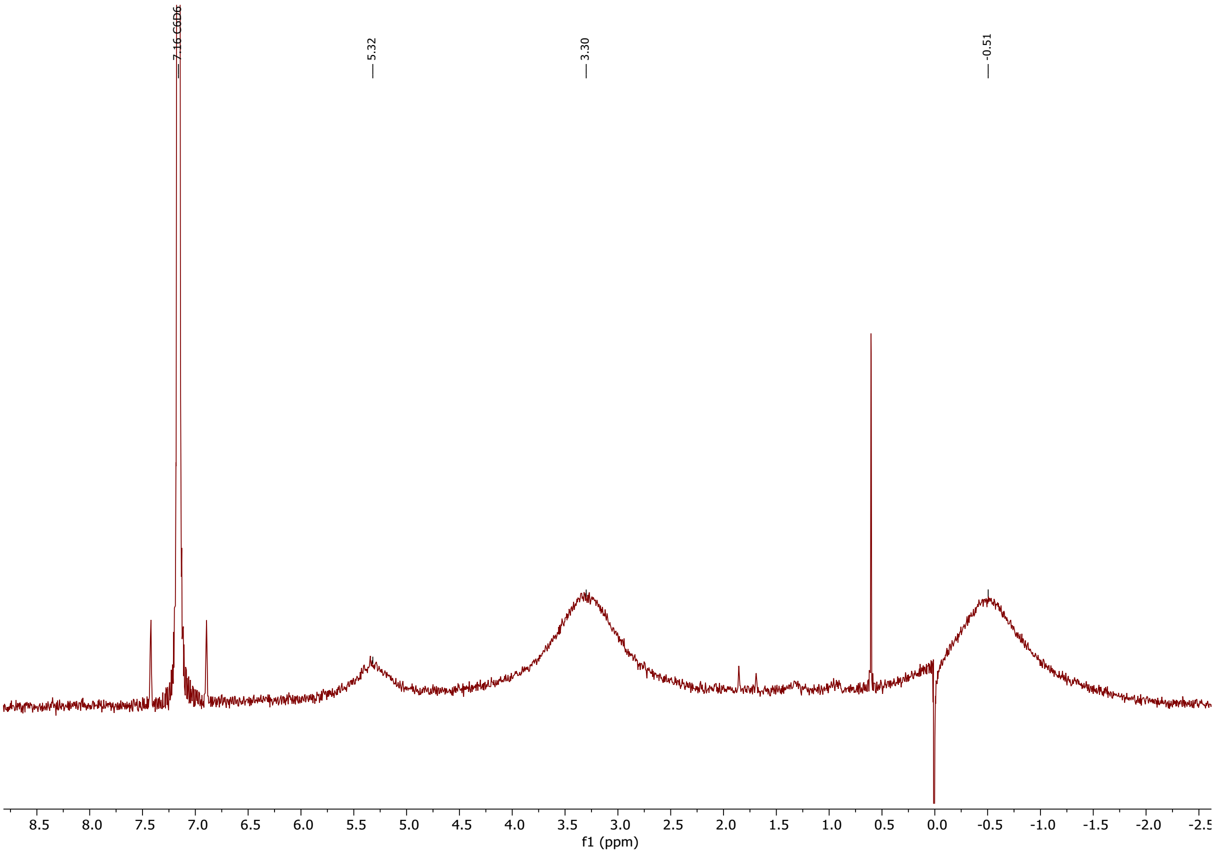

Figure 9: 300 MHz ^{1}H-NMR spectrum of cobalt pyridonate complex **Co2** in C_6D_6.

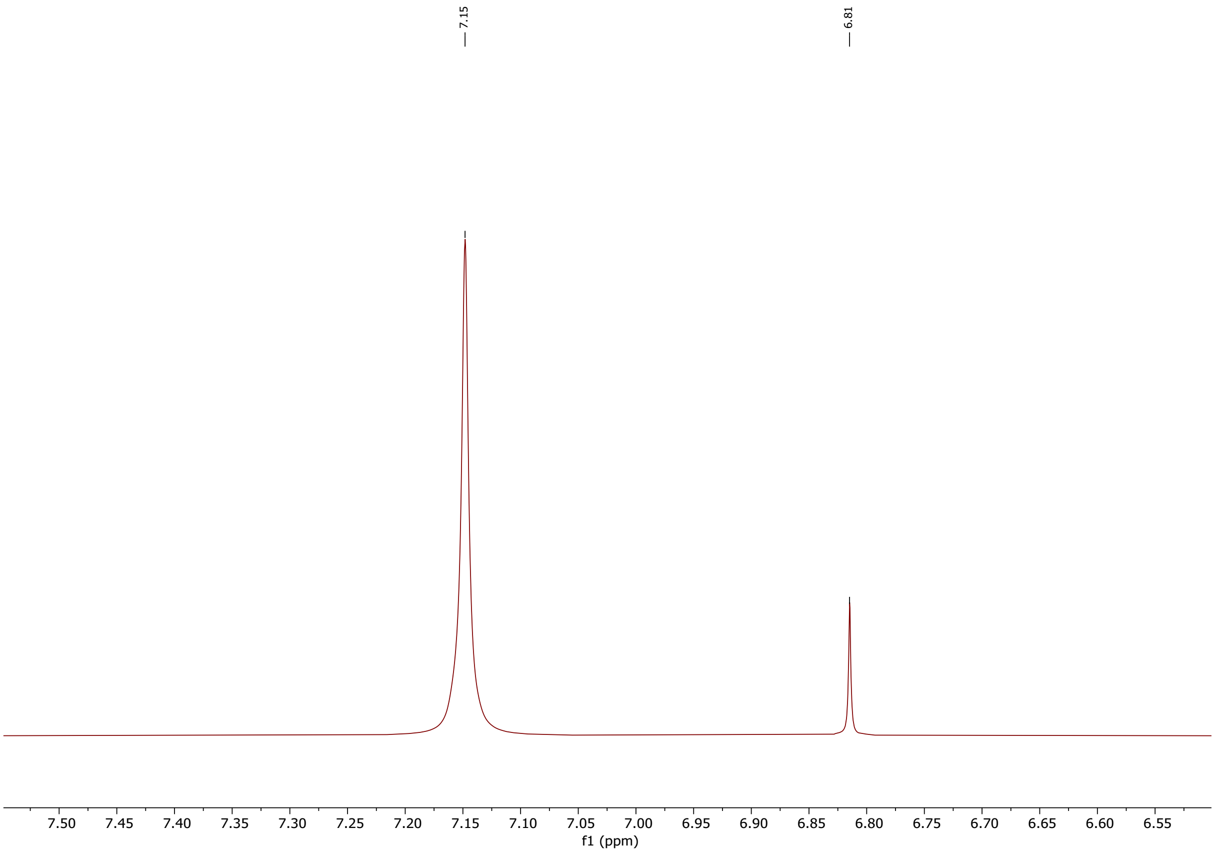

Figure 10: 300 MHz Evans-NMR spectrum of cobalt pyridonate complex **Co2** in C_6D_6 at c = 5.29x10^{-5} mol/mL (298.0 K).

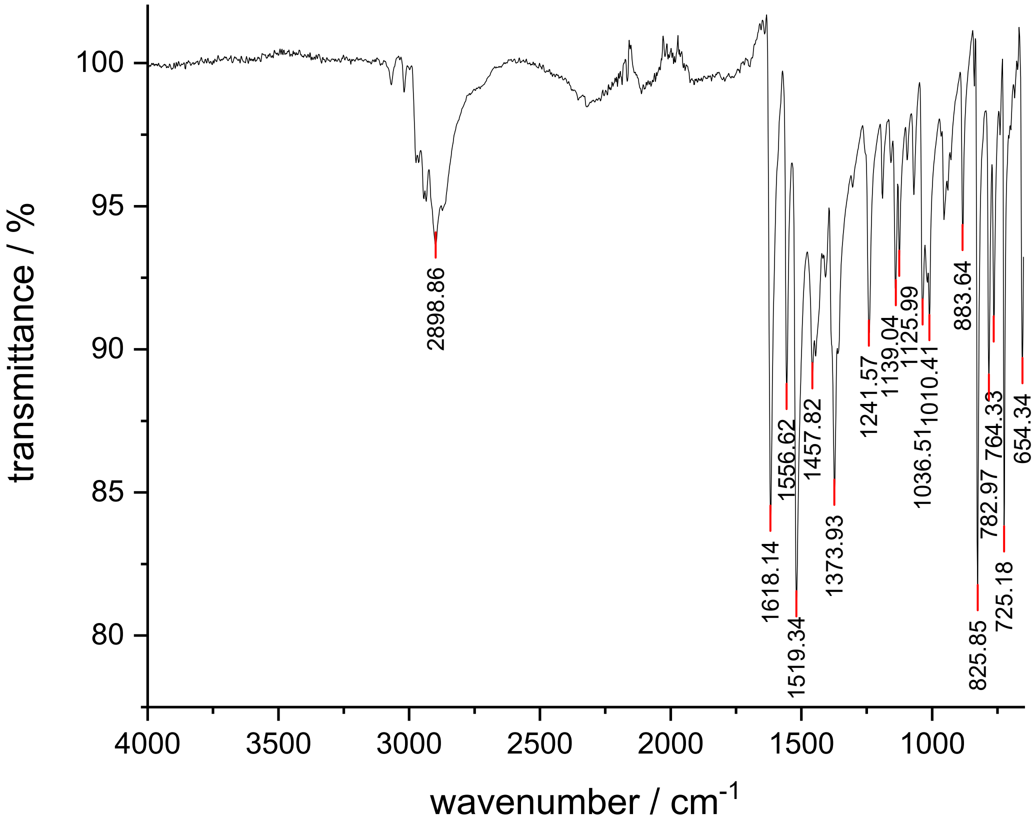

Figure 11: FT-IR spectrum of cobalt pyridonate complex **Co2**.

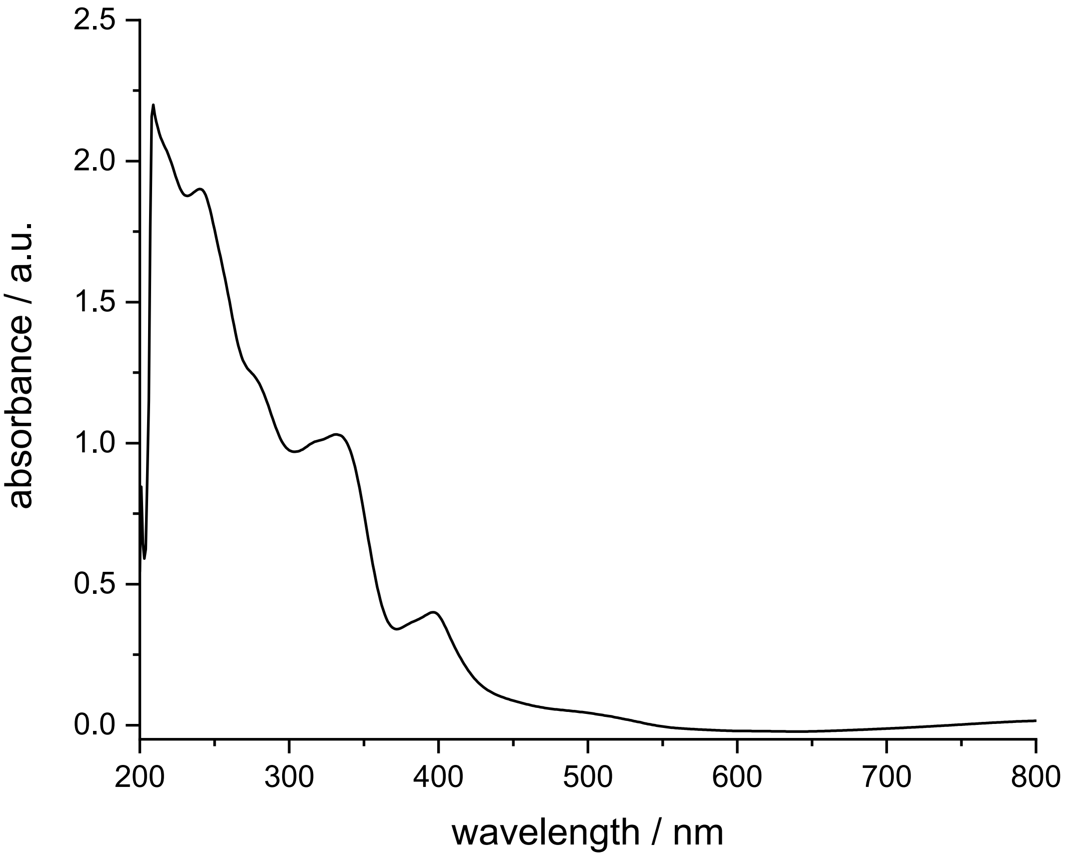

Figure 12: UV-Vis spectrum of cobalt pyridonate complex **Co2** at c = 0.05 mg/mL in THF.

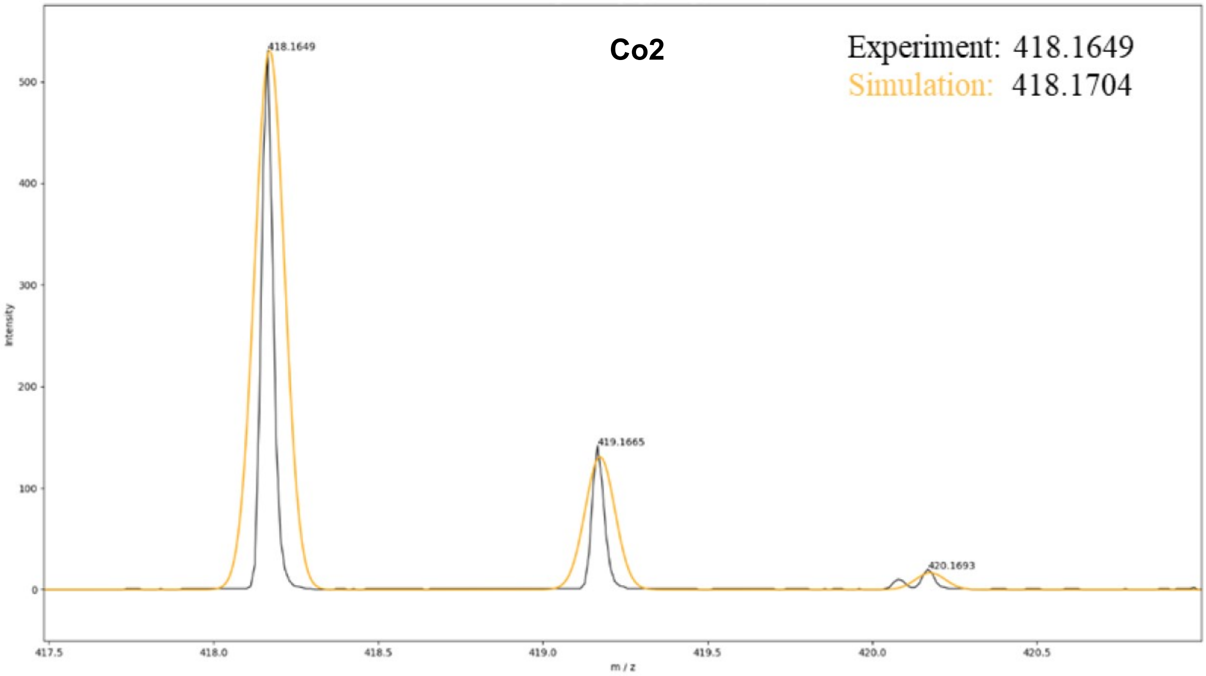

Figure 13: Fragment of the positive-ion mode ESI mass spectrum of **Co2** in THF. Measured and simulated isotopic patterns depicted in black and orange, respectively.

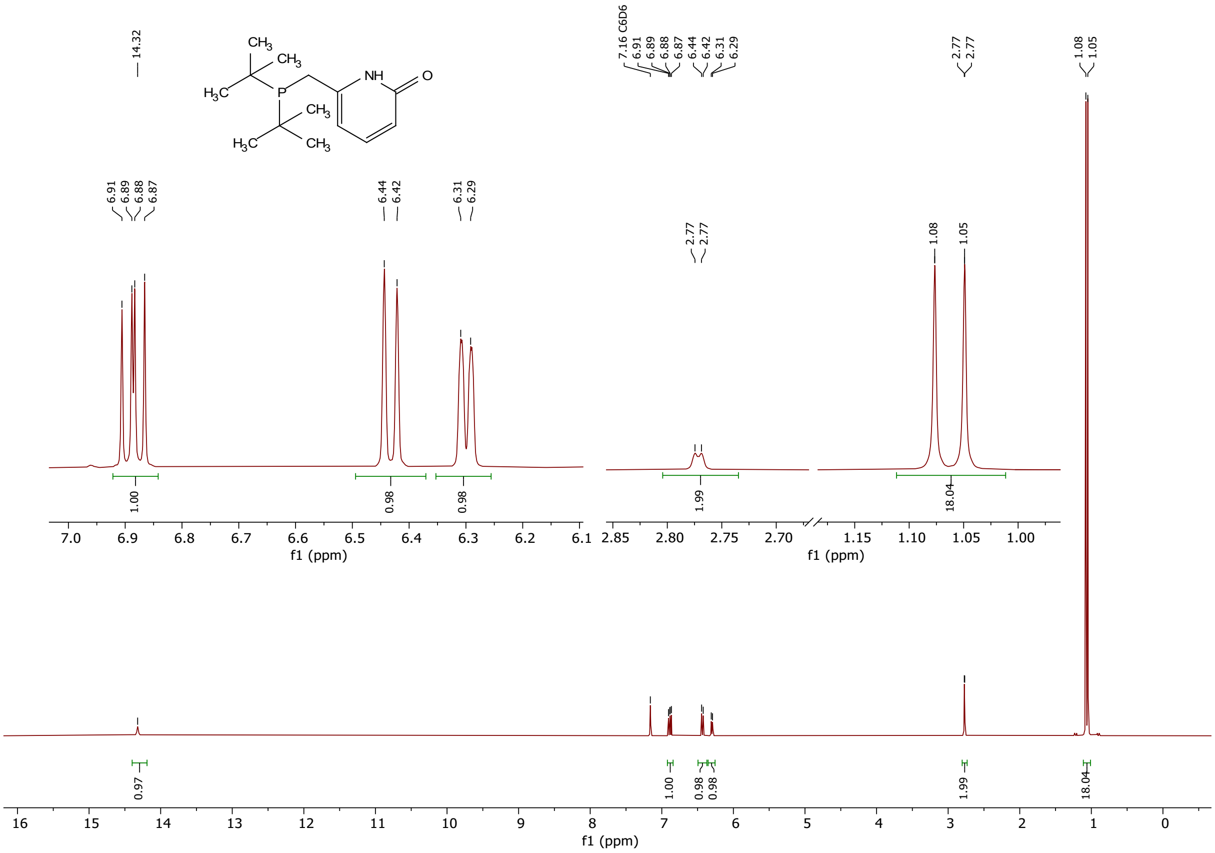

Figure 14: 400 MHz ^{1}H-NMR spectrum of pyridone ligand **L2H** in C_6D_6.

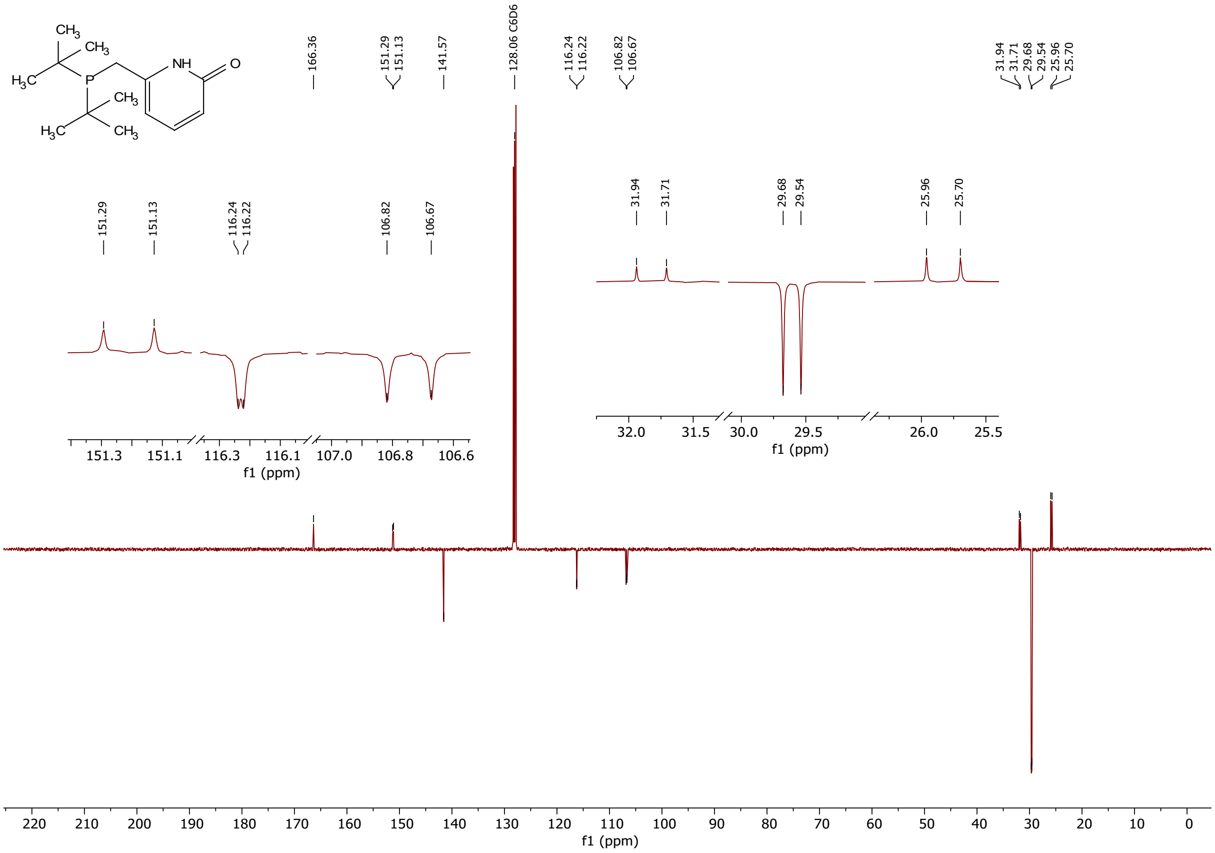

Figure 15: 101 MHz ^{13}C-DEPTQ-NMR spectrum of pyridone ligand **L2H** in C_6D_6.

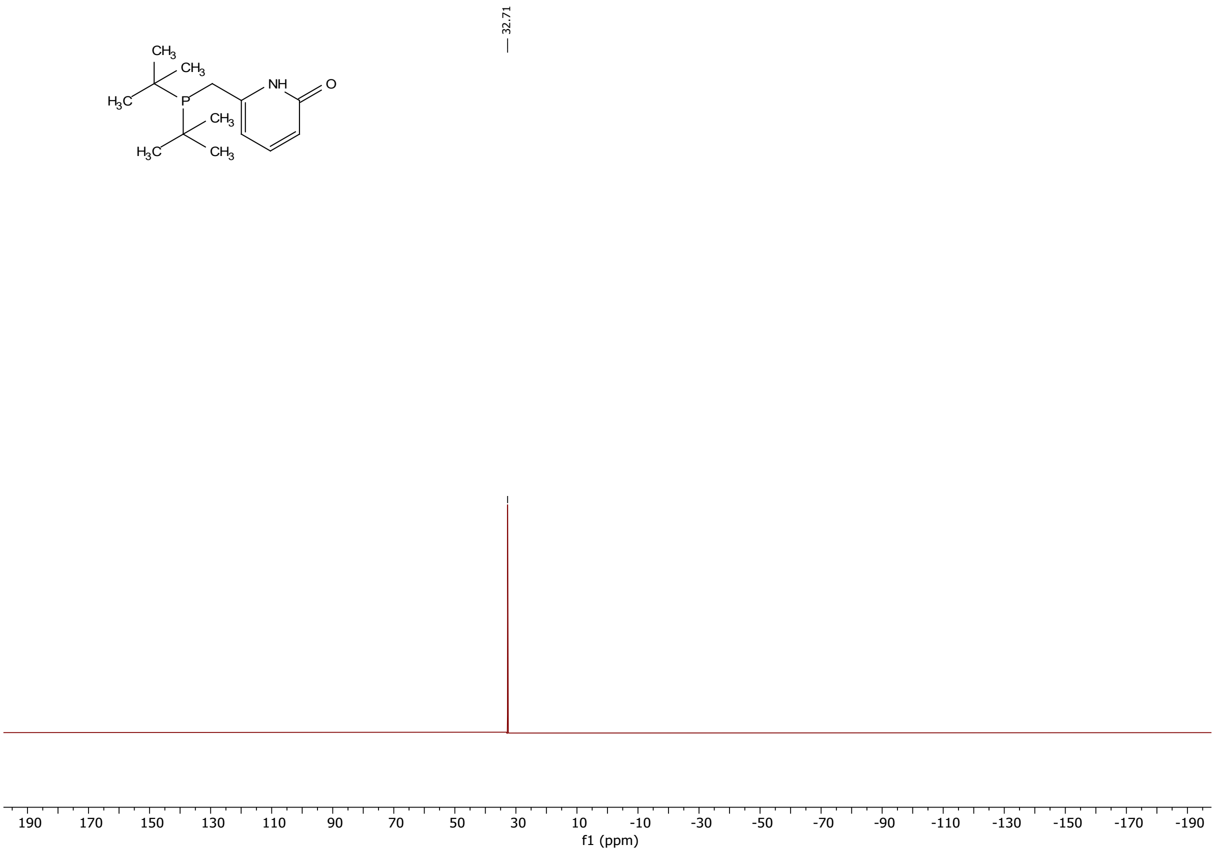

Figure 16: 162 MHz $^{31}P\{^1H\}$-NMR spectrum of pyridone ligand **L2H** in C_6D_6.

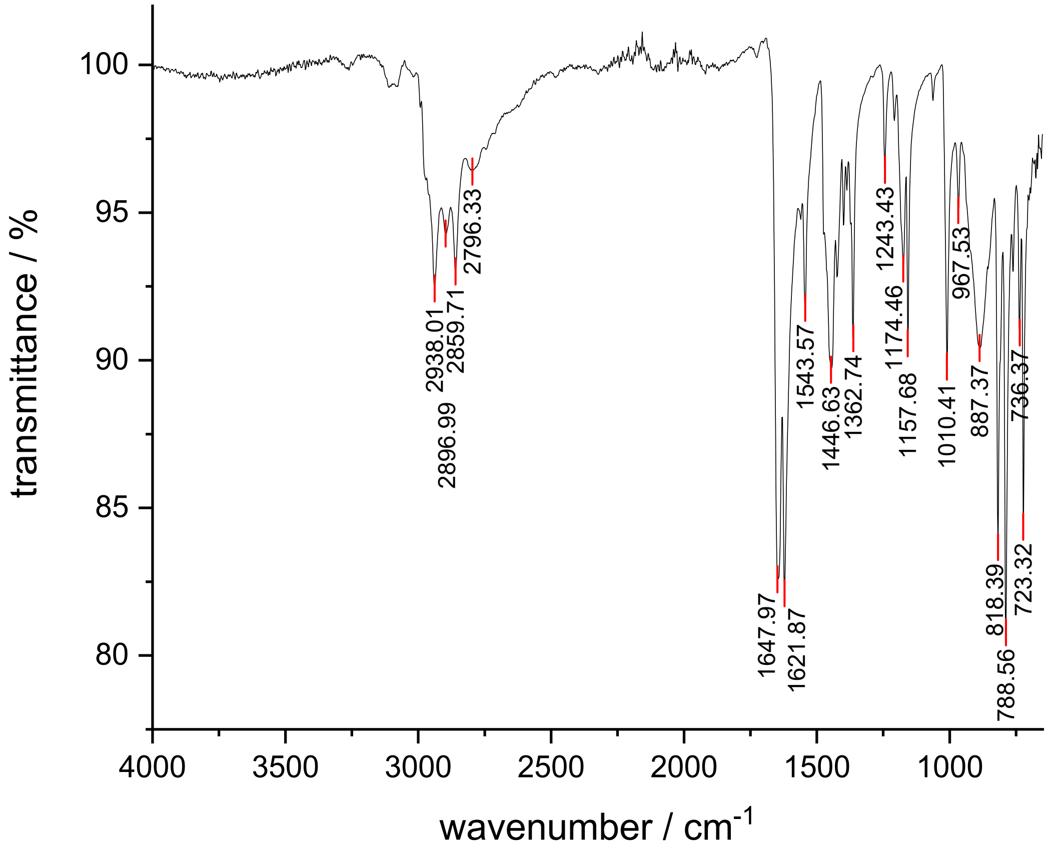

Figure 17: FT-IR spectrum of pyridone ligand **L2H**.

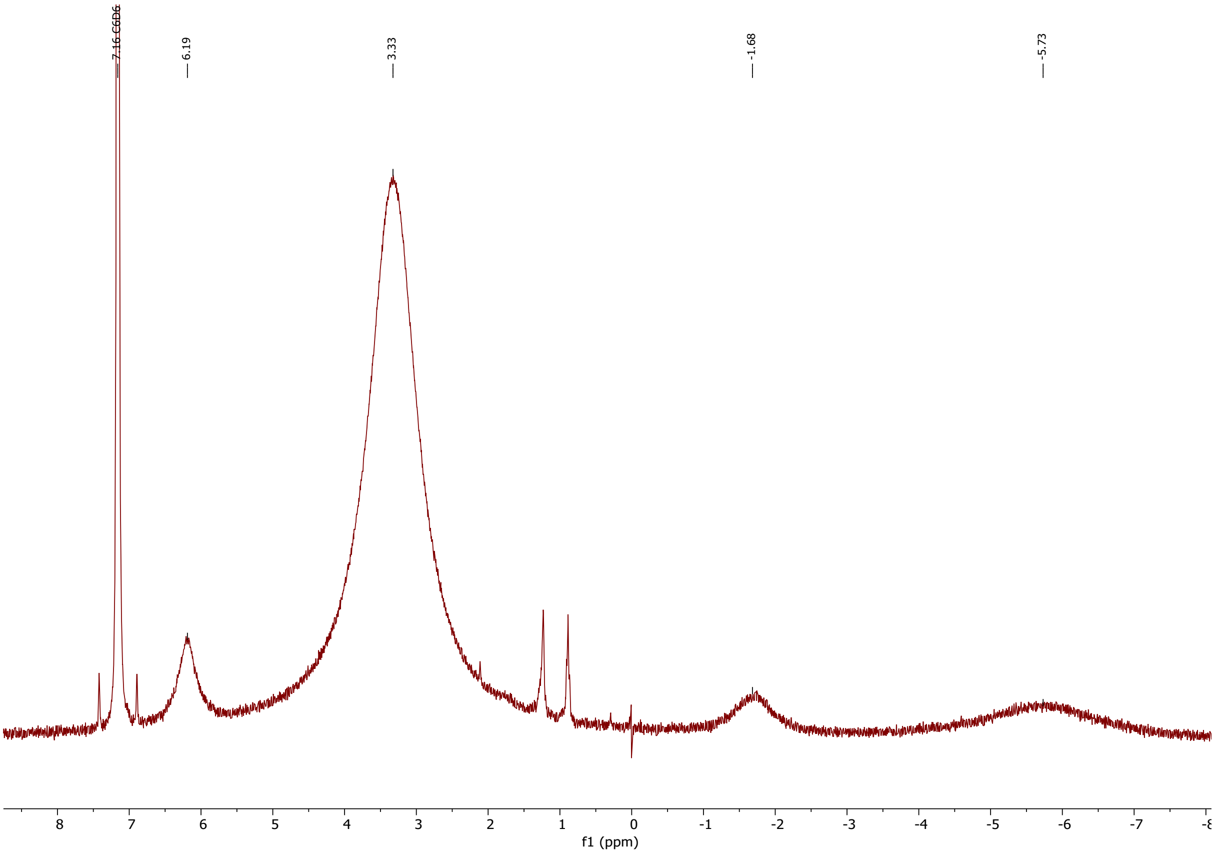

Figure 18: 300 MHz ^{1}H-NMR spectrum of cobalt pyridonate complex **Co3** in C_6D_6.

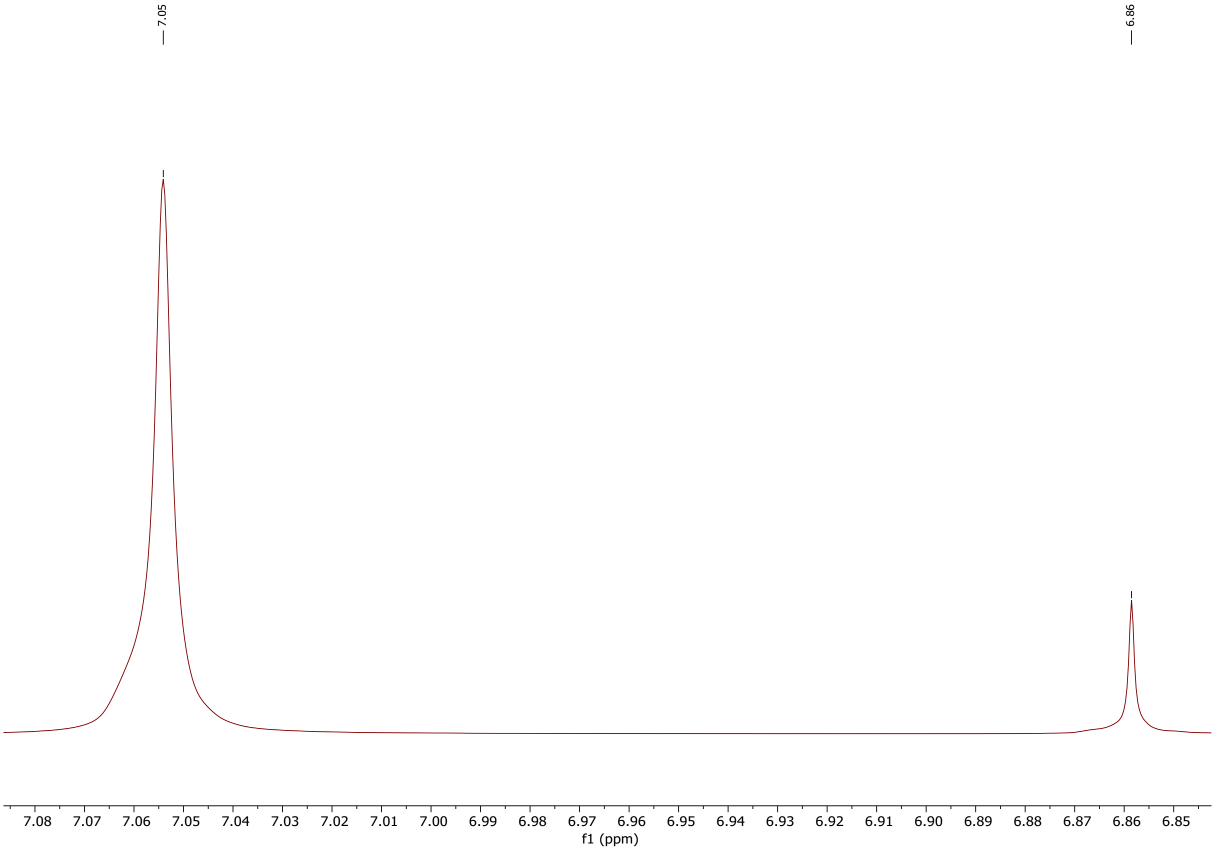

Figure 19: 400 MHz Evans-NMR spectrum of cobalt pyridonate complex **Co3** in C_6D_6 at c = 3.35x10^{-5} mol/mL (299.5 K).

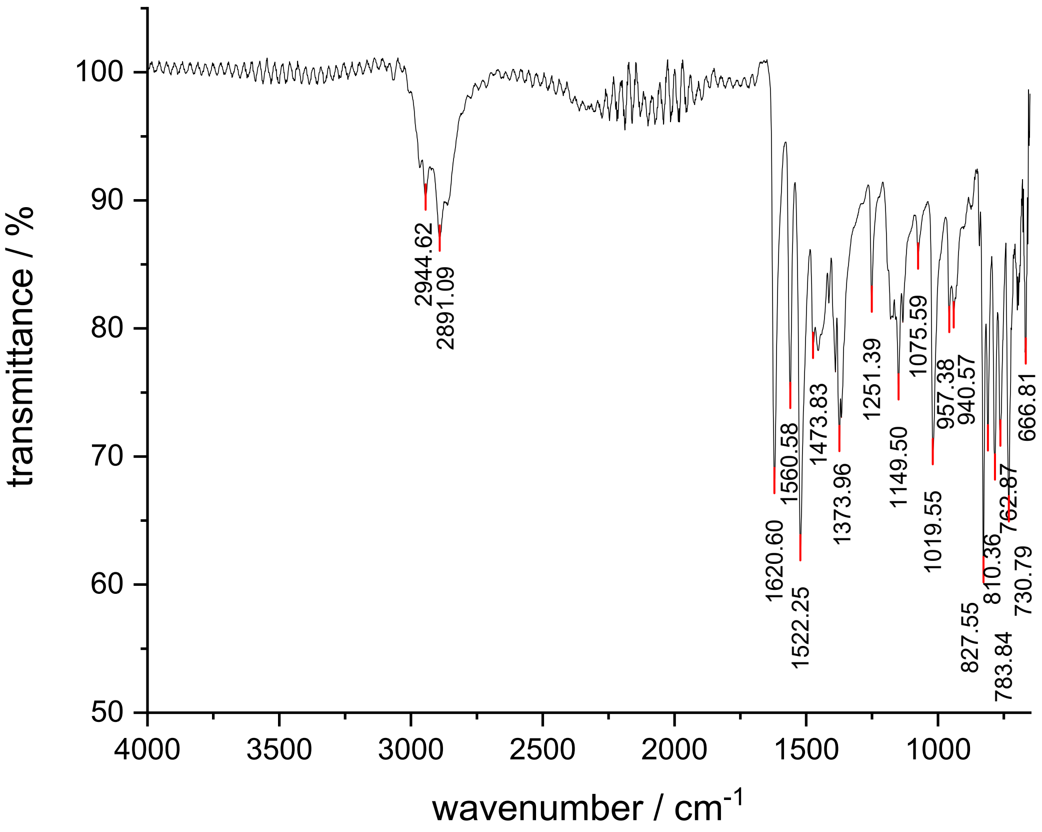

Figure 20: FT-IR spectrum of cobalt pyridonate complex **Co3**.

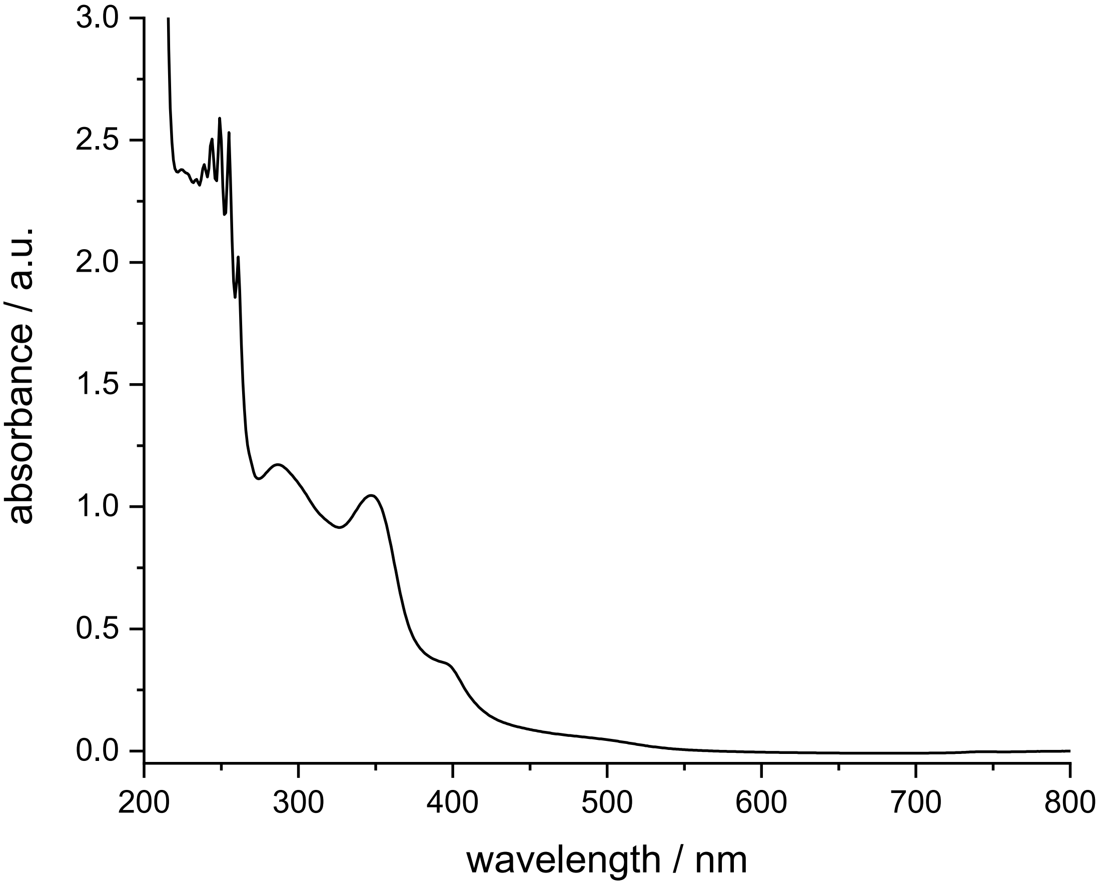

Figure 21: UV-Vis spectrum of cobalt pyridonate complex **Co3** at c = 0.05 mg/mL in THF.

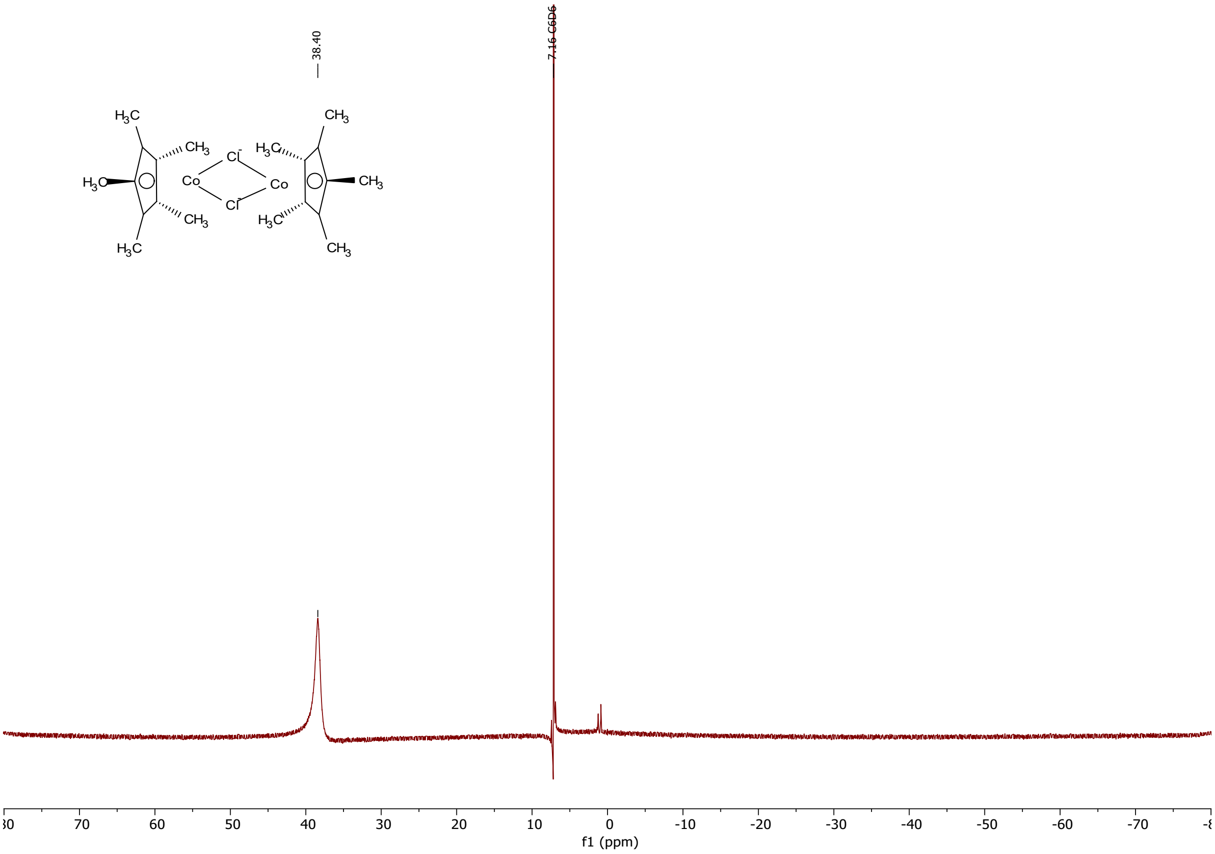

Figure 22: 300 MHz ^{1}H-NMR spectrum of cobalt precursor **Co4** in C_6D_6.

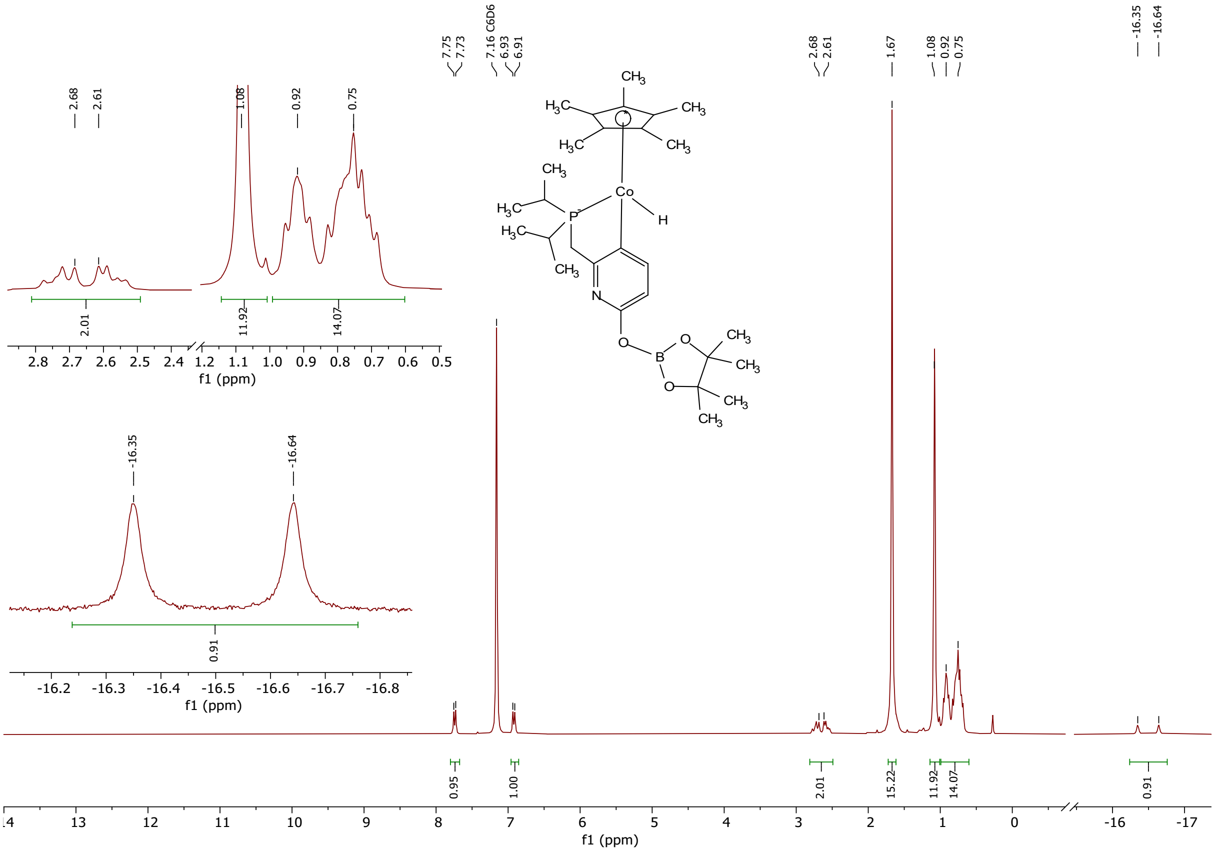

Figure 23: 300 MHz ^{1}H-NMR spectrum of cobalt(III) monohydride **Co6** in C_6D_6.

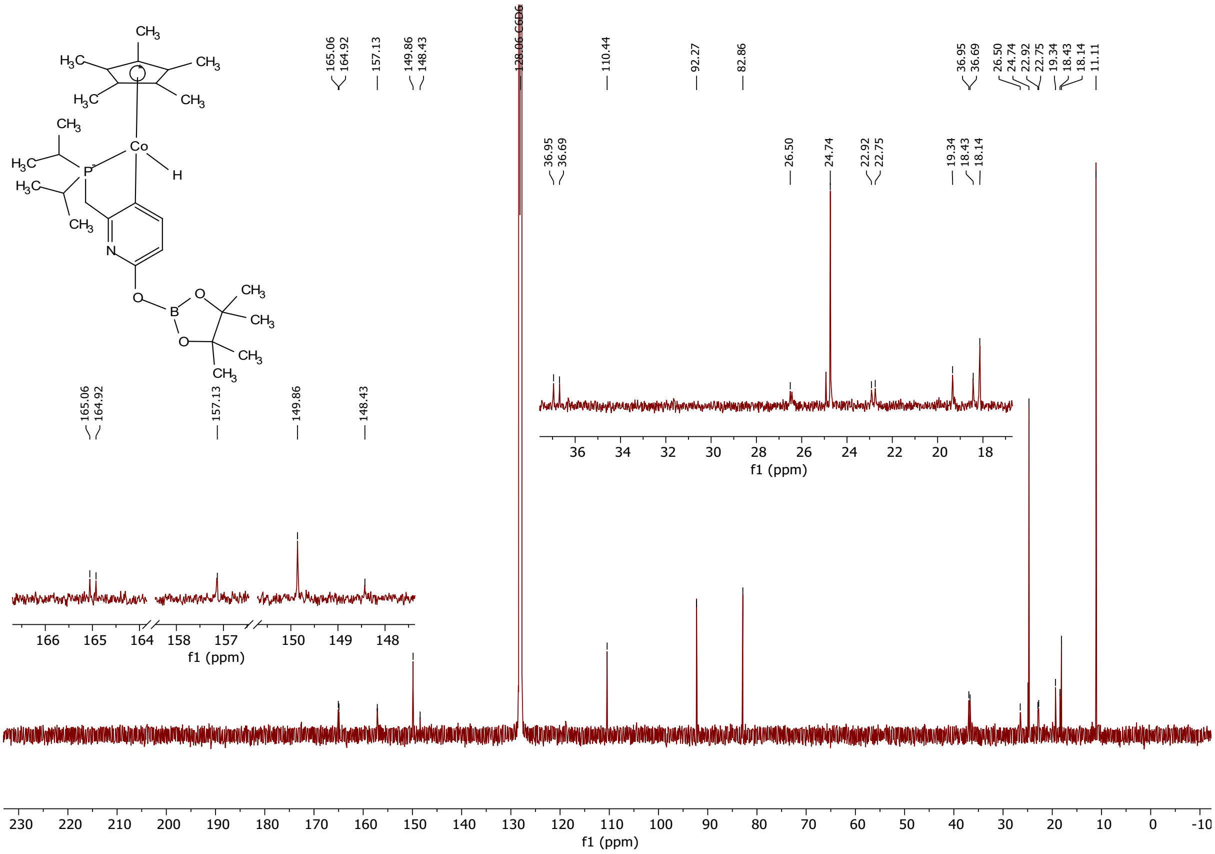

Figure 24: 151 MHz $^{13}C\{^1H\}$-NMR spectrum of cobalt(III) monohydride **Co6** in C_6D_6.

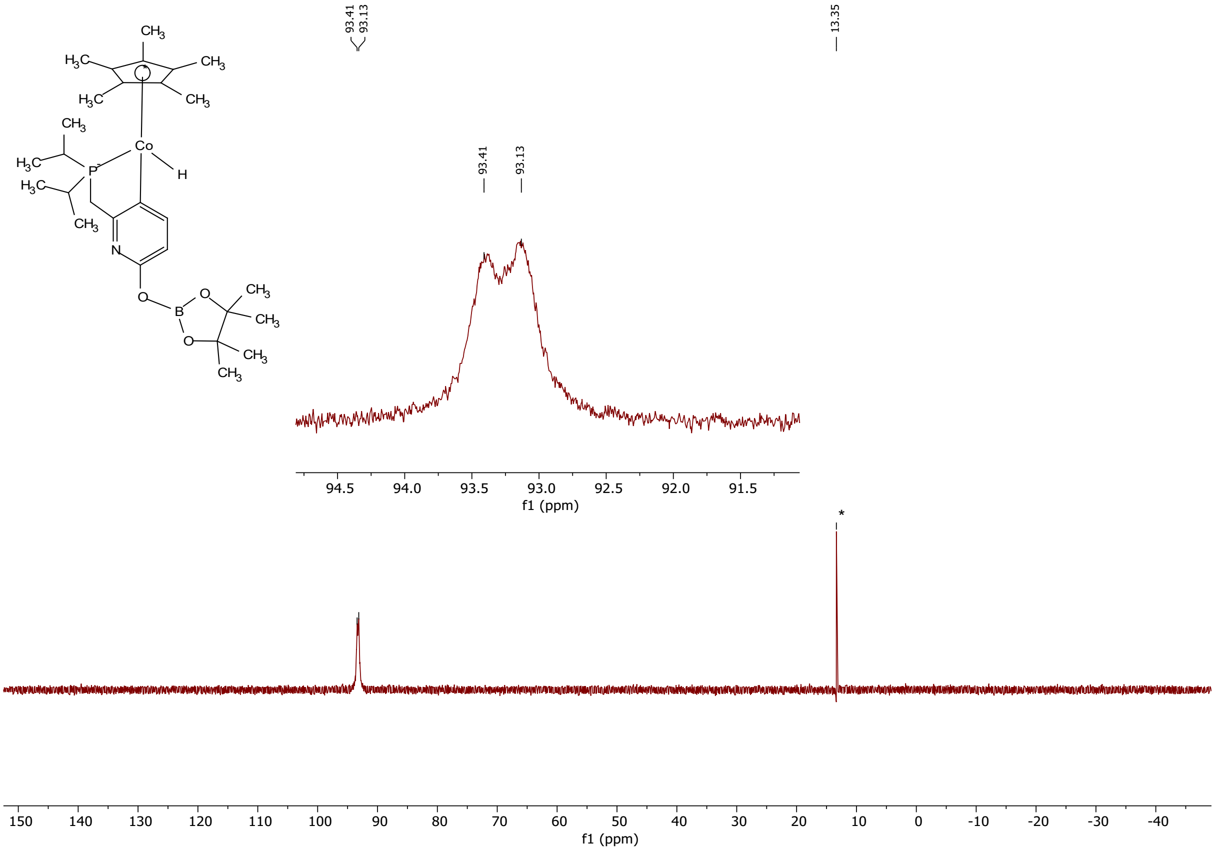

Figure 25: 243 MHz $^{31}P\{^1H\}$-NMR spectrum of cobalt(III) monohydride **Co6** in C_6D_6. (*) Signal of **L1Bpin**.

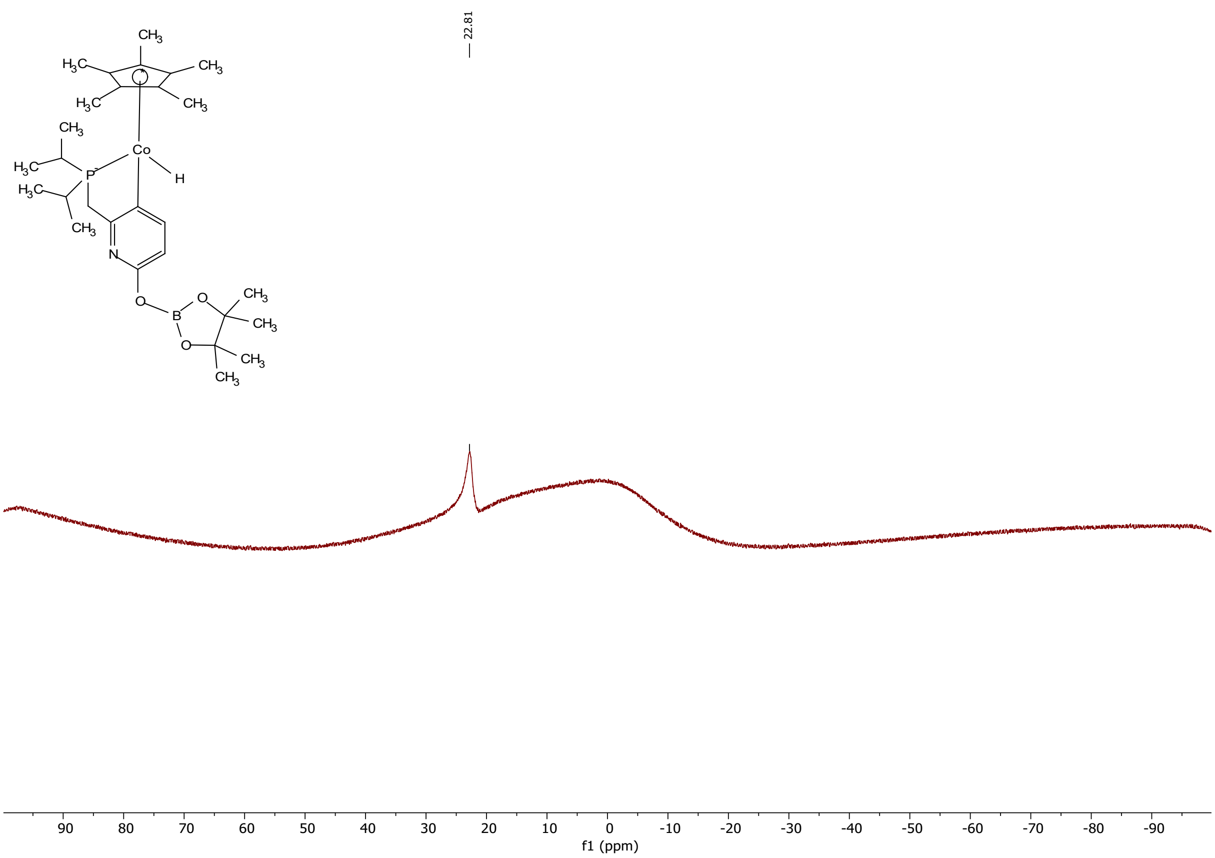

Figure 26: 193 MHz ^{11}B-NMR spectrum of cobalt(III) monohydride **Co6** in C_6D_6.

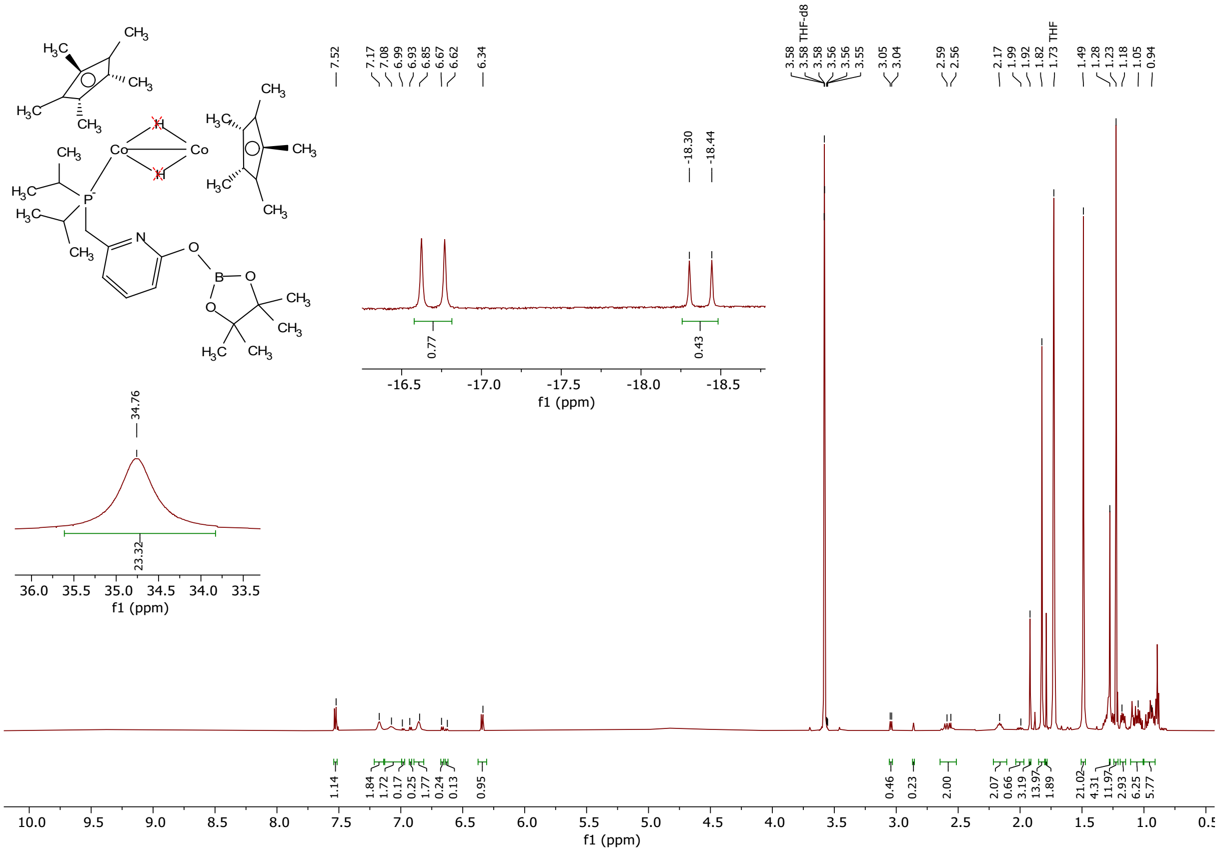

Figure 27: 600 MHz ^{1}H-NMR spectrum of **Co8** in THF-d_8.

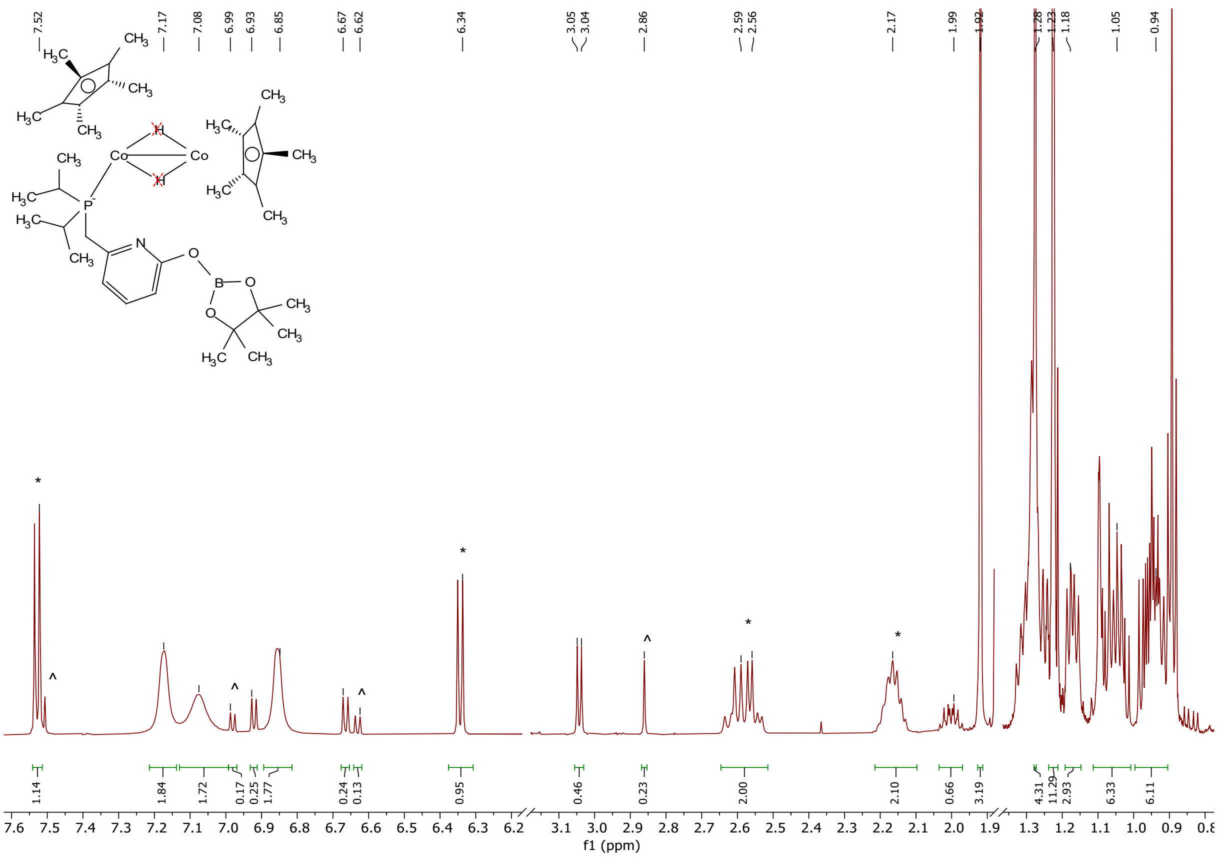

Figure 28: Aromatic and upfield region of the ^{1}H-NMR spectrum of **Co8** in THF-d_8. (*) Signals of **Co6**. (^) Signals of **L1Bpin**.

11.2 Experimental Spectra of bis(borylated) Tetrahydropyridines

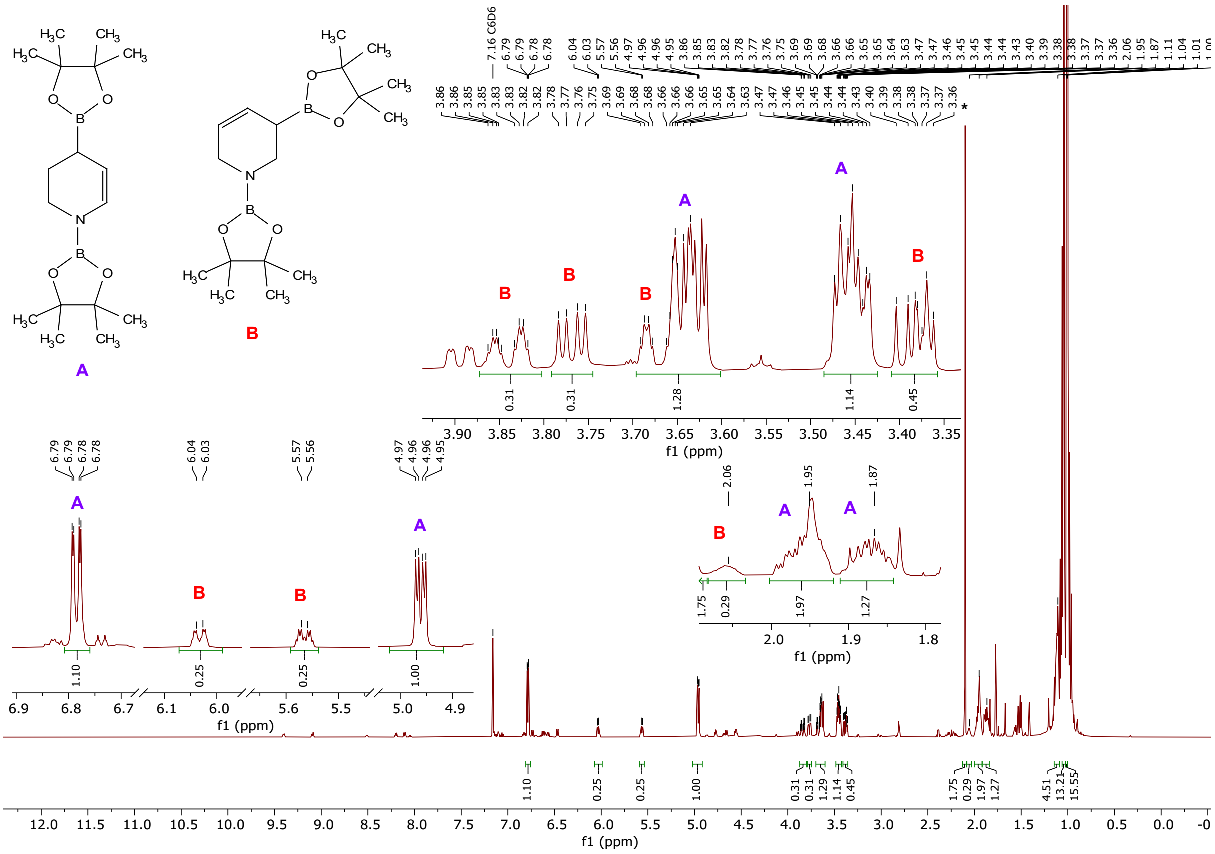

Figure 29: 600 MHz ^{1}H-NMR crude spectrum of **3c** and **3c'** in C_6D_6 obtained after double hydroboration of pyridine. (*) Internal standard hexamethylbenzene (0.02 mmol).

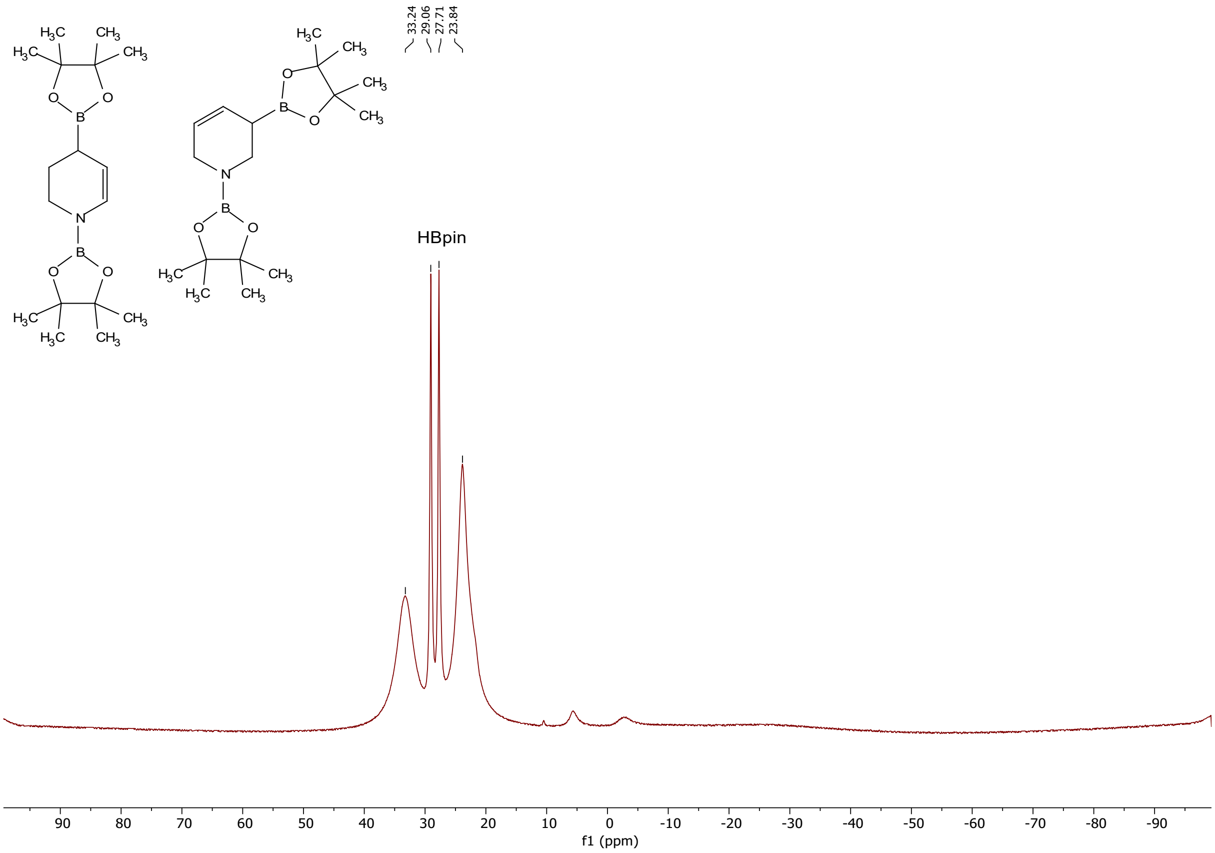

Figure 30: 128 MHz ^{11}B-NMR crude spectrum of **3c** and **3c'** in C_6D_6 obtained after double hydroboration of pyridine.

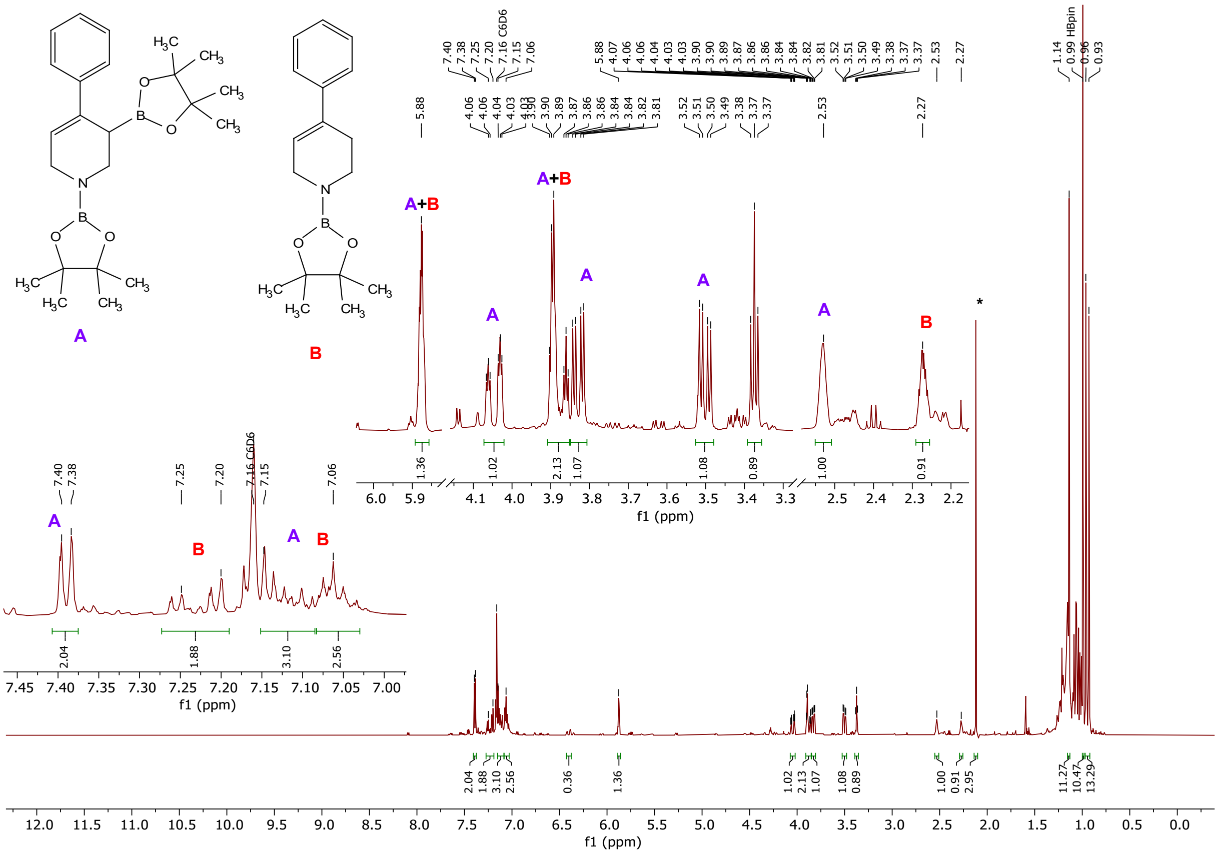

Figure 31: 600 MHz ^{1}H-NMR crude spectrum of **3b** and **3b'** in C_6D_6 obtained after double hydroboration of 4-phenylpyridine. (*) Internal standard hexamethylbenzene (13 µmol).

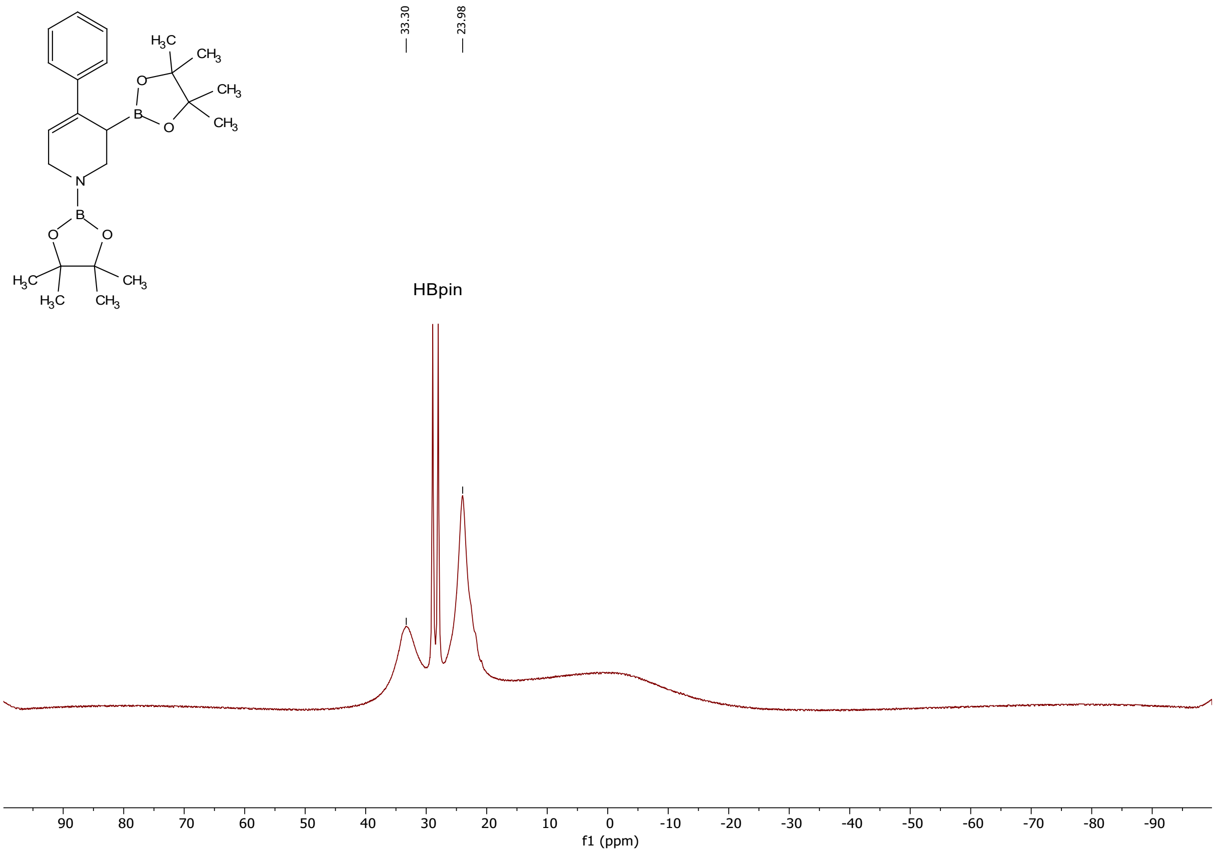

Figure 32: 193 MHz ^{11}B-NMR crude spectrum of **3b** in C_6D_6 obtained after double hydroboration of 4-phenyl-pyridine.

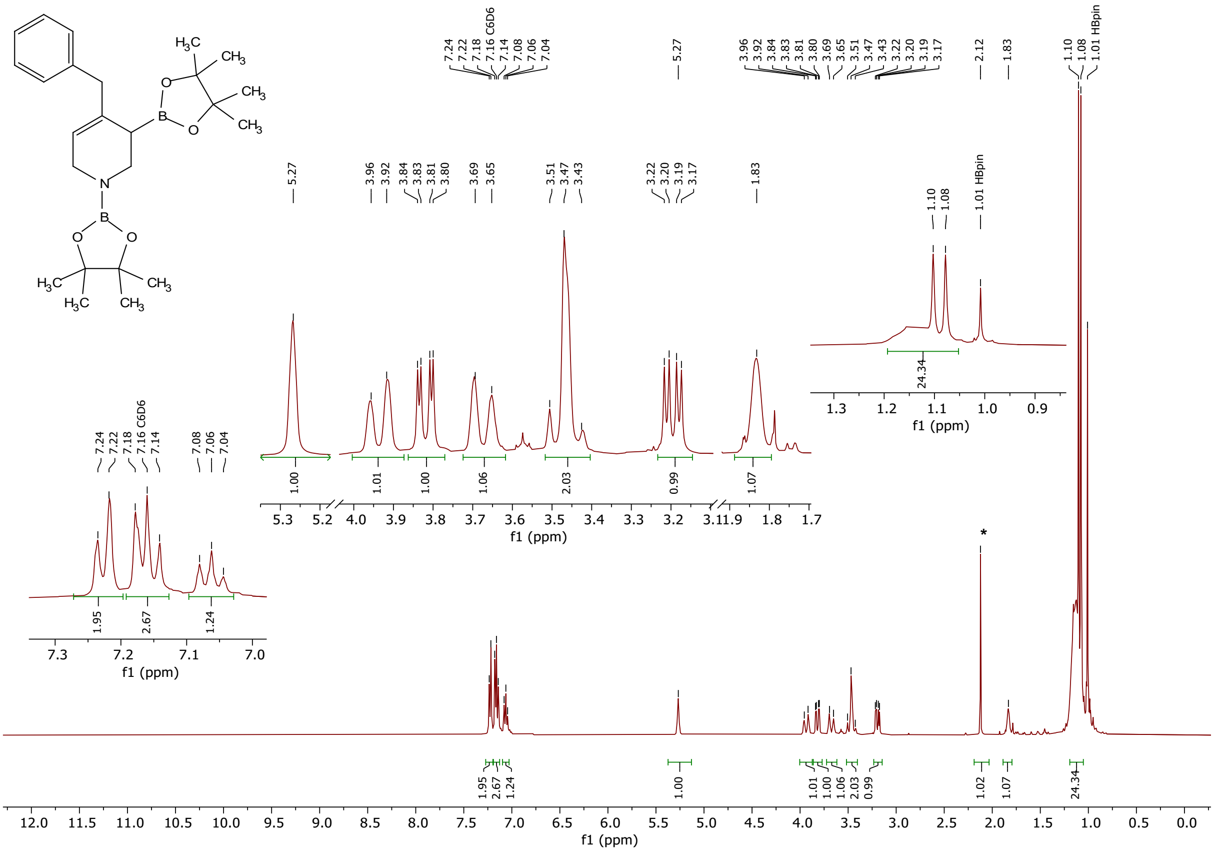

Figure 33: 400 MHz ^{1}H-NMR crude spectrum of **3e** in C_6D_6 obtained after double hydroboration of 4-benzylpyridine. (*) Internal standard hexamethylbenzene (0.02 mmol).

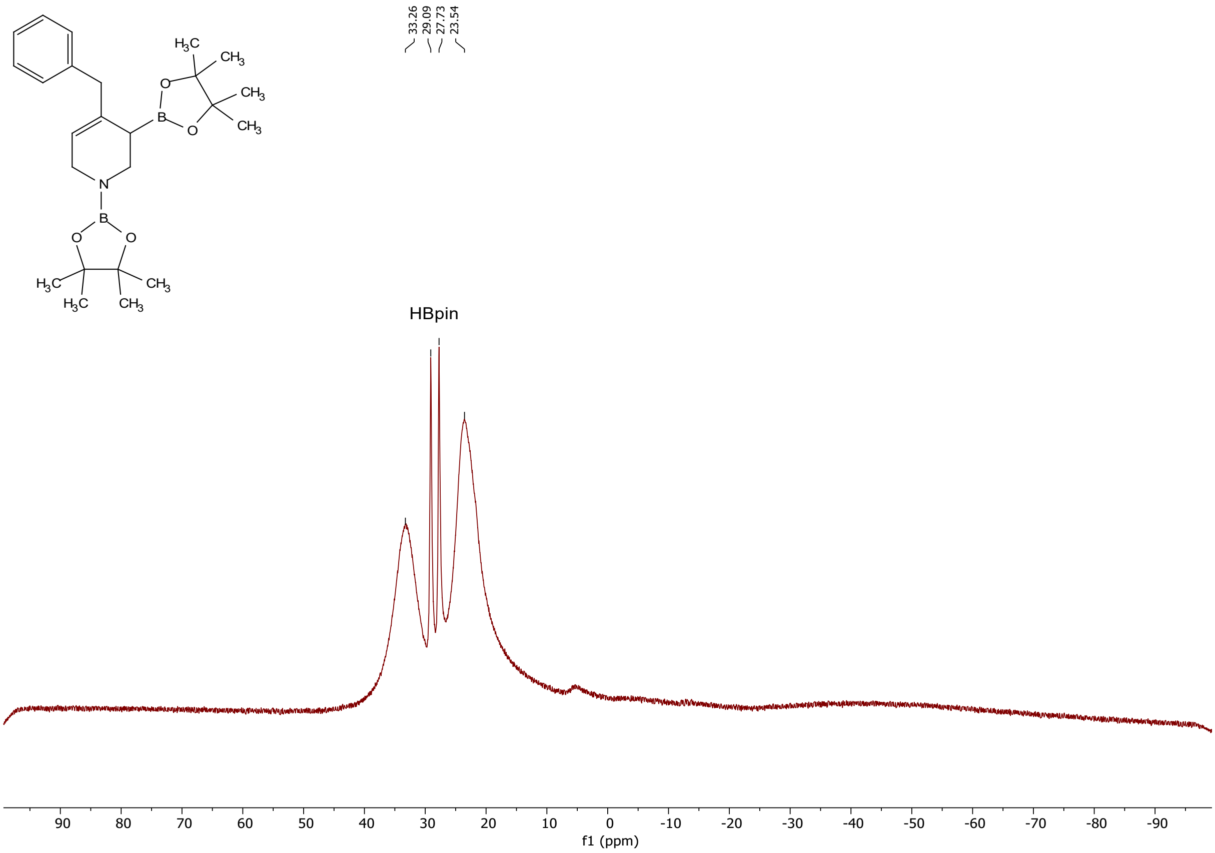

Figure 34: 128 MHz ^{11}B-NMR crude spectrum of **3e** in C_6D_6 obtained after double hydroboration of 4-benzyl-pyridine.

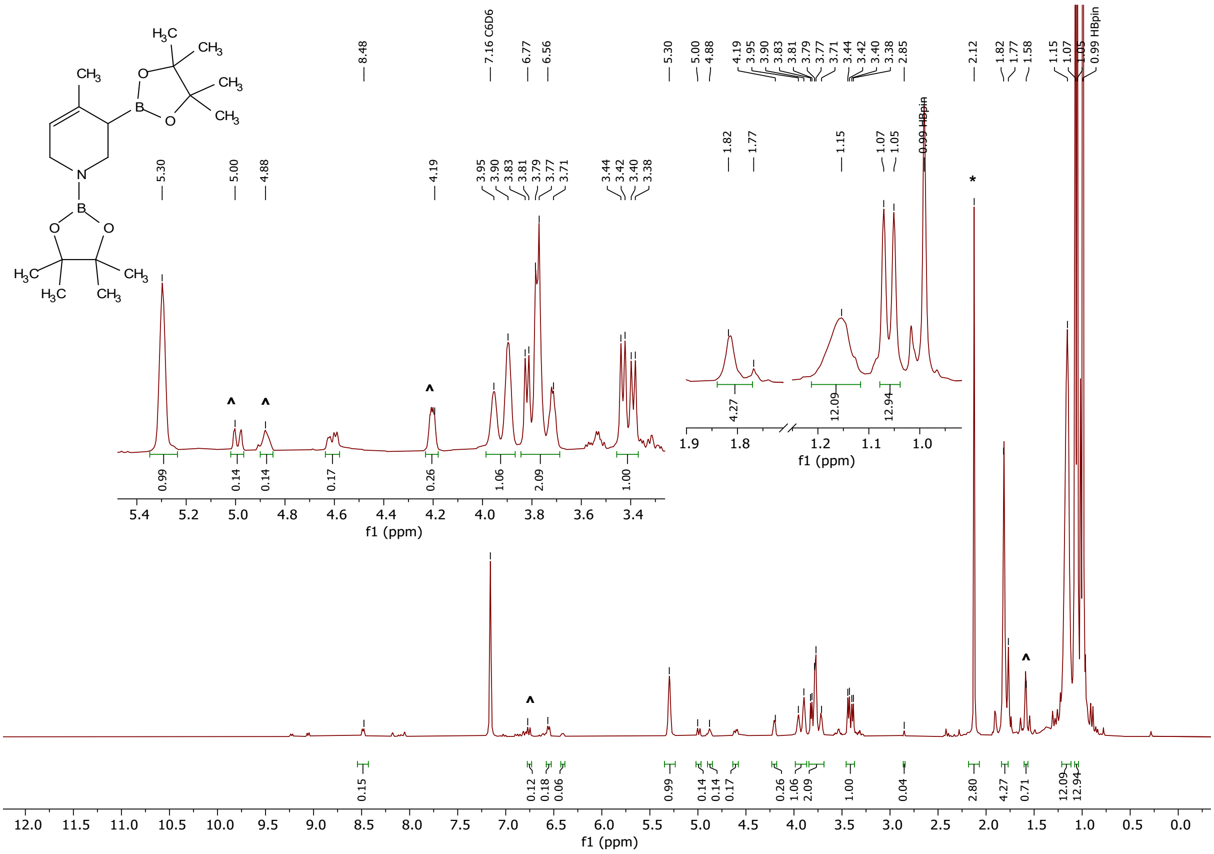

Figure 35: 300 MHz ^{1}H-NMR crude spectrum of **3a** in C_6D_6 obtained after double hydroboration of 4-methylpyridine. (^) Signals of *N*-boryl 1,2-DHP. (*) Internal standard hexamethylbenzene (0.04 mmol).

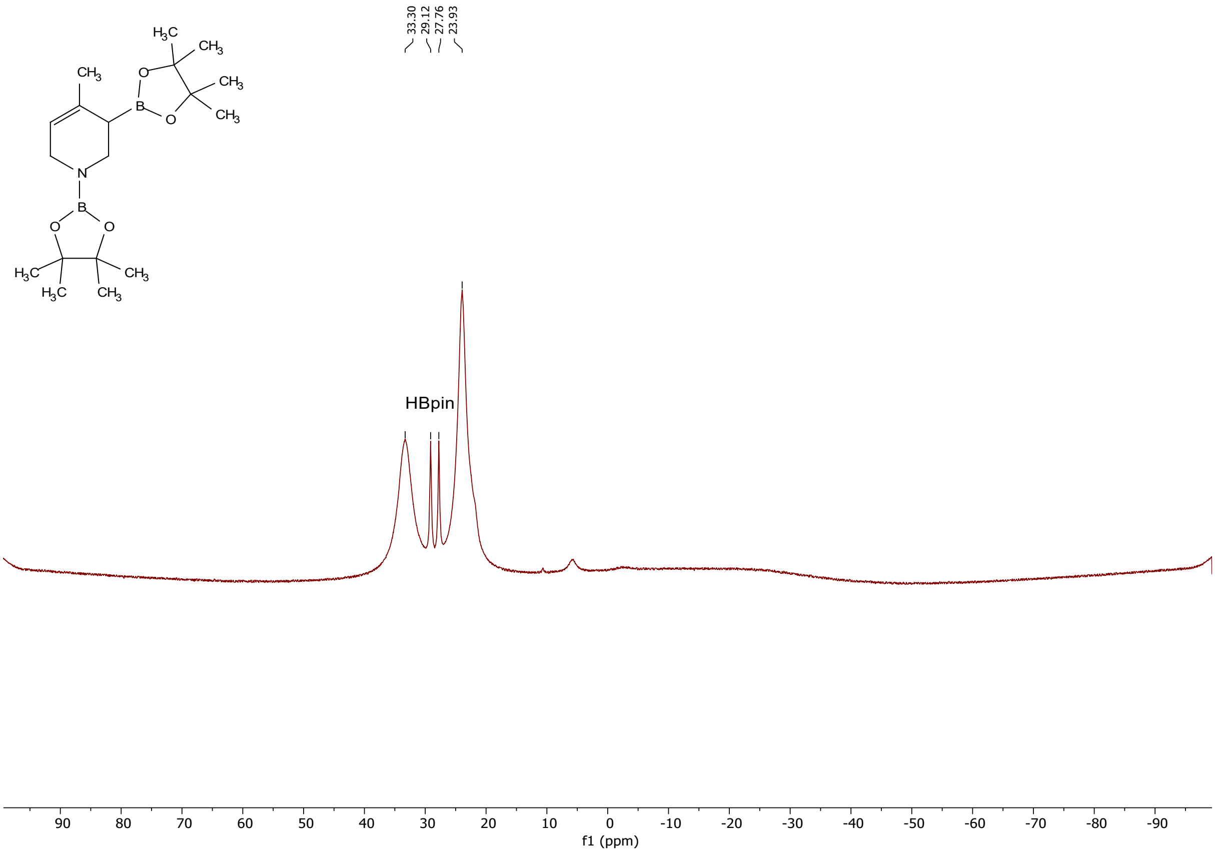

Figure 36: 128 MHz ^{11}B-NMR crude spectrum of **3a** in C_6D_6 obtained after double hydroboration of 4-methyl-pyridine.

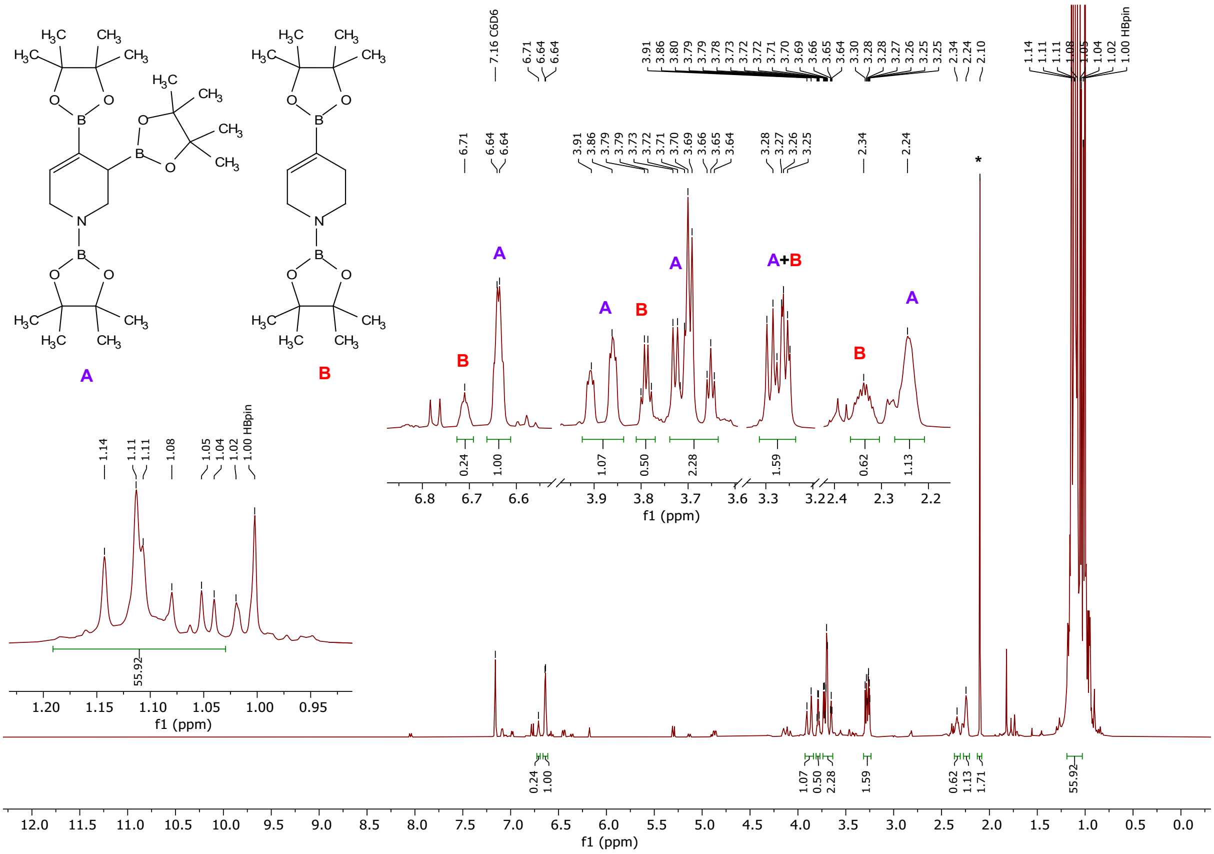

Figure 37: 400 MHz ^{1}H-NMR crude spectrum of **3g** in C_6D_6 obtained after double hydroboration of 4-(4,4,5,5-tetramethyl-1,3,2-dioxaborolan-2-yl)pyridine. (*) Internal standard hexamethylbenzene (0.02 mmol).

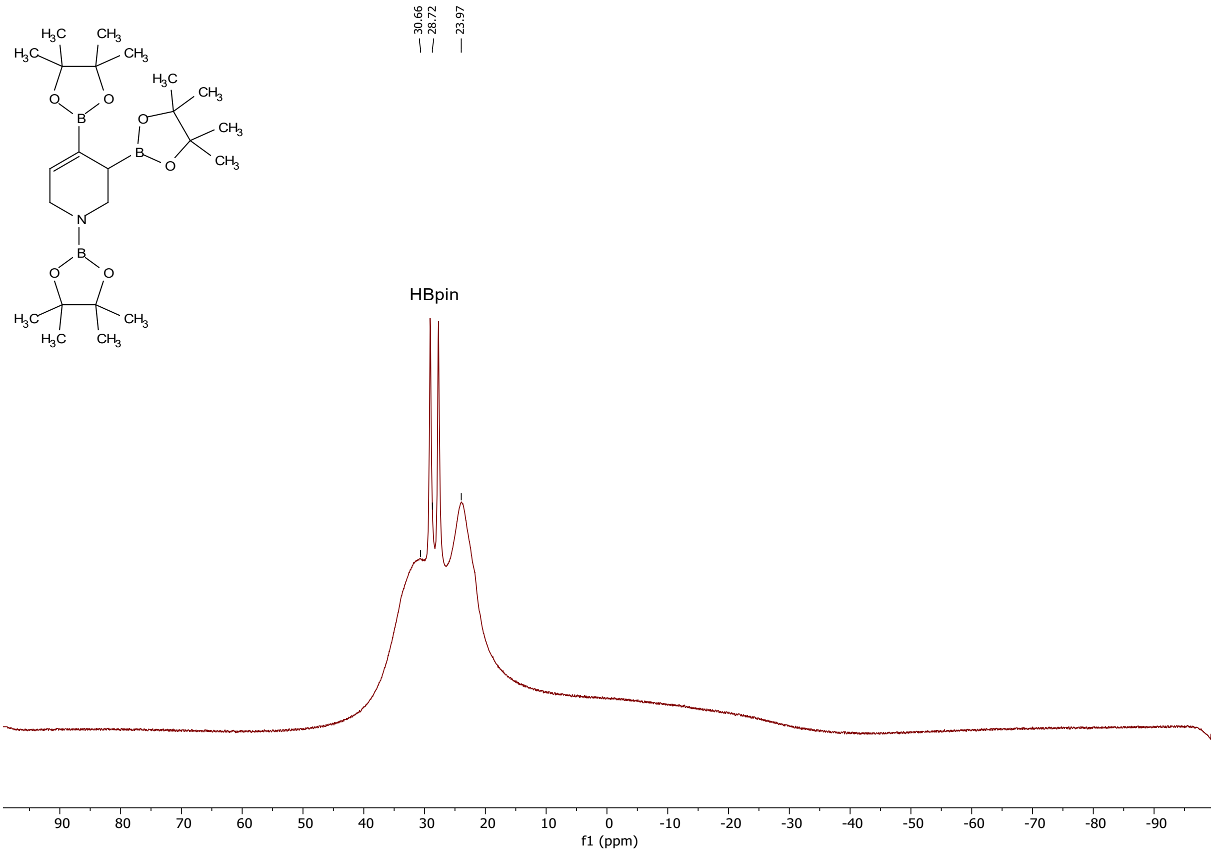

Figure 38: 128 MHz ^{11}B-NMR crude spectrum of **3g** in C_6D_6 obtained after double hydroboration of 4-(4,4,5,5-tetramethyl-1,3,2-dioxaborolan-2-yl)pyridine.

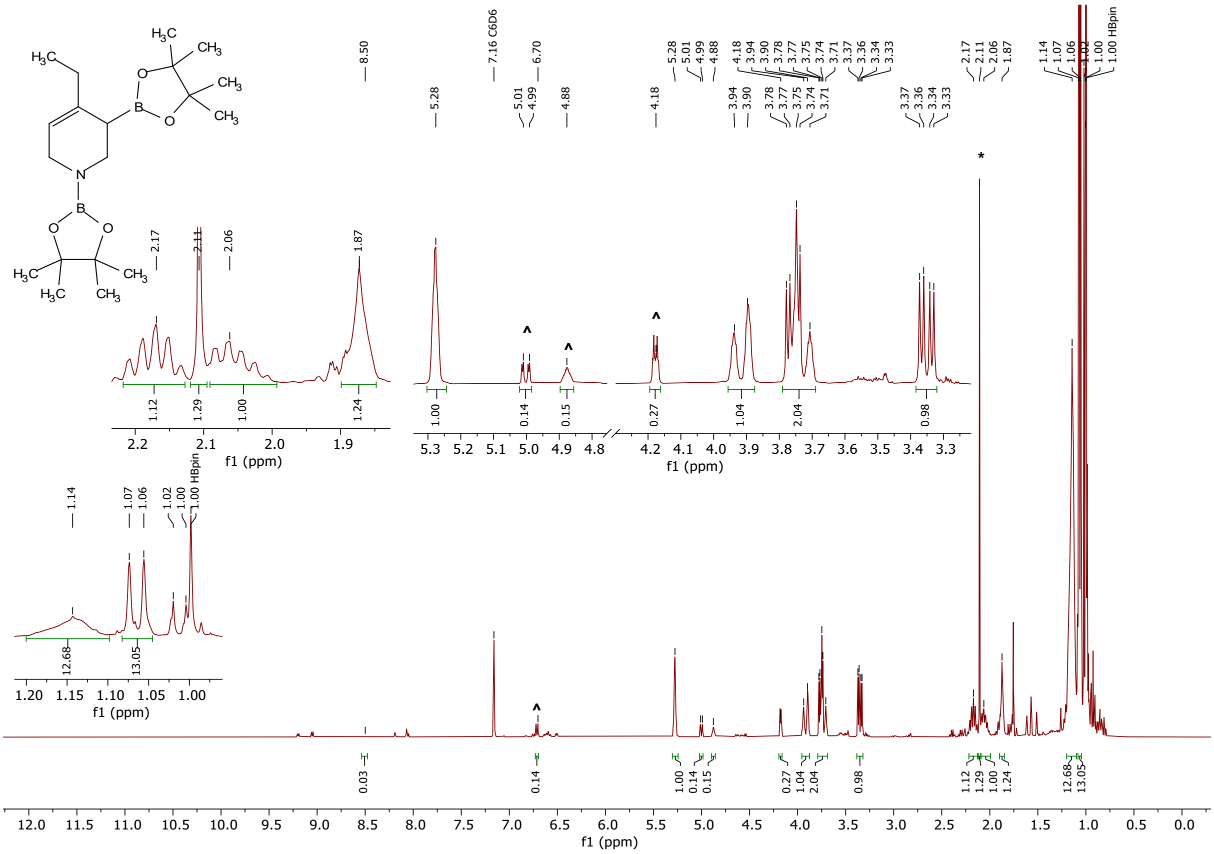

Figure 39: 400 MHz ^1H-NMR crude spectrum of **3d** in C_6D_6 obtained after double hydroboration of 4-ethylpyridine. (^) Signals of *N*-boryl 1,2-DHP. (*) Internal standard hexamethylbenzene (0.02 mmol).

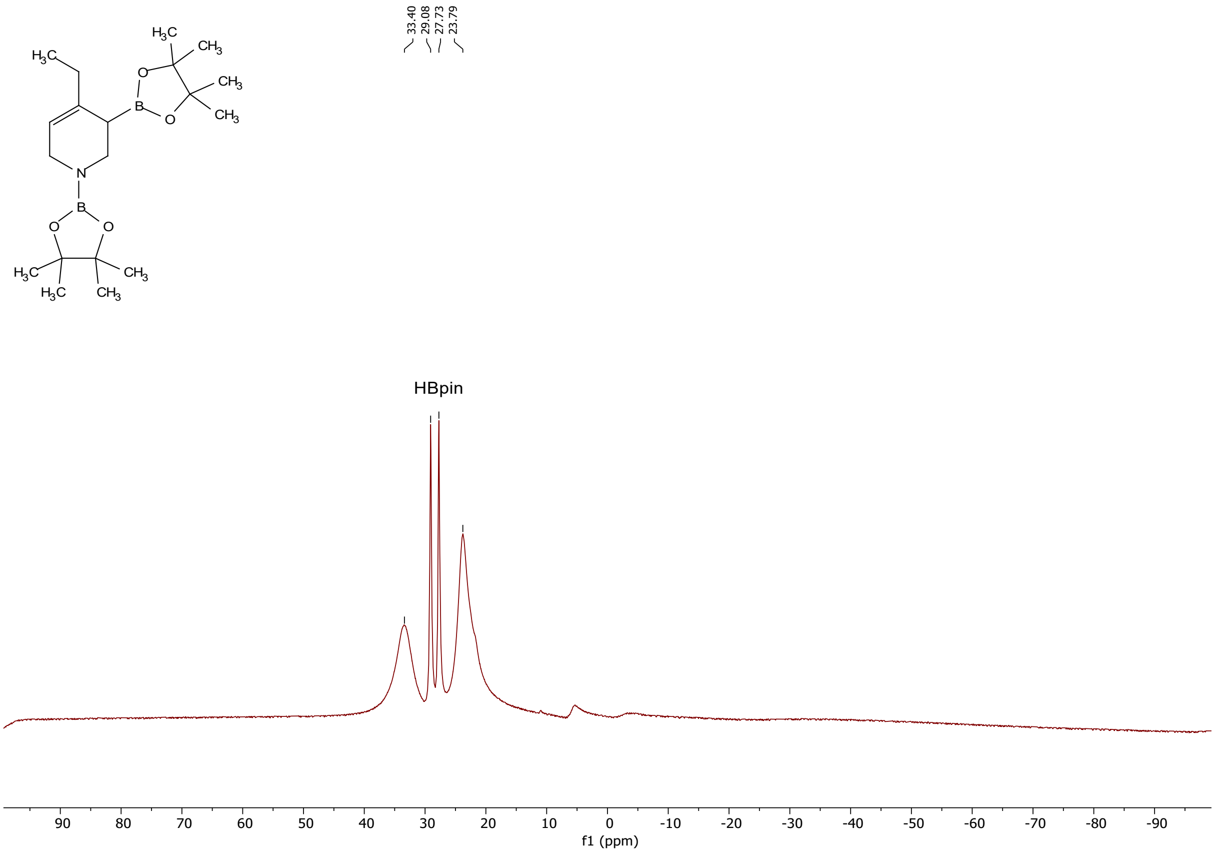

Figure 40: 128 MHz ^{11}B-NMR crude spectrum of **3d** in C_6D_6 obtained after double hydroboration of 4-ethylpyridine.

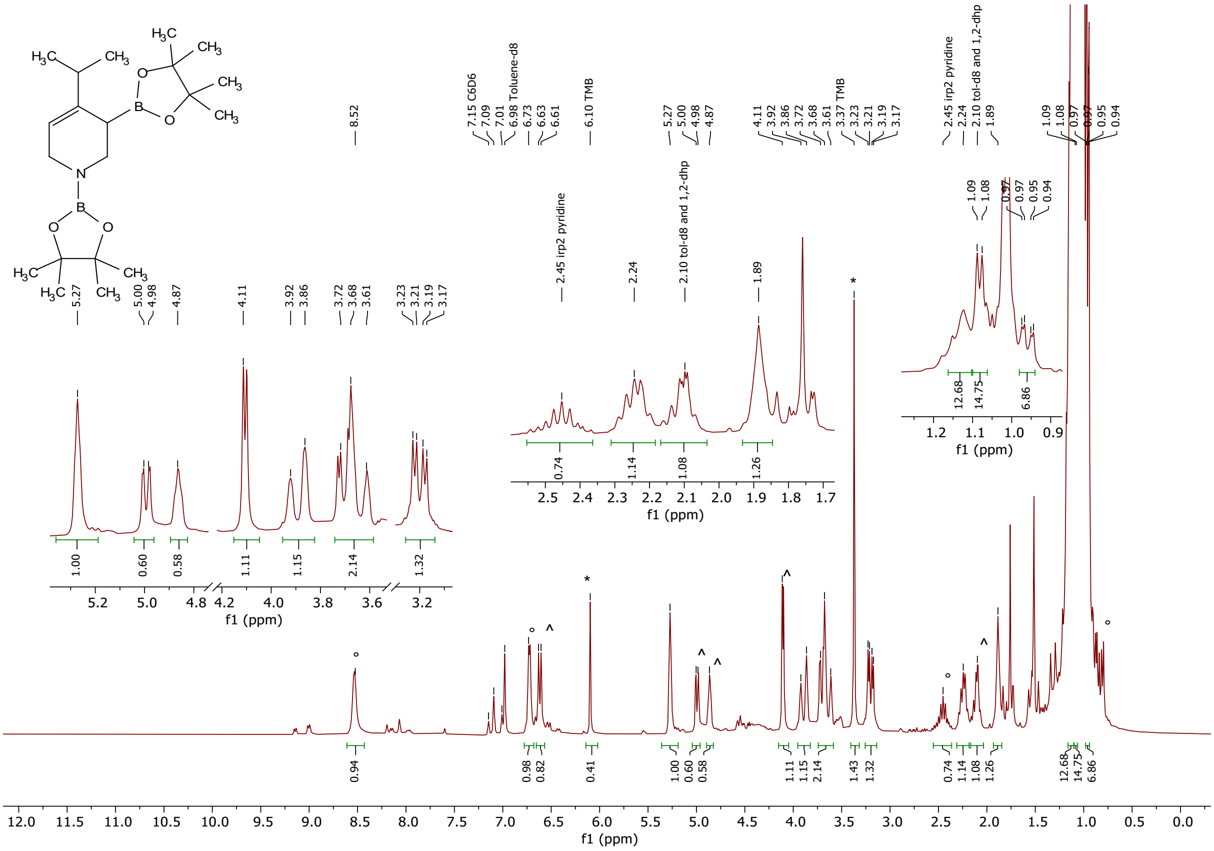

Figure 41: 300 MHz ^{1}H-NMR crude spectrum of **3r** in toluene-d_8 obtained after double hydroboration of 4-isopropylpyridine. (^) Signals of *N*-boryl 1,2-DHP. (°) Signals of 4-isopropylpyridine. (*) Internal standard 1,3,5-trimethoxybenzene (0.02 mmol).

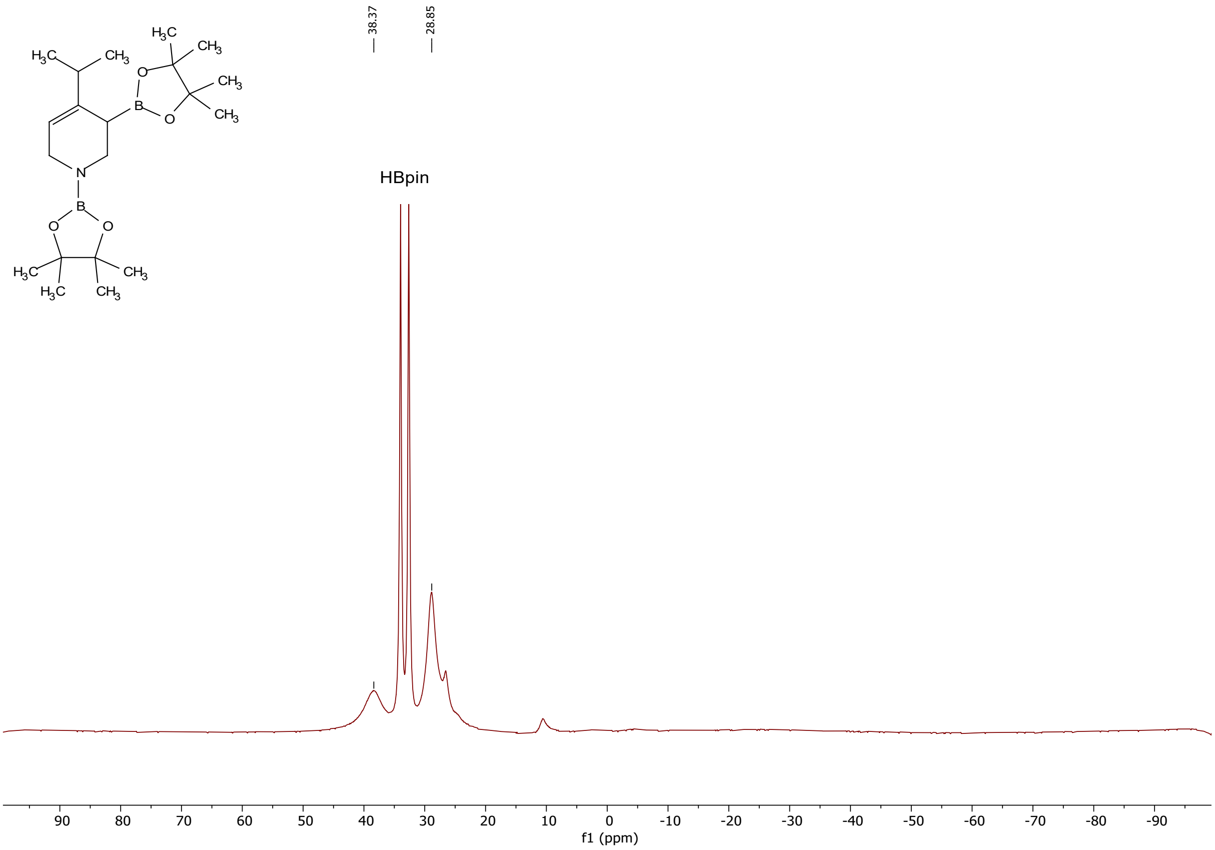

Figure 42: 128 MHz ^{11}B-NMR crude spectrum of **3r** in toluene-d_8 obtained after double hydroboration of 4-isopropylpyridine.

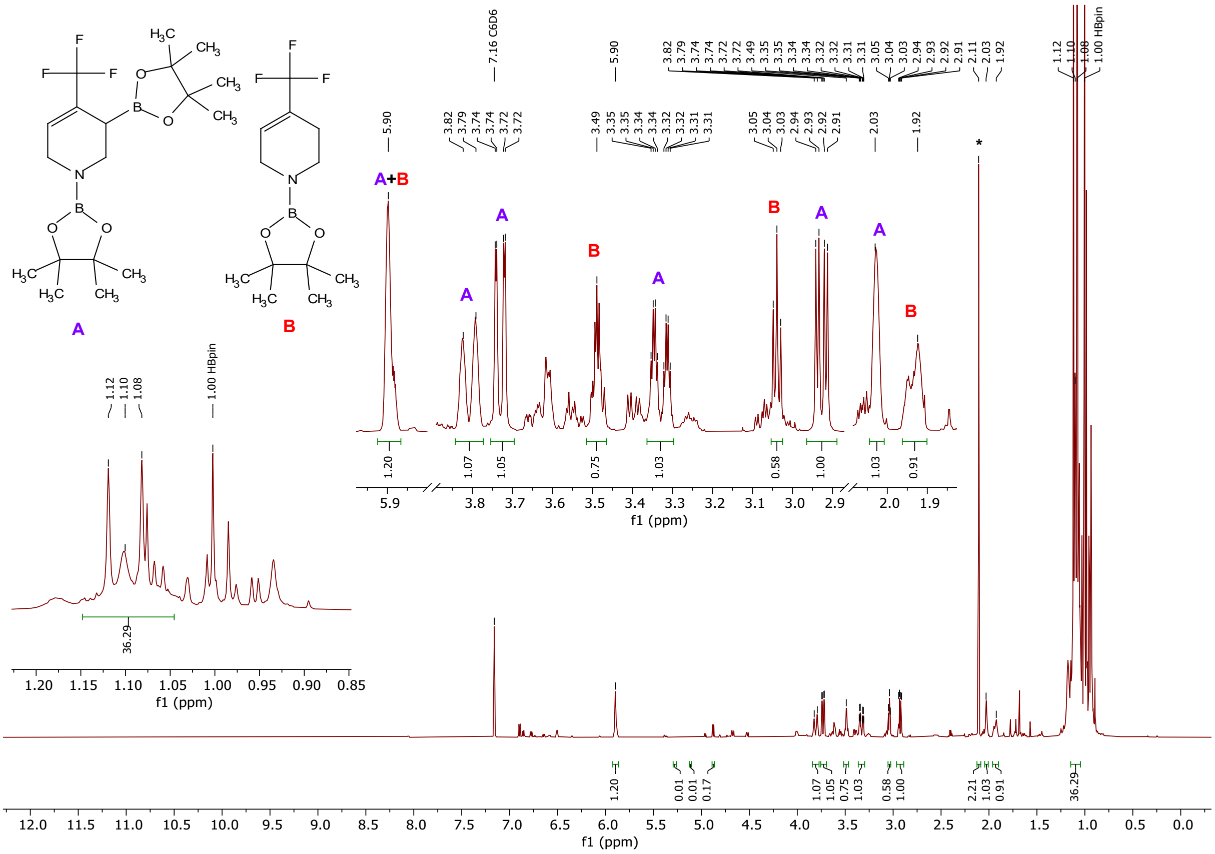

Figure 43: 600 MHz ^{1}H-NMR crude spectrum of **3f** in C_6D_6 obtained after double hydroboration of 4-(trifluoromethyl)pyridine. (*) Internal standard hexamethylbenzene (0.02 mmol).

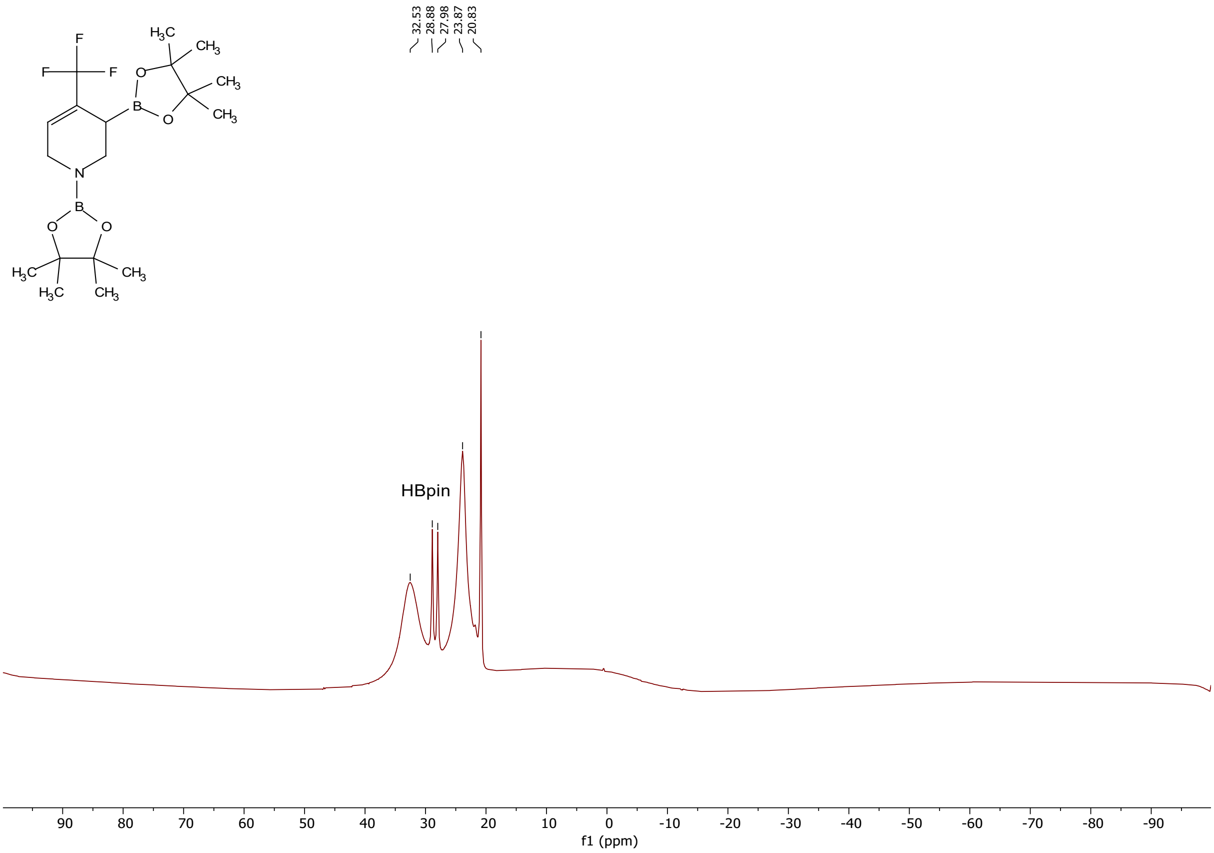

Figure 44: 193 MHz ^{11}B-NMR crude spectrum of **3f** in C_6D_6 obtained after double hydroboration of 4-(trifluoromethyl)pyridine.

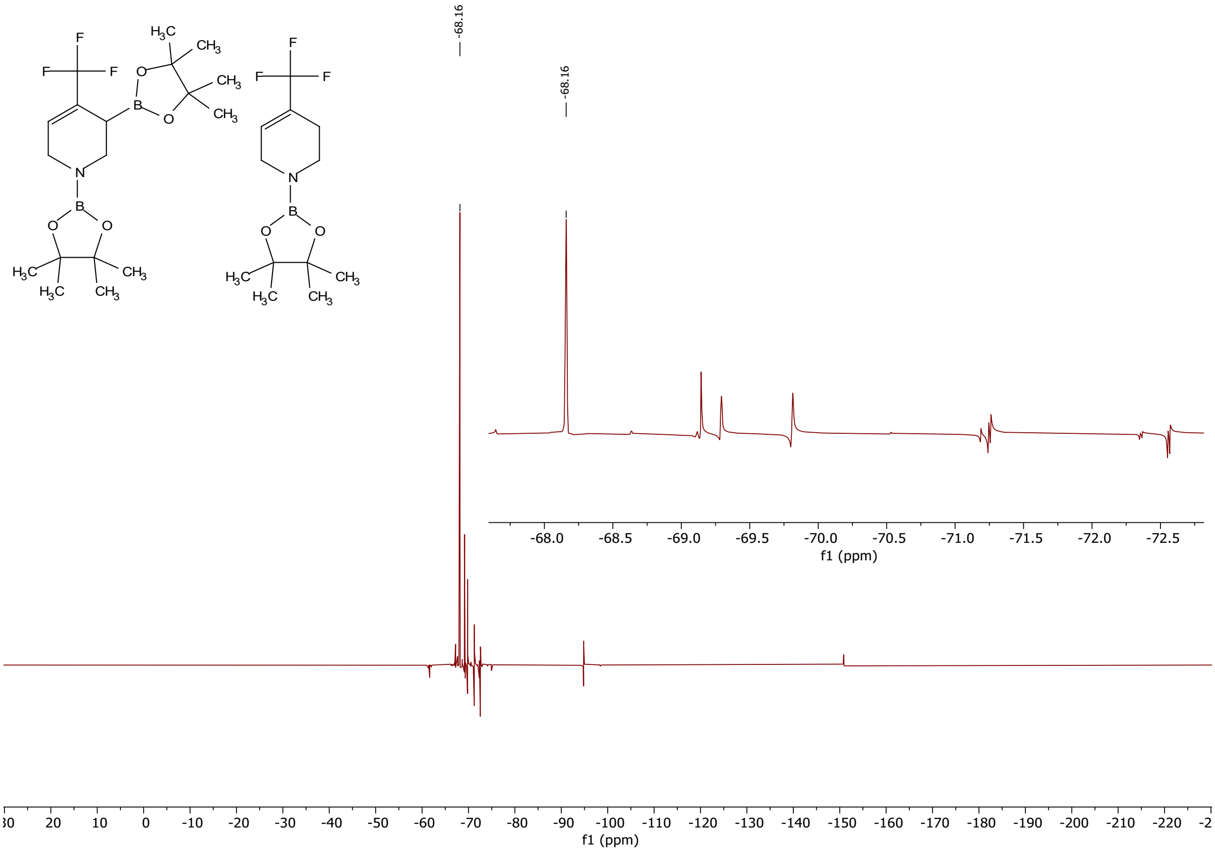

Figure 45: 565 MHz ^{19}F-NMR crude spectrum of **3f** in C_6D_6 obtained after double hydroboration of 4-(trifluoromethyl)pyridine.

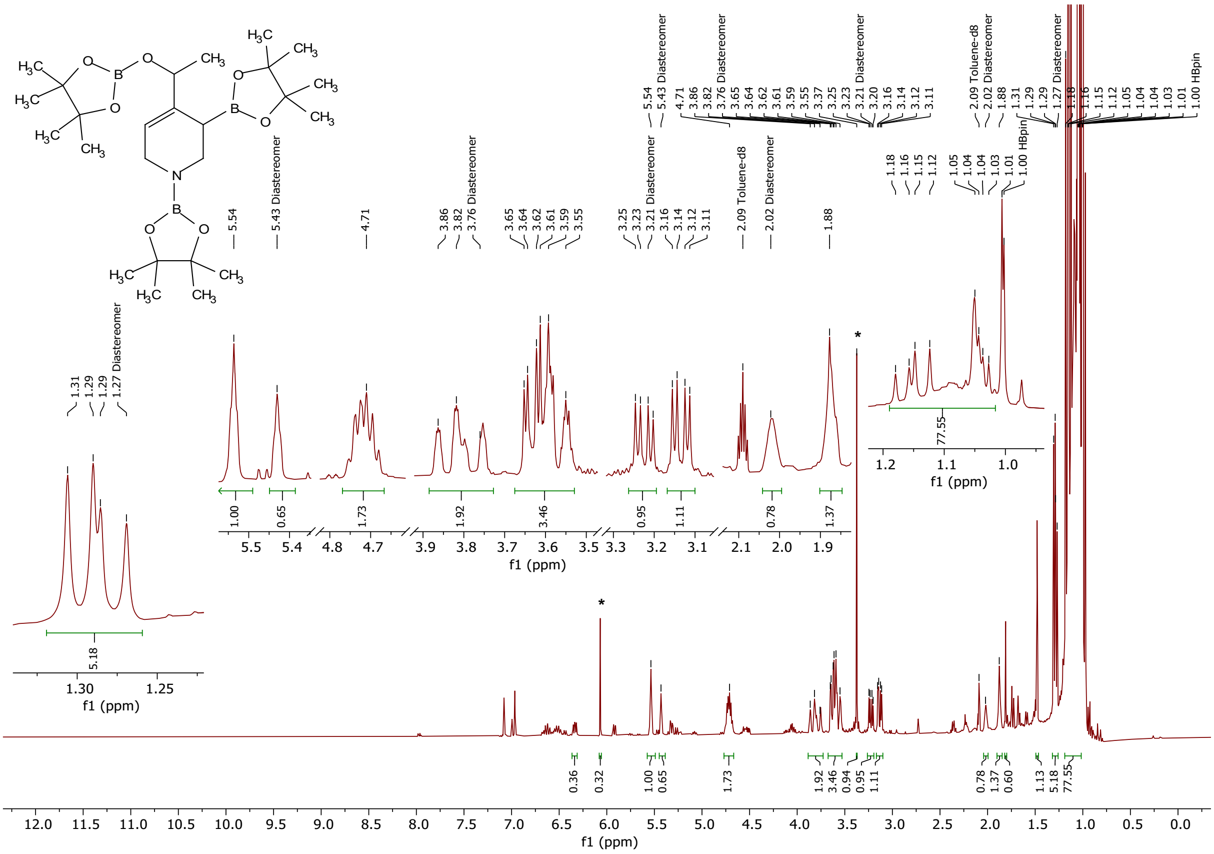

Figure 46: 400 MHz ^{1}H-NMR crude spectrum of **3h** in toluene-d_8 obtained after triple hydroboration of 4-acetylpyridine. The spectrum shows two signal sets due to the presence of diastereomers. The signals of the minor diastereomer (if separated) are marked above. (*) Internal standard 1,3,5-trimethoxybenzene (0.016 mmol).

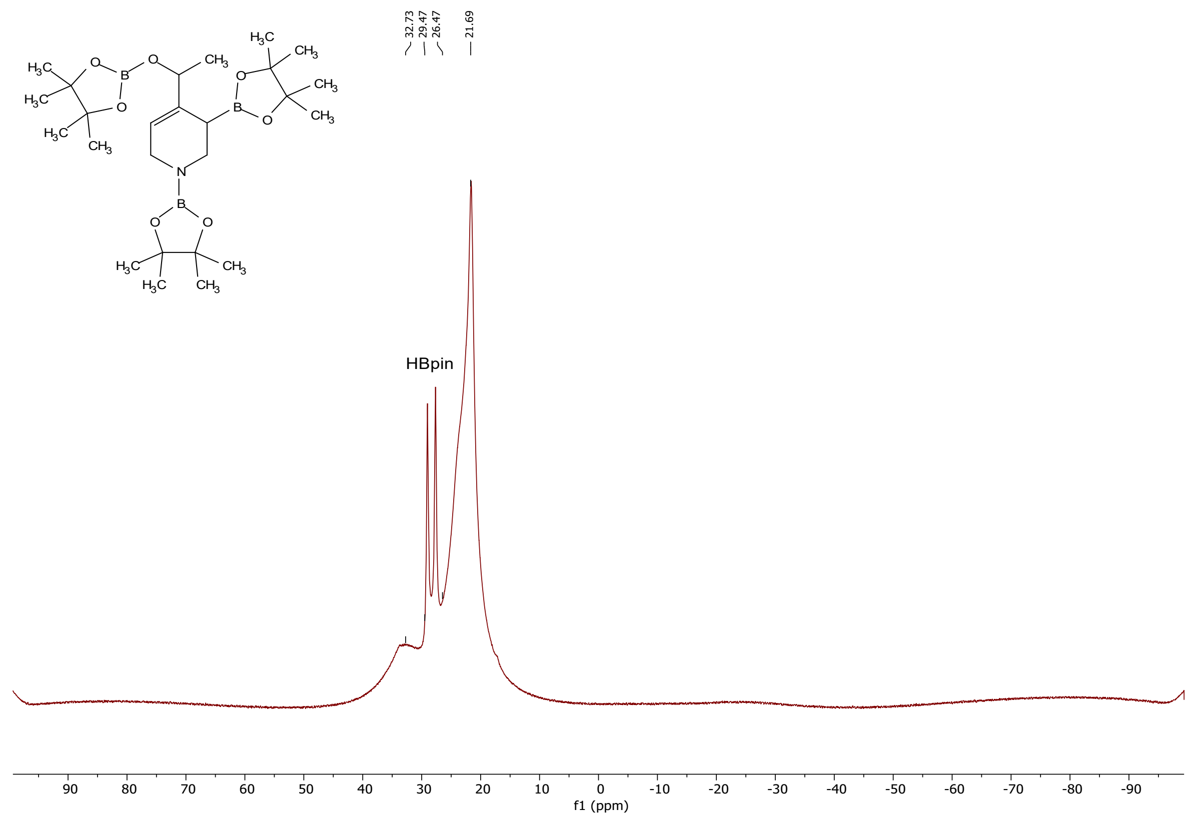

Figure 47: 128 MHz ^{11}B-NMR crude spectrum of **3h** in toluene-d_8 obtained after triple hydroboration of 4-acetyl-pyridine.

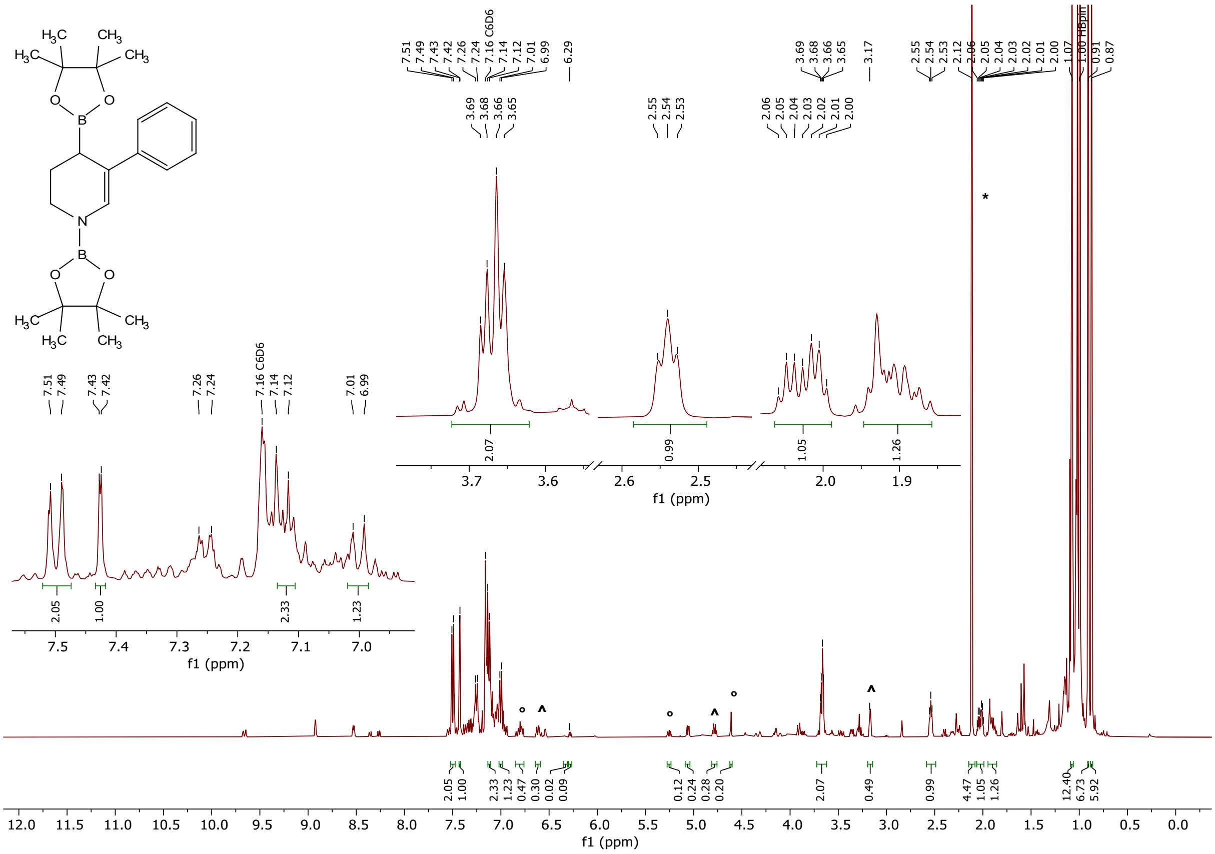

Figure 48: 400 MHz ^{1}H-NMR crude spectrum of **3i** in C_6D_6 obtained after double hydroboration of 3-phenylpyridine. (^) Signals of *N*-boryl 1,4-DHP. (°) Signals of *N*-boryl 1,2-DHP. (*) Internal standard hexamethylbenzene (0.054 mmol).

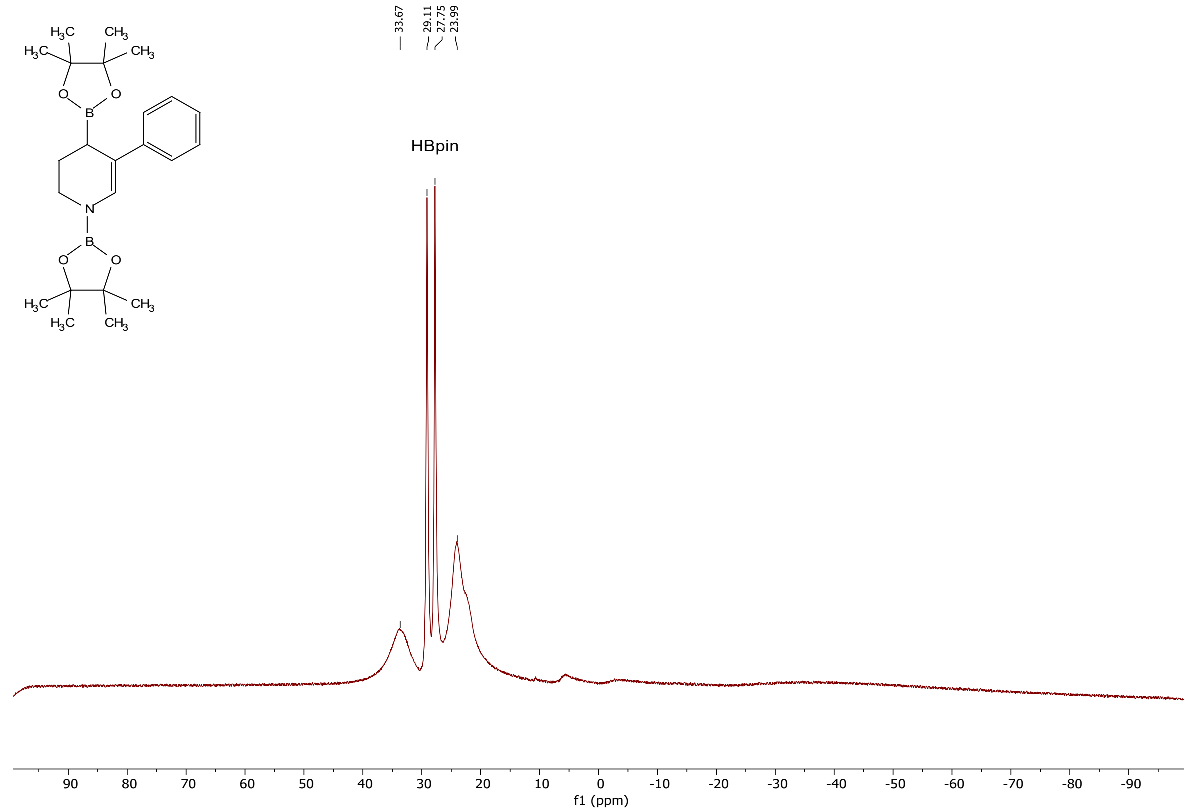

Figure 49: 128 MHz ^{11}B-NMR crude spectrum of **3i** in C_6D_6 obtained after double hydroboration of 3-phenylpyridine.

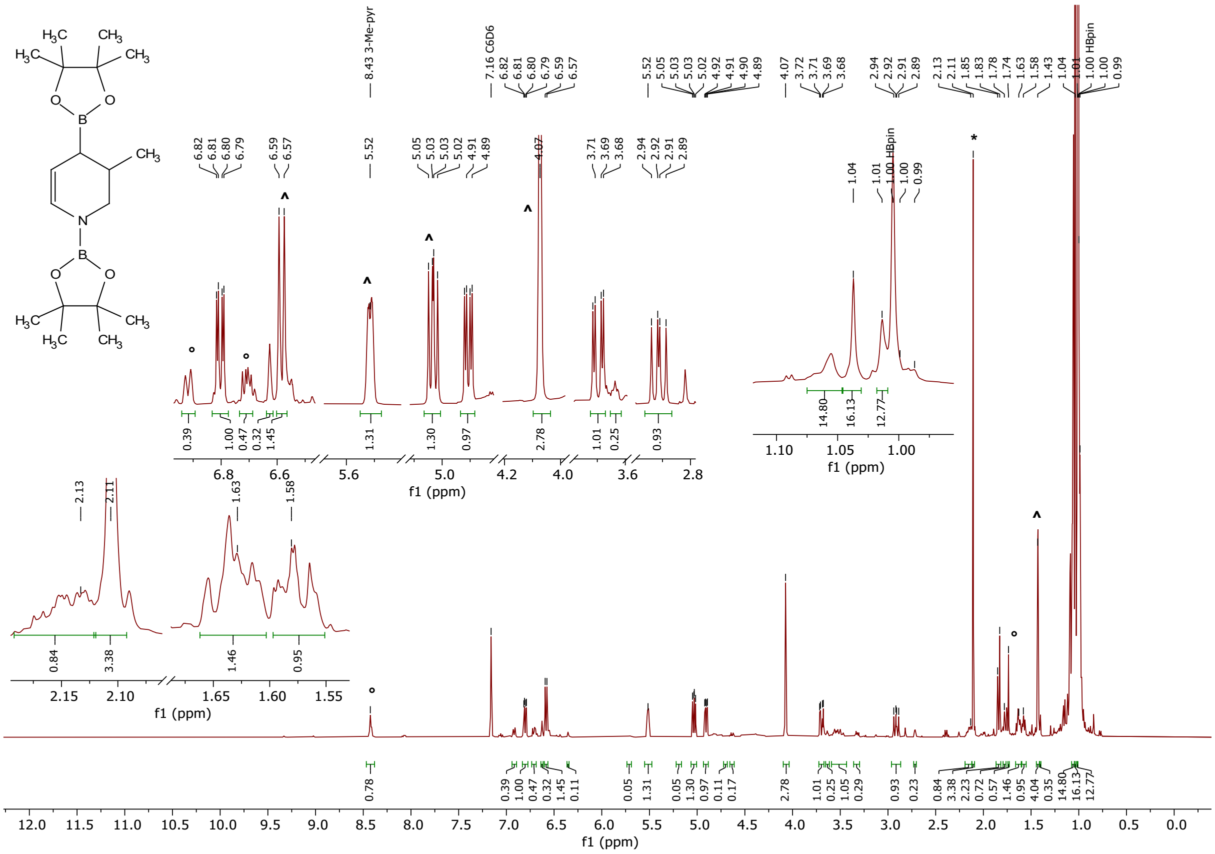

Figure 50: 400 MHz ^{1}H-NMR crude spectrum of **3k** in C_6D_6 obtained after double hydroboration of 3-methylpyridine. (^) Signals of *N*-boryl 1,2-DHP. (°) Signals of 3-methylpyridine. (*) Internal standard hexamethylbenzene (0.02 mmol).

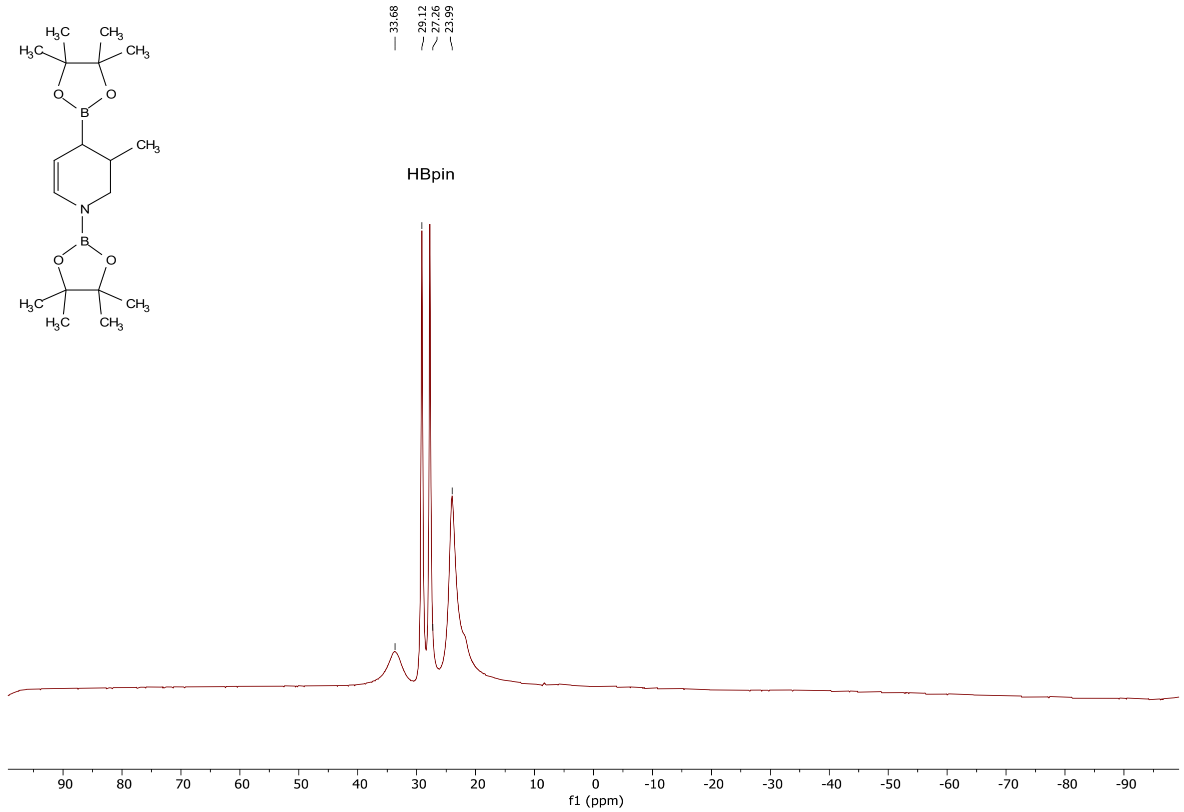

Figure 51: 128 MHz ^{11}B-NMR crude spectrum of **3k** in C_6D_6 obtained after double hydroboration of 3-methyl-pyridine.

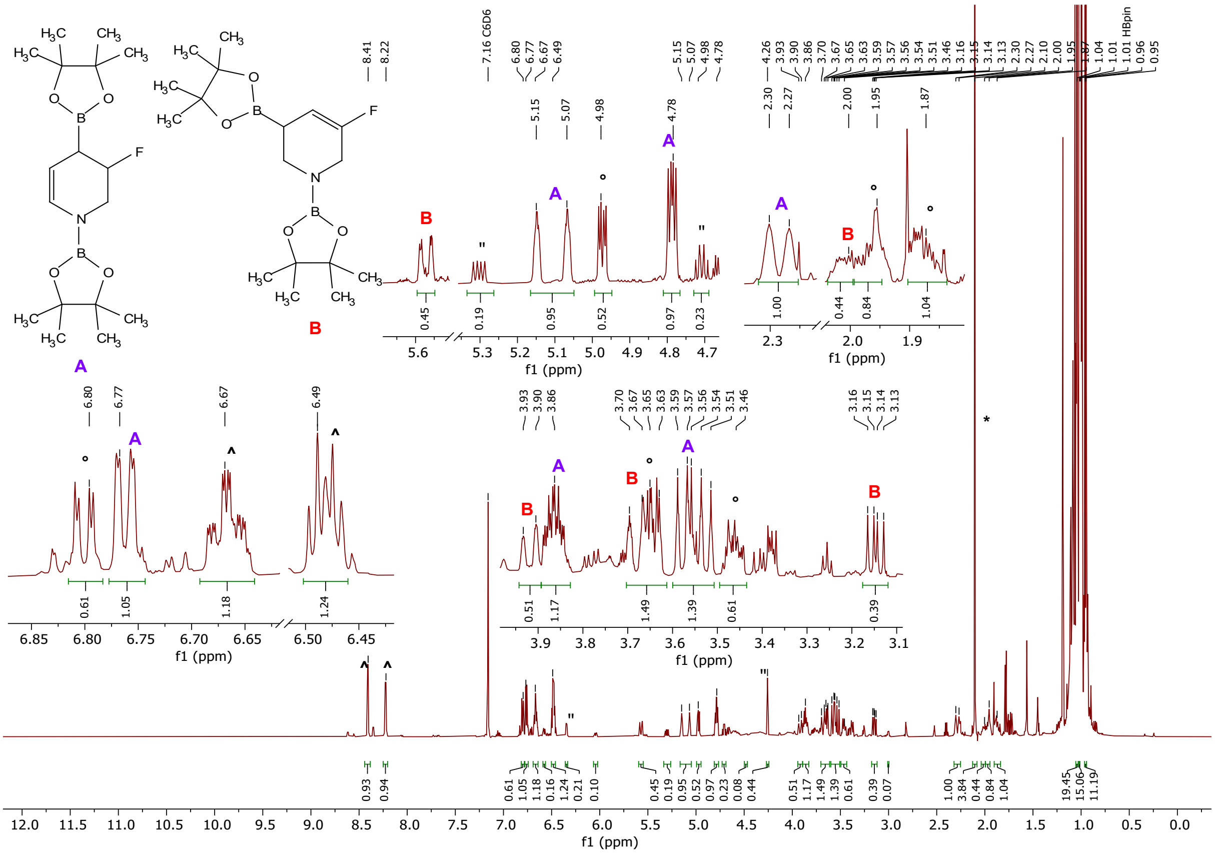

Figure 52: 600 MHz ^{1}H-NMR crude spectrum of **3j** and **3j’** in C_6D_6 obtained after double hydroboration of 3-fluoropyridine. (^) Signals of 3-fluoropyridine. (°) Signals of defluorination product **3c**. (“) Signals of *N*-boryl 1,2-DHP (*) Internal standard hexamethylbenzene (0.02 mmol).

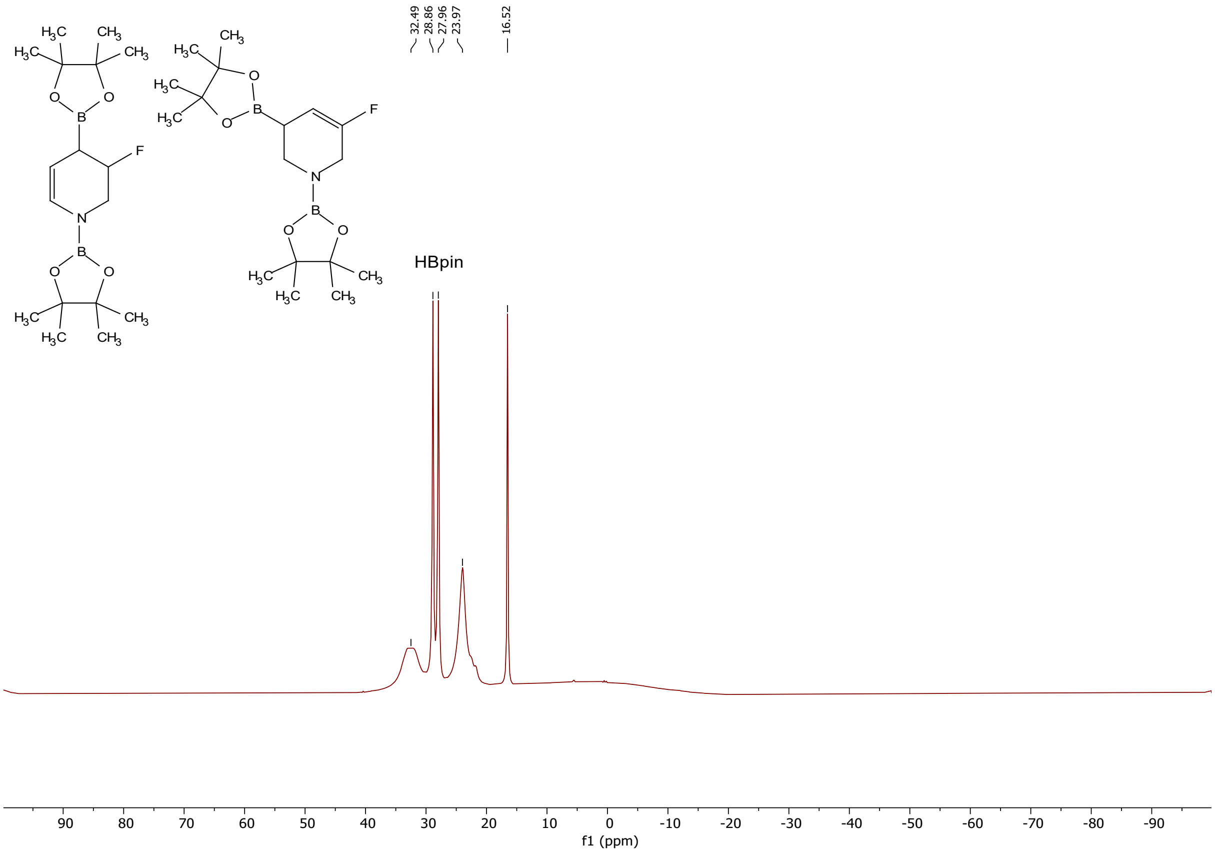

Figure 53: 193 MHz ^{11}B-NMR crude spectrum of **3j** and **3j'** in C_6D_6 obtained after double hydroboration of 3-fluoropyridine.

11.3 Experimental Spectra of Borylated *N*-Acetyl Tetrahydropyridines

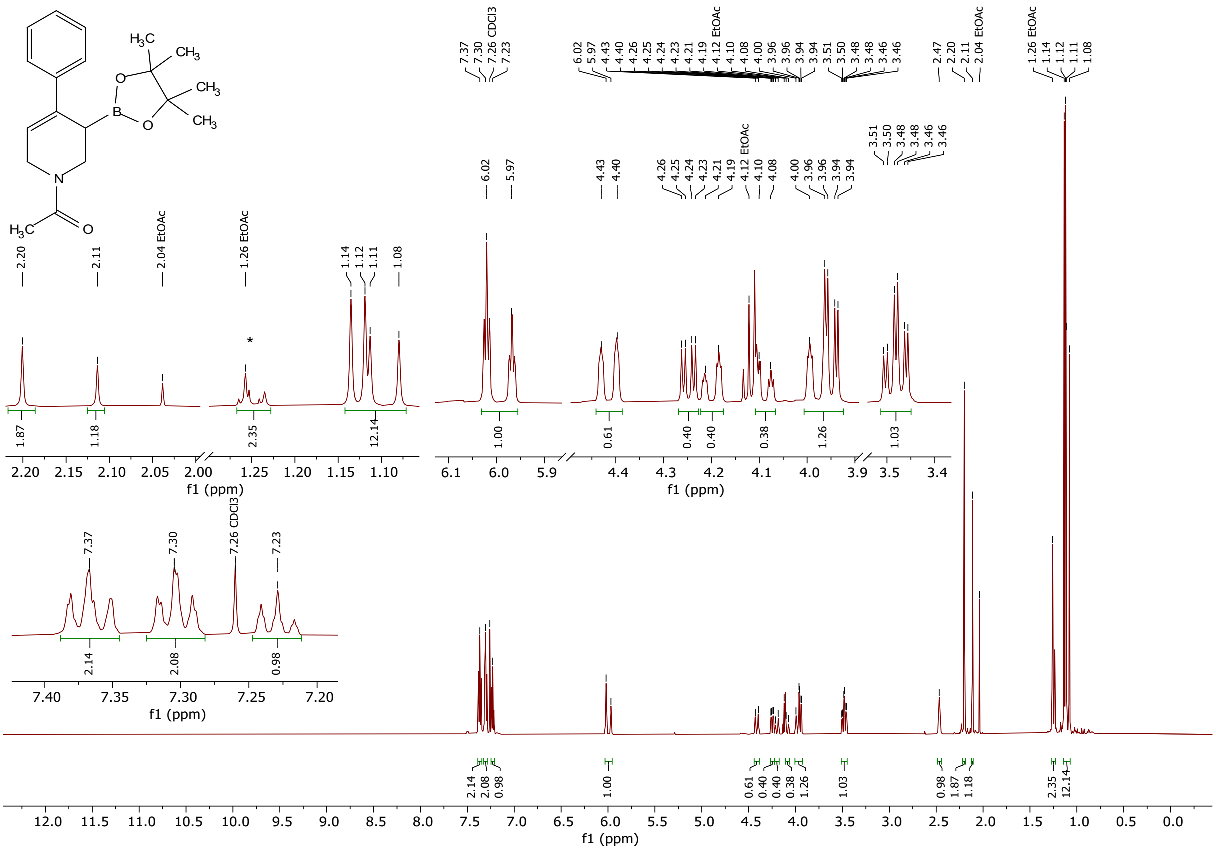

Figure 54: 600 MHz ^{1}H-NMR spectrum of **4b** in $CDCl_3$. The spectrum shows two signal sets due to the presence of rotamers. Signals of residual EtOAc are marked above. (*) $B_x pin_y$ impurity.

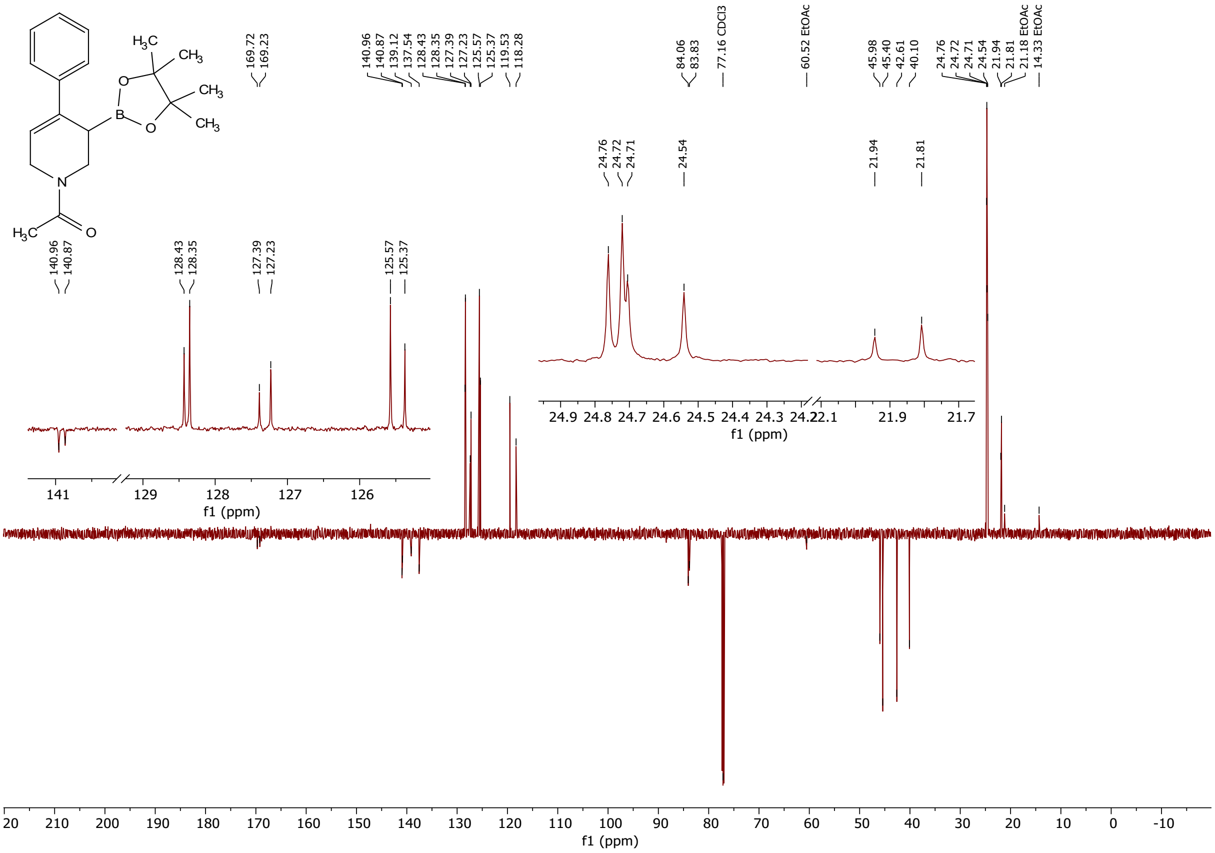

Figure 55: 151 MHz ^{13}C-DEPTQ-NMR spectrum of **4b** in $CDCl_3$. The spectrum shows two signal sets due to the presence of rotamers. Signals of residual EtOAc are marked above.

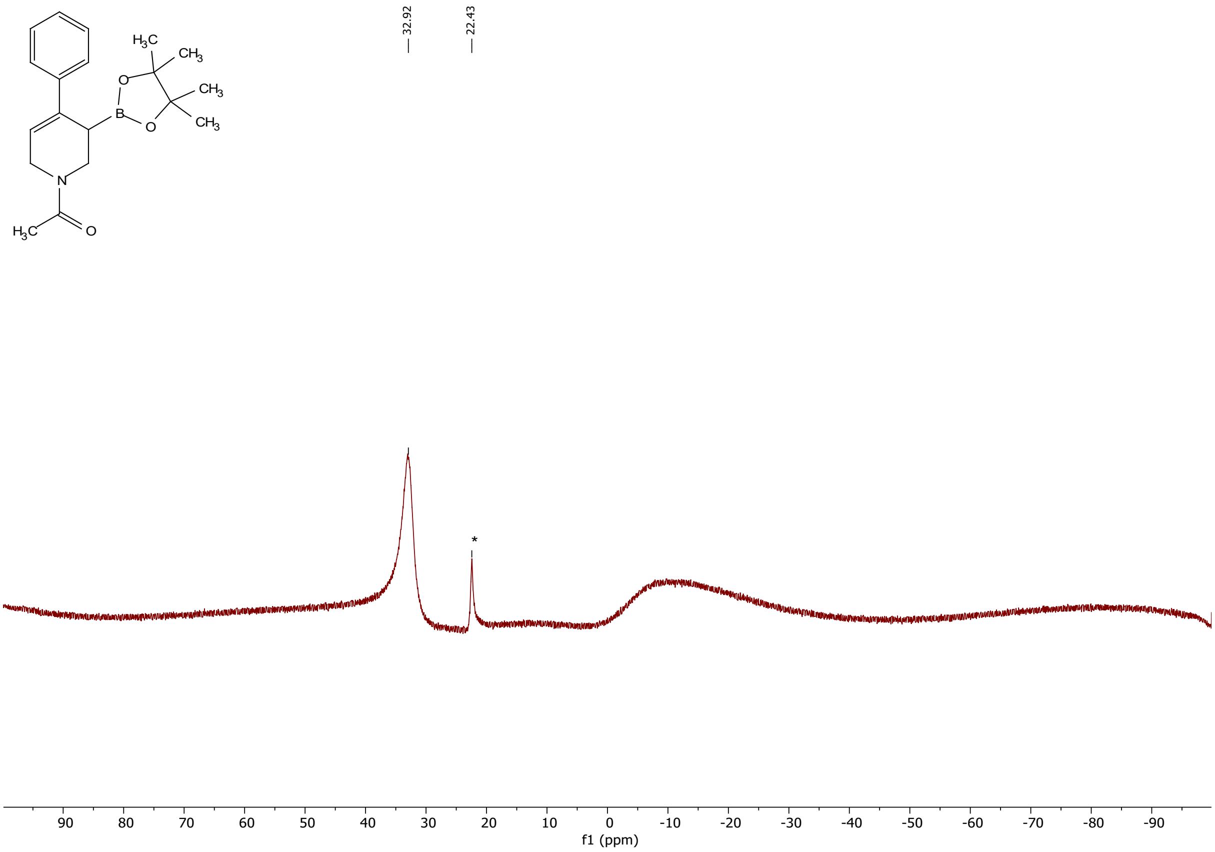

Figure 56: 193 MHz ^{11}B-NMR spectrum of **4b** in $CDCl_3$. (*) $B_x$pin$_y$ impurity.

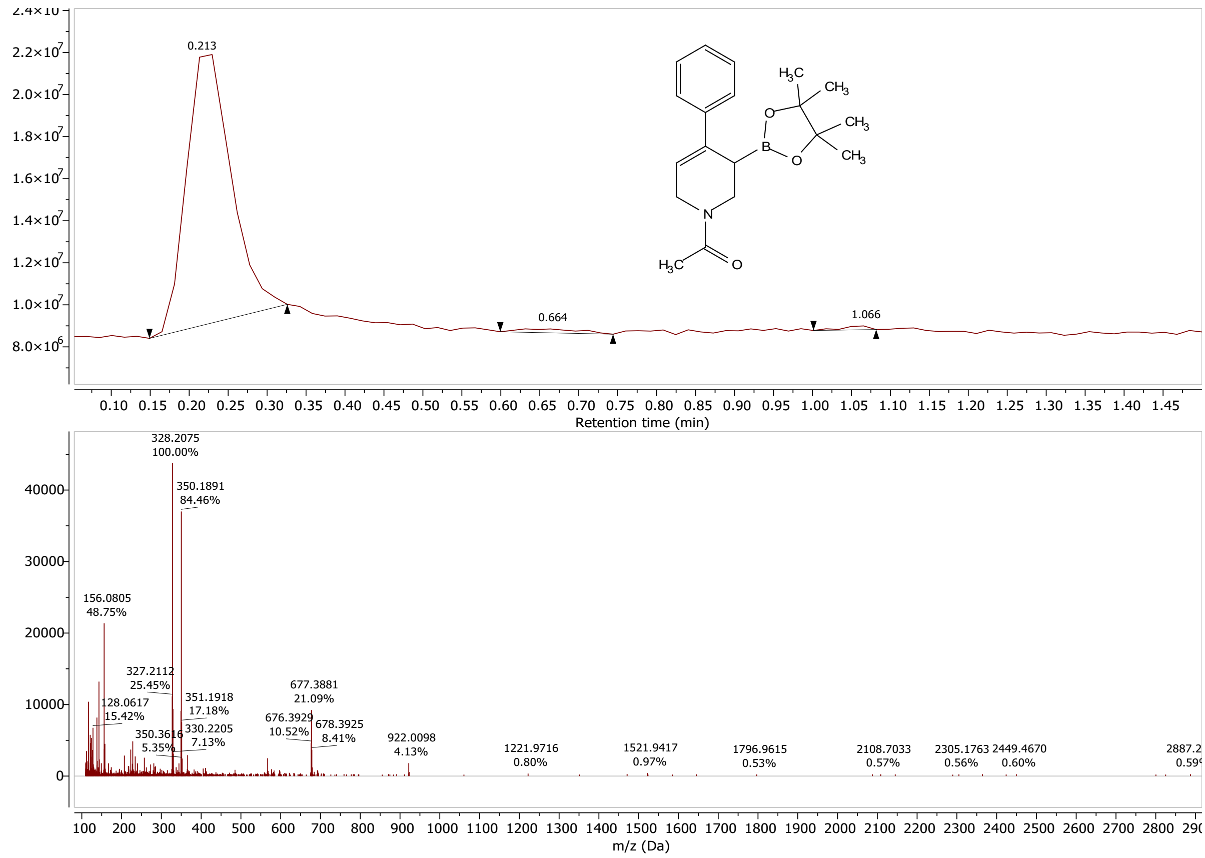

Figure 57: HRMS-ESI spectrum of **4b** measured in positive ion mode.

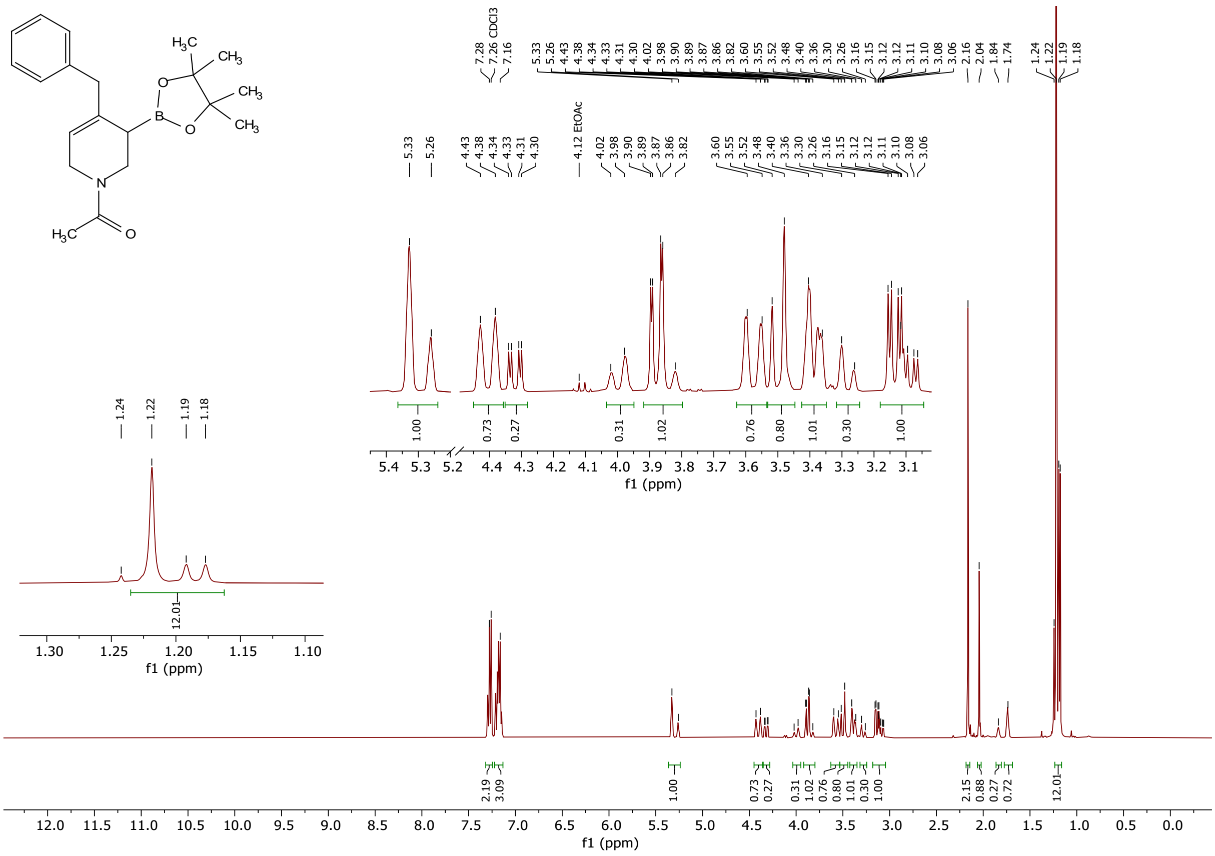

Figure 58: 400 MHz ^{1}H-NMR spectrum of **4e** in $CDCl_3$. The spectrum shows two signal sets due to the presence of rotamers.

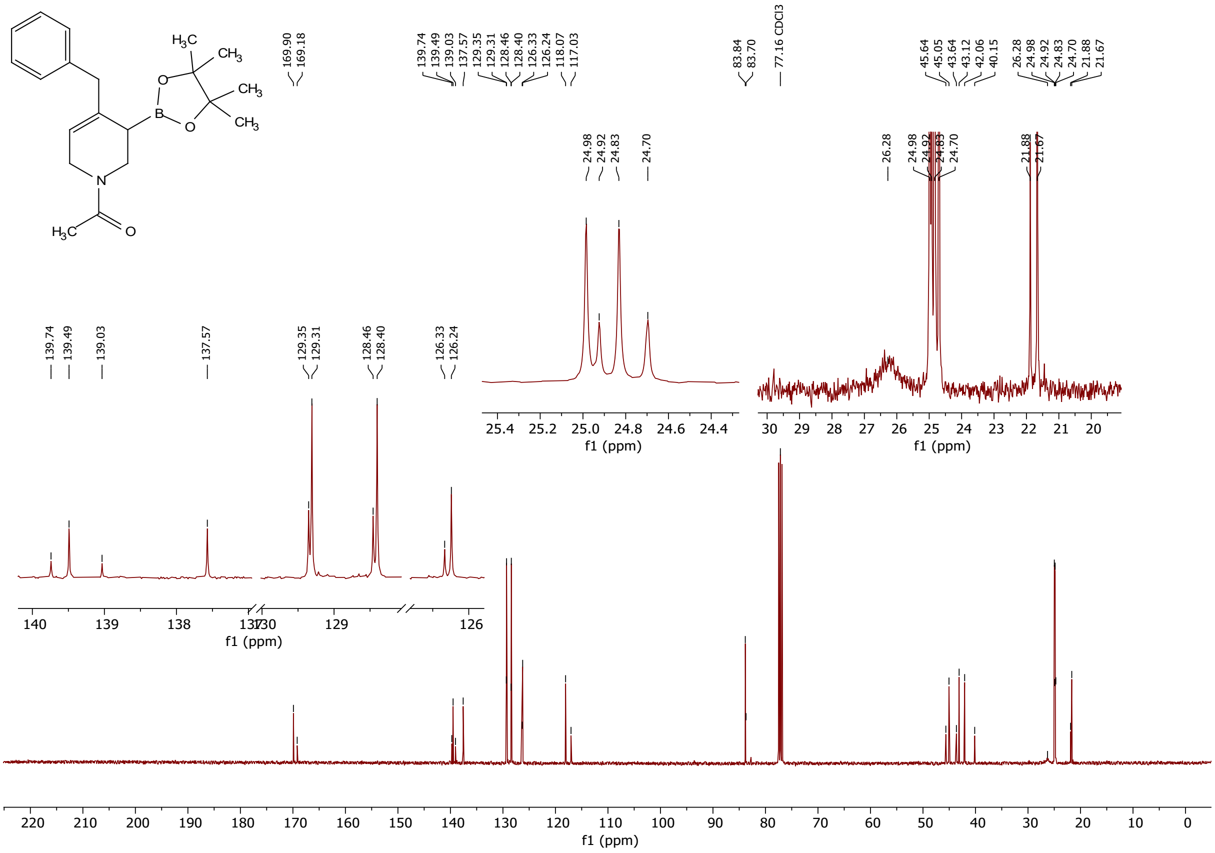

Figure 59: 101 MHz $^{13}C\{^{1}H\}$-NMR spectrum of **4e** in $CDCl_3$. The spectrum shows two signal sets due to the presence of rotamers.

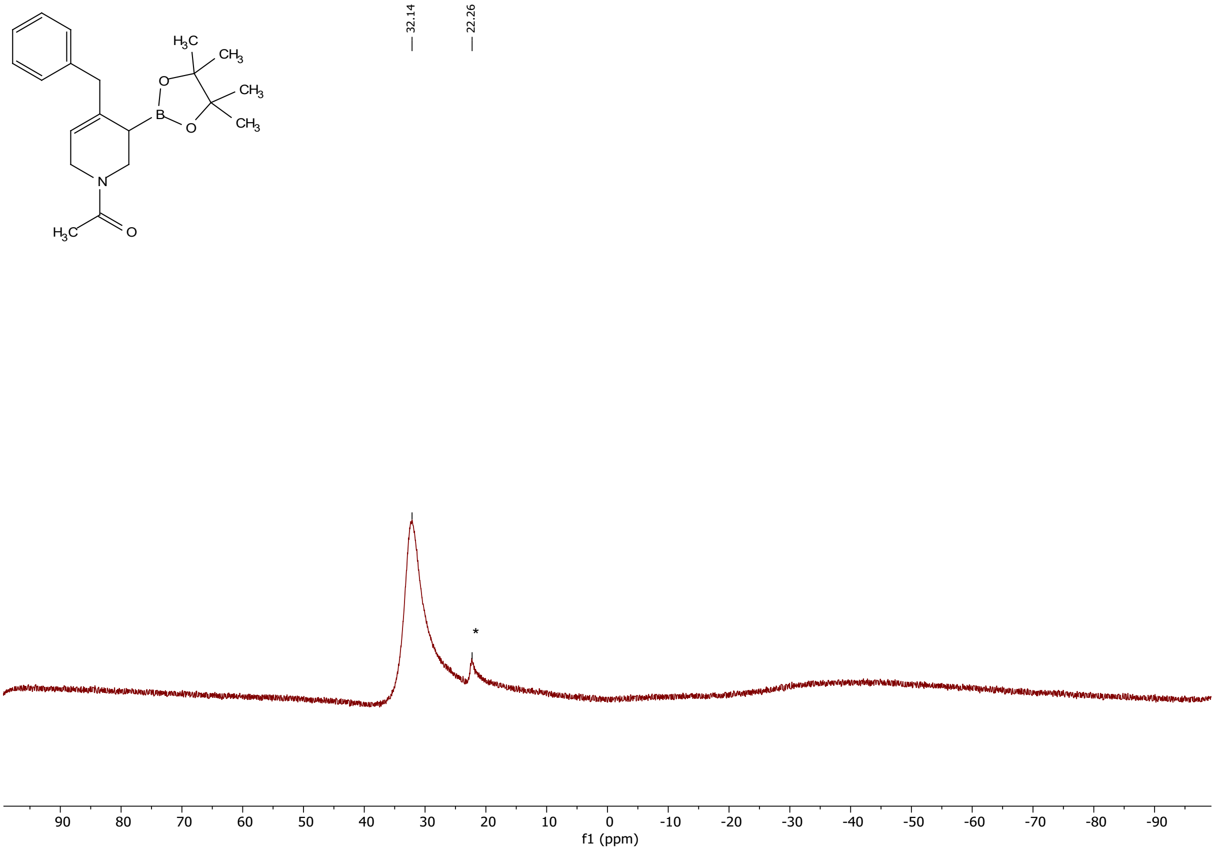

Figure 60: 128 MHz ^{11}B-NMR spectrum of **4e** in $CDCl_3$. (*) $B_x pin_y$ impurity.

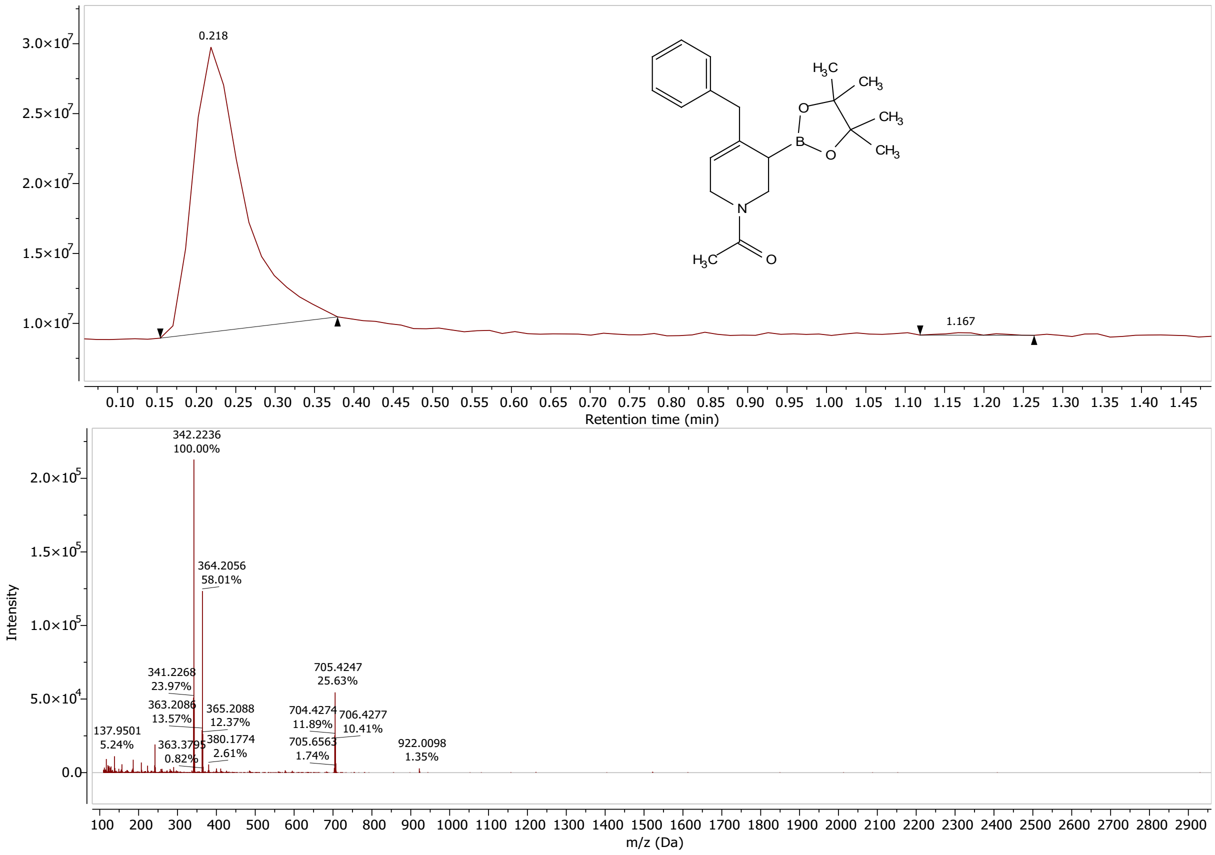

Figure 61: HRMS-ESI spectrum of **4e** measured in positive ion mode.

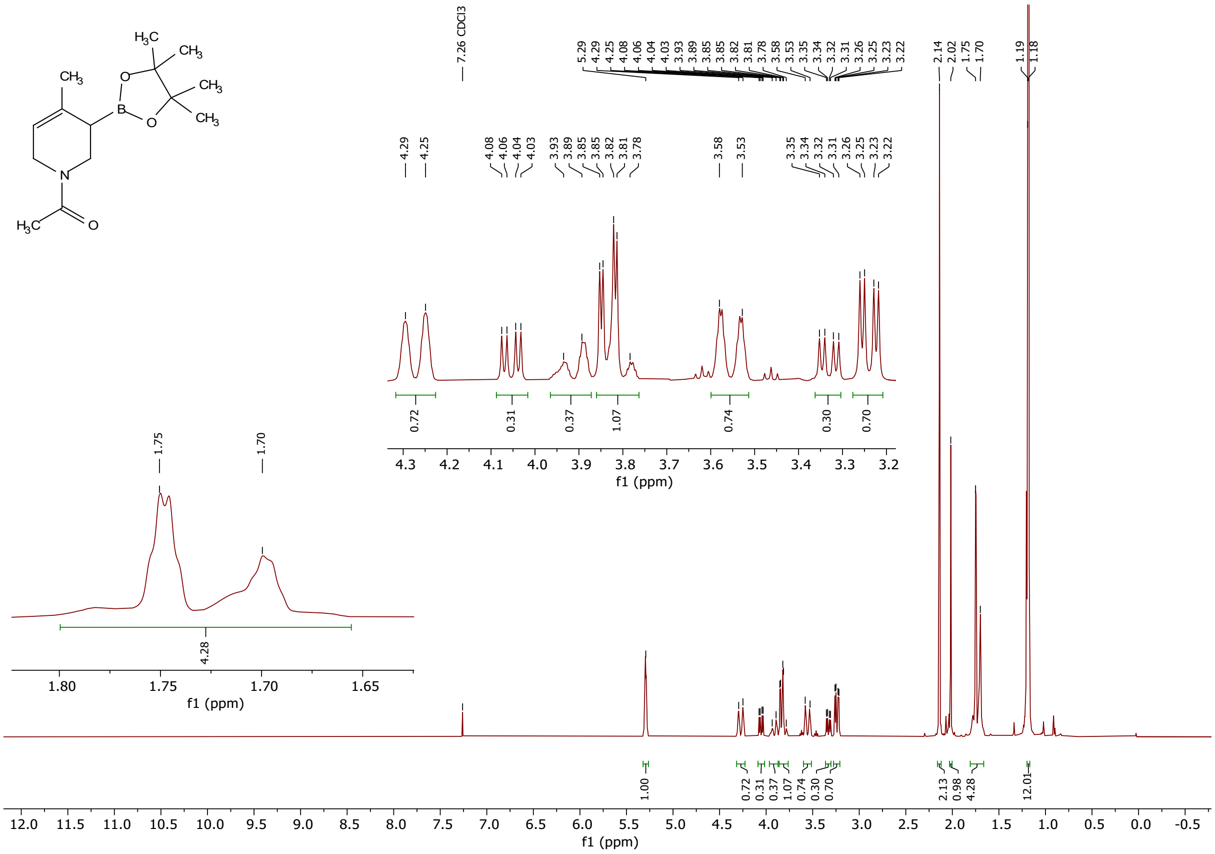

Figure 62: 400 MHz ^{1}H-NMR spectrum of **4a** in $CDCl_3$. The spectrum shows two signal sets due to the presence of rotamers.

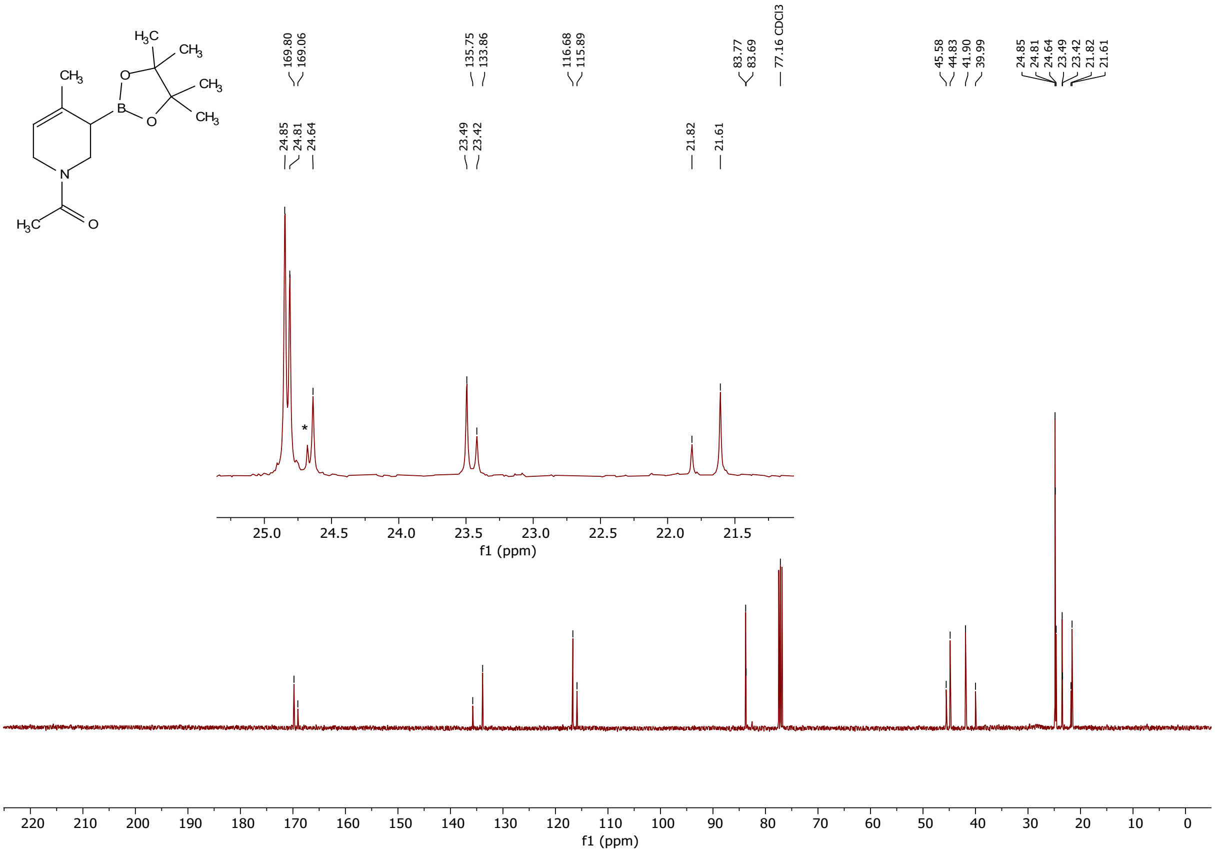

Figure 63: 101 MHz $^{13}C\{^{1}H\}$-NMR spectrum of **4a** in $CDCl_3$. The spectrum shows two signal sets due to the presence of rotamers. (*) B_xpin_y impurity.

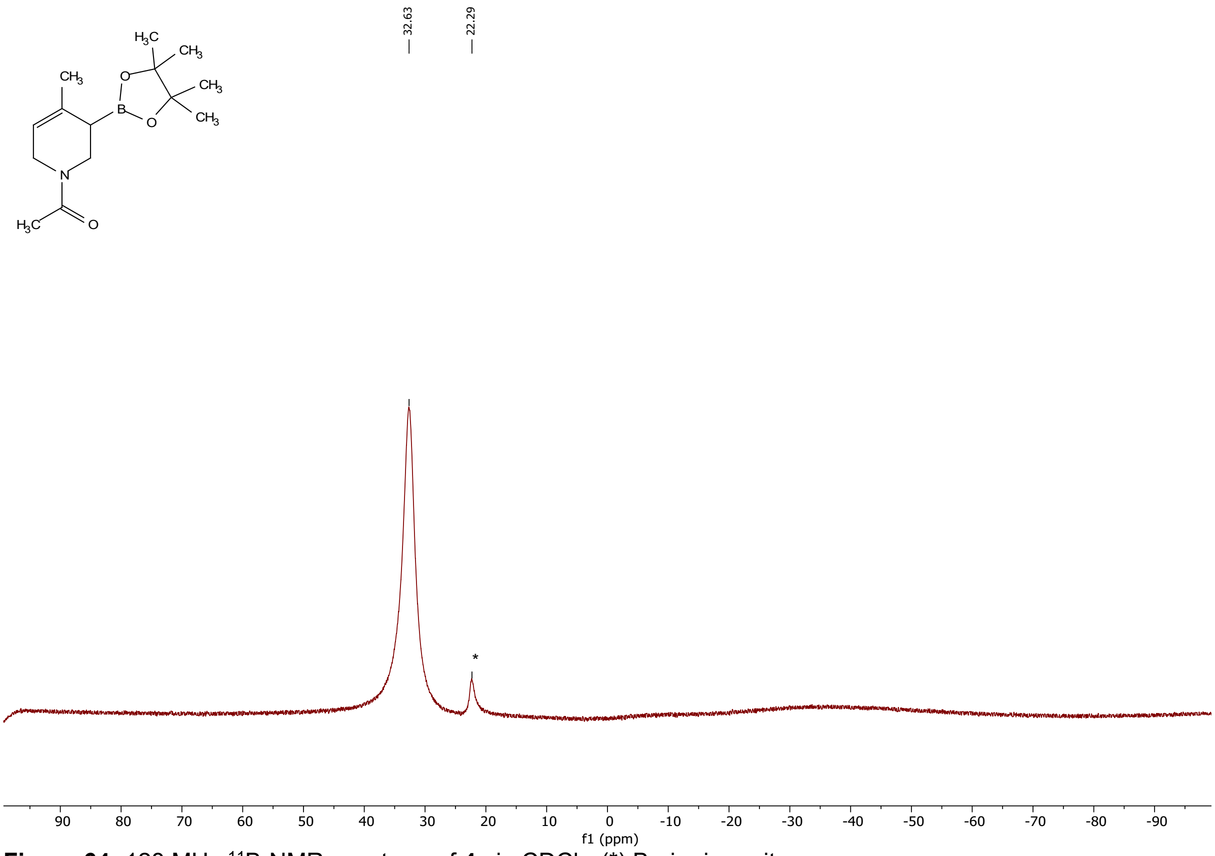

Figure 64: 128 MHz ^{11}B-NMR spectrum of **4a** in $CDCl_3$. (*) B_xpin_y impurity.

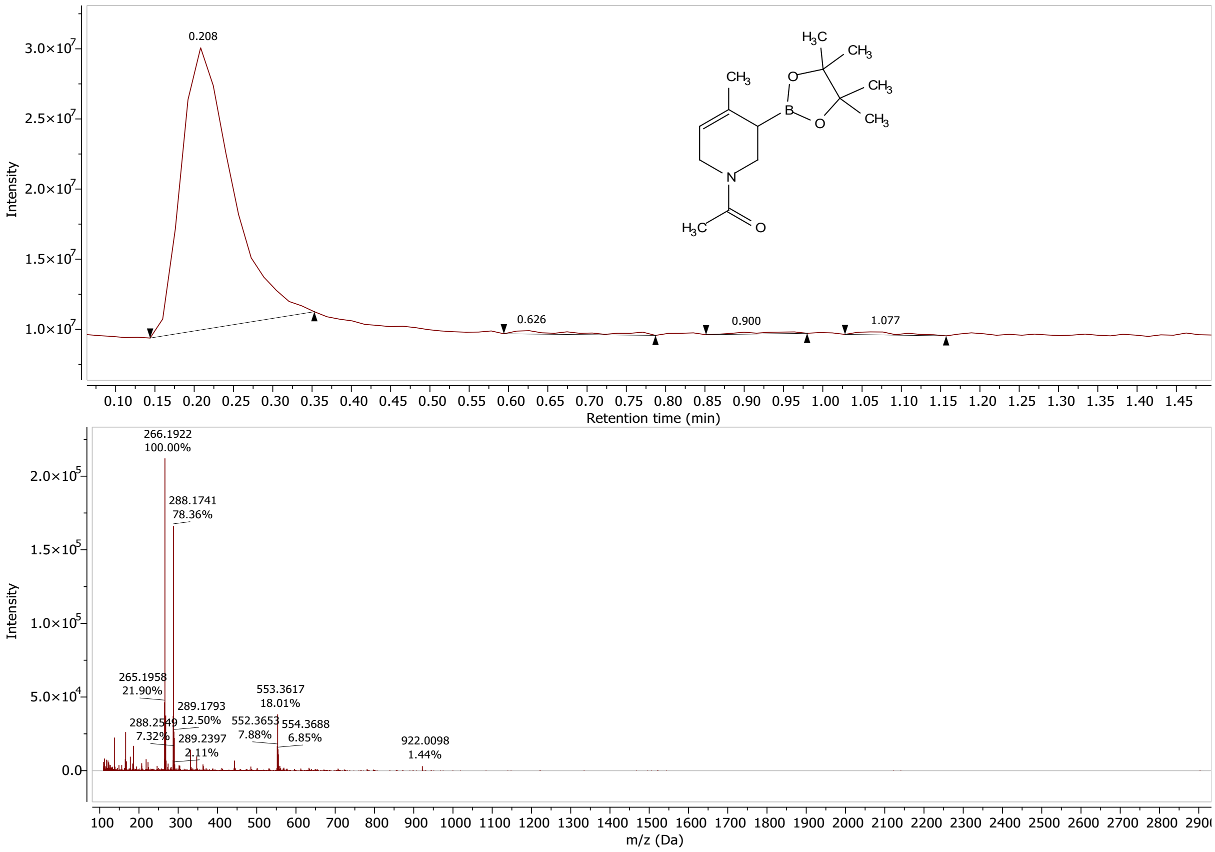

Figure 65: HRMS-ESI spectrum of **4a** measured in positive ion mode.

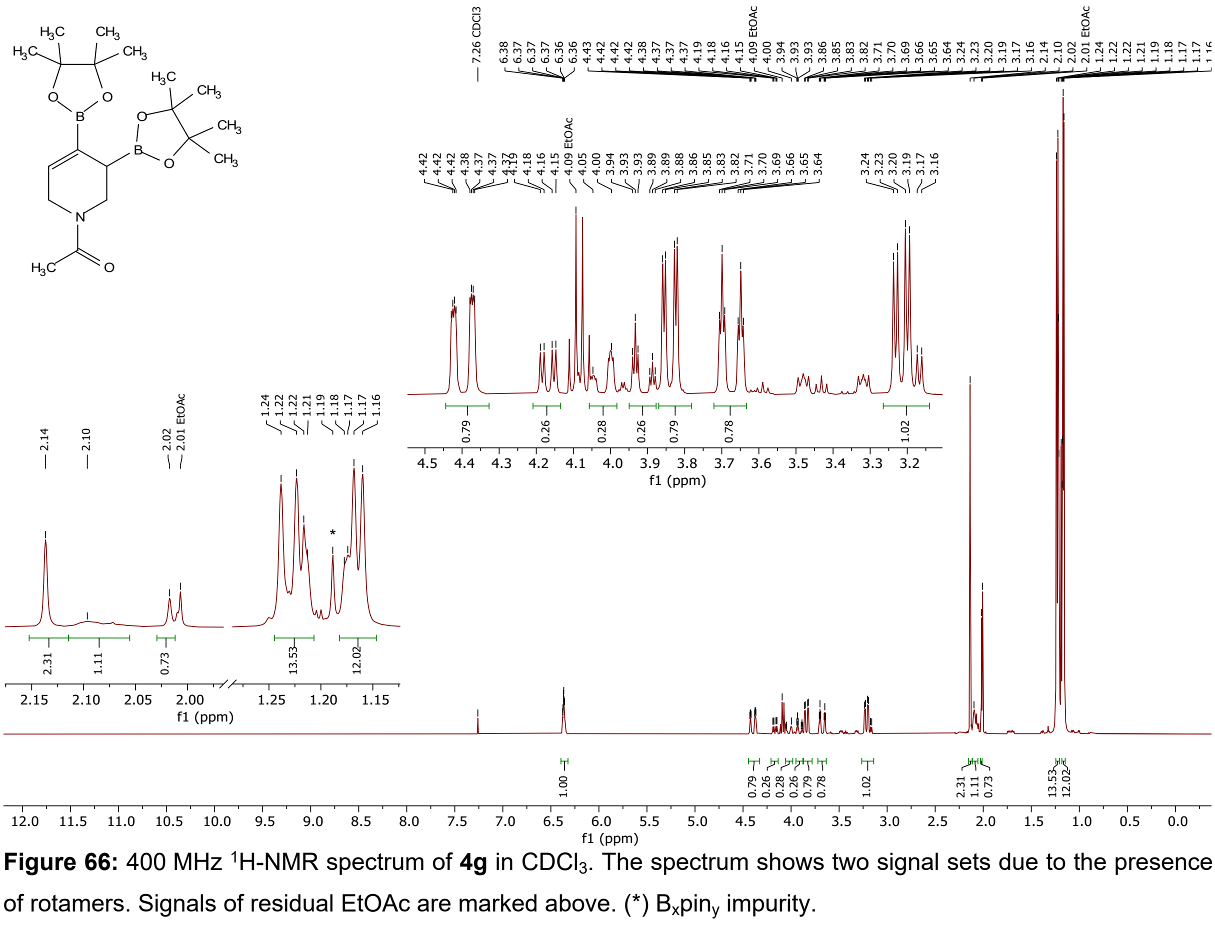

Figure 66: 400 MHz ^{1}H-NMR spectrum of **4g** in $CDCl_3$. The spectrum shows two signal sets due to the presence of rotamers. Signals of residual EtOAc are marked above. (*) B_xpin_y impurity.

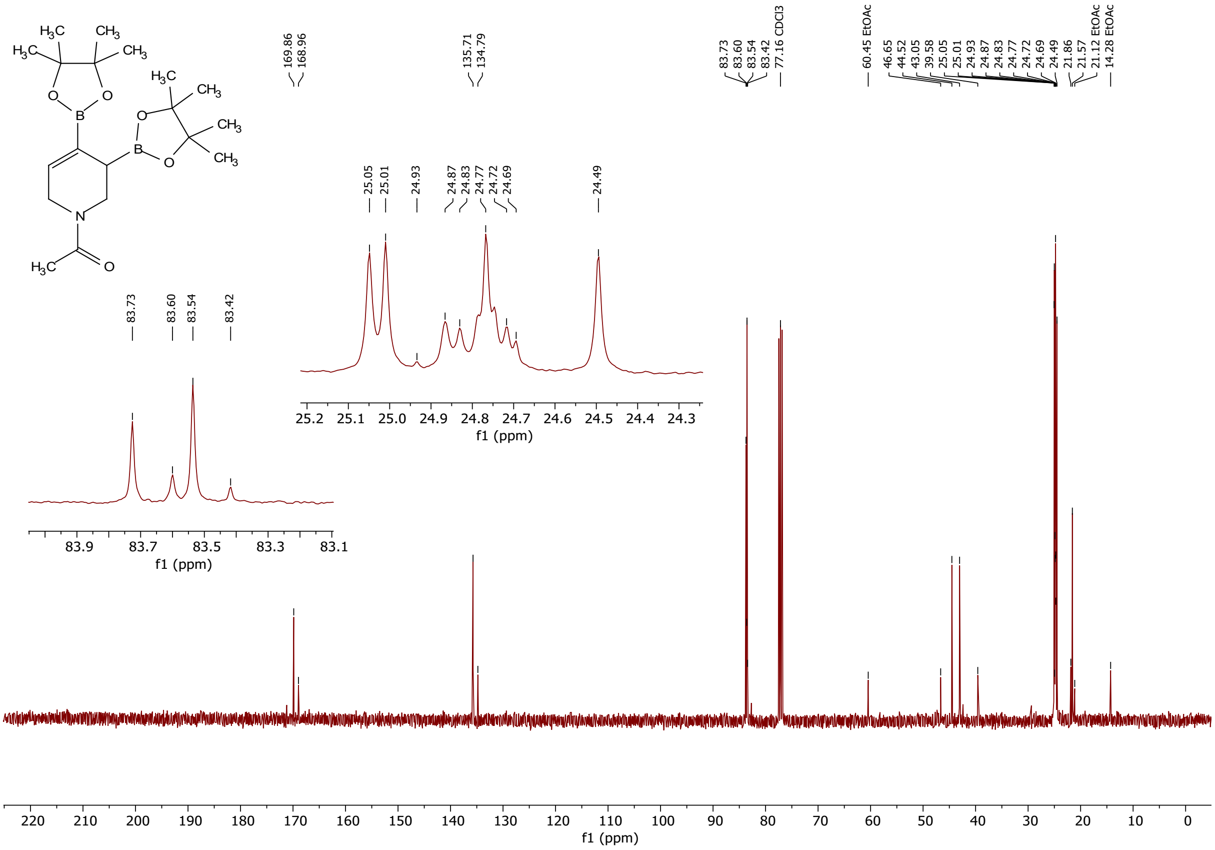

Figure 67: 101 MHz $^{13}C\{^{1}H\}$-NMR spectrum of **4g** in $CDCl_3$. The spectrum shows two signal sets due to the presence of rotamers. Signals of residual EtOAc are marked above.

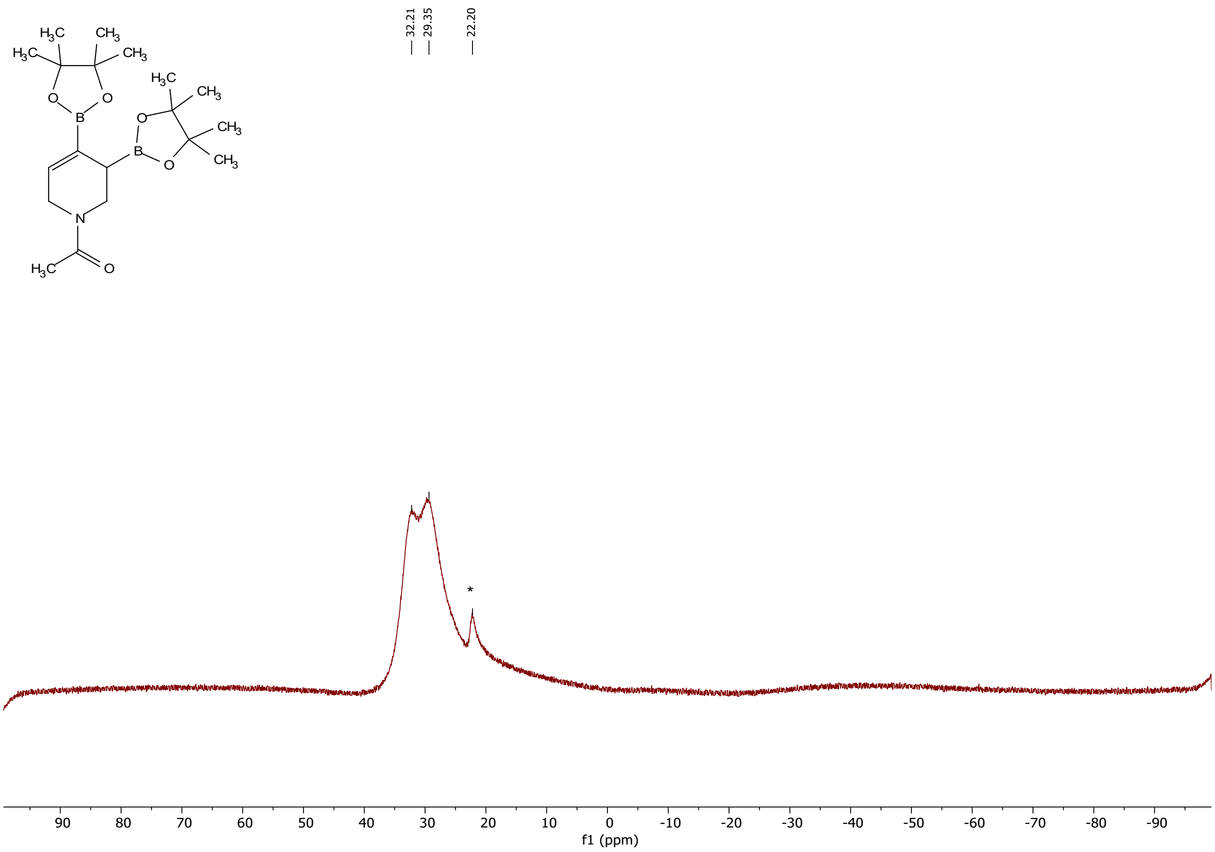

Figure 68: 128 MHz ^{11}B-NMR spectrum of **4g** in $CDCl_3$. (*) $B_x pin_y$ impurity.

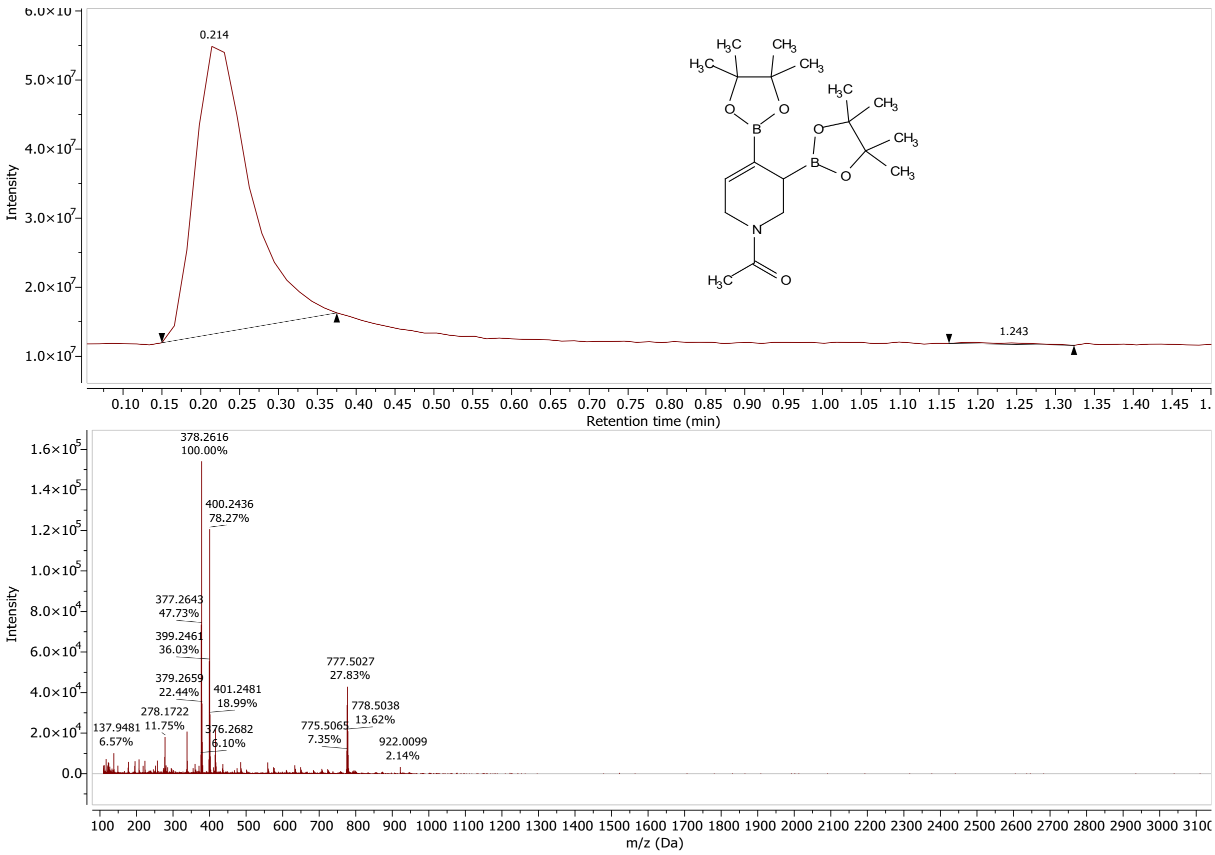

Figure 69: HRMS-ESI spectrum of **4g** measured in positive ion mode.

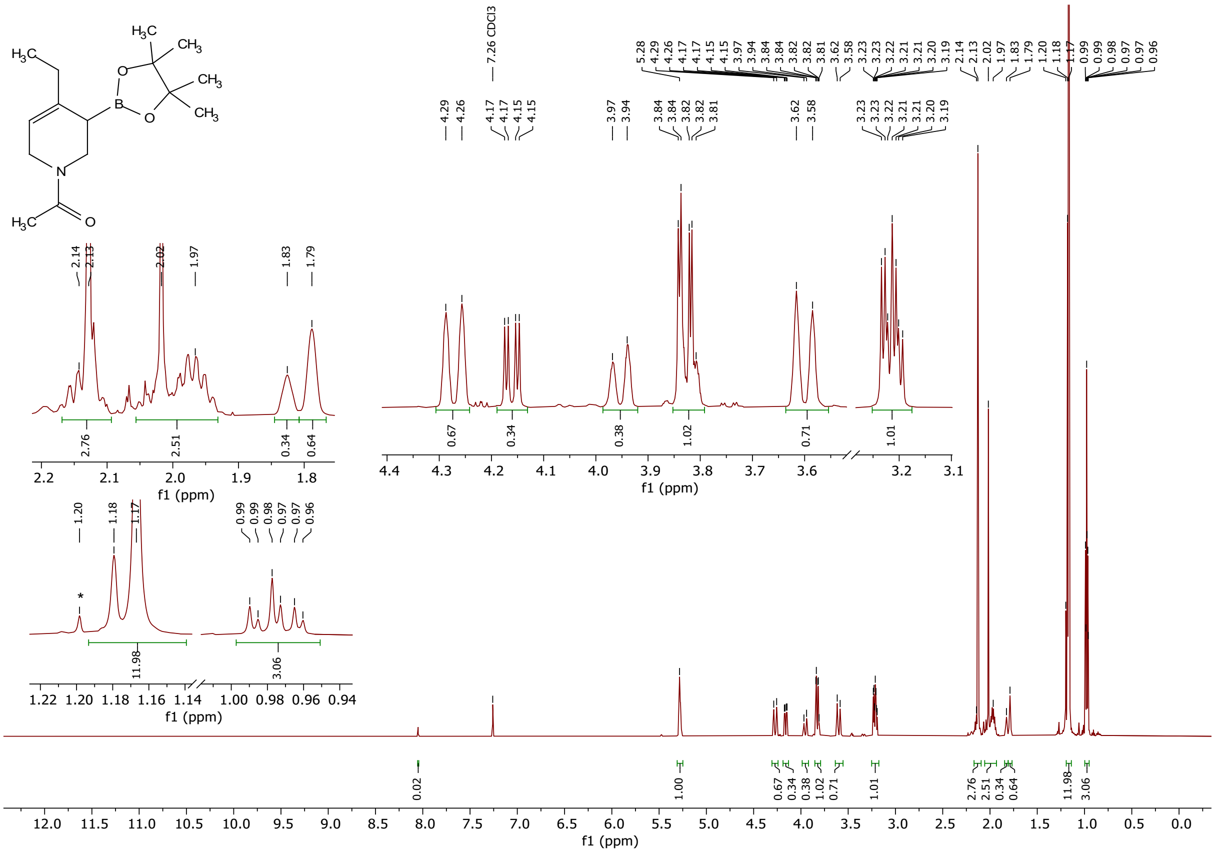

Figure 70: 600 MHz ^{1}H-NMR spectrum of **4d** in $CDCl_3$. The spectrum shows two signal sets due to the presence of rotamers. (*) $B_x pin_y$ impurity.

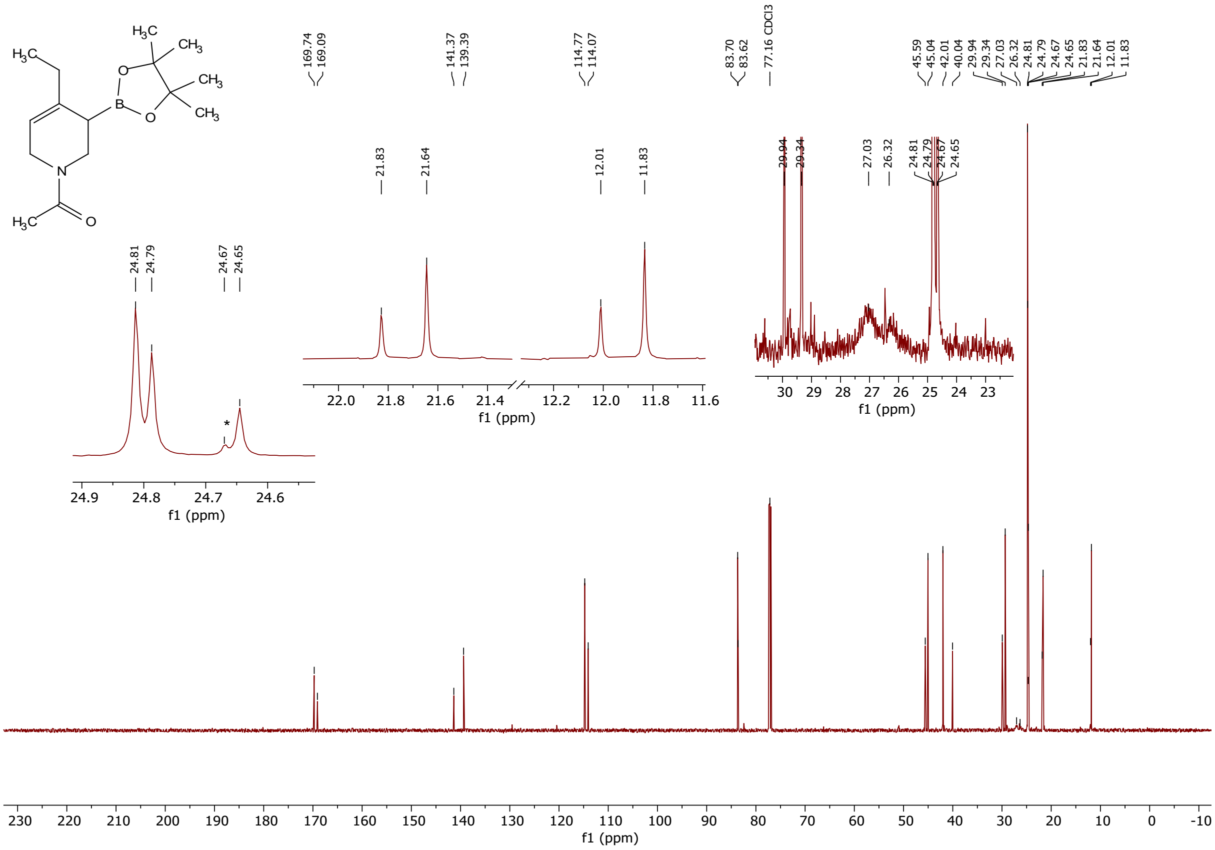

Figure 71: 151 MHz $^{13}C\{^{1}H\}$-NMR spectrum of **4d** in $CDCl_3$. The spectrum shows two signal sets due to the presence of rotamers. (*) $B_x pin_y$ impurity.

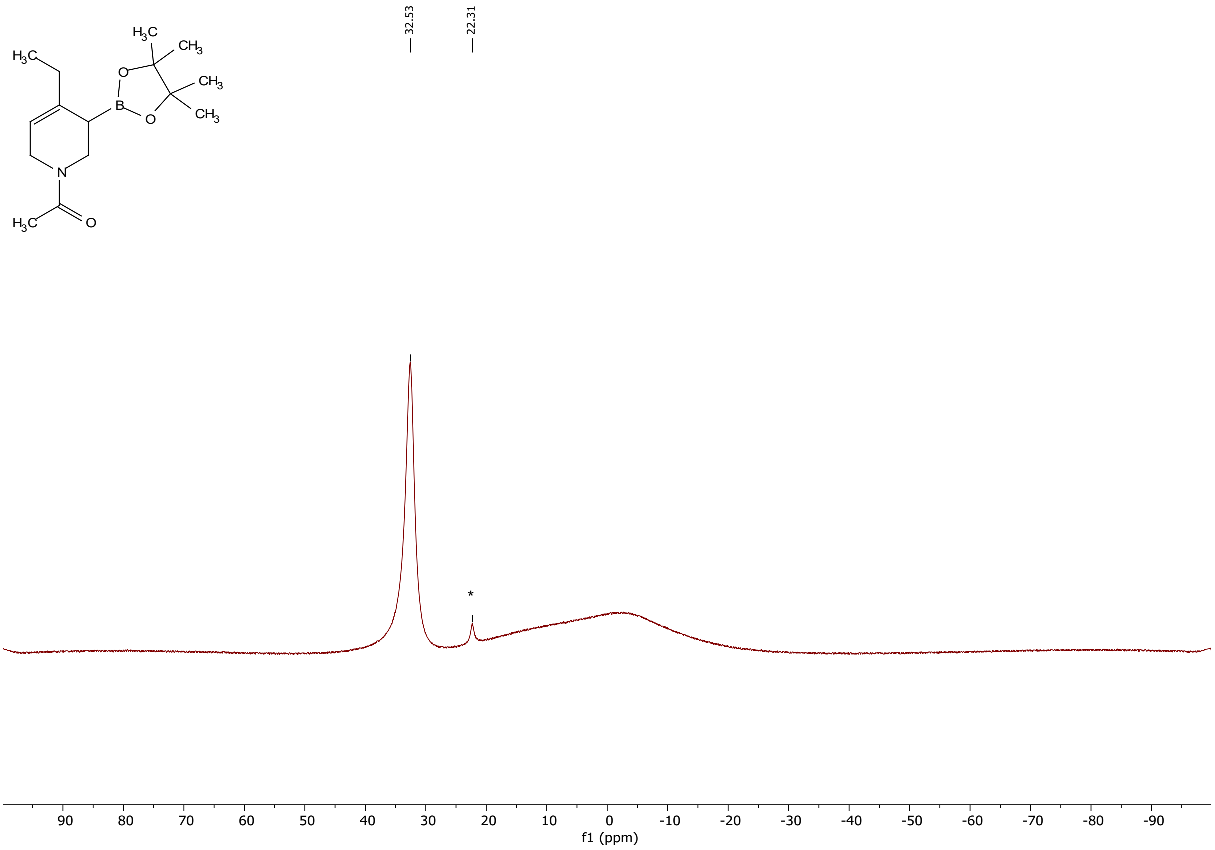

Figure 72: 193 MHz ^{11}B-NMR spectrum of **4d** in $CDCl_3$. (*) $B_x pin_y$ impurity.

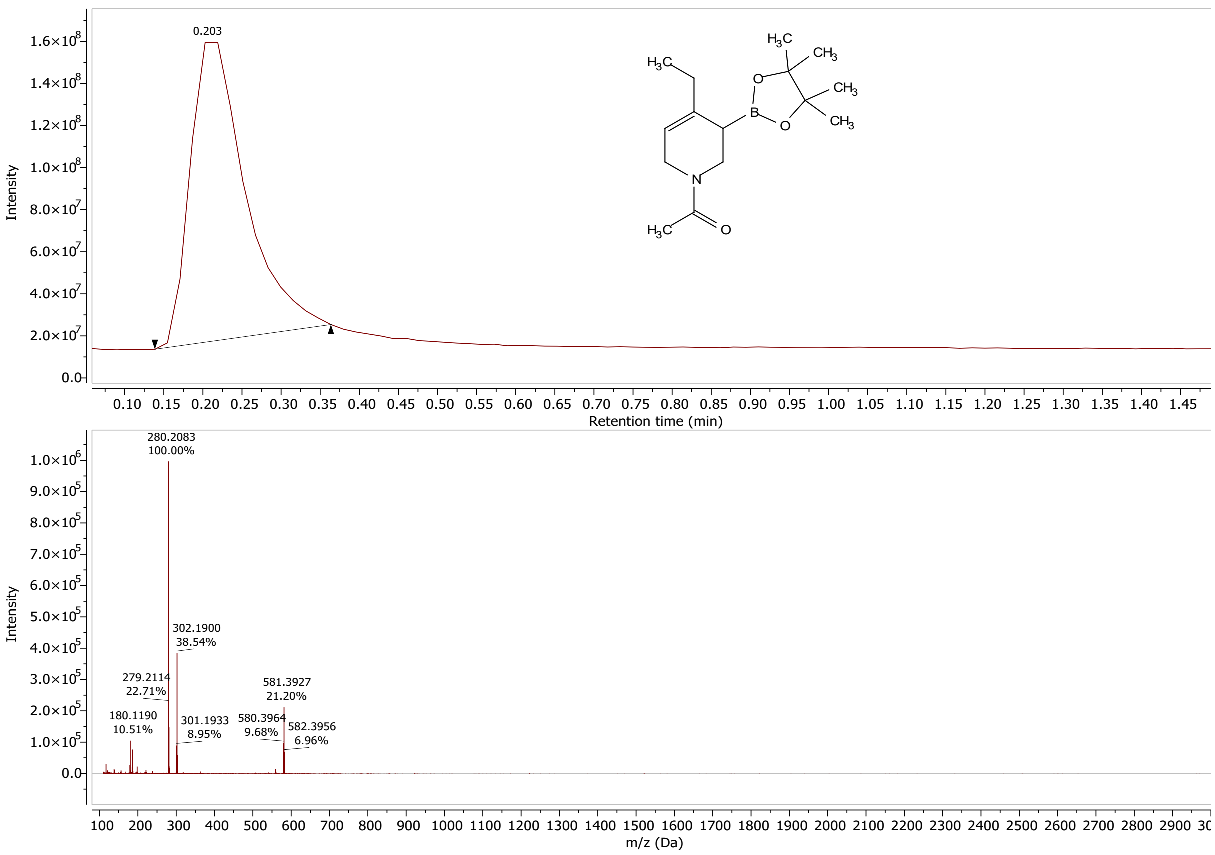

Figure 73: HRMS-ESI spectrum of **4d** measured in positive ion mode.

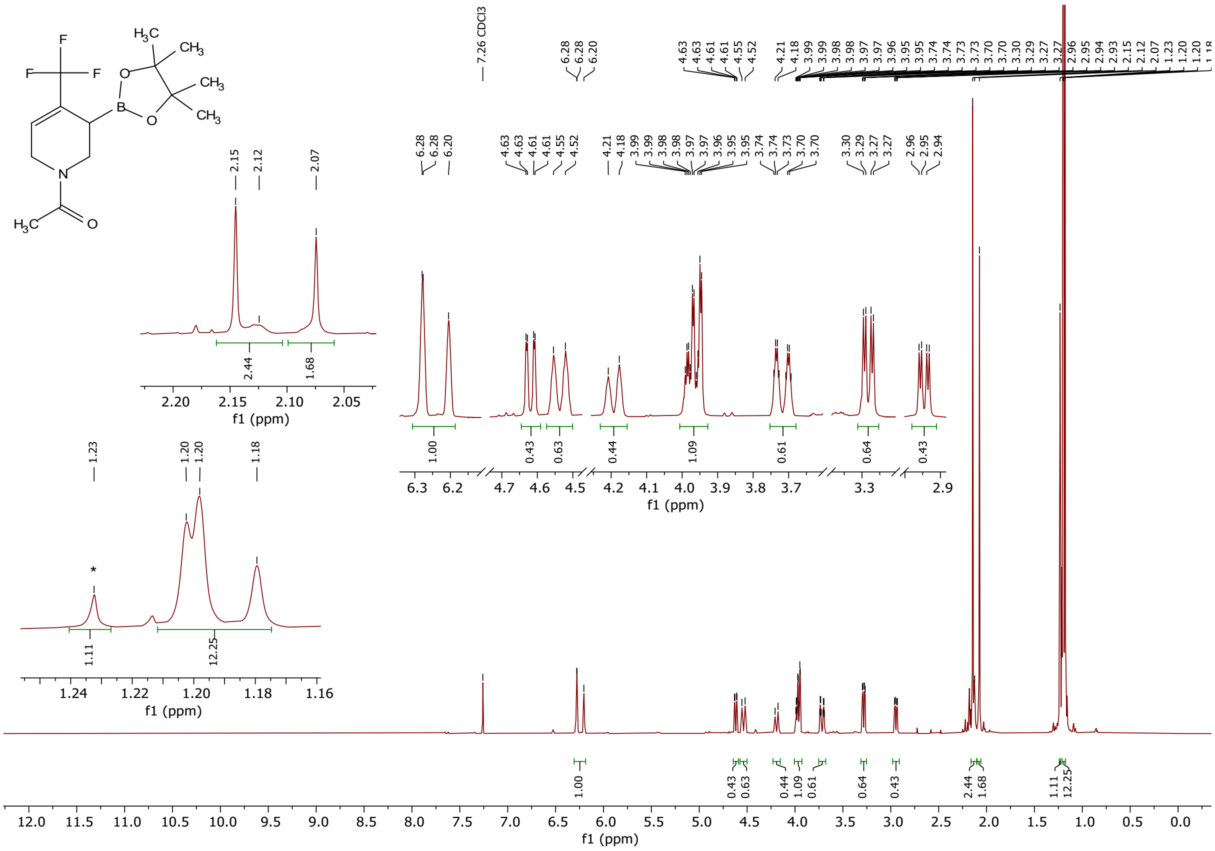

Figure 74: 600 MHz ^{1}H-NMR spectrum of **4f** in $CDCl_3$. The spectrum shows two signal sets due to the presence of rotamers. (*) B_xpin_y impurity.

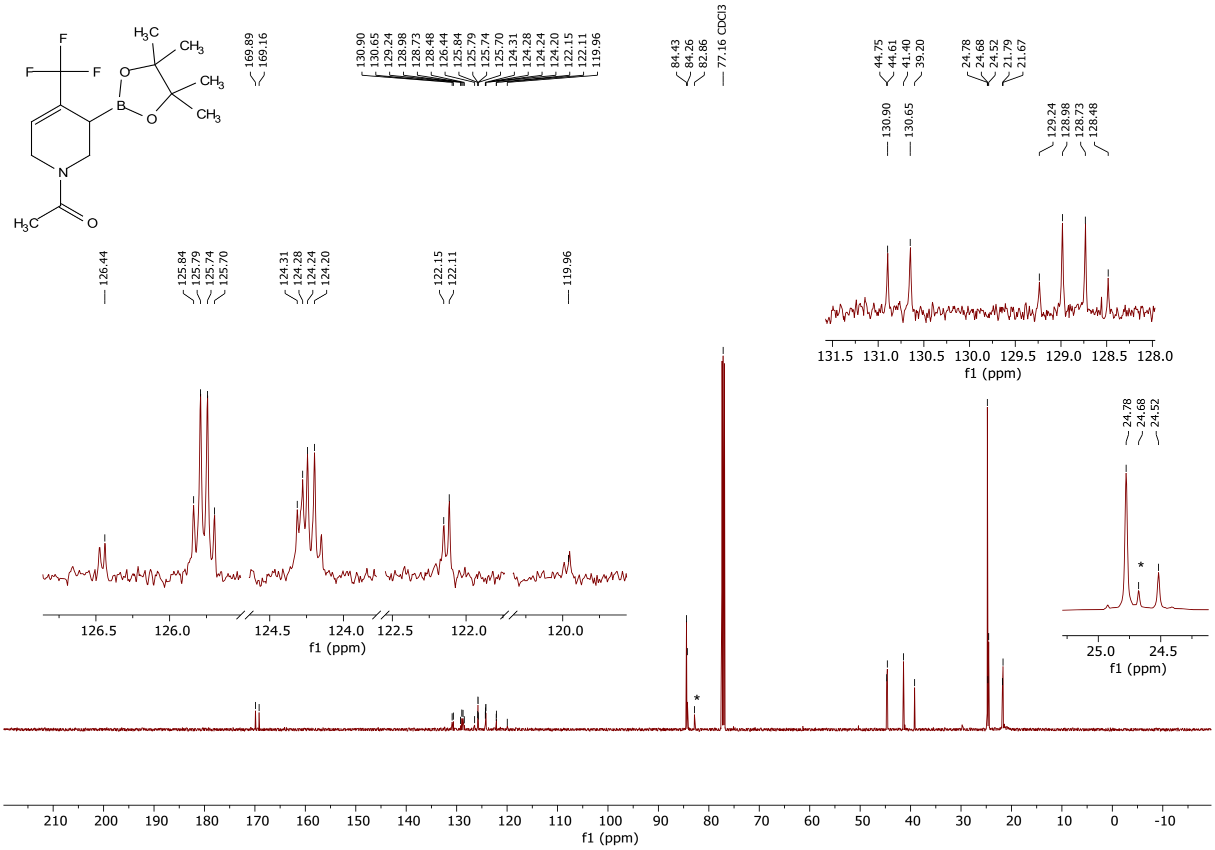

Figure 75: 126 MHz $^{13}C\{^{1}H\}$-NMR spectrum of **4f** in $CDCl_3$. The spectrum shows two signal sets due to the presence of rotamers. (*) B_xpin_y impurity.

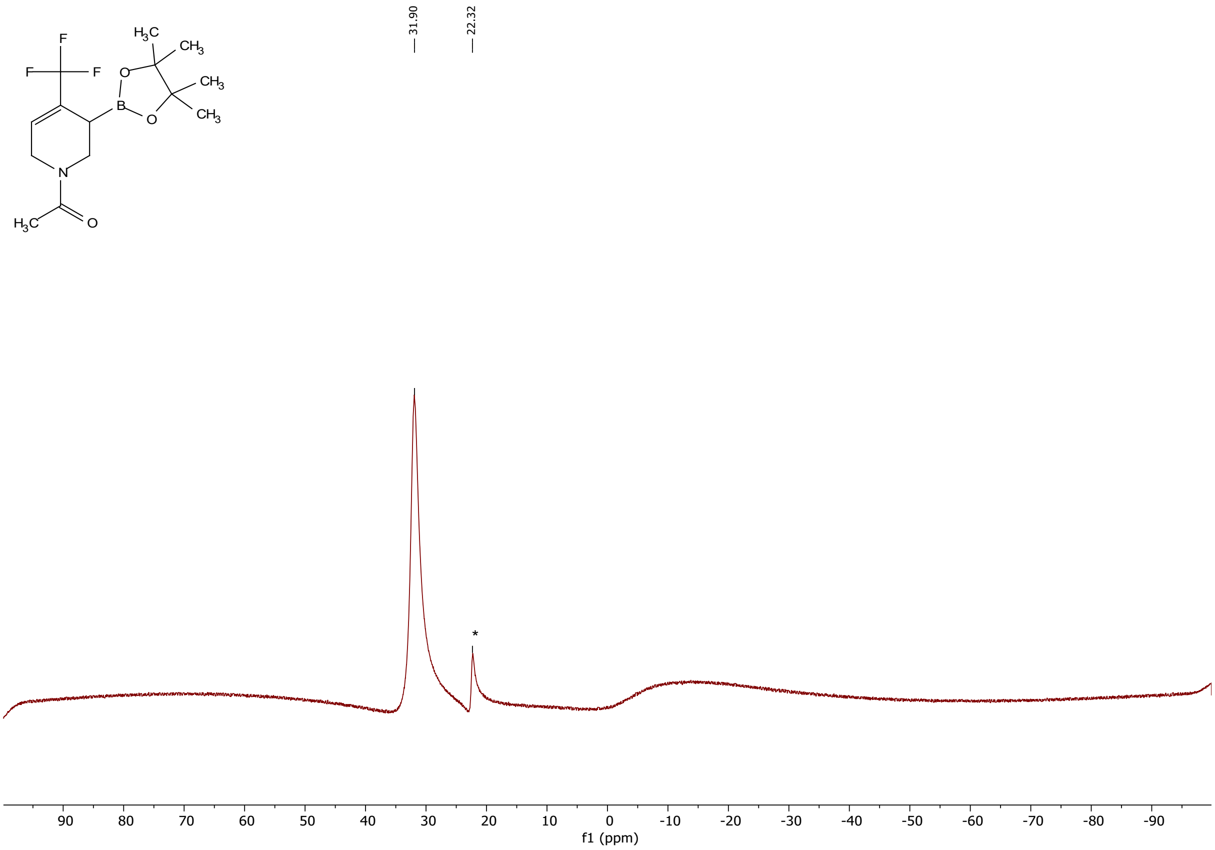

Figure 76: 193 MHz ^{11}B-NMR spectrum of **4f** in $CDCl_3$. (*) B_xpin_y impurity.

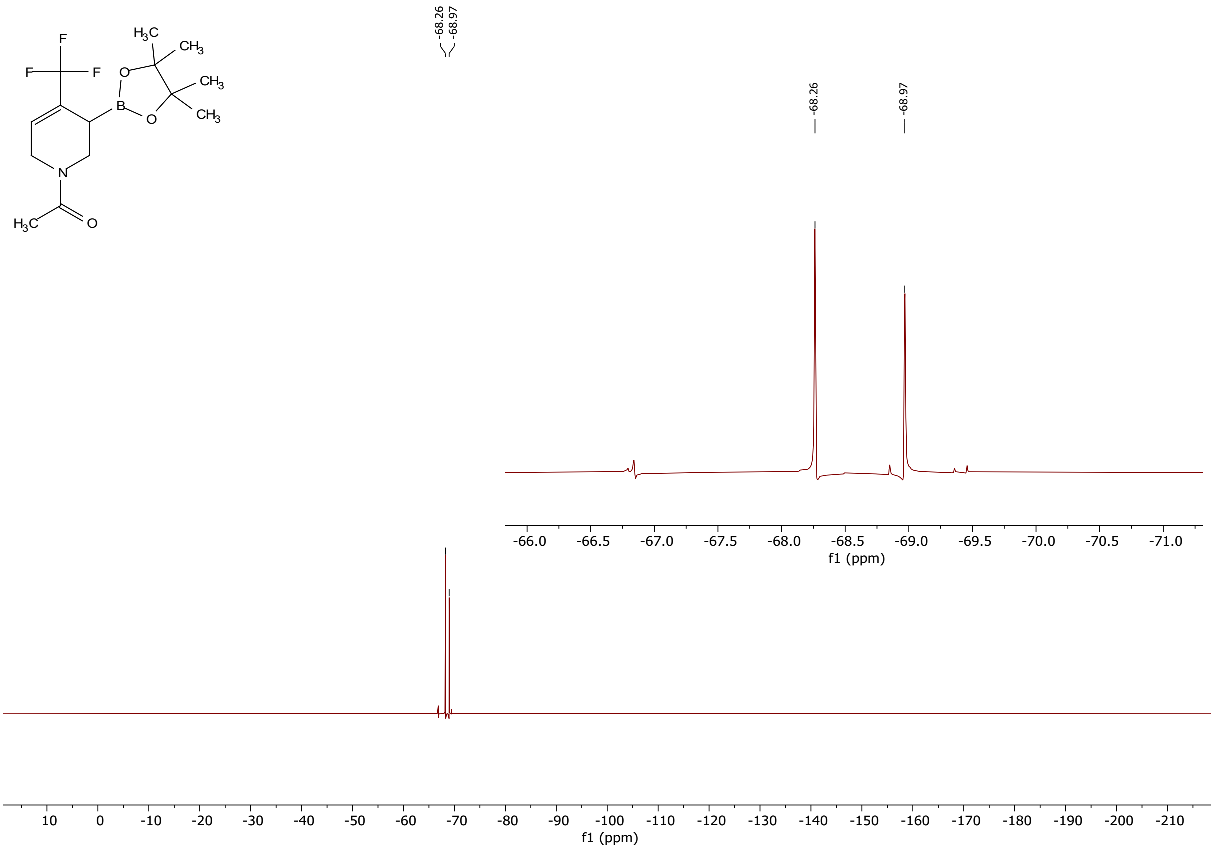

Figure 77: 565 MHz ^{19}F-NMR spectrum of **4f** in $CDCl_3$. The spectrum shows two signal sets due to the presence of rotamers.

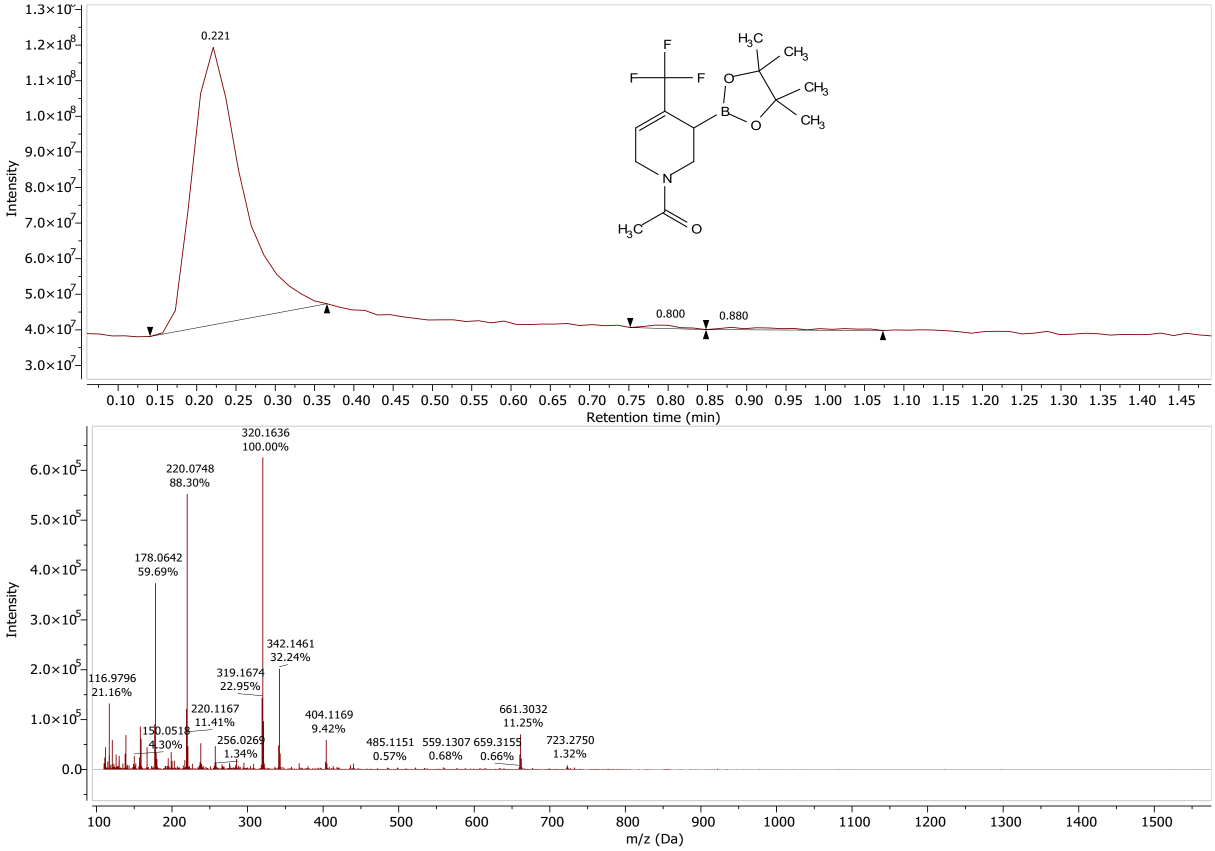

Figure 78: HRMS-ESI spectrum of **4f** measured in positive ion mode.

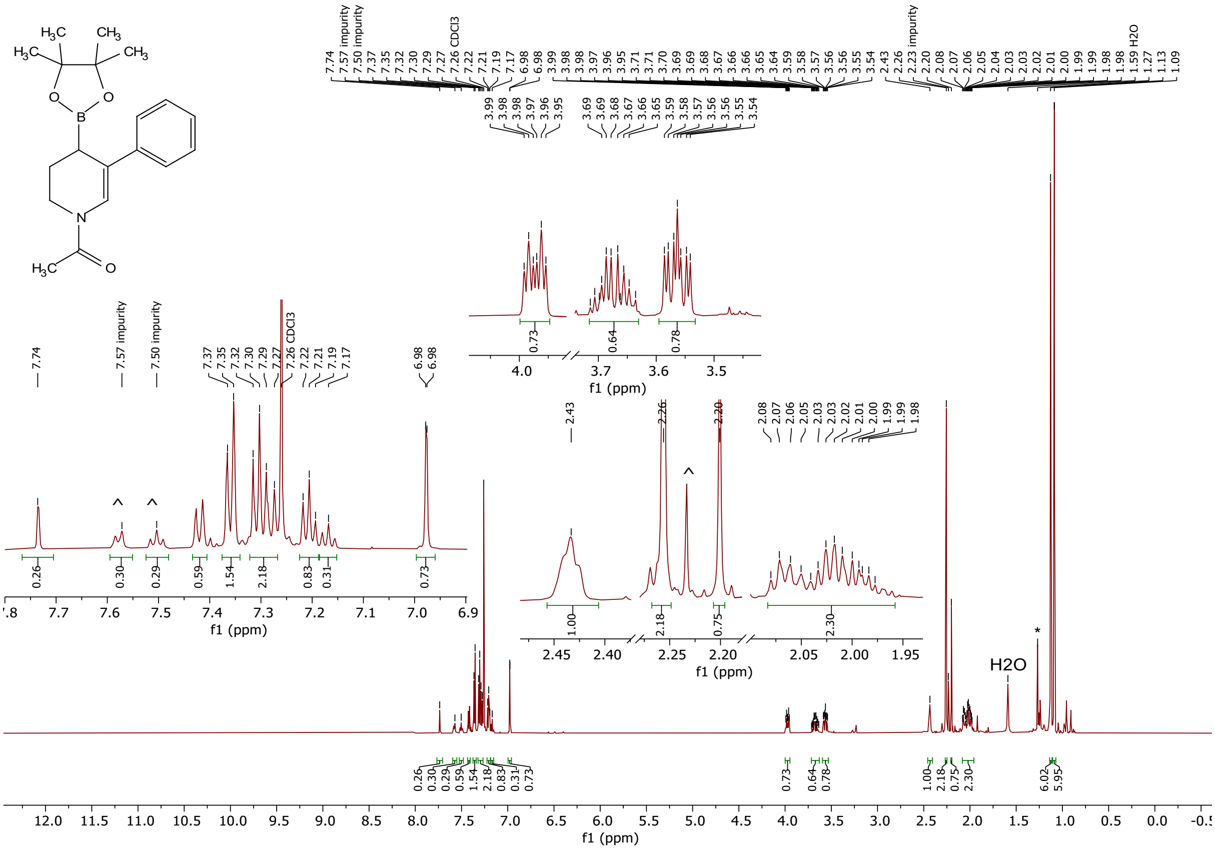

Figure 79: 600 MHz ^{1}H-NMR spectrum of **4i** in $CDCl_3$. The spectrum shows two signal sets due to the presence of rotamers. (^) Unidentified impurity. (*) $B_x pin_y$ impurity.

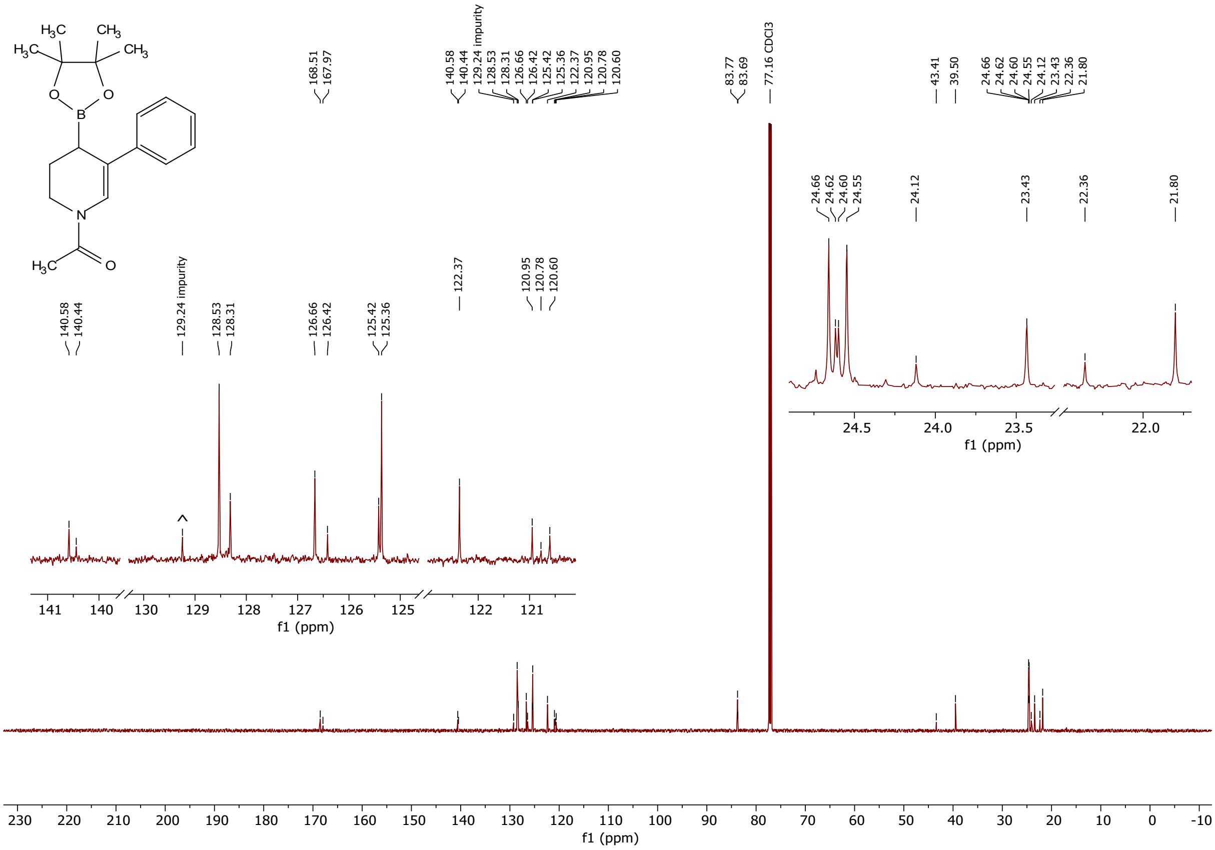

Figure 80: 151 MHz $^{13}C\{^{1}H\}$-NMR spectrum of **4i** in $CDCl_3$. The spectrum shows two signal sets due to the presence of rotamers. (^) Unidentified impurity.

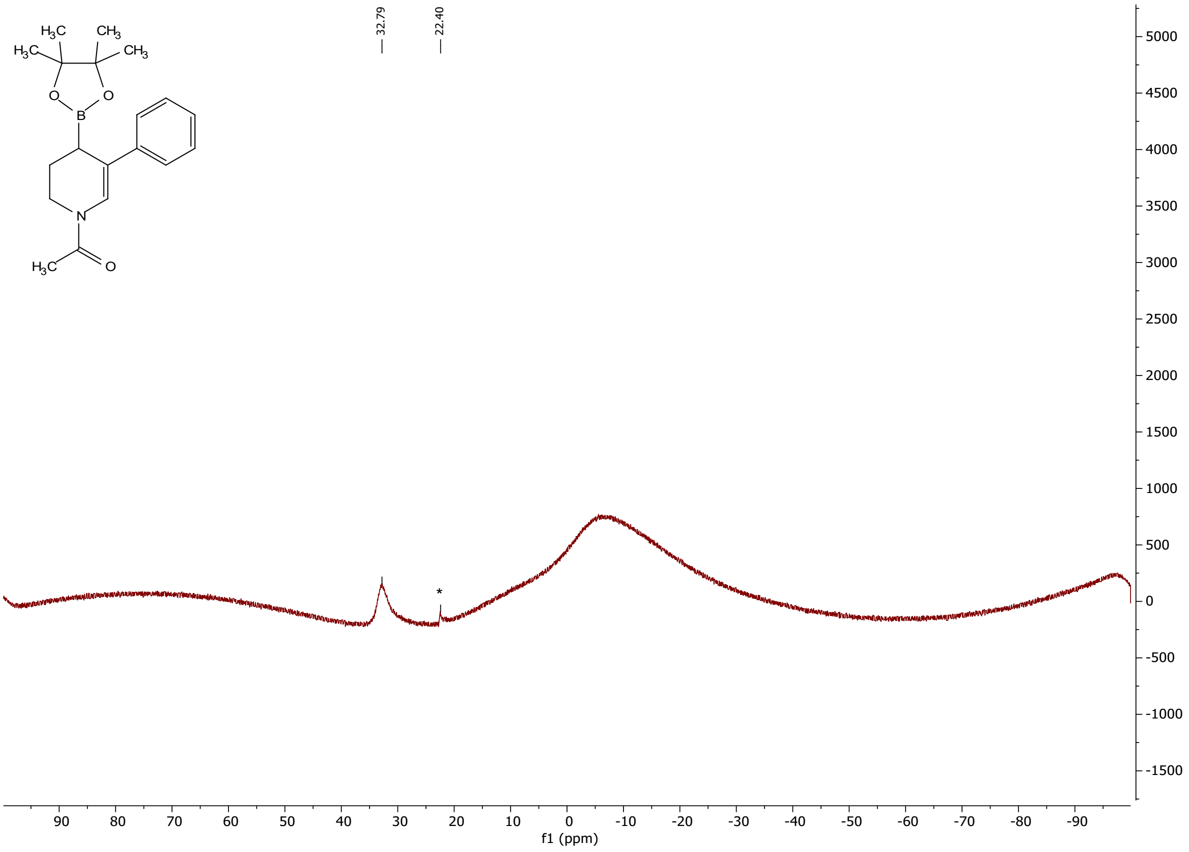

Figure 81: 193 MHz ^{11}B-NMR spectrum of **4i** in $CDCl_3$. (*) B_xpin_y impurity.

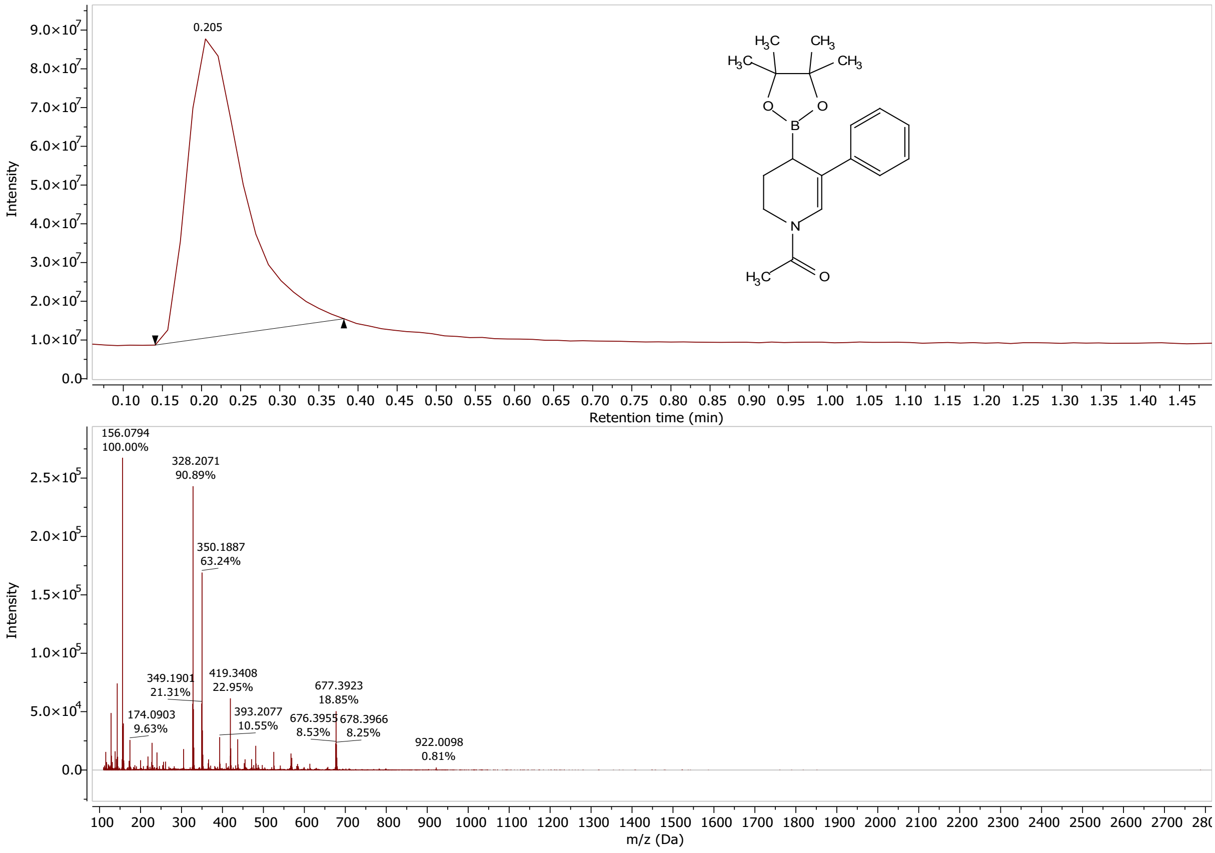

Figure 82: HRMS-ESI spectrum of **4i** measured in positive ion mode.

11.4 Experimental Spectra of Derivatised Tetrahydropyridines

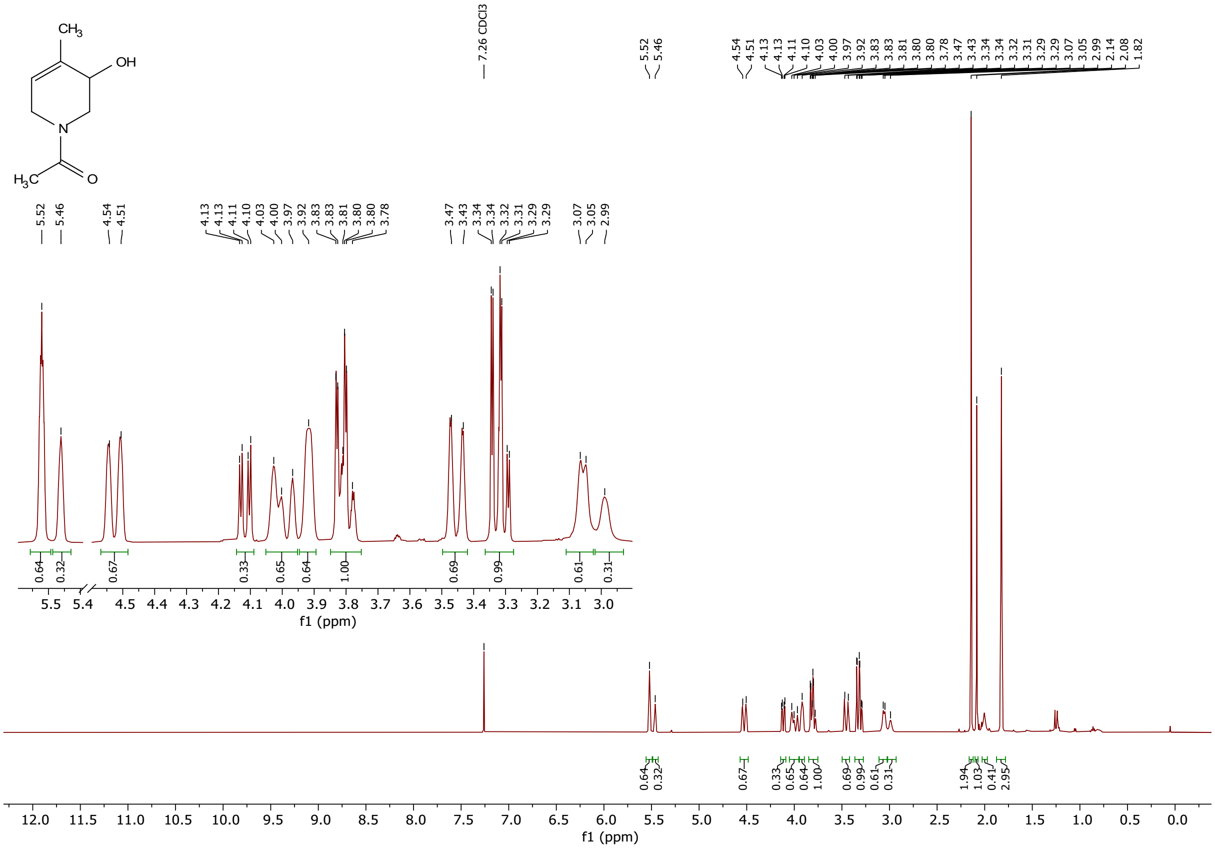

Figure 83: 500 MHz ^{1}H-NMR spectrum of **5** in $CDCl_3$. The spectrum shows two signal sets due to the presence of rotamers.

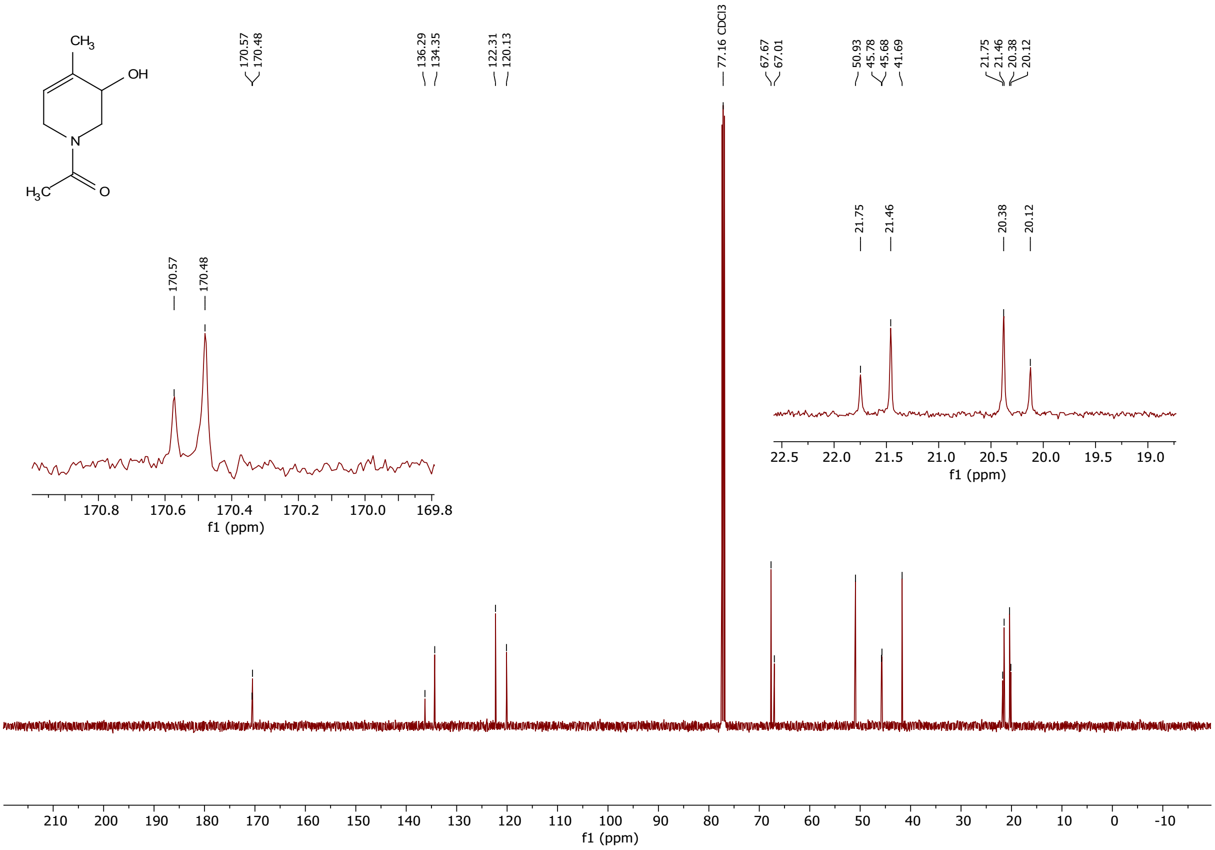

Figure 84: 126 MHz $^{13}C\{^{1}H\}$-NMR spectrum of **5** in $CDCl_3$. The spectrum shows two signal sets due to the presence of rotamers.

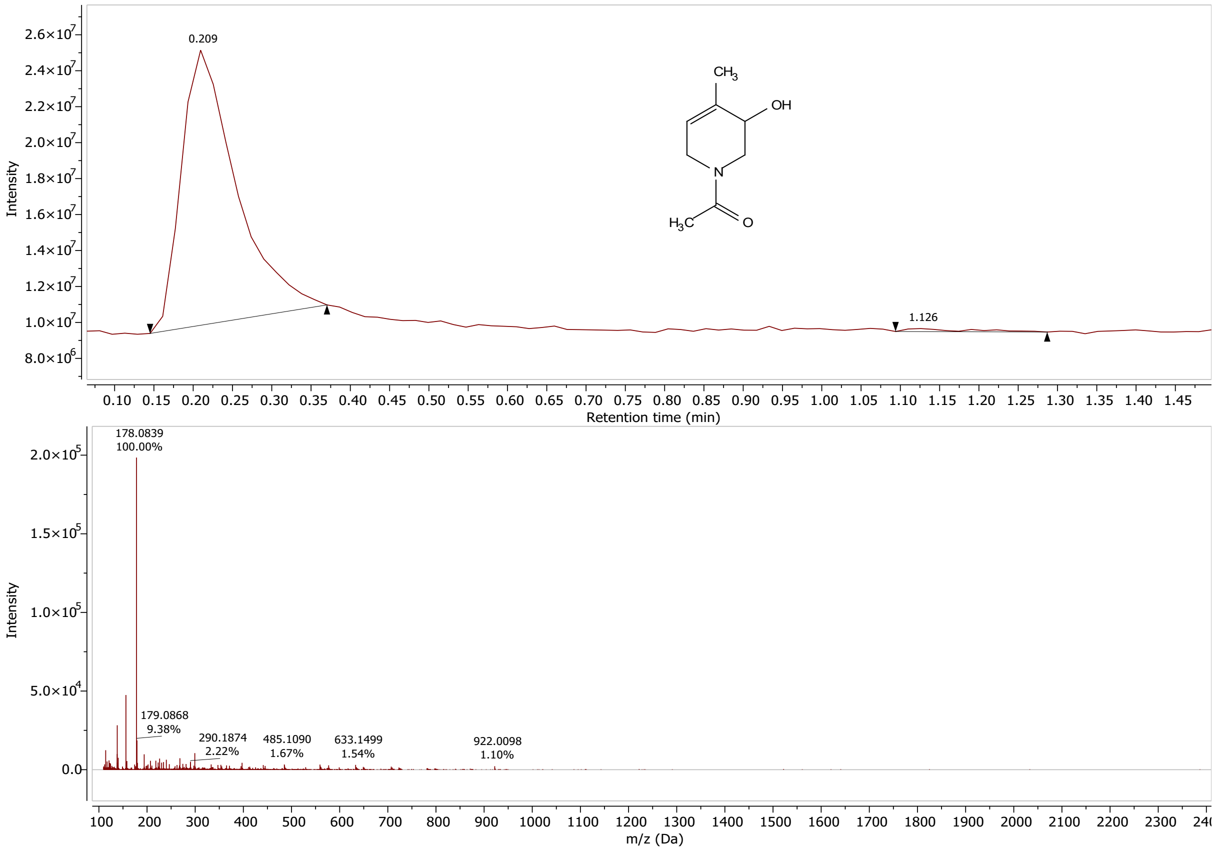

Figure 85: HRMS-ESI spectrum of **5** measured in positive ion mode.

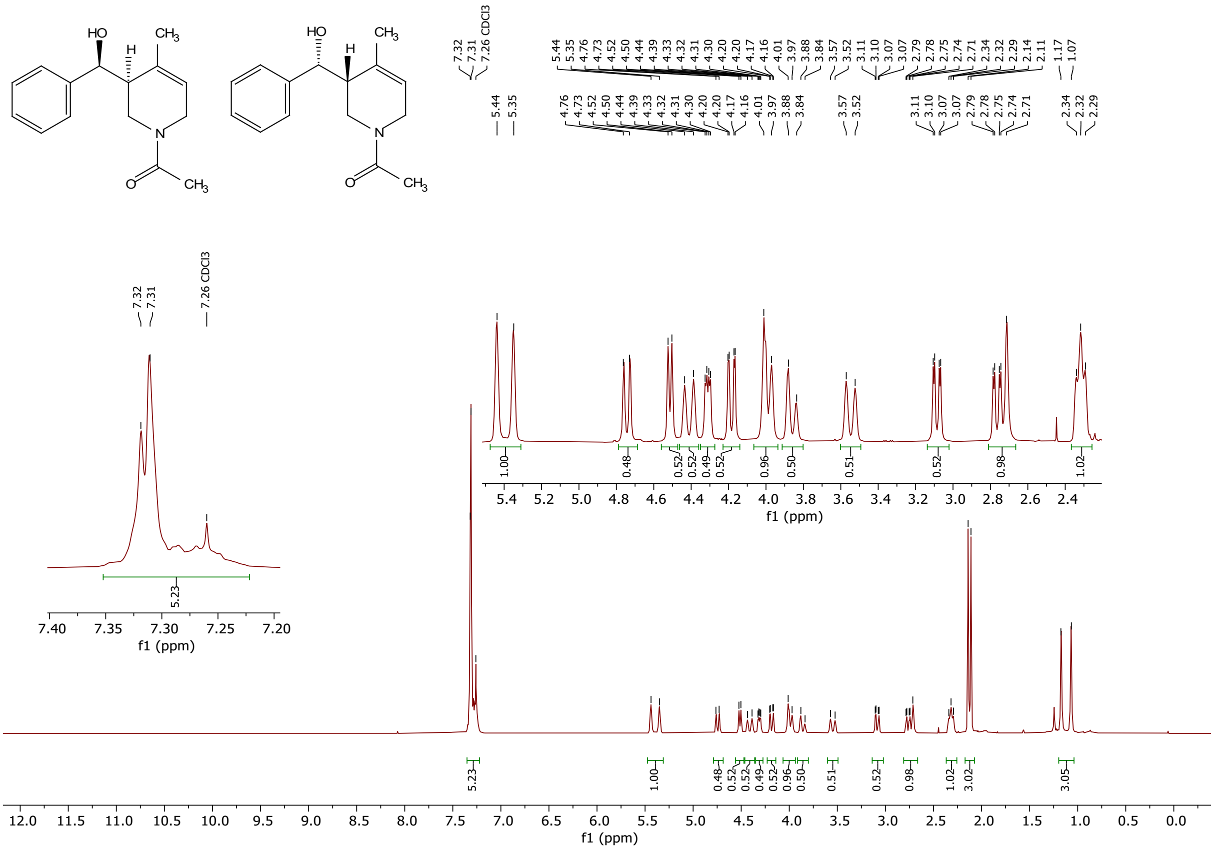

Figure 86: 400 MHz ^{1}H-NMR spectrum of **6** in $CDCl_3$. The spectrum shows two signal sets due to the presence of rotamers.

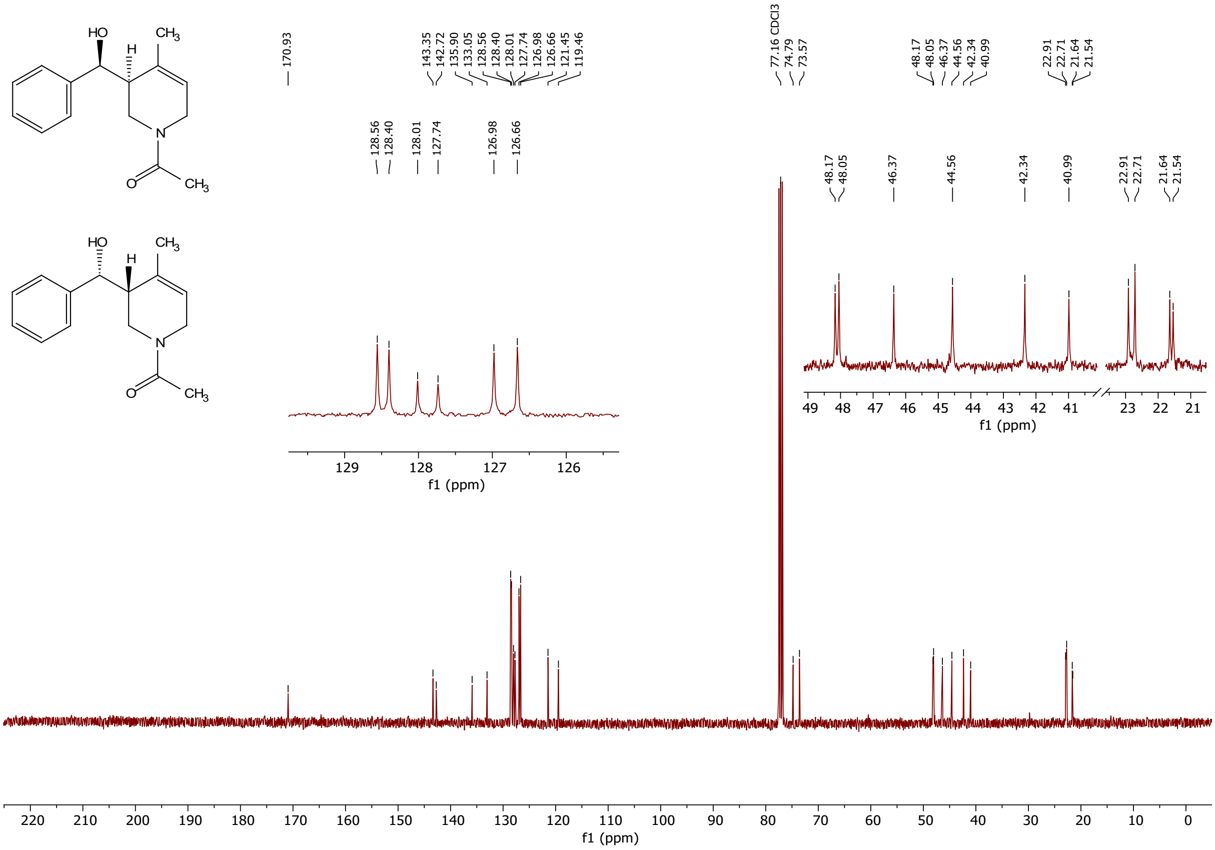

Figure 87: 101 MHz $^{13}C\{^{1}H\}$-NMR of **6** in $CDCl_3$. The spectrum shows two signal sets due to the presence of rotamers.

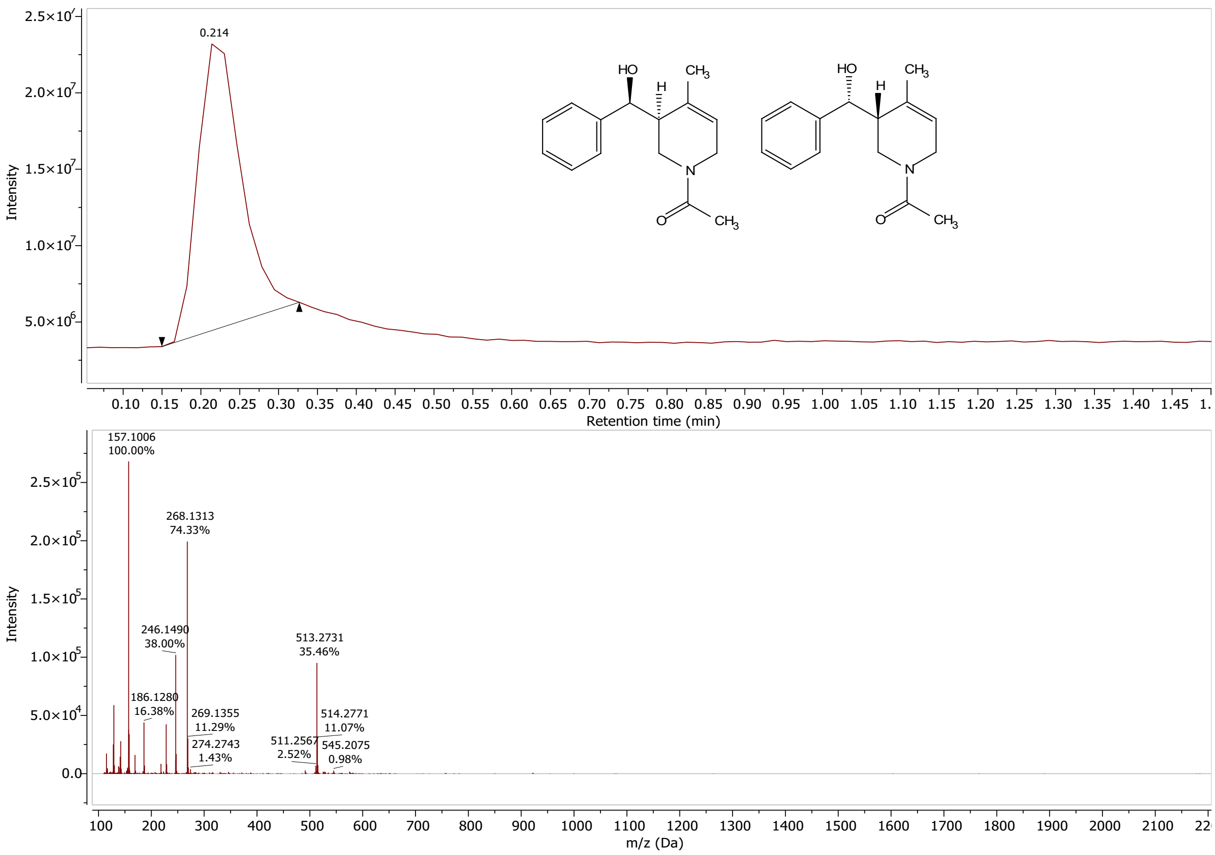

Figure 88: HRMS-ESI spectrum of **6** measured in positive ion mode.

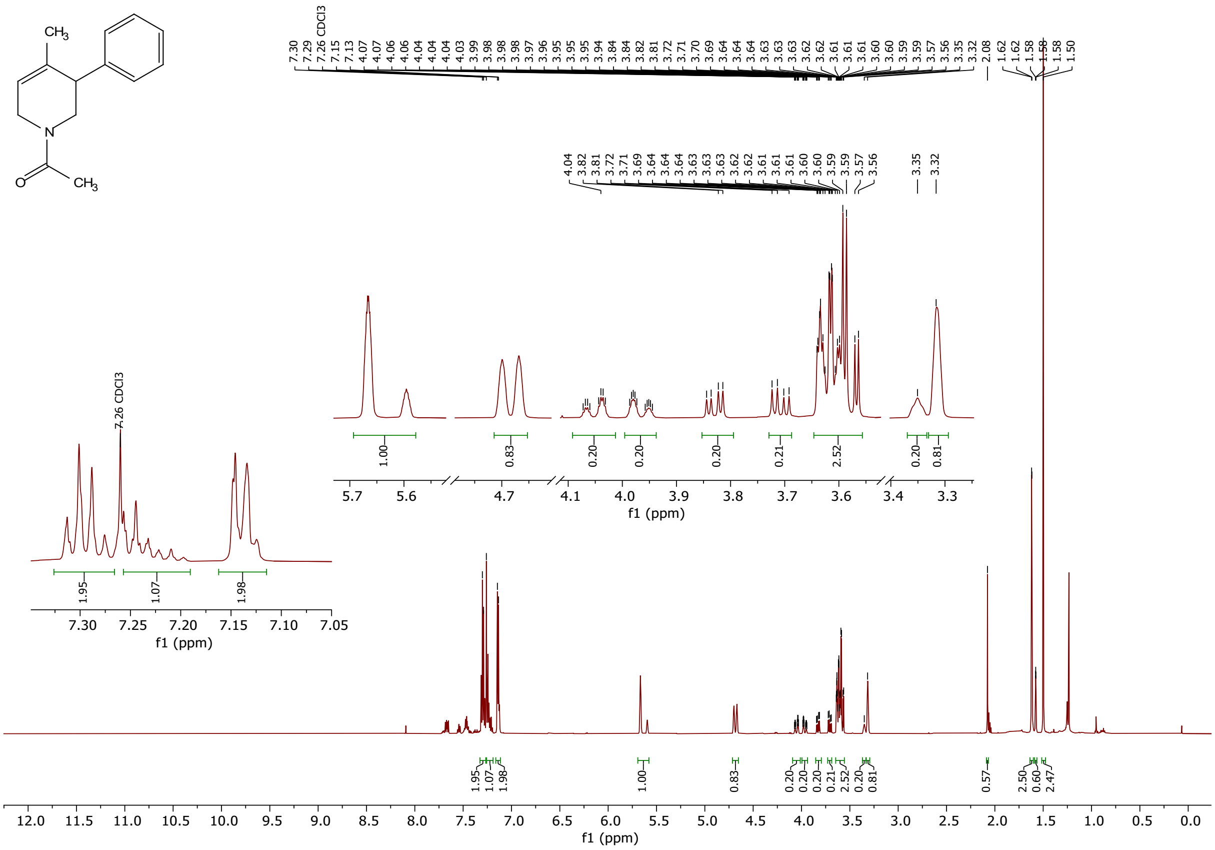

Figure 89: ^{1}H-NMR 600 MHz ^{1}H-NMR spectrum of **7** in $CDCl_3$. The spectrum shows two signal sets due to the presence of rotamers.

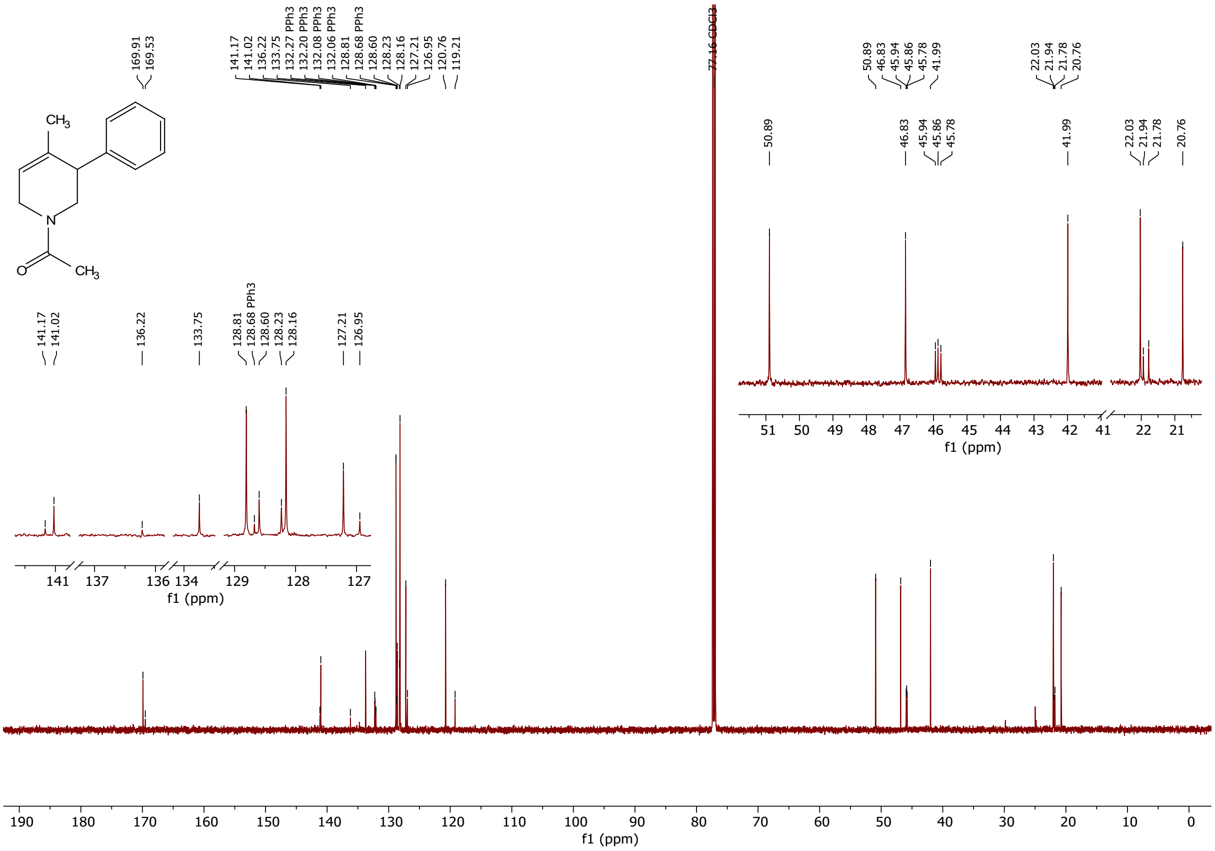

Figure 90: 151 MHz ^{13}C-NMR spectrum of **7** in $CDCl_3$. The spectrum shows two signal sets due to the presence of rotamers.

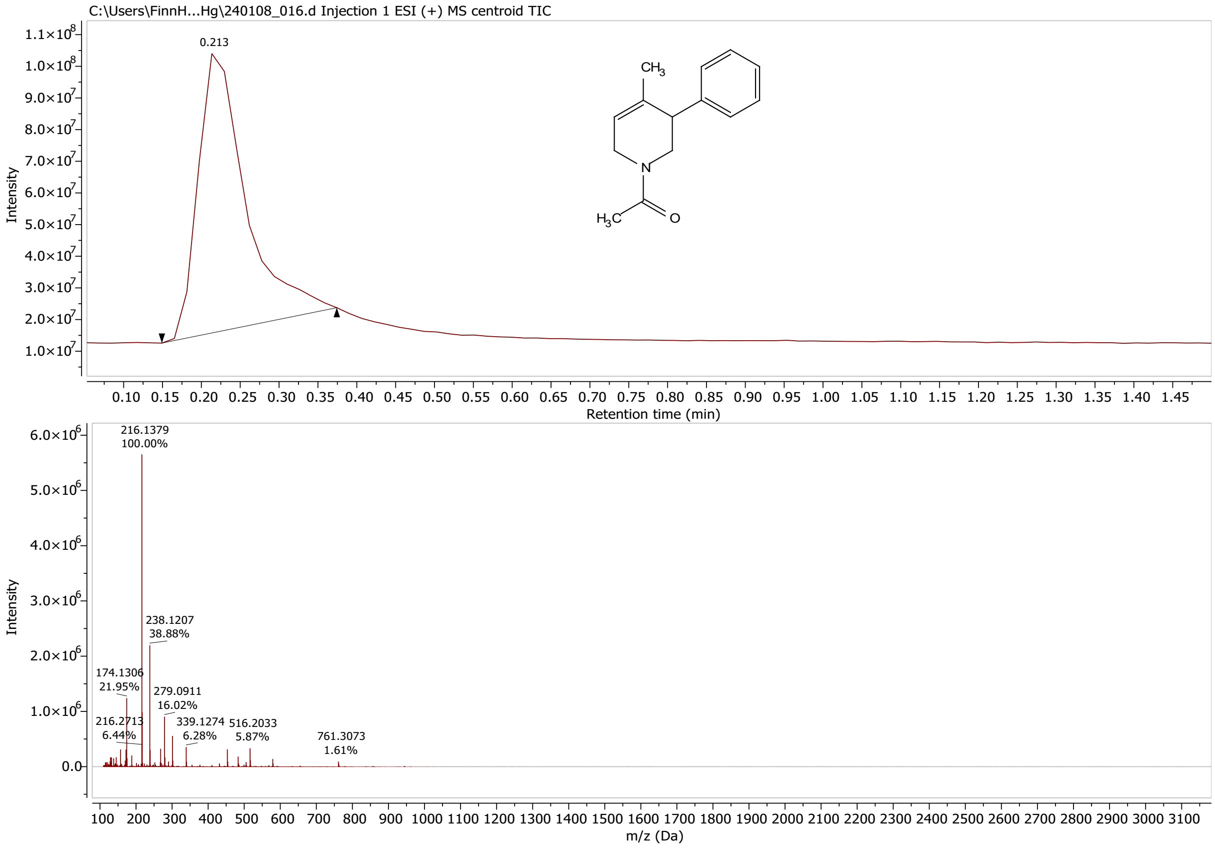

Figure 91: HRMS-ESI spectrum of **7** measured in positive ion mode.

11.5 Other Experimental Spectra

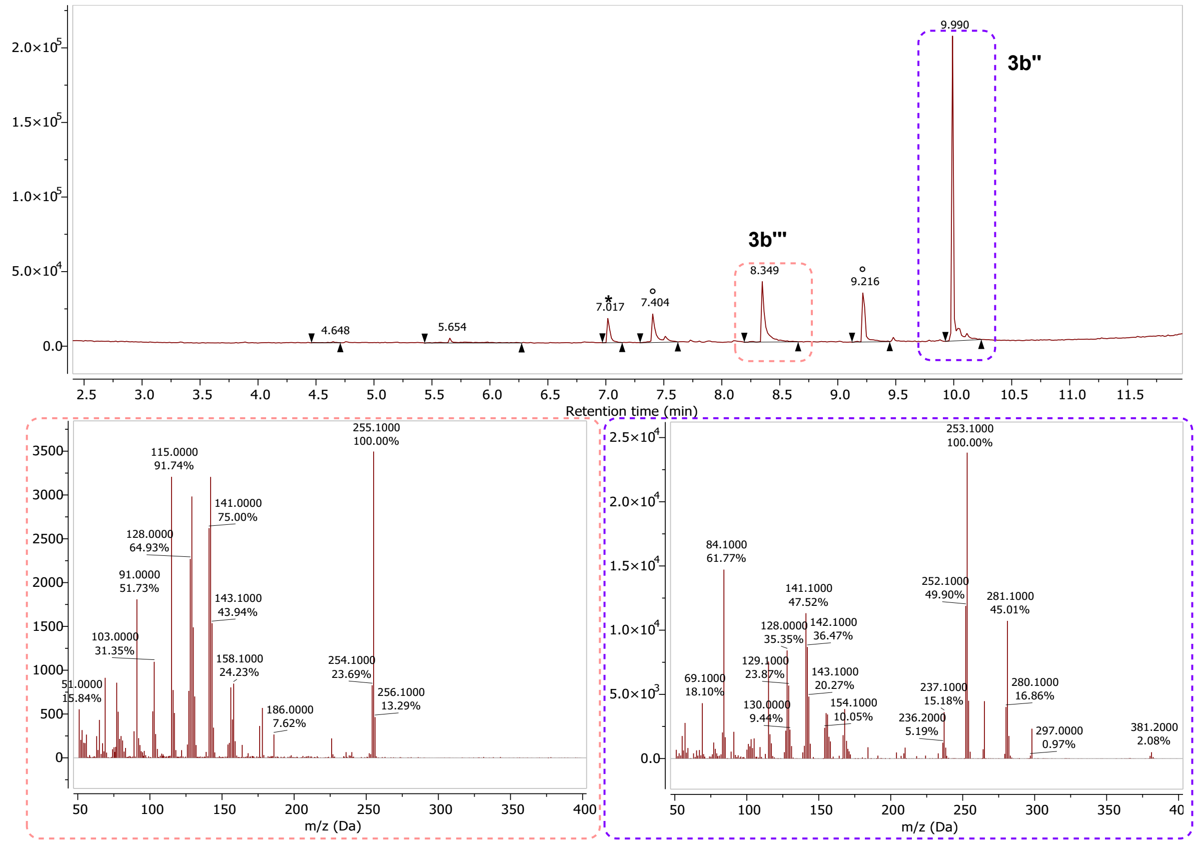

Figure 92: GC-MS spectra obtained after acetylation of the 4-phenylpyridine double hydroboration crude mixture with trifluoroacetic anhydride (1.1 eq.). (°) Contamination from technical grade EtOAc. (*) Internal standard hexamethylbenzene.

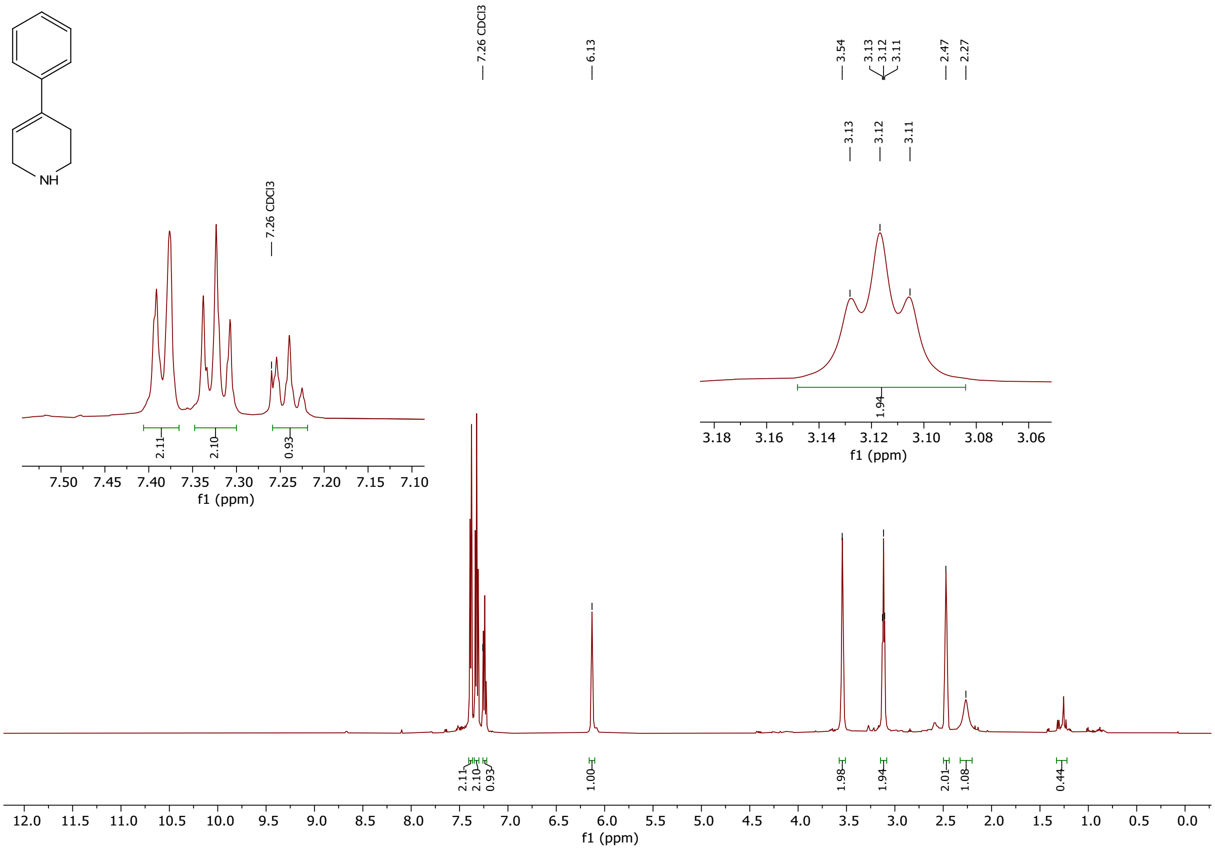

Figure 93: 500 MHz ^{1}H-NMR spectrum of protodeboronation product **7** in $CDCl_3$.

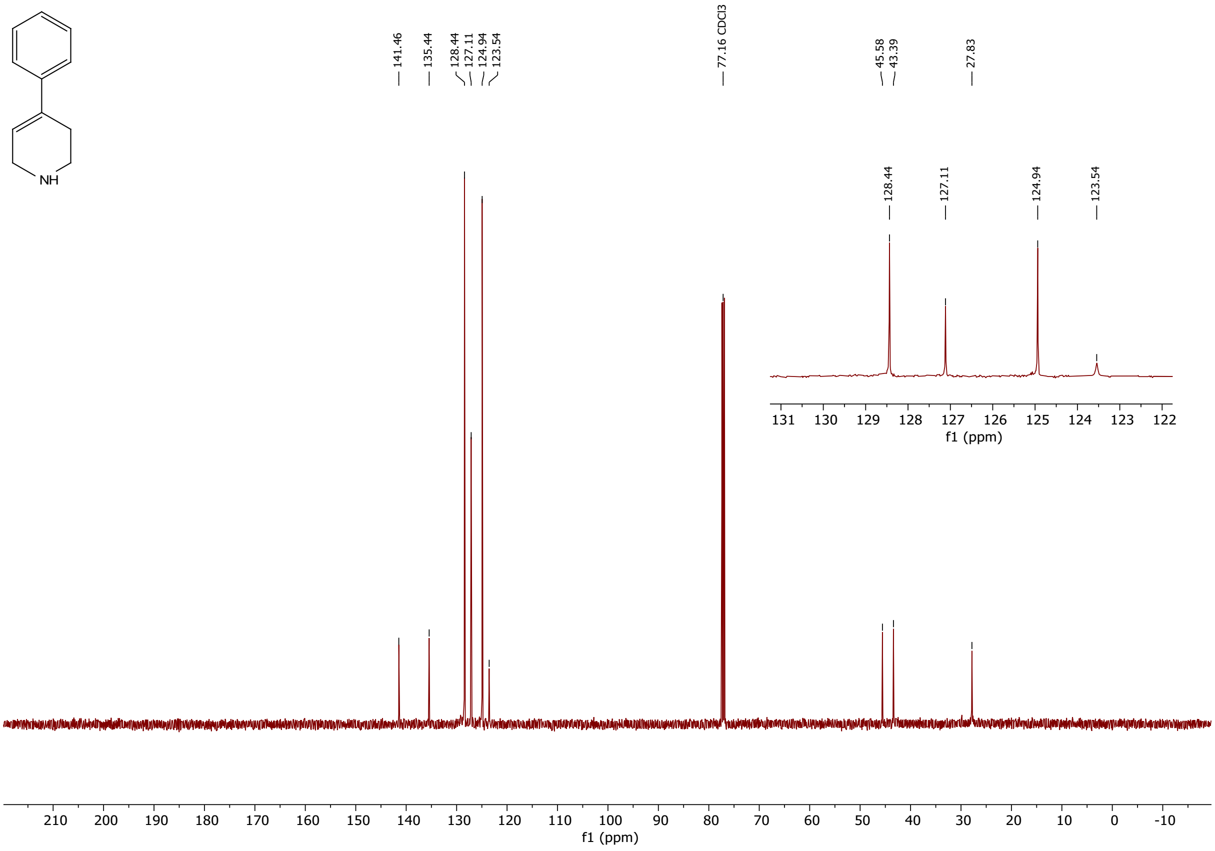

Figure 94: 126 MHz $^{13}C\{^{1}H\}$-NMR spectrum of protodeboronation product **8** in $CDCl_3$.

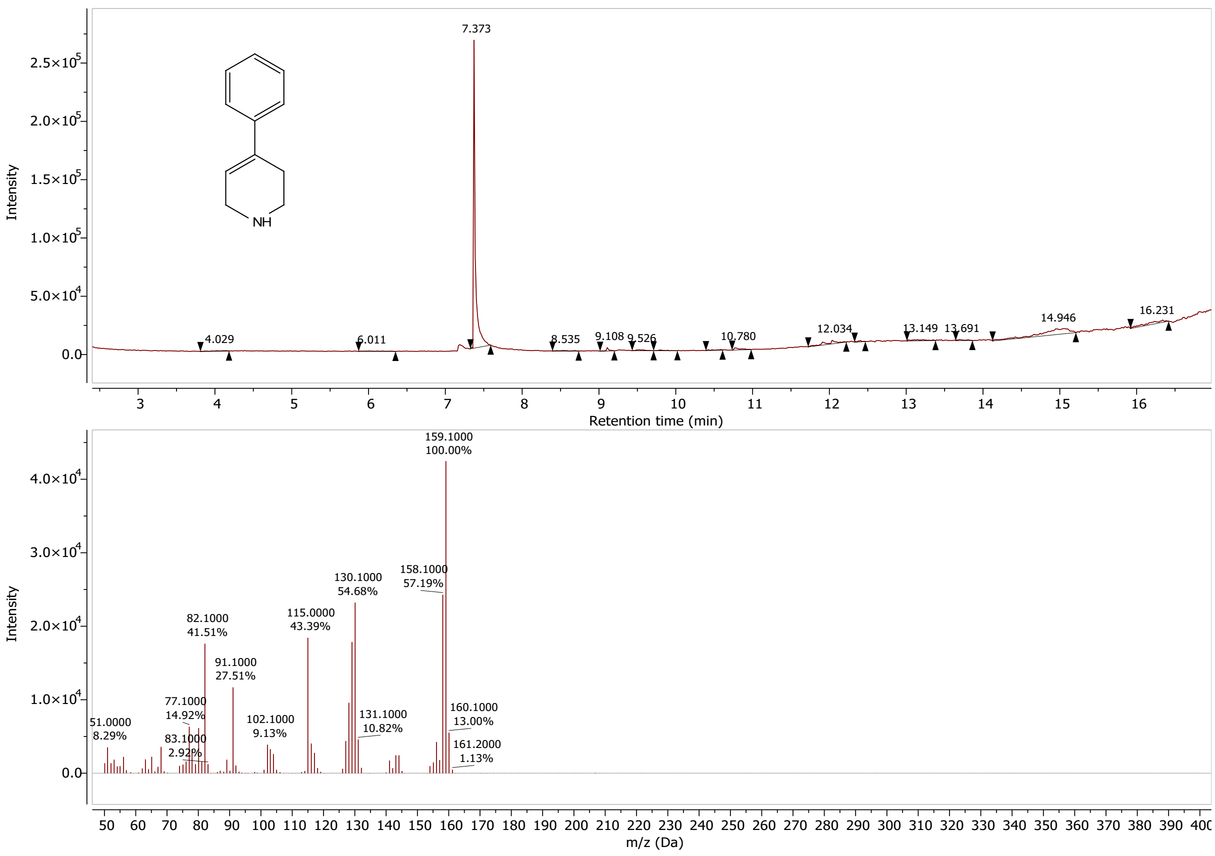

Figure 95: GC-MS spectrum of protodeboronation product **8**.

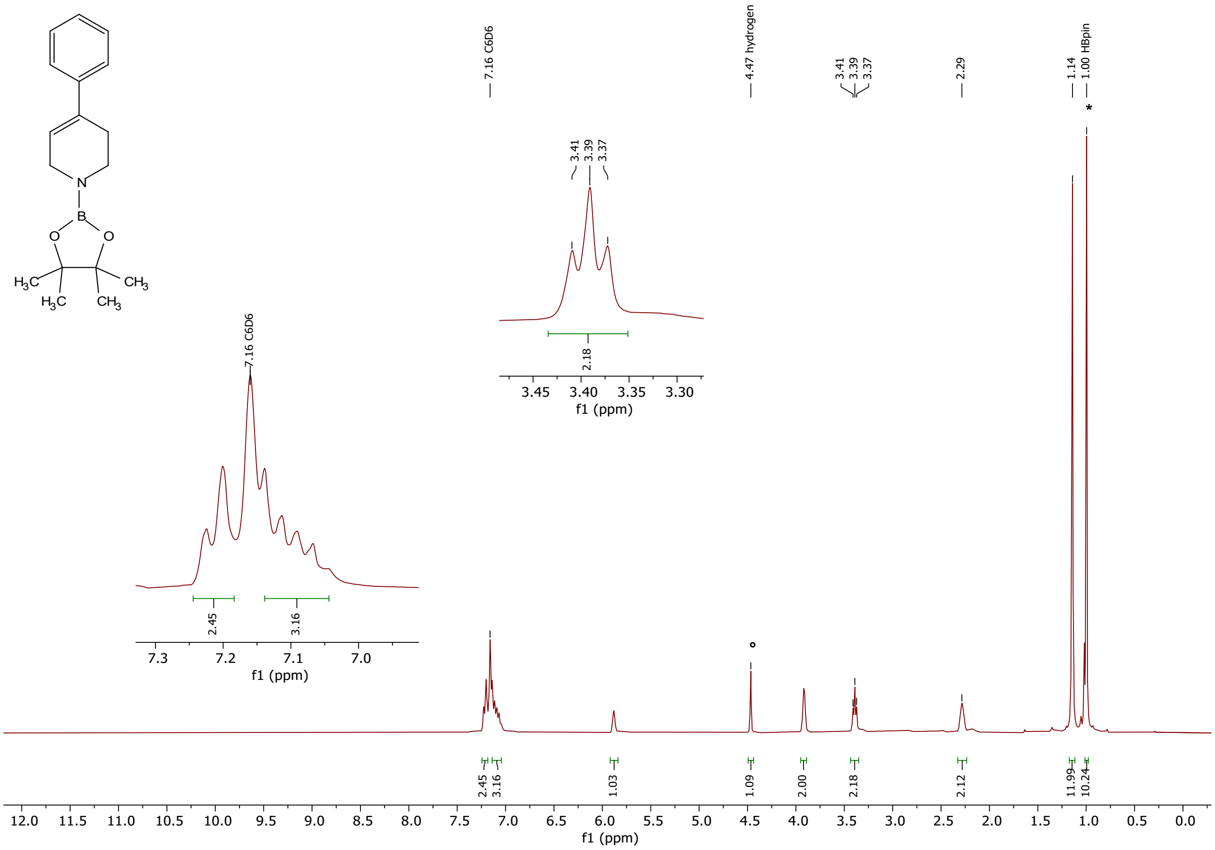

Figure 96: 300 MHz ^{1}H-NMR spectrum of *in situ* generated **3b'** by the reaction of **8** and HBpin in C_6D_6. (*) Signal of HBpin. (°) Signal of hydrogen.

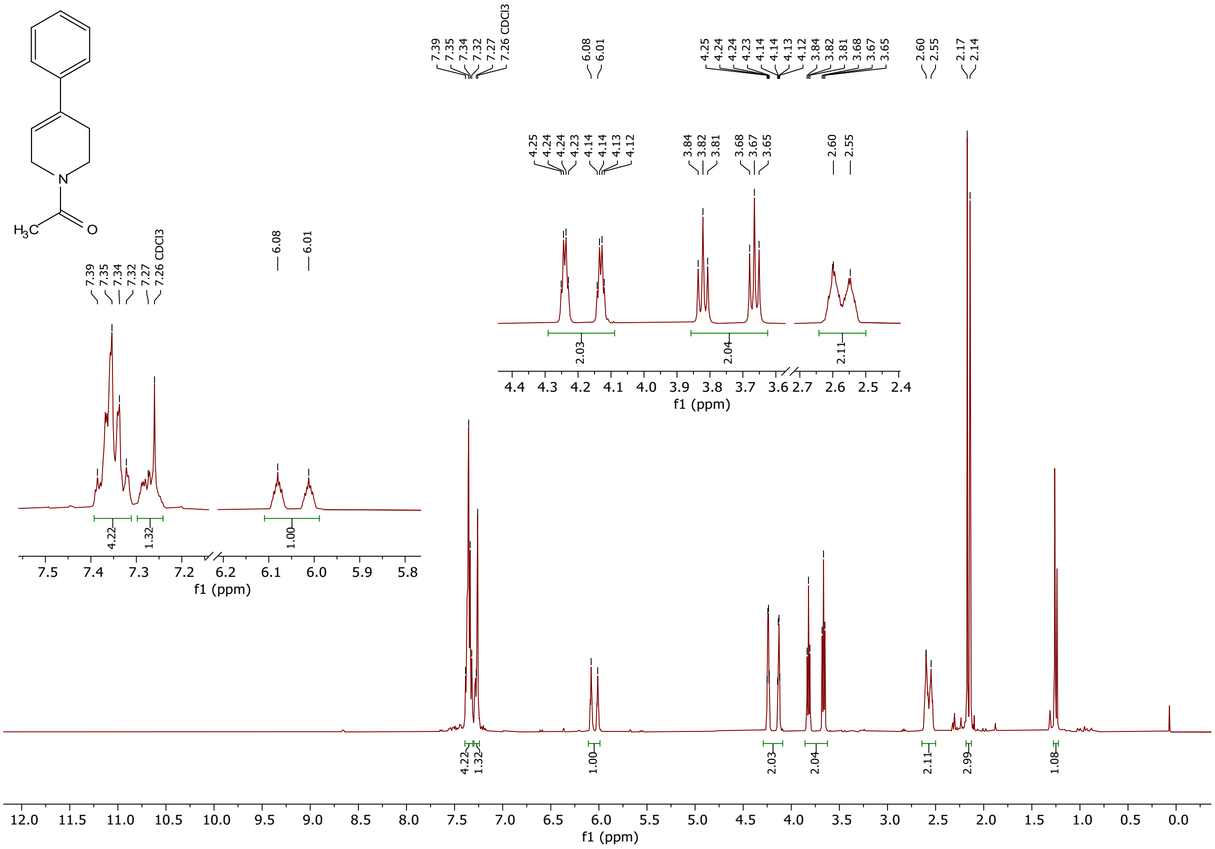

Figure 97: 400 MHz ^{1}H-NMR spectrum of **4b'** in $CDCl_3$ which was isolated during the purification of **4b**. The spectrum shows two signal sets due to the presence of rotamers.

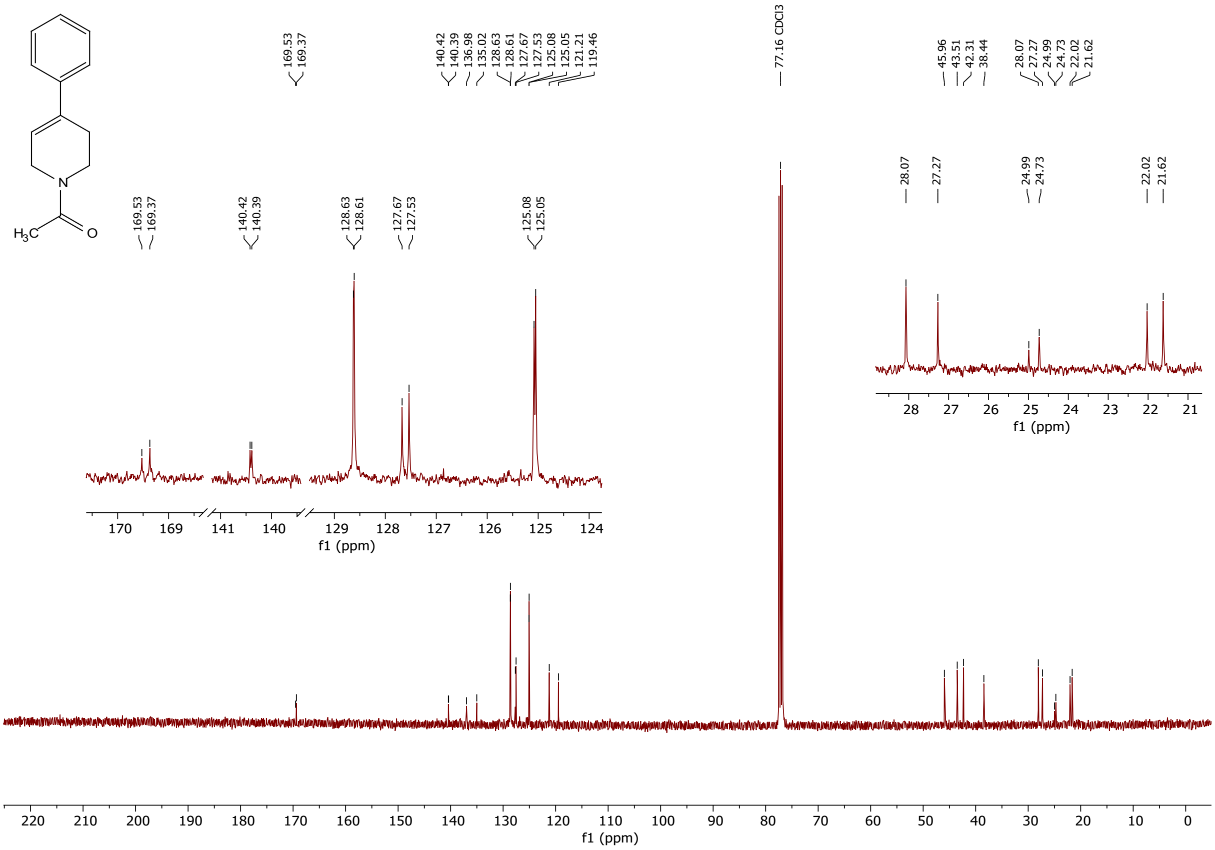

Figure 98: 101 MHz $^{13}C\{^{1}H\}$-NMR spectrum of **4b'** in $CDCl_3$ which was isolated during the purification of **4b**. The spectrum shows two signal sets due to the presence of rotamers.

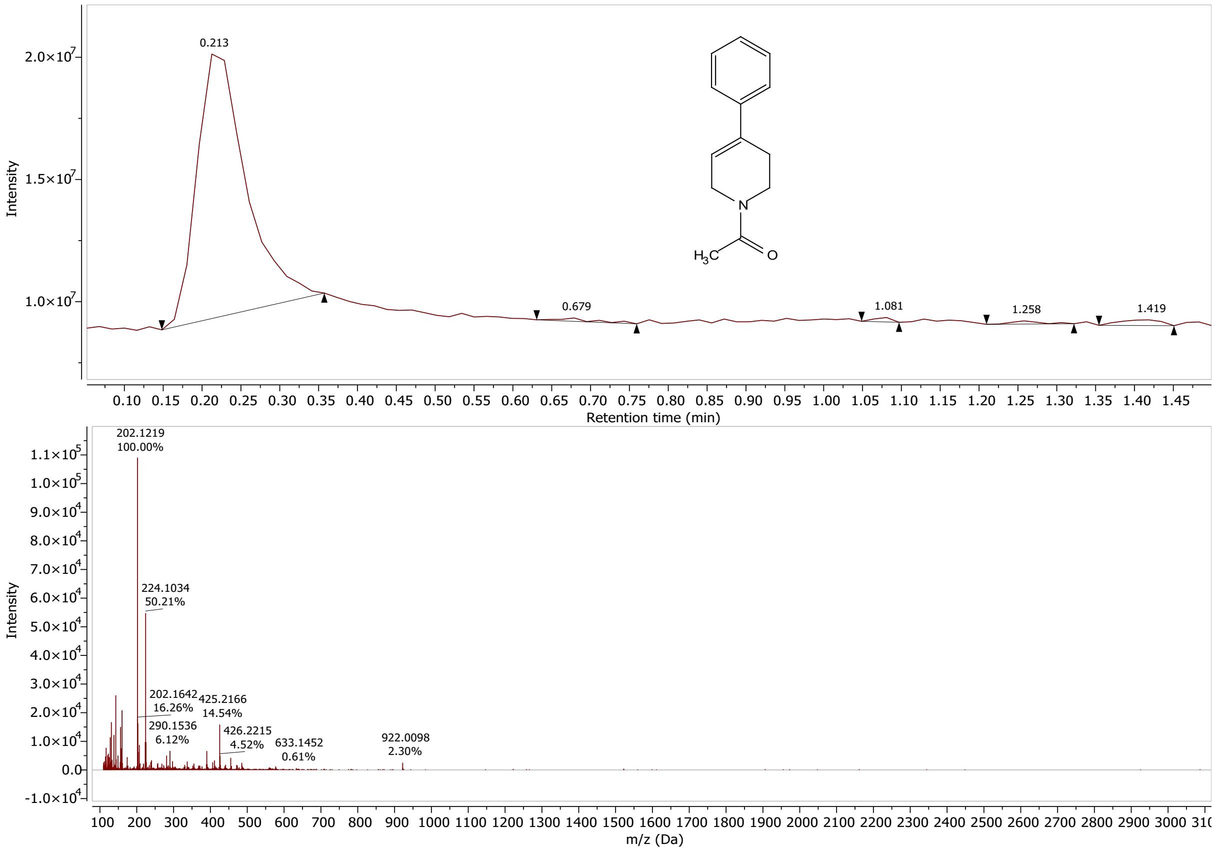

Figure 99: HRMS-ESI spectrum of **4b'** measured in positive ion mode.

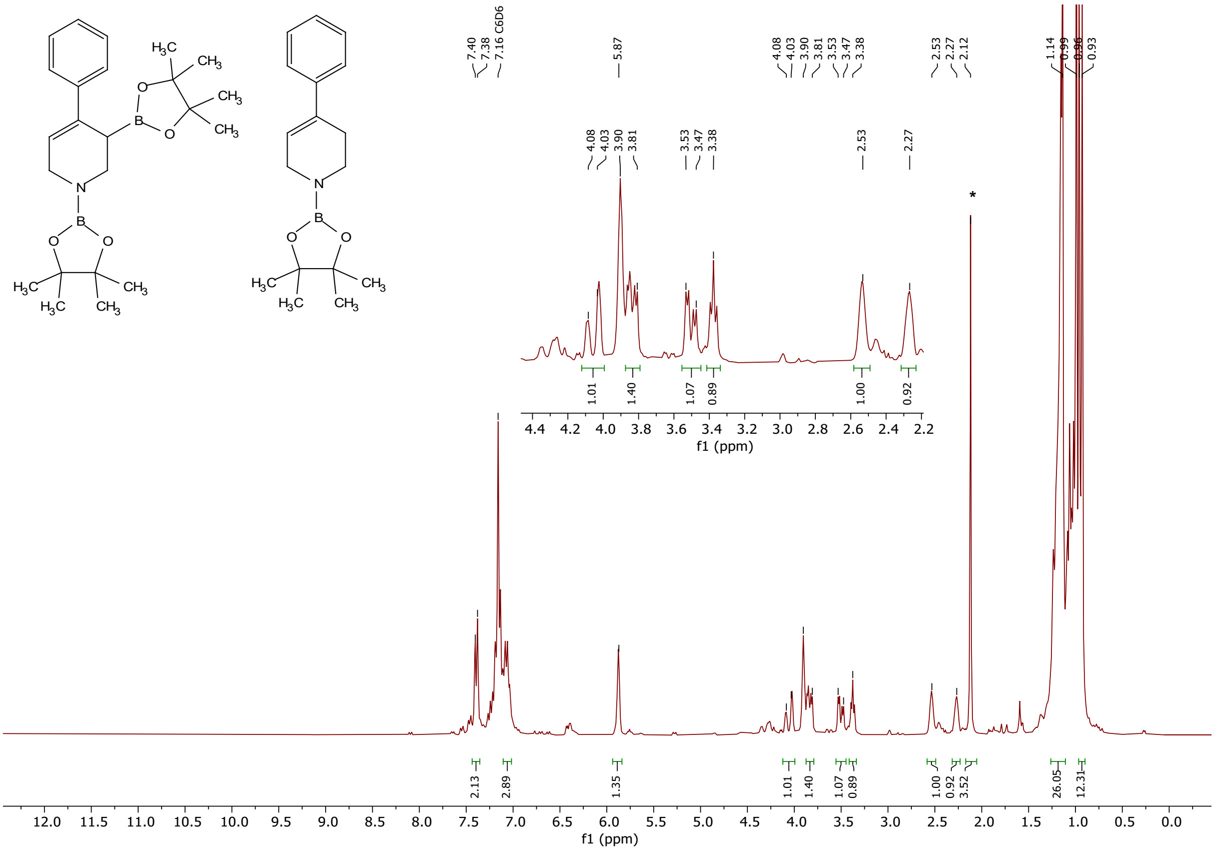

Figure 100: 300 MHz ^{1}H-NMR crude spectrum of the double hydroboration of 4-phenylpyridine using **Co6** (3 mol%) as a catalyst in C_6D_6. (*) Internal standard hexamethylbenzene (0.017 mmol).

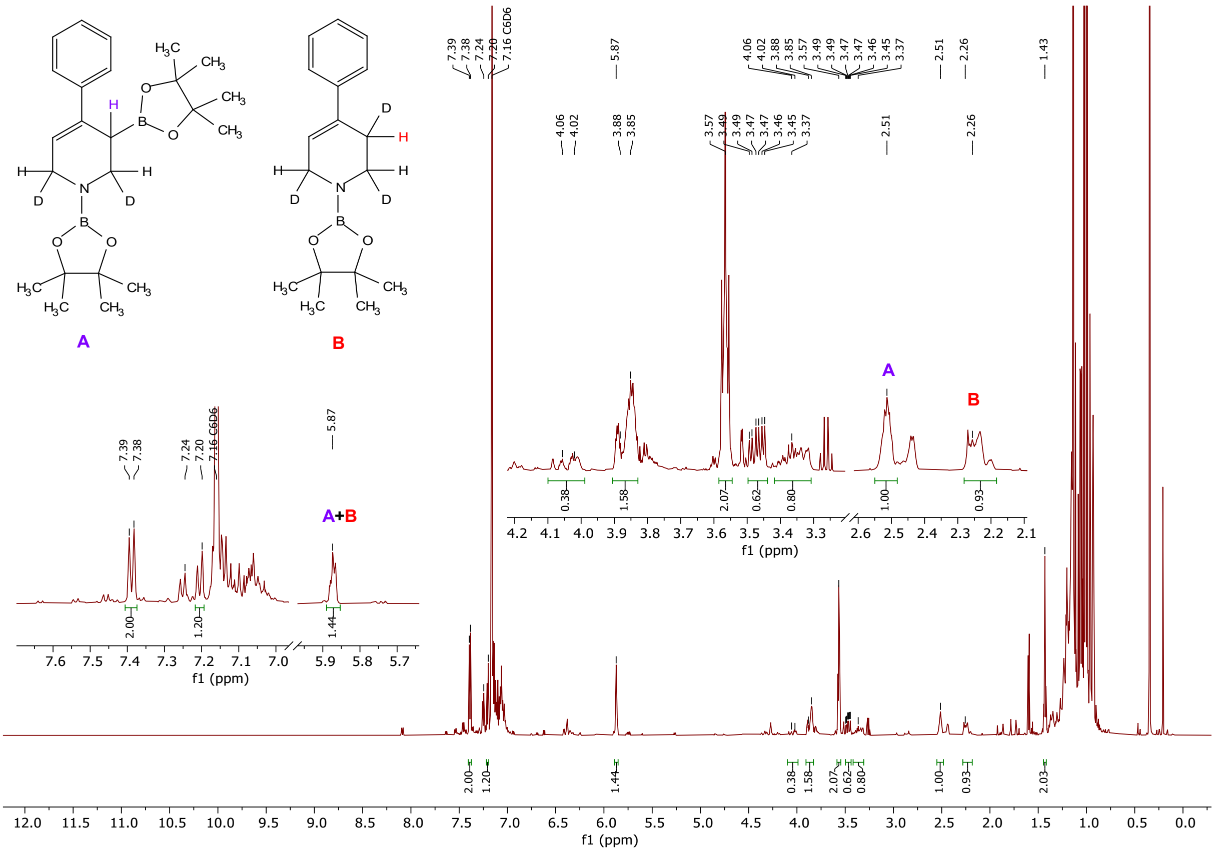

Figure 101: 600 MHz ^{1}H-NMR spectrum of the reaction of 4-phenylpyridine with deuterated pinacolborane in C_6D_6.

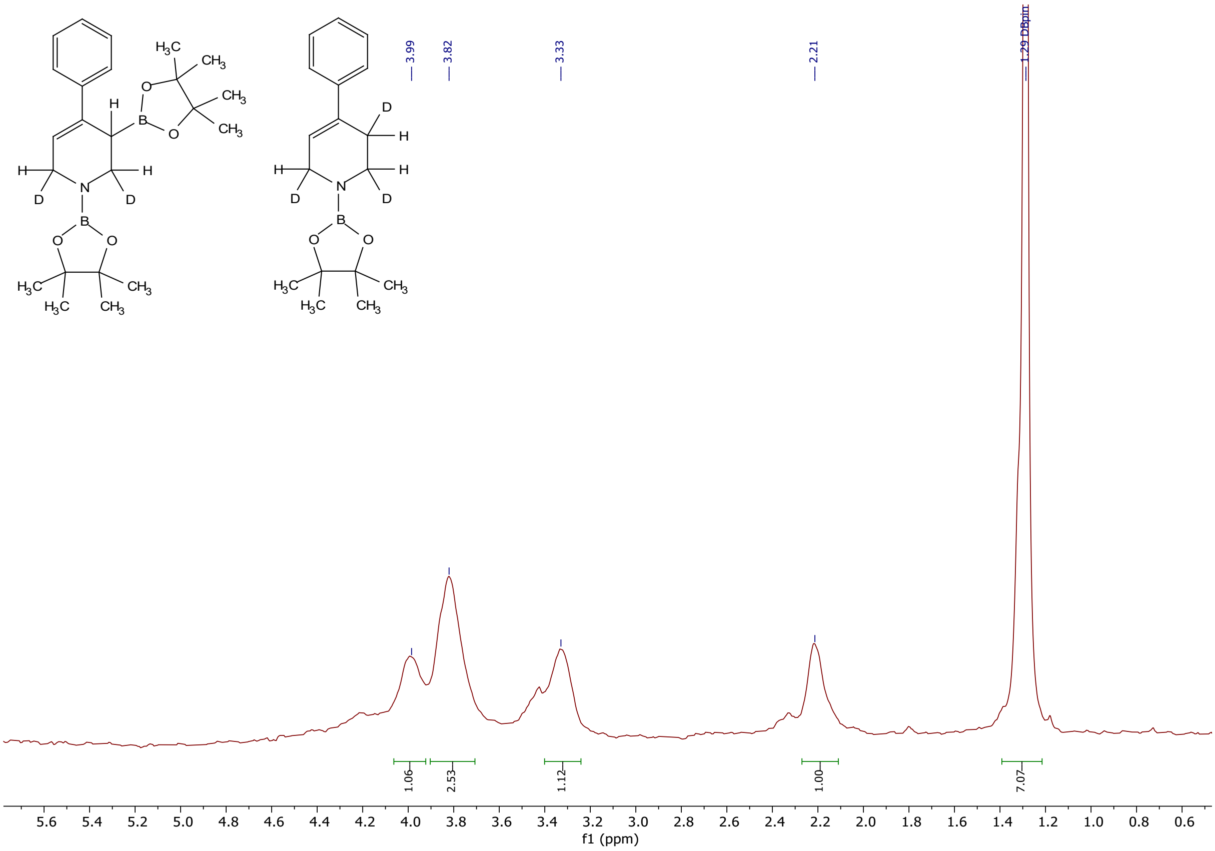

Figure 102: 92 MHz ^{2}H-NMR spectrum of the reaction of 4-phenylpyridine with deuterated pinacolborane in C_6D_6.

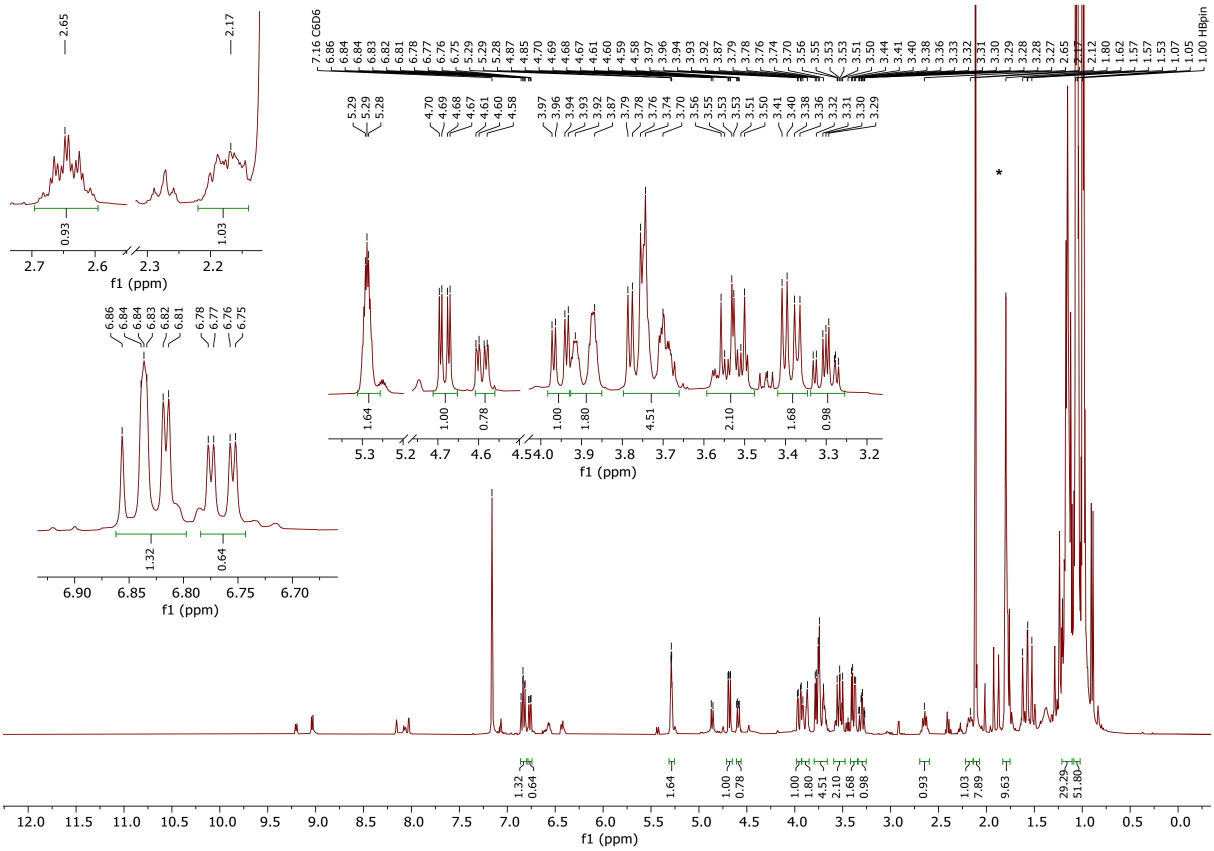

Figure 103: 400 MHz ^{1}H-NMR crude spectra of the double hydroboration of 4-methylpyridine catalysed by **Co3** (3 mol%) in C_6D_6. Apart from **3a**, unidentified products form. (*) Internal standard hexamethylbenzene (0.018 mmol).

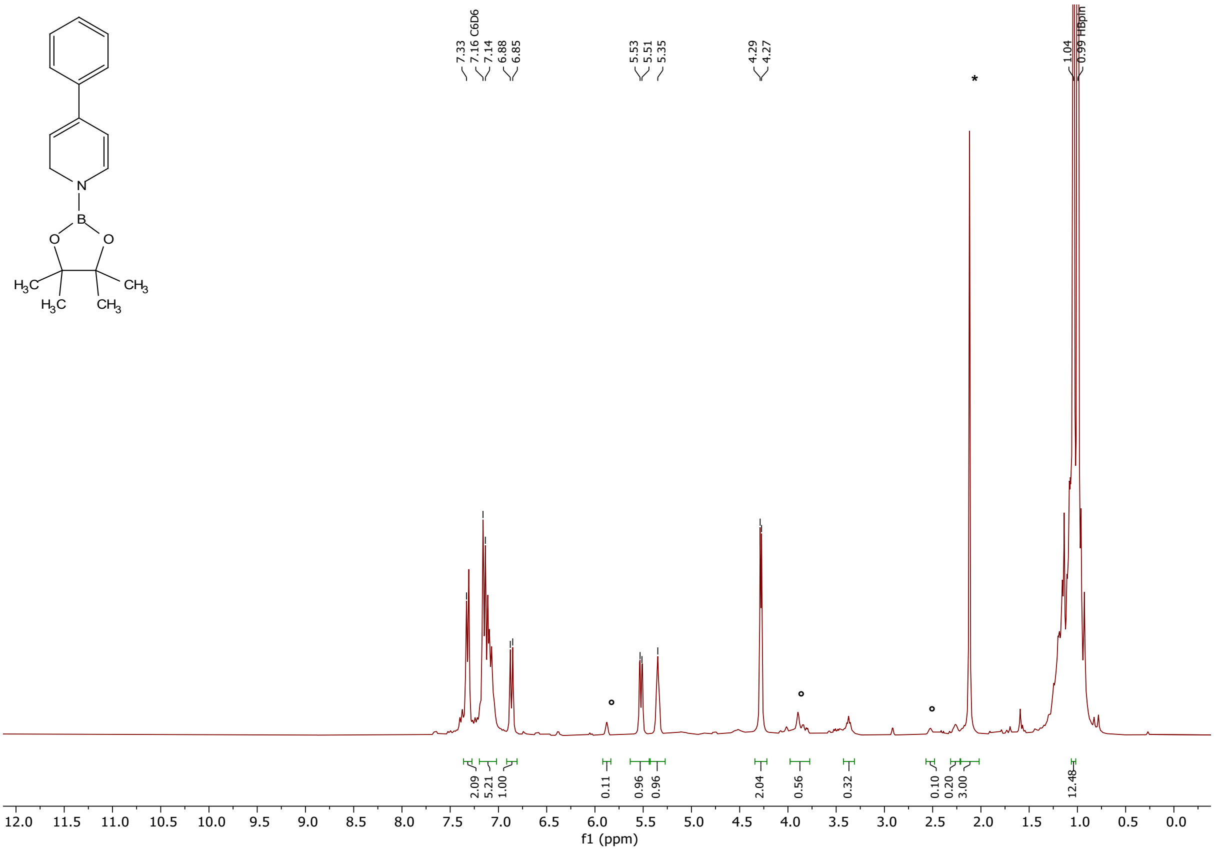

Figure 104: 300 MHz ^{1}H-NMR crude spectra of the double hydroboration of 4-phenylpyridine catalysed by **Co3** (3 mol%) in C_6D_6. *N*-Boryl 1,2-DHP **2b** forms as the main product. (°) Signals of **3b**. (*) Internal standard hexamethylbenzene (0.022 mmol).

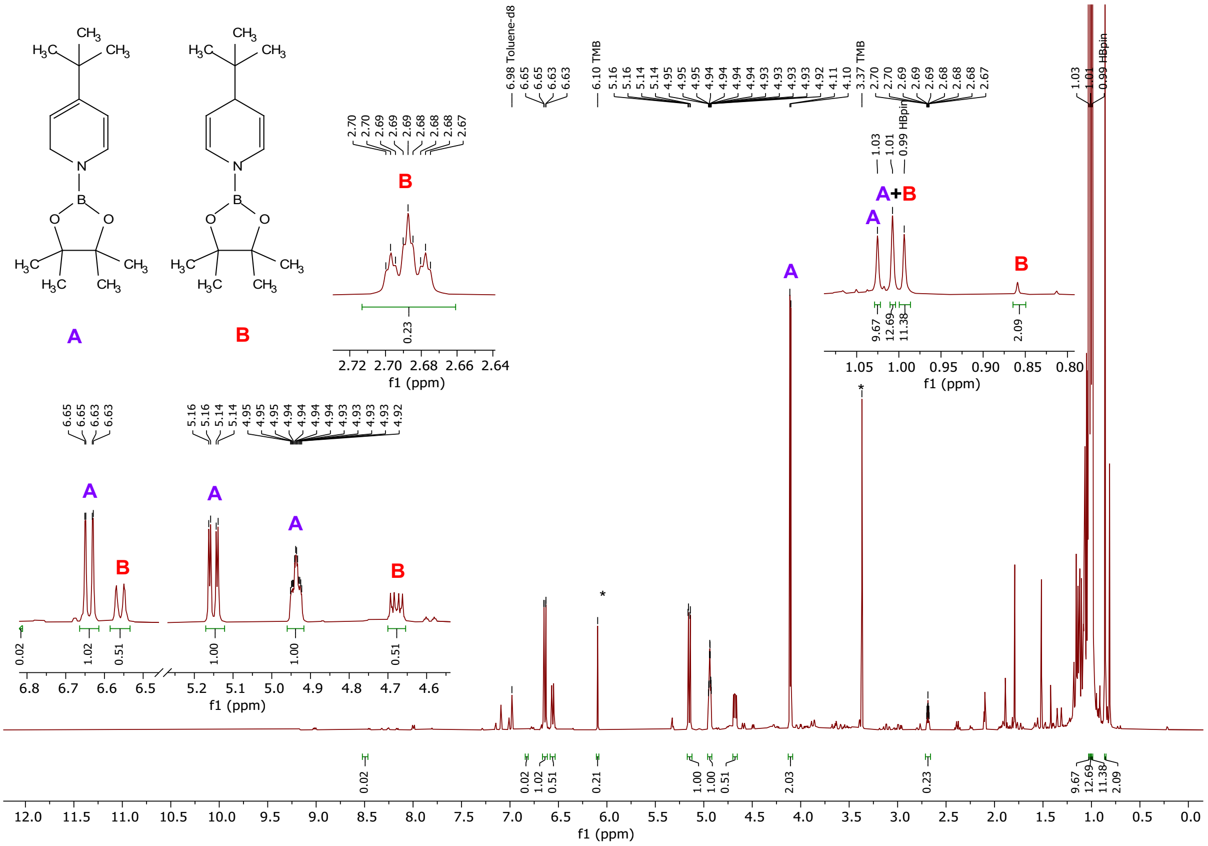

Figure 105: 400 MHz ^{1}H-NMR spectrum of **2y** and **2y'** obtained by the catalytic hydroboration of 4-tert-butyl pyridine in toluene-d_8. (*) Internal standard 1,3,5-trimethoxybenzene (0.02 mmol).

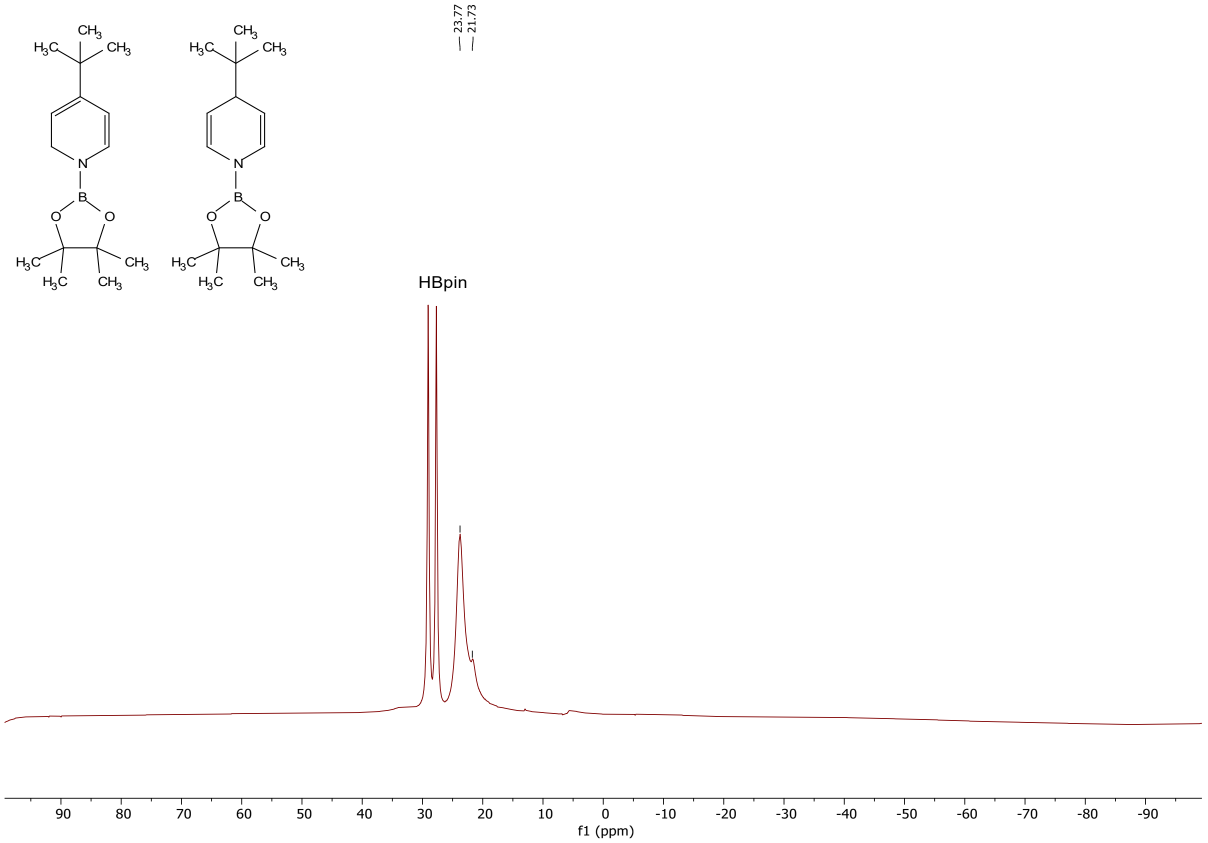

Figure 106: 128 MHz ^{11}B-NMR spectrum of **2y** and **2y'** obtained by the catalytic hydroboration of 4-tert-butyl pyridine in toluene-d_8.

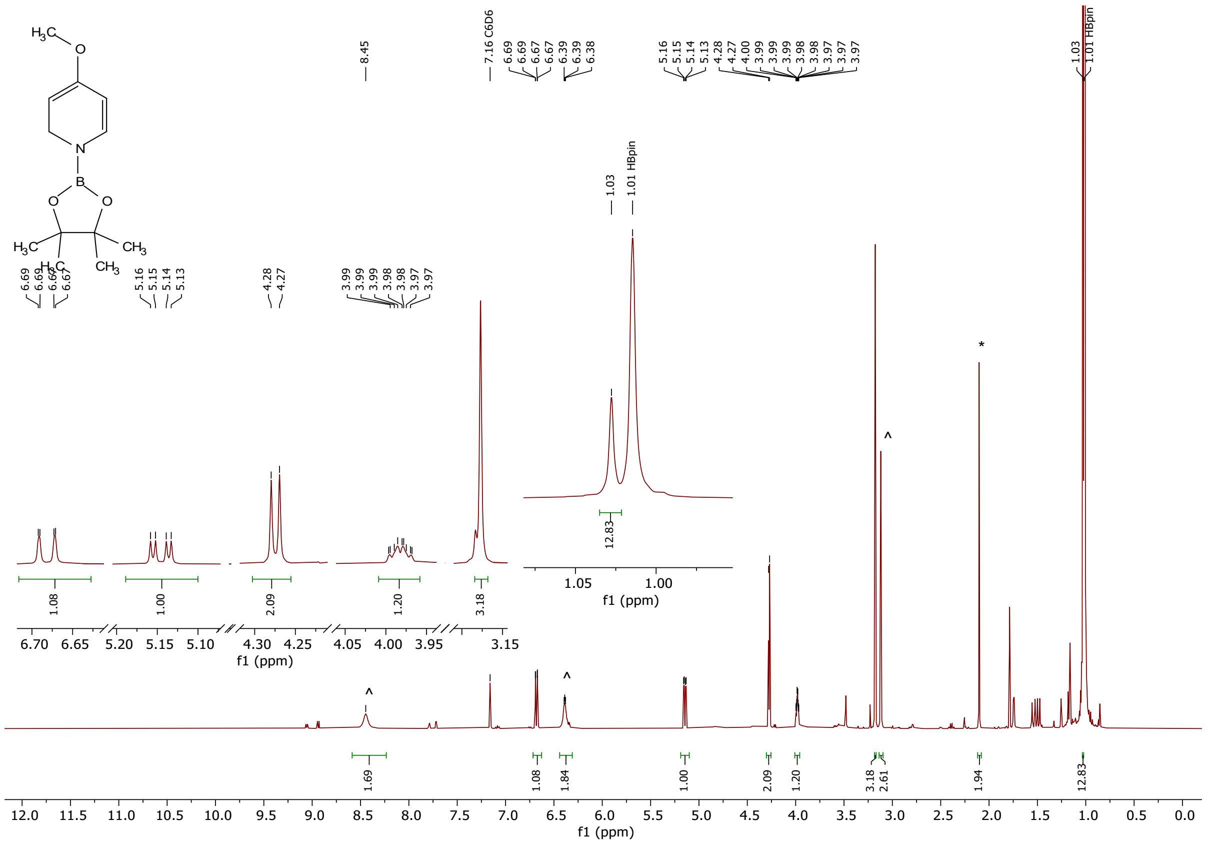

Figure 107: 400 MHz ^{1}H-NMR spectrum of **2z** obtained by the catalytic hydroboration of 4-methoxypyridine in C_6D_6. (^) Signals of 4-methoxypyridine. (*) Internal standard hexamethylbenzene (0.02 mmol).

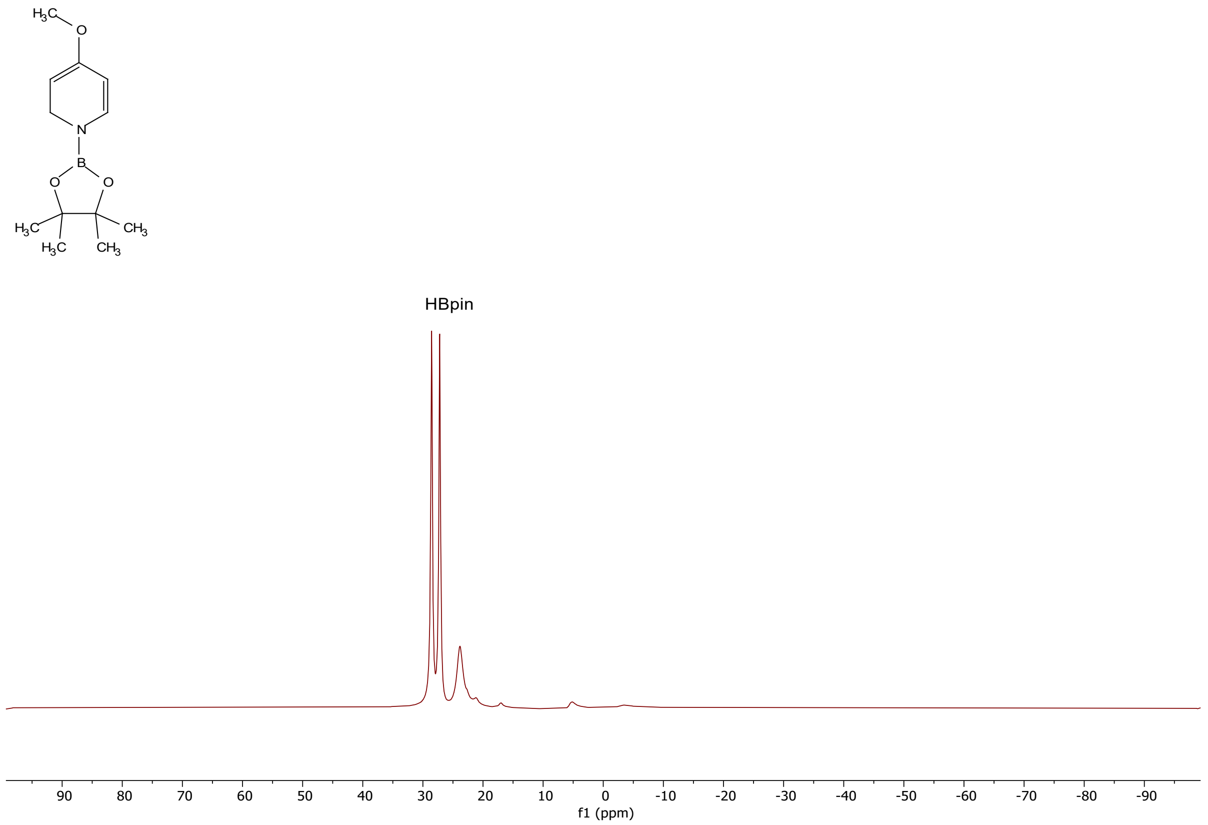

Figure 108: 128 MHz ^{11}B-NMR spectrum of **2z** obtained by the catalytic hydroboration of 4-methoxypyridine in C_6D_6.

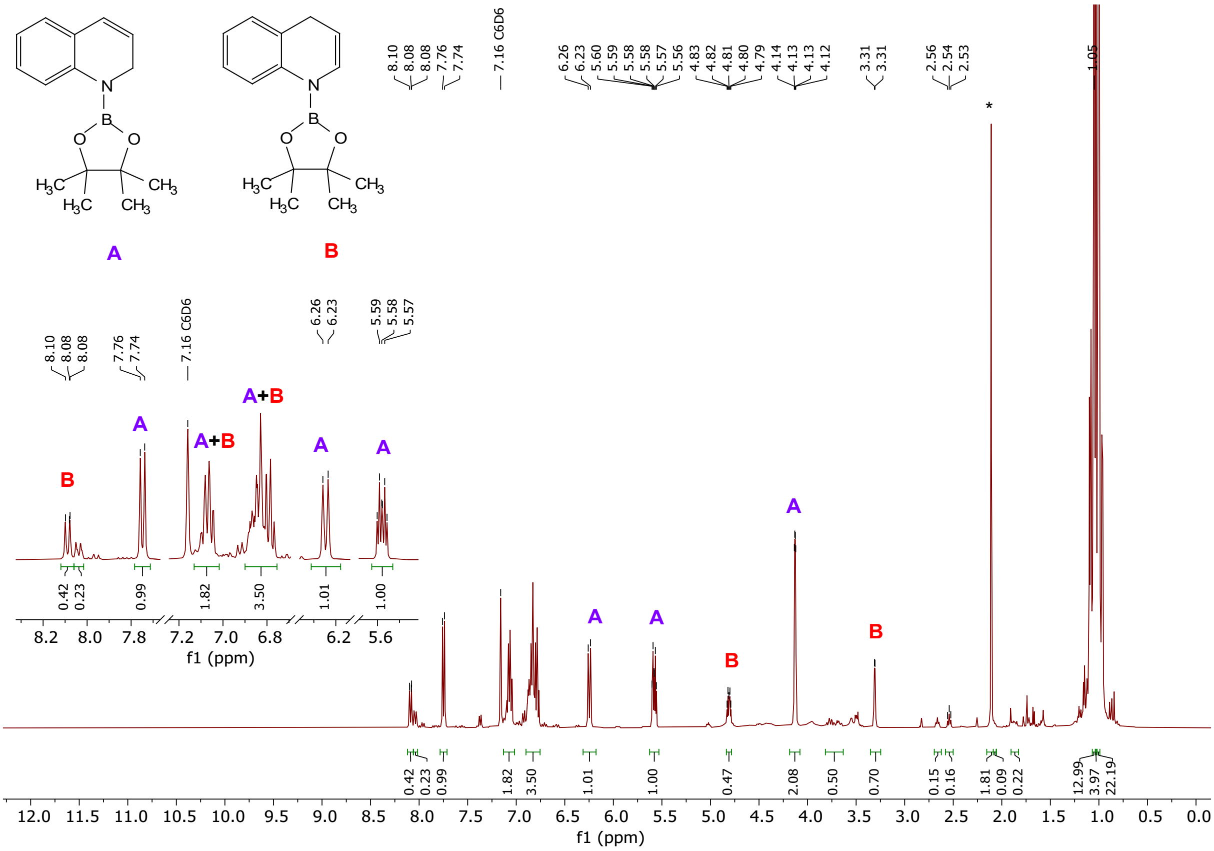

Figure 109: 400 MHz ^{1}H-NMR spectrum of **2o** and **2o’** obtained by the catalytic hydroboration of quinoline in C_6D_6. (*) Internal standard hexamethylbenzene (0.02 mmol).

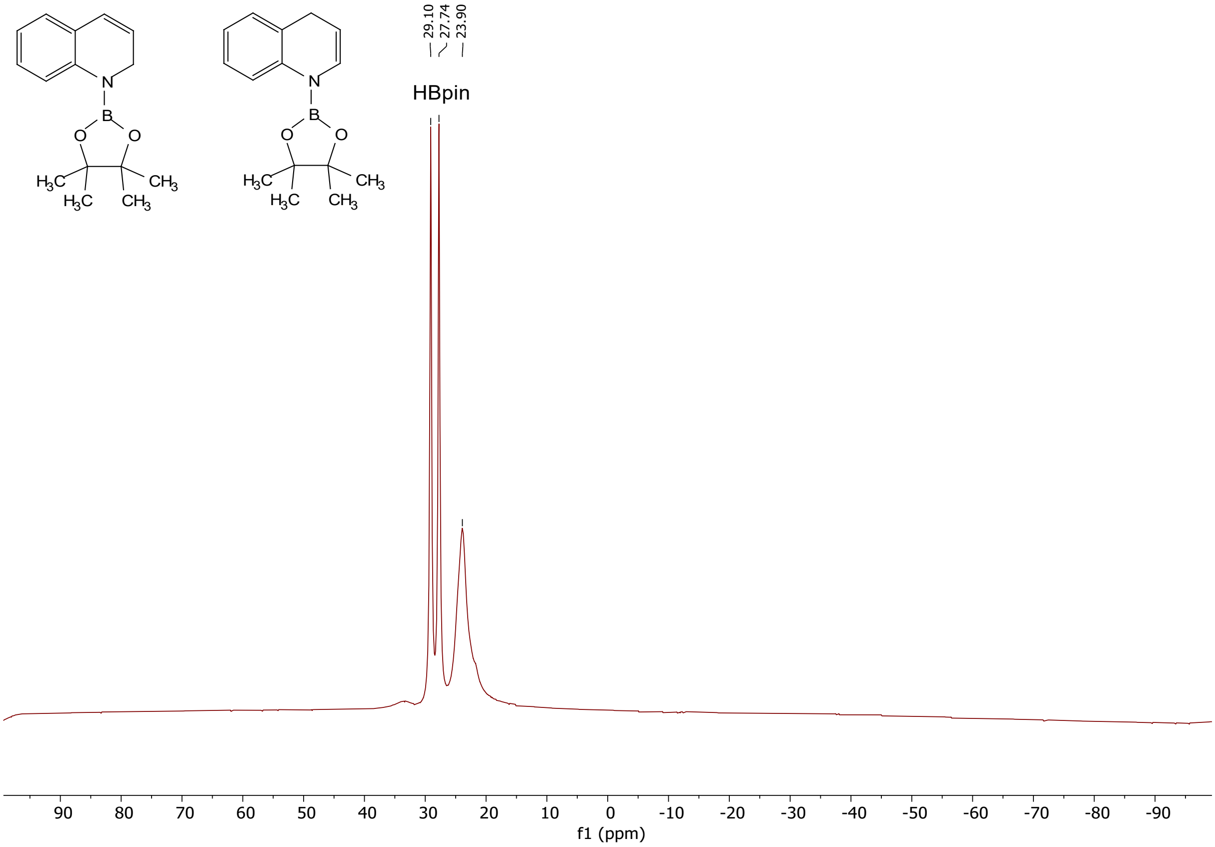

Figure 110: 128 MHz ^{11}B-NMR spectrum of **2o** and **2o’** obtained by the catalytic hydroboration of quinoline in C_6D_6.

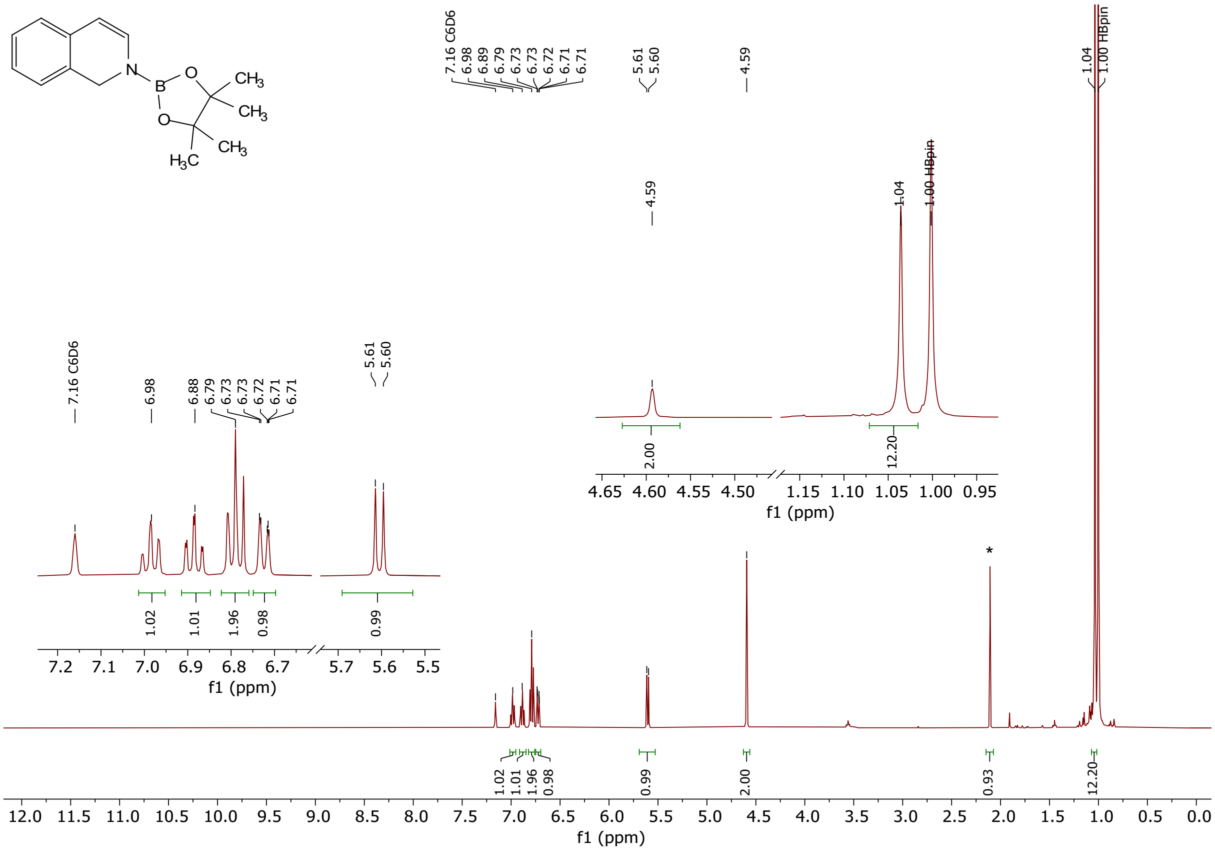

Figure 111: 400 MHz ^{1}H-NMR spectrum of **2p** obtained by the catalytic hydroboration of isoquinoline in C_6D_6. (*) Internal standard hexamethylbenzene (0.02 mmol).

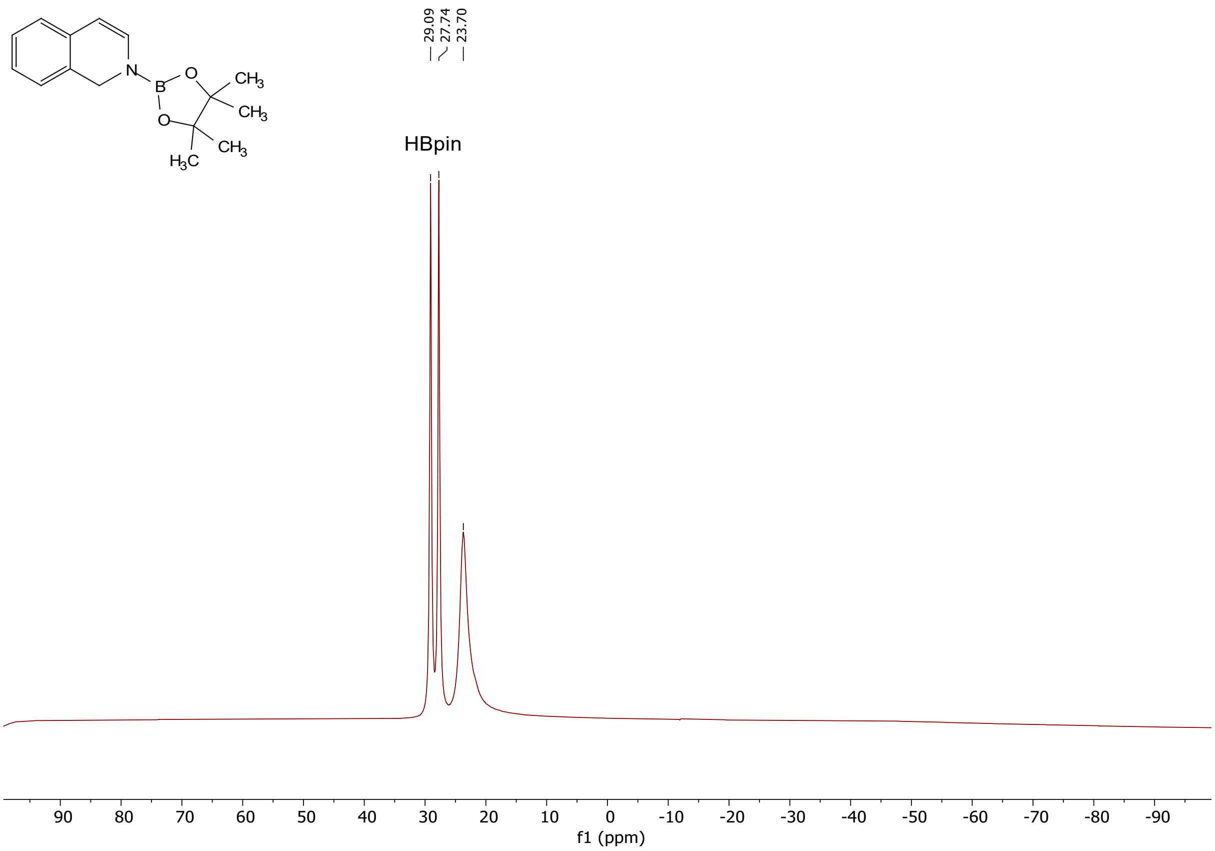

Figure 112: 128 MHz ^{11}B-NMR spectrum of **2p** obtained by the catalytic hydroboration of isoquinoline in C_6D_6.

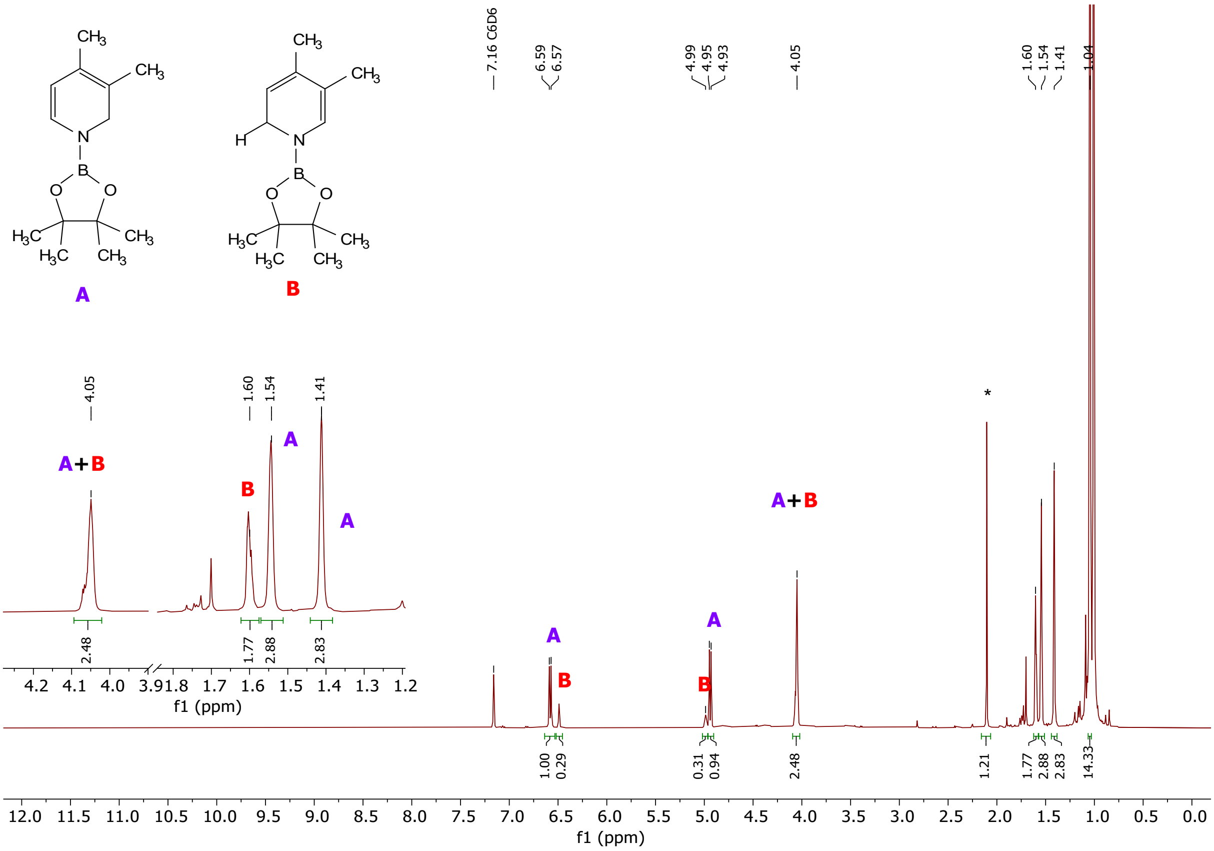

Figure 113: 400 MHz ^{1}H-NMR spectrum of **2q** and **2q'** obtained by the catalytic hydroboration of 3,4-lutidine in C_6D_6. (*) Internal standard hexamethylbenzene (0.02 mmol).

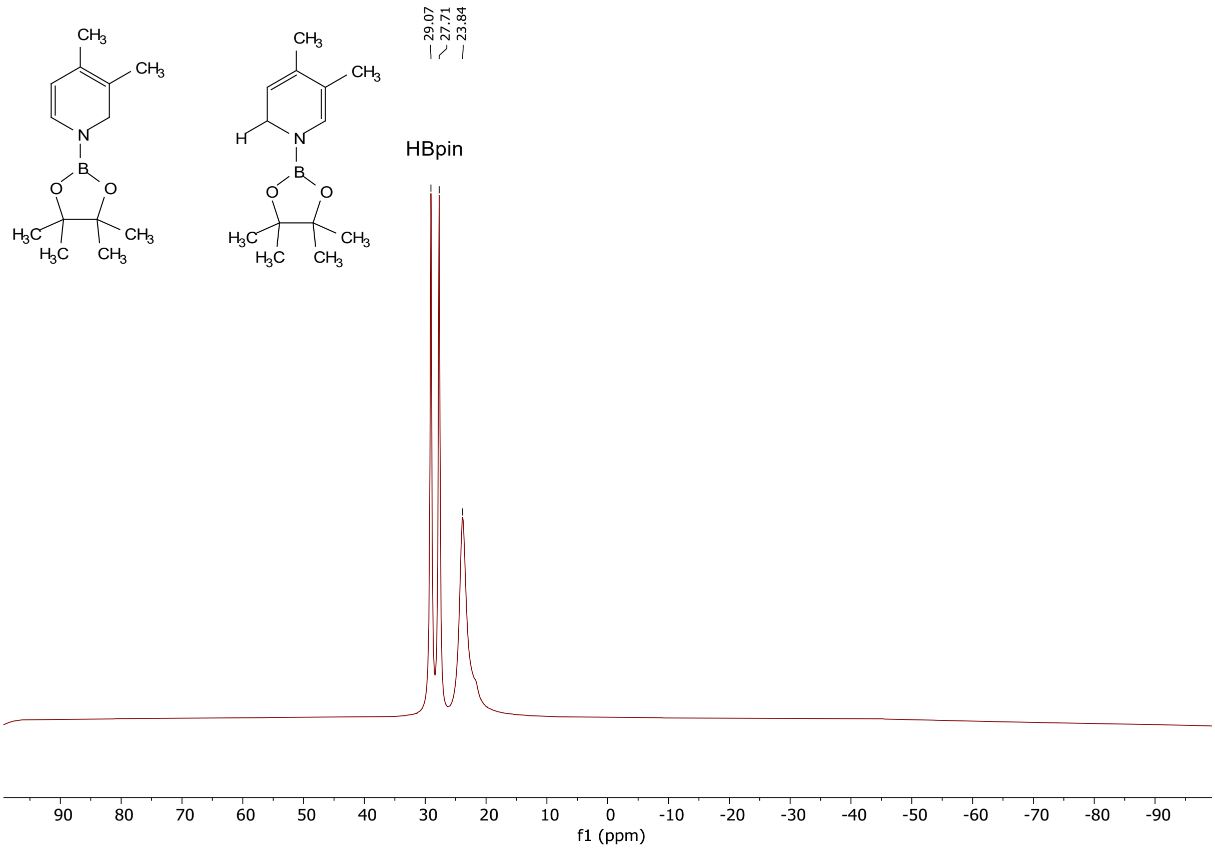

Figure 114: 128 MHz ^{11}B-NMR spectrum of **2q** and **2q'** obtained by the catalytic hydroboration of 3,4-lutidine in C_6D_6.

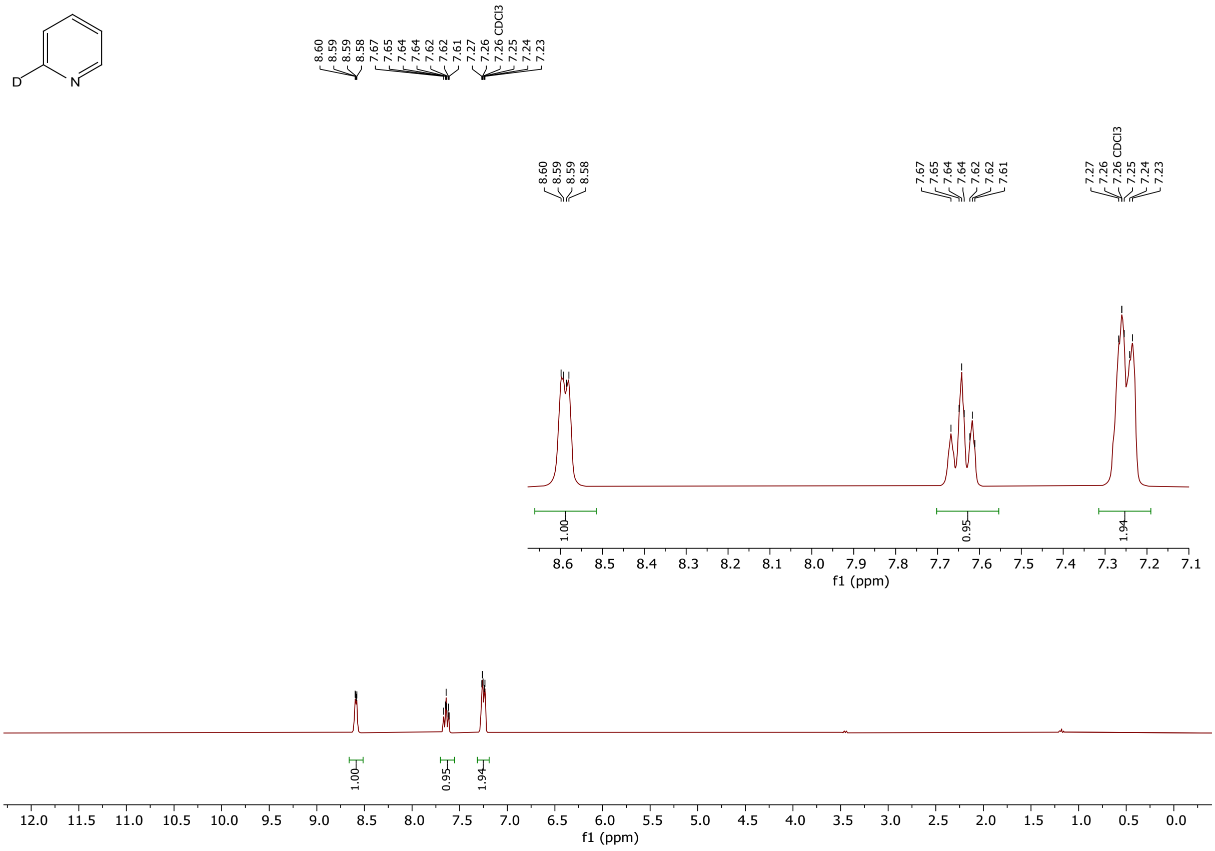

Figure 115: 300 MHz ^{1}H-NMR spectrum of Pyridine-2-d in $CDCl_3$.

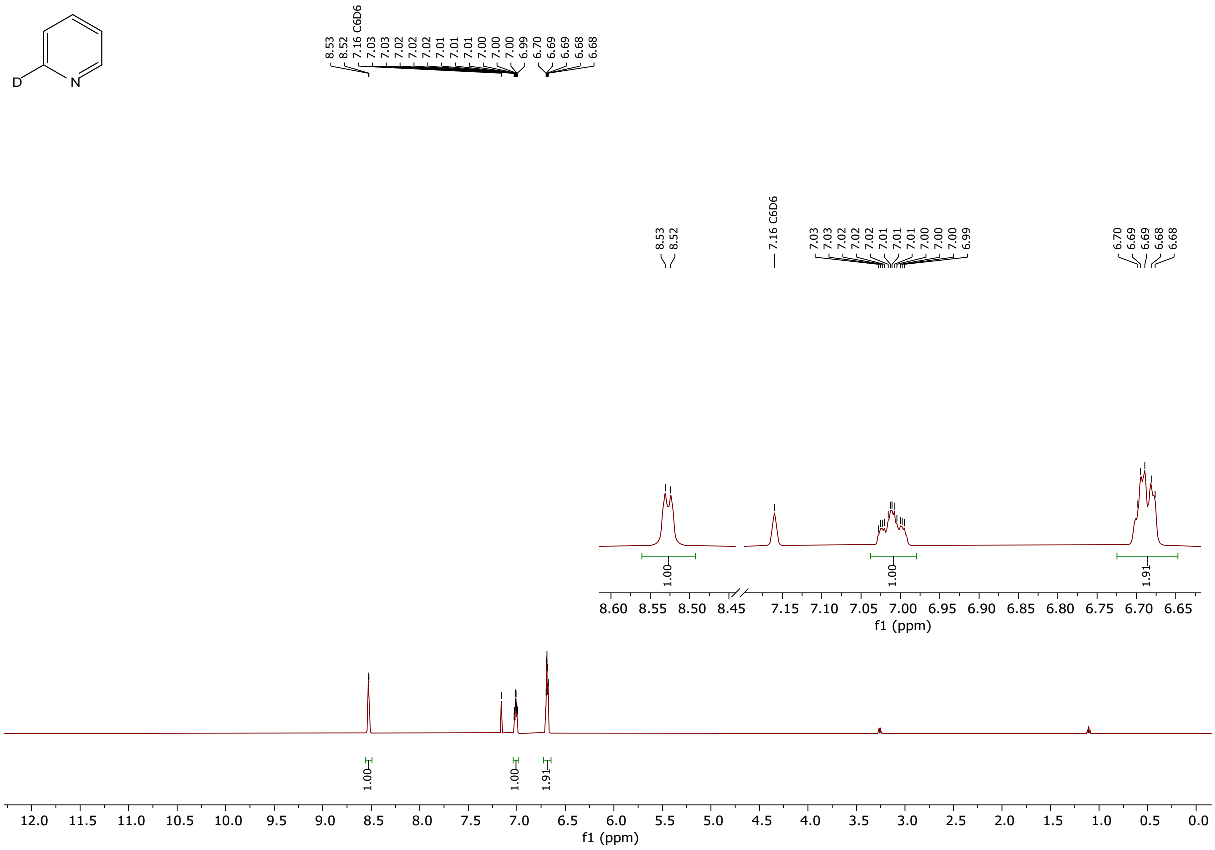

Figure 116: 600 MHz ^{1}H-NMR spectrum of Pyridine-2-d in C_6D_6.

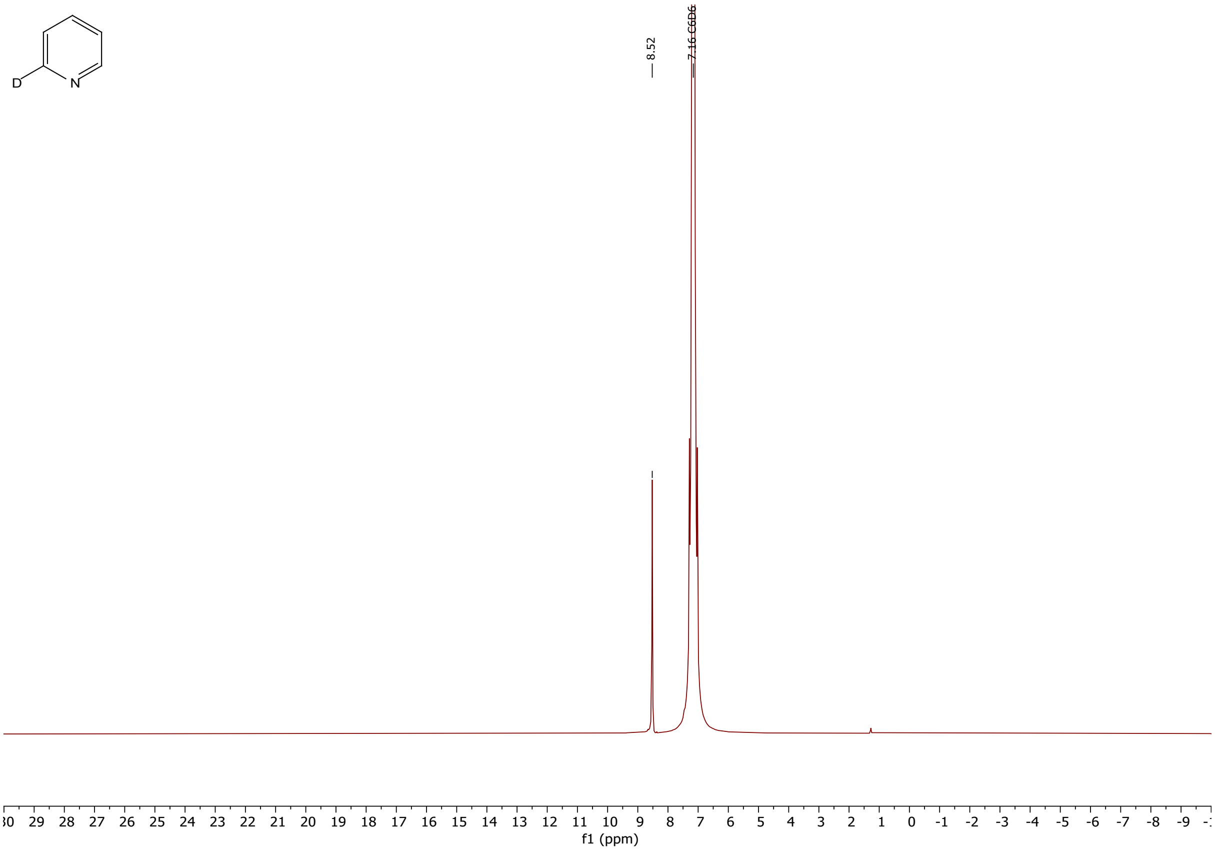

Figure 117: 92 MHz 2H-NMR spectrum of Pyridine-2-d in C_6D_6.

12. X-ray Crystallography Data

Table S2: Crystal data and structure refinement for **Co2**.

Identification code	**Co2**
Empirical formula	$C_{22}H_{34}CoNOP$
Formula weight	418.40
Temperature/K	99.96(18)
Crystal system	orthorhombic
Space group	$Pna2_1$
a/Å	14.7865(5)
b/Å	10.4516(3)
c/Å	13.5026(3)
α/°	90
β/°	90
γ/°	90
Volume/Å^3	2086.73(10)
Z	4
ρ_{calc}g/cm^3	1.332
μ/mm^{-1}	0.910
F(000)	892.0
Crystal size/mm^3	0.35 × 0.22 × 0.18
Radiation	Mo Kα (λ = 0.71073)
2Θ range for data collection/°	6.282 to 59.996
Index ranges	$-19 \le h \le 20, -14 \le k \le 14, -18 \le l \le 18$
Reflections collected	89288
Independent reflections	5397 [R_{int} = 0.0572, R_{sigma} = 0.0229]
Data/restraints/parameters	5397/234/244
Goodness-of-fit on F^2	1.064
Final R indexes [I>=2σ (I)]	R_1 = 0.0531, wR_2 = 0.1122
Final R indexes [all data]	R_1 = 0.0596, wR_2 = 0.1171
Largest diff. peak/hole / e Å^{-3}	2.26/-1.05
Flack parameter	-0.028(6)

Table S3: Crystal data and structure refinement for **Co3**.

Identification code	**Co3**
Empirical formula	$C_{24}H_{38}CoNOP$
Formula weight	446.45
Temperature/K	99.94(17)
Crystal system	monoclinic
Space group	$P2_1/n$
a/Å	22.3080(5)
b/Å	9.2019(2)
c/Å	22.7534(5)
α/°	90
β/°	95.001(2)
γ/°	90
Volume/Å^3	4652.95(18)
Z	8
ρ_{calc}g/cm^3	1.275
μ/mm^{-1}	6.523
F(000)	1912.0
Crystal size/mm^3	0.25 × 0.16 × 0.02
Radiation	Cu Kα (λ = 1.54184)
2Θ range for data collection/°	5.32 to 147.706
Index ranges	$-27 \le h \le 26$, $-11 \le k \le 11$, $-28 \le l \le 28$
Reflections collected	92564
Independent reflections	9286 [R_{int} = 0.1098, R_{sigma} = 0.0427]
Data/restraints/parameters	9286/0/527
Goodness-of-fit on F^2	1.085
Final R indexes [I>=2σ (I)]	R_1 = 0.0570, wR_2 = 0.1529
Final R indexes [all data]	R_1 = 0.0663, wR_2 = 0.1634
Largest diff. peak/hole / e Å^{-3}	0.52/-0.93

Table S4: Crystal data and structure refinement for **Co6**.

Identification code	**Co6**
Empirical formula	$C_{28}H_{46}BNO_3PCo$
Formula weight	545.37
Temperature/K	99.97(13)
Crystal system	triclinic
Space group	P-1
a/Å	8.5688(5)
b/Å	12.8423(5)
c/Å	13.3857(5)
α/°	100.810(3)
β/°	95.593(4)
γ/°	92.022(4)
Volume/Å^3	1437.81(12)
Z	2
ρ_{calc}g/cm^3	1.260
μ/mm^{-1}	5.418
F(000)	584.0
Crystal size/mm^3	0.24 × 0.14 × 0.08
Radiation	CuKα (λ = 1.54184)
2Θ range for data collection/°	6.76 to 153.768
Index ranges	-10 ≤ h ≤ 10, -15 ≤ k ≤ 16, -16 ≤ l ≤ 16
Reflections collected	28557
Independent reflections	5969 [R_{int} = 0.0465, R_{sigma} = 0.0310]
Data/restraints/parameters	5969/0/325
Goodness-of-fit on F^2	1.112
Final R indexes [I>=2σ (I)]	R_1 = 0.0561, wR_2 = 0.1494
Final R indexes [all data]	R_1 = 0.0611, wR_2 = 0.1588
Largest diff. peak/hole / e Å^{-3}	1.92/-1.47

Table S5: Crystal data and structure refinement for **Co8**.

Identification code	**Co8**
Empirical formula	$C_{38}H_{63}BCo_2NO_3P$
Formula weight	741.53
Temperature/K	99.92(18)
Crystal system	monoclinic
Space group	$P2_1/c$
a/Å	16.4812(5)
b/Å	8.4448(2)
c/Å	28.0381(8)
α/°	90
β/°	103.883(3)
γ/°	90
Volume/Å^3	3788.36(19)
Z	4
ρ_{calc}g/cm^3	1.300
μ/mm^{-1}	0.953
F(000)	1584.0
Crystal size/mm^3	0.22 × 0.12 × 0.08
Radiation	Mo Kα (λ = 0.71073)
2Θ range for data collection/°	5.05 to 59.24
Index ranges	-22 ≤ h ≤ 22, -11 ≤ k ≤ 11, -38 ≤ l ≤ 38
Reflections collected	127400
Independent reflections	10251 [R_{int} = 0.0397, R_{sigma} = 0.0214]
Data/restraints/parameters	10251/0/441
Goodness-of-fit on F^2	1.100
Final R indexes [I>=2σ (I)]	R_1 = 0.0489, wR_2 = 0.1291
Final R indexes [all data]	R_1 = 0.0583, wR_2 = 0.1352
Largest diff. peak/hole / e Å^{-3}	0.89/-1.37

Table S6: Crystal data and structure refinement for **6**.

Identification code	**6**
Empirical formula	$C_{15}H_{19}NO_2$
Formula weight	245.31
Temperature/K	99.95(13)
Crystal system	monoclinic
Space group	C2
a/Å	14.1533(4)
b/Å	6.0972(2)
c/Å	15.4812(4)
α/°	90
β/°	100.501(2)
γ/°	90
Volume/Å^3	1313.58(7)
Z	4
ρ_{calc}g/cm^3	1.240
μ/mm^{-1}	0.652
F(000)	528.0
Crystal size/mm^3	0.24 × 0.06 × 0.05
Radiation	CuKα (λ = 1.54184)
2Θ range for data collection/°	5.806 to 153.02
Index ranges	-17 ≤ h ≤ 16, -7 ≤ k ≤ 7, -19 ≤ l ≤ 19
Reflections collected	13446
Independent reflections	2734 [R_{int} = 0.0448, R_{sigma} = 0.0286]
Data/restraints/parameters	2734/1/169
Goodness-of-fit on F^2	1.059
Final R indexes [I>=2σ (I)]	R_1 = 0.0627, wR_2 = 0.1533
Final R indexes [all data]	R_1 = 0.0656, wR_2 = 0.1588
Largest diff. peak/hole / e Å^{-3}	0.51/-0.22
Flack parameter	0.45(19)

13. References

[1] F. Jiang, M. Achard, T. Roisnel, V. Dorcet, C. Bruneau, *Eur. J. Inorg. Chem.* **2015**, *2015*, 4312–4317.

[2] X. Zhuang, J. Y. Chen, Z. Yang, M. Jia, C. Wu, R. Z. Liao, C. H. Tung, W. Wang, *Organometallics* **2019**, *38*, 3752–3759.

[3] A. M. Bryan, G. J. Long, F. Grandjean, P. P. Power, *Inorg. Chem.* **2013**, *52*, 12152–12160.

[4] S. K. Sur, *J. Magn. Reson.* **1989**, *82*, 169–173.

[5] Agilent, *Technol. UK Ltd, Yarnton, Oxford, UK* **2014**, *44*, 1–53.

[6] B. A. Inc., **2014**.

[7] G. M. Sheldrick, *Acta Crystallogr. Sect. A Found. Crystallogr.* **2008**, *64*, 112–122.

[8] G. M. Sheldrick, *Acta Crystallogr. Sect. A Found. Crystallogr.* **2015**, *71*, 3–8.

[9] A. L. Spek, *Acta Crystallogr. Sect. D Biol. Crystallogr.* **2009**, *65*, 148–155.

[10] J. W. Brandt, EARLY AND LATE TRANSITION METAL COMPLEXES FEATURING 1,3-N,O CHELATES: DEVELOPMENT OF HOMOGENOUS CATALYSTS FOR THE α- ALKYLATION OF AMINES VIA HYDROAMINOALKYLATION, The University of British Columbia, **2018**.

[11] U. Kölle, F. Khouzami, B. Fuss, *Angew. Chem. Suppl.* **1982**, 230–240.

[12] U. Koelle, B. Fuss, M. Belting, E. Raabe, *Organometallics* **1986**, *5*, 980–987.

[13] X. Shi, S. Li, L. Wu, *Angew. Chemie - Int. Ed.* **2019**, *58*, 16167–16171.

[14] H. Choi, R. Wang, S. Kim, D. Kim, M. H. Baik, S. Park, *Catal. Sci. Technol.* **2023**, DOI 10.1039/d3cy00347g.

[15] J. Liu, J. Y. Chen, M. Jia, B. Ming, J. Jia, R. Z. Liao, C. H. Tung, W. Wang, *ACS Catal.* **2019**, *9*, 3849–3857.

[16] F. Zhang, H. Song, X. Zhuang, C. H. Tung, W. Wang, *J. Am. Chem. Soc.* **2017**, *139*, 17775–17778.

[17] P. Ghosh, A. Jacobi von Wangelin, *Angew. Chemie - Int. Ed.* **2021**, *60*, 16035–16043.

[18] K. Oshima, T. Ohmura, M. Suginome, *J. Am. Chem. Soc.* **2012**, *134*, 3699–3702.

[19] A. S. Dudnik, V. L. Weidner, A. Motta, M. Delferro, T. J. Marks, *Nat. Chem.* **2014**, *6*, 1100–1107.

[20] N. K. Meher, P. K. Verma, K. Geetharani, *Org. Lett.* **2023**, *25*, 87–92.

[21] M. T. Huggins, T. Kesharwani, J. Buttrick, C. Nicholson, *J. Chem. Educ.* **2020**, *97*, 1425–1429.

[22] H. L. D. Hayes, R. Wei, M. Assante, K. J. Geogheghan, N. Jin, S. Tomasi, G. Noonan, A. G. Leach, G. C. Lloyd-Jones, *J. Am. Chem. Soc.* **2021**, *143*, 14814–14826.

[23] A. D. Bage, K. Nicholson, T. A. Hunt, T. Langer, S. P. Thomas, *ACS Catal.* **2020**, *10*, 13479–13486.

[24] A. D. Bage, T. A. Hunt, S. P. Thomas, *Org. Lett.* **2020**, *22*, 4107–4112.

[25] S. Bera, C. Fan, X. Hu, *Nat. Catal.* **2022**, *5*, 1180–1187.

[26] H. Iwamoto, Y. Ozawa, Y. Takenouchi, T. Imamoto, H. Ito, *J. Am. Chem. Soc.* **2021**, *143*, 6413–6422.

[27] D. Imao, B. W. Glasspoole, V. S. Laberge, C. M. Crudden, *J. Am. Chem. Soc.* **2009**, *131*, 5024–5025.

[28] C. C. Bories, G. Gontard, M. Barbazanges, G. Lemière, M. Petit, *Chem. - A Eur. J.* **2023**, *29*, e202303178.

[29] R. Davidson, Y. T. Hsu, T. Batchelor, D. Yufit, A. Beeby, *Dalt. Trans.* **2016**, *45*, 11496–11507.

[30] W. Nguyen, B. L. Howard, D. P. Jenkins, H. Wulff, P. E. Thompson, D. T. Manallack, *Bioorganic Med. Chem. Lett.* **2012**, *22*, 7106–7109.

[31] P. Ghosh, R. Schoch, M. Bauer, A. Jacobi von Wangelin, *Angew. Chemie - Int. Ed.* **2022**, *61*, DOI 10.1002/anie.202110821.

[32] B. Liu, Y. Huang, J. Lan, F. Song, J. You, *Chem. Sci.* **2013**, *4*, 2163–2167.
